# Supplementary material for: Researching COVID to enhance recovery (RECOVER) pediatric study protocol: Rationale, objectives and design
Source: PLoS One. 2024 May 7;19(5):e0285635. doi: 10.1371/journal.pone.0285635 (PMC11075869; doi:10.1371/journal.pone.0285635)
Supplement: S1 Table — (DOCX) [file pone.0285635.s002.docx]

### S1 Table: Hubs and enrolling sites

| Cohort Type | Hub Name | Enrolling Sites | Location |
| --- | --- | --- | --- |
| *de novo* pediatric RECOVER cohort | Arkansas Children’s Research Institute | Arkansas Children’s Research Institute | Arkansas |
|  | Arkansas Children’s Research Institute | Avera Research Institute | South Dakota |
|  | Arkansas Children’s Research Institute | Dartmouth Hitchcock Medical Center | New Hampshire |
|  | Arkansas Children’s Research Institute | Kapiolani Medical Center for Women and Children | Hawaii |
|  | Arkansas Children’s Research Institute | Medical University of South Carolina | South Carolina |
|  | Arkansas Children’s Research Institute | Nemours Children’s Health System | Delaware |
|  | Arkansas Children’s Research Institute | Northeastern University, Puerto Rico Testsite | Puerto Rico |
|  | Arkansas Children’s Research Institute | Pennington Biomedical Research Center | Louisiana |
|  | Arkansas Children’s Research Institute | University of Louisville Research Foundation | Kentucky |
|  | Arkansas Children’s Research Institute | University of Nebraska Medical Center | Nebraska |
|  | Arkansas Children’s Research Institute | University of New Mexico Health Sciences Center | New Mexico |
|  | Arkansas Children’s Research Institute | University of Oklahoma Health Sciences Center | Oklahoma |
|  | Arkansas Children’s Research Institute | University of Vermont Medical Center | Vermont |
|  | Arkansas Children’s Research Institute | West Virginia University | West Virginia |
|  | Children’s Hospital of Los Angeles | Children’s Hospital of Los Angeles | California |
|  | Columbia University College of Physicians & Surgeons | Columbia University College of Physicians & Surgeons | New York |
|  | Columbia University College of Physicians & Surgeons | Best Healthcare Inc. | New York |
|  | Rutgers Robert Wood Johnson Medical Center | Rutgers Robert Wood Johnson Medical School | New Jersey |
|  | Rutgers Robert Wood Johnson Medical Center | American Academy of Pediatrics | National reach |
|  | Rutgers Robert Wood Johnson Medical Center | Children's Mercy Kansas City | Missouri |
|  | Rutgers Robert Wood Johnson Medical Center | Connecticut Children's Medical Center | Connecticut |
|  | Rutgers Robert Wood Johnson Medical Center | DARTNet Institute | National reach |
|  | Rutgers Robert Wood Johnson Medical Center | Hackensack Meridian Health Hospitals Corporation | New Jersey |
|  | Rutgers Robert Wood Johnson Medical Center | The MetroHealth System | Ohio |
|  | Rutgers Robert Wood Johnson Medical Center | New York Medical Center, Westchester Medical Center | New York |
|  | Rutgers Robert Wood Johnson Medical Center | Saint Barnabas Medical Center | New Jersey |
|  | Rutgers Robert Wood Johnson Medical Center | Yale School of Medicine | Connecticut |
|  | University of California San Diego/Rady Children’s Hospital | University of California San Diego/Rady Children’s Hospital | California |
|  | Virginia Commonwealth University | Virginia Commonwealth University | Virginia |
|  | Virginia Commonwealth University | New York University Grossman School of Medicine | New York |
|  | Virginia Commonwealth University | Rhode Island Hospital | Rhode Island |
| Adolescent Brain Cognitive Development (ABCD) | University of California San Diego | University of California San Diego | California |
|  | University of California San Diego | Children's Hospital, Los Angeles | California |
|  | University of California San Diego | Florida International University | Florida |
|  | University of California San Diego | Laureate Institute for Brain Research | Oklahoma |
|  | University of California San Diego | Medical University of South Carolina | South Carolina |
|  | University of California San Diego | Oregon Health & Science University | Oregon |
|  | University of California San Diego | SRI International | California |
|  | University of California San Diego | University of California, Los Angeles | California |
|  | University of California San Diego | University of Colorado Boulder | Colorado |
|  | University of California San Diego | University of Florida | Florida |
|  | University of California San Diego | University of Maryland Baltimore | Maryland |
|  | University of California San Diego | University of Michigan | Michigan |
|  | University of California San Diego | University of Minnesota | Minnesota |
|  | University of California San Diego | University of Pittsburgh Medical Center | Pennsylvania |
|  | University of California San Diego | University of Rochester | New York |
|  | University of California San Diego | University of Utah | Utah |
|  | University of California San Diego | University of Vermont | Vermont |
|  | University of California San Diego | University of Wisconsin, Milwaukee | Wisconsin |
|  | University of California San Diego | Virginia Commonwealth University | Virginia |
|  | University of California San Diego | Washington University St. Louis | Missouri |
|  | University of California San Diego | Yale University | Connecticut |
| COVID MUSIC Study | Carelon Research | Ann & Robert Lurie Children's Hosp | Illinois |
|  | Carelon Research | Baylor/Texas Children’s Hospital | Texas |
|  | Carelon Research | Boston Children’s Hospital | Massachusetts |
|  | Carelon Research | Children’s Healthcare of Atlanta | Georgia |
|  | Carelon Research | Children’s Hospital of Colorado | Colorado |
|  | Carelon Research | Children’s Hospital Los Angeles | California |
|  | Carelon Research | Children’s Hospital of Michigan | Michigan |
|  | Carelon Research | Children’s Hospital of New Orleans | Louisiana |
|  | Carelon Research | Children's Hospital of Philadelphia (CHOP) | Pennsylvania |
|  | Carelon Research | Children’s Mercy Hospital | Missouri |
|  | Carelon Research | Children's National Hospital | Washington DC |
|  | Carelon Research | Cincinnati Children’s Hospital Medical Center | Ohio |
|  | Carelon Research | Cohen Children’s Medical Center | New York |
|  | Carelon Research | CS Mott Children’s Hospital/University of Michigan | Michigan |
|  | Carelon Research | Dell Children’s Medical Center | Texas |
|  | Carelon Research | Hospital for Sick Children, Toronto | Toronto |
|  | Carelon Research | Joe DiMaggio Children’s Hospital | Florida |
|  | Carelon Research | Medical College of Wisconsin, Children's Hospital | Wisconsin |
|  | Carelon Research | Medical University of South Carolina | South Carolina |
|  | Carelon Research | Morgan Stanley Children's Hospital | New York |
|  | Carelon Research | Nemours, Alfred I. duPont Hospital for Children | Delaware |
|  | Carelon Research | Phoenix Children’s Hospital | Arizona |
|  | Carelon Research | Primary Children’s Hospital/University of Utah | Utah |
|  | Carelon Research | Rady Children’s Hospital | California |
|  | Carelon Research | Riley Children’s Hospital | Indiana |
|  | Carelon Research | Seattle Children’s Hospital | Washington |
|  | Carelon Research | University of Alabama | Alabama |
|  | Carelon Research | University of Mississippi | Mississippi |
|  | Carelon Research | University of New Mexico | New Mexico |
|  | Carelon Research | UT Southwestern, Children's Health Dallas | Texas |
|  | Carelon Research | Valley Children's Healthcare and Hospital | California |
| In-utero exposure cohort | University of California San Francisco | University of California San Francisco, | California and nationally through home visits |
|  | NICHD Maternal-Fetal Medicine Units (MFMU) Network | University of Utah | Utah |
|  | NICHD Maternal-Fetal Medicine Units (MFMU) Network | Brown University Women and Infants Hospital | Rhode Island |
|  | NICHD Maternal-Fetal Medicine Units (MFMU) Network | ChristianaCare Health | Delaware |
|  | NICHD Maternal-Fetal Medicine Units (MFMU) Network | Columbia University | New York |
|  | NICHD Maternal-Fetal Medicine Units (MFMU) Network | Duke University Medical Center | North Carolina |
|  | NICHD Maternal-Fetal Medicine Units (MFMU) Network | The MetroHealth System | Ohio |
|  | NICHD Maternal-Fetal Medicine Units (MFMU) Network | Miami Valley Hospital | Ohio |
|  | NICHD Maternal-Fetal Medicine Units (MFMU) Network | New York-Presbyterian, Queens | New York |
|  | NICHD Maternal-Fetal Medicine Units (MFMU) Network | NorthShore University HealthSystem | Illinois |
|  | NICHD Maternal-Fetal Medicine Units (MFMU) Network | Northwestern University | Illinois |
|  | NICHD Maternal-Fetal Medicine Units (MFMU) Network | Ohio State University | Ohio |
|  | NICHD Maternal-Fetal Medicine Units (MFMU) Network | Saint Peter's University Hospital | New Jersey |
|  | NICHD Maternal-Fetal Medicine Units (MFMU) Network | UH MacDonald's Women's Hospital | Ohio |
|  | NICHD Maternal-Fetal Medicine Units (MFMU) Network | University of Alabama at Birmingham | Alabama |
|  | NICHD Maternal-Fetal Medicine Units (MFMU) Network | University of Colorado | Colorado |
|  | NICHD Maternal-Fetal Medicine Units (MFMU) Network | University of North Carolina, Chapel Hill | North Carolina |
|  | NICHD Maternal-Fetal Medicine Units (MFMU) Network | University of Pennsylvania | Pennsylvania |
|  | NICHD Maternal-Fetal Medicine Units (MFMU) Network | University of Pittsburgh | Pennsylvania |
|  | NICHD Maternal-Fetal Medicine Units (MFMU) Network | University of Texas HSC at Houston, Memorial Herma | Texas |
|  | NICHD Maternal-Fetal Medicine Units (MFMU) Network | University of Texas Medical Branch at Galveston | Texas |
|  | NICHD Maternal-Fetal Medicine Units (MFMU) Network | Wakemed Raleigh/Wakemed North | North Carolina |
|  | NICHD Maternal-Fetal Medicine Units (MFMU) Network | Yale University | Connecticut |
