# Supplementary material for: Researching COVID to enhance recovery (RECOVER) pediatric study protocol: Rationale, objectives and design
Source: PLoS One. 2024 May 7;19(5):e0285635. doi: 10.1371/journal.pone.0285635 (PMC11075869; doi:10.1371/journal.pone.0285635)
Supplement: S2 Table — (DOCX) [file pone.0285635.s003.docx]

### S2 Table. Inclusion and exclusion criteria

| **Inclusion Criteria** | **Exclusion Criteria** |
| --- | --- |
| - Birth through 25 years old - Any SARS-CoV-2 infection status (never infected, suspected, probable, or confirmed) | - Co-morbid illness with expected survival less than 2 years - Any child or young adult who in the opinion of the site investigator may be at increased risk of adverse events during participation in the study, or who may not be able to complete study procedures due to co-morbid disease or disability - Any young adult above the age of majority who lacks capacity to provide consent - Any child with a plan for adoption or where the state is the legal guardian - Any young adult who is incarcerated, or who lacks capacity to provide consent - Young adult currently enrolled in RECOVER-Adult or RECOVER-Pregnancy |

Note: Participants are eligible without exclusion related to sex, race/ethnicity, geography, nationality, severity of disease, underlying health conditions, the presence or absence of PASC symptoms, or COVID vaccine status.
