# Supplementary material for: Researching COVID to enhance recovery (RECOVER) pediatric study protocol: Rationale, objectives and design
Source: PLoS One. 2024 May 7;19(5):e0285635. doi: 10.1371/journal.pone.0285635 (PMC11075869; doi:10.1371/journal.pone.0285635)
Supplement: S3 Table — (DOCX) [file pone.0285635.s004.docx]

### S3 Table: Inclusion into analytic groups

| **“Infected”: Children and Young Adults with a History of SARS-CoV-2 Infection** |
| --- |
| - Ages newborn through 25 years old - Suspected, probable, or confirmed SARS-CoV-2 infection as defined by WHO criteria since January 1, 2020 - Children/young adults with or without history of MIS-C - Children/young adults with or without history of SARS-CoV-2 vaccination - Children/young adults with evidence of past SARS-CoV-2 infection based on serum antibody profile (with or without history of acute symptoms) - Children/young adults with recurrent SARS-CoV-2 infections and those with post-vaccination (breakthrough) infections |
| **“Uninfected”: Children and Young Adults without a Known History of SARS-CoV-2 Infection** |
| - Does not meet WHO criteria for a suspected, probable, or confirmed case of SARS-CoV-2 infection AND - Does not have serological evidence of past asymptomatic SARS-CoV-2 infection in medical history or Tier 1 testing, AND - Lives in the same communities or recruited from the same sources as those in the SARS-CoV-2 infected cohort, AND - Either not hospitalized for any reason in prior 3 months, or hospitalized (with or without ICU stay) within the prior 3 months   Note: Uninfected individuals who develop SARS-CoV-2 infection during the study period will be reassigned to the SARS-Cov-2 infected group and will be considered to have been enrolled prior to SARS-CoV-2 infection. |
