# Supplementary material for: Researching COVID to enhance recovery (RECOVER) pediatric study protocol: Rationale, objectives and design
Source: PLoS One. 2024 May 7;19(5):e0285635. doi: 10.1371/journal.pone.0285635 (PMC11075869; doi:10.1371/journal.pone.0285635)
Supplement: S4 Table — (DOCX) [file pone.0285635.s005.docx]

**S4 Table: World Health Organization (WHO) criteria**

| **Children and Young Adults with Suspected SARS-Cov-2 Infection** |
| --- |
| 1. Children/young adults who meet these clinical criteria:   At least one of these clinical criteria:   - - Acute onset of fever and cough OR   - Acute onset of any three or more of the following signs or symptoms: fever, cough, general weakness /fatigue, headache, myalgia, sore throat, coryza, dyspnea, anorexia/nausea/vomiting, diarrhea, altered mental status.   AND at least one of these epidemiological criteria:   - - Residing or working in an area with a high risk of transmission of virus: closed residential, school or camp settings anytime within the 14 days before symptom onset; OR   - Residing or travel to an area with community transmission anytime within the 14 days before symptom onset; OR   - Any known household contact or any member of the household working in any health care setting, including within health facilities or within the community; anytime within the 14 days before symptom onset.  1. Patient with history of severe acute respiratory illness (SARI): acute respiratory infection with history of fever or measured fever of ≥ 38 C°; and cough; with onset within the last 10 days; and requires hospitalization 2. An asymptomatic person not meeting epidemiologic criteria with a positive SARS-CoV-2 Antigen-RDT |
| **Children and Young Adults with Probable SARS-Cov-2 Infection** |
| 1. A patient who meets clinical criteria above AND is a contact of a probable or confirmed case or linked to a COVID-19 cluster; OR 2. A suspect case with chest imaging showing findings suggestive of COVID-19 disease; OR 3. A person with recent onset of anosmia (loss of smell) or ageusia (loss of taste) in the absence of any other identified cause |
| **Children and Young Adults with Confirmed SARS-Cov-2 Infection** |
| 1. A person with a positive Nucleic Acid Amplification Test (NAAT); OR 2. A person with a positive SARS-CoV-2Antigen-RDT AND meeting either the probable case definition or suspect criteria A OR B; OR 3. An asymptomatic person with a positive SARS-CoV-2 Antigen-RDT who is a contact of a probable or confirmed case |
| **Children and Young Adults with Asymptomatic SARS-CoV-2 Infection** |
| 1. A person without history of acute COVID-19 symptoms who has one or more of the epidemiological exposures for suspected infection and who also meets criteria b or c for suspected or probable infection, or who meets any of the criteria for confirmed infection 2. A person without history of acute COVID-19 symptoms who has positive nucleocapsid antibody test result in medical history or Tier 1 testing with or without NAAT or RDT testing or known contact to a probable or confirmed case |
