# Supplementary material for: Researching COVID to enhance recovery (RECOVER) pediatric study protocol: Rationale, objectives and design
Source: PLoS One. 2024 May 7;19(5):e0285635. doi: 10.1371/journal.pone.0285635 (PMC11075869; doi:10.1371/journal.pone.0285635)
Supplement: S5 Table — (DOCX) [file pone.0285635.s006.docx]

### S5 Table: Survey topics in tiers 1 and 2 questionnaires

| Survey Instrument | Topic | Asked in Tier 2  follow-up surveys | Source of survey,  if not developed for RECOVER |
| --- | --- | --- | --- |
| Household-Level and Child-Level Surveys | | | |
| Demographics | Name and contact information | ✓ |  |
| Demographics | Alternate contacts | ✓ |  |
| Demographics | Date of birth |  |  |
| Demographics | Sex assigned at birth |  | All of Us Research Program |
| Demographics | Gender identity |  | All of Us Research Program |
| Demographics | Race and ethnicity |  | All of Us Research Program |
| Demographics | Languages spoken |  | California Health Interview Survey |
| Demographics | Country of origin |  | American Community Survey (ACS) |
| Demographics | Educational attainment (grade, school type) |  | National Health and Nutrition Examination Survey (NHANES) |
| Child birth history | Birth mother age |  | National Survey of Children’s Health |
| Child birth history | Child birth weight |  | National Survey of Children’s Health |
| Child birth history | Child birth length |  | National Survey of Children’s Health |
| Child birth history | Prematurity/  gestational age |  | National Survey of Children’s Health |
| Child birth history | Delivery type |  |  |
| Child birth history | NICU admision |  |  |
| Child birth history | Pregnancy complications |  |  |
| Child birth history | Breastfeeding |  |  |
| Child current health status | Child current length  or height | ✓ | National Survey of Children’s Health |
| Child current health status | Child current weight | ✓ | National Survey of Children’s Health |
| Child current health status | Biological parents' height and weight |  |  |
| Child current health status | Child menses | ✓ |  |
| Child current health status | Child disabilities |  | National Survey of Children’s Health |
| Child current health status | Household smoking exposure | ✓ |  |
| Special Health Care Needs Screener | Special Health Care Needs Screener | ✓ | Children with Special Health Care Needs (CSHCN) Screener |
| Special Health Care Needs Screener | Asthma | ✓ | National Survey of Children’s Health |
| Special Health Care Needs Screener | Cerebral Palsy | ✓ | National Survey of Children’s Health |
| Special Health Care Needs Screener | Diabetes | ✓ | National Survey of Children’s Health |
| Special Health Care Needs Screener | Epilepsy or seizure disorder | ✓ | National Survey of Children’s Health |
| Special Health Care Needs Screener | Heart problem | ✓ | National Survey of Children’s Health |
| Special Health Care Needs Screener | Frequent or severe headaches, including migraines | ✓ | National Survey of Children’s Health |
| Special Health Care Needs Screener | Tourette's syndrome or tics | ✓ | National Survey of Children’s Health |
| Special Health Care Needs Screener | Anxiety (feeling nervous or anxious) | ✓ | National Survey of Children’s Health |
| Special Health Care Needs Screener | Depression (feeling very sad) | ✓ | National Survey of Children’s Health |
| Special Health Care Needs Screener | Down syndrome | ✓ | National Survey of Children’s Health |
| Special Health Care Needs Screener | Blood disorders | ✓ | National Survey of Children’s Health |
| Special Health Care Needs Screener | Cystic fibrosis | ✓ | National Survey of Children’s Health |
| Special Health Care Needs Screener | Other genetic or inherited condition | ✓ | National Survey of Children’s Health |
| Special Health Care Needs Screener | Problems with behavior | ✓ | National Survey of Children’s Health |
| Special Health Care Needs Screener | Developmental delay | ✓ | National Survey of Children’s Health |
| Special Health Care Needs Screener | Intellectual disability | ✓ | National Survey of Children’s Health |
| Special Health Care Needs Screener | Speech or other language disorder (problems with talking or understanding words) | ✓ | National Survey of Children’s Health |
| Special Health Care Needs Screener | Learning disability (problem with learning) | ✓ | National Survey of Children’s Health |
| Special Health Care Needs Screener | Autism or Autism Spectrum Disorder (ASD) | ✓ | National Survey of Children’s Health |
| Special Health Care Needs Screener | Attention Deficit Disorder (ADD) or Attention Deficit/Hyperactivity Disorder (ADHD) | ✓ | National Survey of Children’s Health |
| Special Health Care Needs Screener | Eating disorders (like Anorexia or Binge eating disorder) | ✓ | National Survey of Children’s Health |
| Special Health Care Needs Screener | Other health problems | ✓ |  |
| Global Health | Self-reported or caregiver-reported overall, physical and mental health | ✓ | Early Childhood Parent Report Global Health 8a; PROMIS Parent Proxy Scale v1.0 – Global Health 7; PROMIS-10 v1.2 |
| COVID infection history | Infection date | ✓ |  |
| COVID infection history | How family learned about COVID infection | ✓ |  |
| COVID infection history | Presence of symptoms | ✓ |  |
| COVID infection history | Duration of symptoms | ✓ |  |
| COVID infection history | Symptom severity | ✓ |  |
| COVID infection history | Health care utilization during COVID infection | ✓ |  |
| COVID infection history | COVID treatments | ✓ |  |
| Related conditions | Multisystem Inflammatory Syndrome in Children (MIS-C) | ✓ |  |
| Related conditions | POTS (Postural Orthostatic Tachycardia Syndrome) or other form of dysautonomia or autonomic dysfunction | ✓ |  |
| Related conditions | Long COVID diagnosis | ✓ |  |
| COVID Testing History | Testing history |  |  |
| COVID Testing History | Testing access |  |  |
| COVID Family Infection | COVID infection |  |  |
| COVID Family Infection | COVID-related hospitalization |  |  |
| COVID Family Infection | COVID-related death | ✓ |  |
| COVID Symptoms | General symptoms or problems | ✓ |  |
| COVID Symptoms | Symptoms or problems in the eyes, ears, nose, and throat | ✓ |  |
| COVID Symptoms | Symptoms or problems involving the heart and lungs | ✓ |  |
| COVID Symptoms | Symptoms or problems involving the belly | ✓ |  |
| COVID Symptoms | Symptoms or problems involving the skin, hair, and nails | ✓ |  |
| COVID Symptoms | Symptoms or problems involving the bones and muscles | ✓ |  |
| COVID Symptoms | Symptoms or problems involving the brain and nerves | ✓ |  |
| COVID Symptoms | Symptoms or problems involving feelings or behavior | ✓ |  |
| COVID Symptoms | Symptoms or problems involving periods | ✓ |  |
| COMPASS-31 | Symptoms associated with dysautonomia | ✓ | COMPASS-31 |
| COVID vaccine history | Child COVID vaccine history | ✓ |  |
| COVID vaccine history | Birth mother COVID vaccine history while pregnant |  |  |
| COVID vaccine history | Birth mother COVID vaccine history while breastfeeding |  |  |
| COVID vaccine history | COVID vaccine intentions |  |  |
| COVID Health Consequences | Perceived weight status | ✓ | Youth Risk Behavior Survey |
| COVID Health Consequences | Child diet | ✓ | Youth Risk Behavior Survey |
| COVID Health Consequences | Physical activity | ✓ | Youth Risk Behavior Survey |
| COVID Health Consequences | Outdoor play | ✓ |  |
| COVID Health Consequences | Screen time | ✓ | Youth Risk Behavior Survey |
| COVID Health Consequences | Sleep | ✓ | Youth Risk Behavior Survey |
| COVID Health Consequences | School disruption | ✓ |  |
| COVID Health Consequences | Grades | ✓ |  |
| COVID Health Consequences | Developmental services (Early intervention, Individualized Education Programs, home visiting) | ✓ |  |
| COVID Health Consequences | Discipline | ✓ | Quick Parenting Assessment |
| COVID Health Consequences | Caregiver-child relationship quality | ✓ | Adult Child Relationship Scale |
| COVID Health Consequences | Cognitive stimulation (reading, teaching, playing, talking) | ✓ | StimQ cognitive home environment questionnaire (self-report version of infant/toddler; preschool; elementary school age) |
| Social Determinants Of Health | Household composition |  |  |
| Social Determinants Of Health | Birth order |  |  |
| Social Determinants Of Health | Housing |  |  |
| Social Determinants Of Health | Marital status |  |  |
| Social Determinants Of Health | Health care utilization | ✓ | National Survey of Children’s Health |
| Social Determinants Of Health | Health literacy |  | Brief health literacy screener |
| Social Determinants Of Health | Health insurance | ✓ |  |
| Social Determinants Of Health | Unmet needs | ✓ |  |
| Social Determinants Of Health | Health care access | ✓ |  |
| Social Determinants Of Health | COVID-related guidelines (e.g., masking, social distancing) |  |  |
| Social Determinants Of Health | Financial difficulties | ✓ |  |
| Social Determinants Of Health | Financial assistance programs | ✓ |  |
| Social Determinants Of Health | Food insecurity | ✓ | USDA Core Food Security Module |
| Social Determinants Of Health | Perceived neighborhood safety | ✓ |  |
| Social Determinants Of Health | Neighhood cohesion | ✓ |  |
| Social Determinants Of Health | Discrimination | ✓ | Everyday Discrimination Scale |
| Social Determinants Of Health | Early childhood experiences |  | Other Childhood Stressors |
| Social Determinants Of Health | Mental health | ✓ | DSM-5 Cross-Cutting Symptom Measure |
| Caregiver-Level Surveys | | | |
| Identity | Caregiver relationship to child |  |  |
| Demographics | Caregiver date of birth |  |  |
| Demographics | Caregiver sex assigned at birth |  | All of Us Research Program |
| Demographics | Caregiver gender identity |  | All of Us Research Program |
| Demographics | Caregiver race and ethnicity |  | All of Us Research Program |
| Demographics | Caregiver languages spoken |  | California Health Interview Survey |
| Demographics | Caregiver country of origin |  | American Community Survey (ACS) |
| Global Health | Caregiver overall, physical and mental health | ✓ | PROMIS global health scale |
| Current health status | Caregiver disabilities |  |  |
| COVID infection history | Caregiver infection date | ✓ |  |
| COVID infection history | How caregiver learned about their own COVID infection | ✓ |  |
| COVID infection history | Caregiver presence of symptoms | ✓ |  |
| COVID infection history | Caregiver duration of symptoms | ✓ |  |
| COVID infection history | Caregiver symptom severity | ✓ |  |
| COVID infection history | Caregiver health care utilization during COVID infection | ✓ |  |
| COVID infection history | Caregiver COVID treatments | ✓ |  |
| COVID Testing History | Caregiver testing history |  |  |
| COVID vaccine history | Caregiver COVID vaccine history | ✓ |  |
| COVID Symptoms | Caregiver general symptoms or problems | ✓ |  |
| COVID Symptoms | Caregiver symptoms or problems in the eyes, ears, nose, and throat | ✓ |  |
| COVID Symptoms | Caregiver symptoms or problems involving the heart and lungs | ✓ |  |
| COVID Symptoms | Symptoms or problems involving the belly | ✓ |  |
| COVID Symptoms | Caregiver symptoms or problems involving the skin, hair, and nails | ✓ |  |
| COVID Symptoms | Caregiver symptoms or problems involving the bones and muscles | ✓ |  |
| COVID Symptoms | Caregiver symptoms or problems involving the brain and nerves | ✓ |  |
| COVID Symptoms | Caregiver symptoms or problems involving feelings or behavior | ✓ |  |
| COVID Symptoms | Caregiver symptoms or problems involving periods | ✓ |  |
| COVID Health Consequences | Caregiver perceived weight status | ✓ | Behavioral Risk Factor Surveillance System (BRFSS) |
| COVID Health Consequences | Caregiver diet | ✓ | Behavioral Risk Factor Surveillance System (BRFSS) |
| COVID Health Consequences | Caregiver physical activity | ✓ | Behavioral Risk Factor Surveillance System (BRFSS) |
| COVID Health Consequences | Caregiver screen time | ✓ | Behavioral Risk Factor Surveillance System (BRFSS) |
| COVID Health Consequences | Caregiver sleep | ✓ | Behavioral Risk Factor Surveillance System (BRFSS) |
| Social Determinants Of Health | Caregiver work | ✓ |  |
| Social Determinants Of Health | Caregiver health insurance | ✓ |  |
| Social Determinants Of Health | Caregiver health care utilization | ✓ |  |
| Social Determinants Of Health | Caregiver positive childhood experiences (PCEs) |  | Positive Childhood Experiences (PCEs) |
| Social Determinants Of Health | Caregiver discrimination | ✓ | Everyday Discrimination Scale |
| Social Determinants Of Health | Caregiver social support | ✓ | RAND Social Support Survey |
| Caregiver wellbeing | Caregiver depressive symptoms | ✓ | Patient Health Questionnaire-9 |
| Caregiver wellbeing | Caregiver anxiety symptoms | ✓ | Generalized Anxiety Disorder-7 |
| Caregiver wellbeing | Caregiver stress | ✓ | Perceived Stress Scale |
| Caregiver wellbeing | Caregiver mental health | ✓ | DSM-5 Cross-Cutting Symptom Measure |
