# Supplementary material for: Researching COVID to enhance recovery (RECOVER) pediatric study protocol: Rationale, objectives and design
Source: PLoS One. 2024 May 7;19(5):e0285635. doi: 10.1371/journal.pone.0285635 (PMC11075869; doi:10.1371/journal.pone.0285635)
Supplement: S6 Table — (DOCX) [file pone.0285635.s007.docx]

### S6 Table: Clinical and laboratory assessments across the tiers in the *de novo* RECOVER-Pediatrics cohort

| Category | Assessment | Tier 1 | Tier 2 | Tier 3 |
| --- | --- | --- | --- | --- |
| Clinical Assessment | Weight |  | ✓ |  |
| Clinical Assessment | Height or length |  | ✓ |  |
| Clinical Assessment | Waist circumference |  | ✓ |  |
| Clinical Assessment | Skin fold thickness  (triceps and subscapular) |  | ✓ |  |
| Clinical Assessment | Temperature |  | ✓ |  |
| Clinical Assessment | Heart rate |  | ✓ |  |
| Clinical Assessment | Respiratory rate |  | ✓ |  |
| Clinical Assessment | Oxygen saturation |  | ✓ |  |
| Clinical Assessment | Blood pressure |  | ✓ |  |
| Clinical Assessment | 10 Minute Active Standing Test (Assessing blood pressure and heart rate after 5 minutes supine, then after standing for 1, 3, 5, 7 and 10 minutes) |  | ✓ |  |
| Clinical Assessment | Electrocardiogram |  | ✓ |  |
| Clinical Assessment | Spirometry |  | ✓ |  |
| Clinical Assessment | Beighton Scale for Joint Hypermobility |  | ✓ |  |
| Clinical Assessment | NIH toolbox |  | ✓ |  |
| Clinical Assessment | Echocardiogram |  |  | ✓ |
| Clinical Assessment | Cardiac MRI without contrast |  |  | ✓ |
| Clinical Assessment | Pulmonary Function Tests (PFTs) |  |  | ✓ |
| Clinical Assessment | Lung Microbiome (Sputum Induction) |  |  | ✓ |
| Clinical Assessment | Cardiopulmonary Exercise Testing |  |  | ✓ |
| Clinical Assessment | Abdominal ultrasound |  |  | ✓ |
| Clinical Assessment | Brain MRI without contrast |  |  | ✓ |
| Clinical Assessment | Brain EEG |  |  | ✓ |
| Clinical Assessment | Neurocognitive testing |  |  | ✓ |
| Laboratory study | SARS-CoV-2 spike and nucleocapsid antibody | ✓ | ✓ |  |
| Laboratory study | Complete metabolic panel |  | ✓ |  |
| Laboratory study | Complete blood count |  | ✓ |  |
| Laboratory study | Anti nuclear antibody (ANA) |  | ✓ |  |
| Laboratory study | Anti-cyclic citrullinated peptide antibodies (Anti-CCP) |  | ✓ |  |
| Laboratory study | Anti dsDNA antibody |  | ✓ |  |
| Laboratory study | Rheumatoid factor (RF) |  | ✓ |  |
| Laboratory study | Lipid Panel |  | ✓ |  |
| Laboratory study | Hemoglobin A1c |  | ✓ |  |
| Laboratory study | Thyroid stimulating hormone (TSH) |  | ✓ |  |
| Laboratory study | Free T4 |  | ✓ |  |
| Laboratory study | 25-hydroxyvitamin D |  | ✓ |  |
| Laboratory study | Serum calcium |  | ✓ |  |
| Laboratory study | EBV anti early antigen IgG, viral capsid IgM, viral capsid IgG, nuclear antigen IgG |  | ✓ |  |
| Laboratory study | D-Dimer |  |  | ✓ |
| Laboratory study | High sensitivity Troponin |  |  | ✓ |
| Laboratory study | High sensitivity C-reactive protein |  |  | ✓ |
| Laboratory study | Procalcitonin |  |  | ✓ |
| Laboratory study | N-terminal pro-brain natriuretic peptide |  |  | ✓ |
| Laboratory study | Insulin C-peptide |  |  | ✓ |
| Laboratory study | Microbiome specimens: sputum, skin swabs, nasal swabs, oral swabs, urine and stool |  |  | ✓ |
