# Supplementary material for: Researching COVID to enhance recovery (RECOVER) pediatric study protocol: Rationale, objectives and design
Source: PLoS One. 2024 May 7;19(5):e0285635. doi: 10.1371/journal.pone.0285635 (PMC11075869; doi:10.1371/journal.pone.0285635)
Supplement: S7 Table — (DOCX) [file pone.0285635.s008.docx]

| Effect | Assumption | All | | | Age 0-5 (26%) | | | Age 6-11 (28%) | | | Age 12-17 (26%) | | | Age 18-25 (20%) | | | |
| --- | --- | --- | --- | --- | --- | --- | --- | --- | --- | --- | --- | --- | --- | --- | --- | --- | --- |
|  |  | Inf. | Uninf. | Risk diff. | Inf. | Uninf. | Risk diff. | Inf. | Uninf. | Risk  diff. | Inf. | Uninf. | Risk diff. | Inf. | Uninf. | Risk  diff. |  |
| Risk difference in outcome frequency comparing infected and uninfected participants | Risk among uninfected is 10% | 8,300* | 7,700* | 1.9% | 1,248 | 312 | 8.7% | 1,344 | 336 | 8.4% | 4,748* | 6,812* | 2.3% | 960 | 240 | 10.1% |  |
|  |  | PASC+ | PASC- | Odds ratio | PASC+ | PASC- | Odds ratio | PASC+ | PASC- | Odds ratio | PASC+ | PASC- | Odds ratio | PASC+ | PASC- | Odds  ratio |  |
| Odds ratio for PASC comparing those with a risk factor vs. those without | Risk factor has 50% prevalence in PASC- group | 3,600 | 1,800 | 1.25 | 936 | 468 | 1.55 | 1,008 | 504 | 1.53 | 936 | 468 | 1.55 | 720 | 360 | 1.65 |  |
|  | Risk factor has 25% prevalence in PASC- group | 3,600 | 1,800 | 1.28 | 936 | 468 | 1.62 | 1,008 | 504 | 1.59 | 936 | 468 | 1.62 | 720 | 360 | 1.73 |  |
|  |  | PASC+ | PASC- | Odds ratio | PASC+ | PASC- | Odds ratio | PASC+ | PASC- | Odds ratio | PASC+ | PASC- | Odds ratio | PASC+ | PASC- | Odds  ratio |  |
| Odds ratio for PASC comparing those with a risk factor vs. those without | Risk factor has 10% prevalence in PASC- group | 400 | 200 | 2.60 | 104 | 52 | 5.48 | 112 | 56 | 5.21 | 104 | 52 | 5.48 | 80 | 40 | 6.66 |  |

### S7 Table: Power calculations to determine minimum detectable effect sizes, stratified by age group

Inf. = Infected; Uninf. = Uninfected; Risk diff. = Risk Difference
