# Supplementary material for: Researching COVID to enhance recovery (RECOVER) pediatric study protocol: Rationale, objectives and design
Source: PLoS One. 2024 May 7;19(5):e0285635. doi: 10.1371/journal.pone.0285635 (PMC11075869; doi:10.1371/journal.pone.0285635)
Supplement: S1 Protocol — (PDF) [file pone.0285635.s010.pdf]

Study Title: A Multi-Center Observational Study: The RECOVER Post Acute Sequelae of SARS-CoV-2 (PASC) Pediatric Cohort Study

Short plain language title: Understanding the long-term impact of COVID on children and families

RECOVER Clinical Science Core Principal Investigators:	Stuart Katz, MD MS<br>Helen L. and Martin S. Kimmel Professor of Advanced Cardiac Therapeutics<br>NYU Grossman School of Medicine<br>Leon H. Charney Division of Cardiology<br>530 First Avenue, Skirball 9R<br>New York, NY 10016<br>1-833-422-6819<br><br>Rachel Gross MD MS<br>Assistant Professor, Department of Pediatrics at NYU Grossman School of Medicine<br>Assistant Professor, Department of Population Health at NYU Grossman School of Medicine<br>462 First Avenue, 8th floor<br>New York, NY 10016<br>(212) 263-8974<br><br>Andrea B. Troxel, ScD<br>Professor and Director, Division of Biostatistics<br>Department of Population Health<br>NYU Grossman School of Medicine<br>180 Madison Avenue, Suite 5-55<br>New York, NY 10016<br>Tel (646) 501-3654<br><br>Leora Horwitz, MD<br>Associate Professor, Department of Population Health at NYU Grossman School of Medicine<br>Associate Professor, Department of Medicine at NYU Grossman School of Medicine<br>Director, Center for Healthcare Innovation and Delivery Science<br>Director, Division of Healthcare Delivery Science<br>550 1st Avenue, Suite 1803<br>New York, NY 10016
RECOVER pediatric and prenatal/infant cohort site Principal Investigators:	1. Jessica Snowden, Arkansas Children's Research Institute<br>2. David Warburton Children's Hospital Los Angeles/University of Southern California<br>3. Melissa Stockwell, Columbia University Irving Medical Center<br>4. Julie Miller, New England Research Institutes<br>5. Patricia Kinser and Amy Salisbury, Virginia Commonwealth University<br>6. Lawrence Kleinman, Robert Wood Johnson Medical School<br>7. Terry Jernigan, University of California at San Diego<br>8. Kelan Tantisira, University of California at San Diego<br>9. Valerie Flaherman, University of California at San Francisco<br>10. Torri Metz, University of Utah Health
NYULH Study Number:	s21-0123
Clinicaltrials.gov ID Number	NCT05172011
Funding Sponsor:	Other Transactional Authority OT2HL161847-01<br>National Heart, Lung, And Blood Institute (NHLBI)

	National Institutes Of Health (NIH)<br>9000 Rockville Pike Bethesda, MD 20892

Initial version: V1.0 17 SEP 2021
Amended: V2.0 10 DEC 2021, V2.1 24 DEC 2021, V2.2 31 JAN 2022, V2.3 01 MAR 2022, V2.4 03 OCT 2022, V3.0 10 NOV 2022, V3.1 20 JAN 2023, V3.2 28 MAR 2023, V3.3 23 AUG 2023

Table of Contents

1 SUMMARY OF PROTOCOL REVISIONS 9

2 PROTOCOL SUMMARY 21

3 KEY ROLES 23

4 OVERVIEW OF STUDY PROTOCOL ENROLLMENT FOR RECOVER META-COHORT 24

5 INTRODUCTION, BACKGROUND INFORMATION AND SCIENTIFIC RATIONALE 24

5.1 BACKGROUND INFORMATION AND RELEVANT LITERATURE 24
5.2 RATIONALE 26

6 POTENTIAL RISKS & BENEFITS 26

6.1.1 KNOWN POTENTIAL RISKS 26
6.1.2 KNOWN POTENTIAL BENEFITS 31

7 OBJECTIVES AND PURPOSE 31

7.1 SPECIFIC AIMS 31

8 STUDY DESIGN AND ENDPOINTS 32

8.1 OVERALL STUDY DESIGN 32
8.2 RECRUITMENT PROCEDURES 33
8.3 COHORT AND CASE-NONCASE STUDY RECRUITMENT POOLS 33
8.3.1 OVERVIEW OF COHORT TYPES CONTRIBUTING TO THE RECOVER META-COHORT ..... ERROR! BOOKMARK NOT DEFINED.
8.3.2 CATEGORIES OF RECRUITMENT POOLS IN THE RECOVER META-COHORT 35

9 STUDY ENROLLMENT AND WITHDRAWAL 37

9.1 Inclusion Criteria 37
9.1.1 Infected Cohort: 37
9.1.2 Children/Young Adults with Suspected SARS-Cov-2 Infection 38
9.1.3 Children/Young Adults with Probable SARS-Cov-2 Infection 38
9.1.4 Children/Young Adults with Confirmed SARS-Cov-2 Infection 38
9.1.5 Children/Young Adults with Asymptomatic SARS-CoV-2 Infection 39
9.1.6 Non-Infected Cohort 39
9.1.7 *Children (≤3 years of age) born in and out of the context of maternal SARS-CoV-2 infection during pregnancy.* 39
9.1.8 Children with MIS-C 39
9.1.9 Children/Young Adults with Post-Vaccine Myocarditis .... **Error! Bookmark not defined.**
9.1.10 Primary Caregiver Entry Criteria 39
9.1.11 Biological Parent Entry Criteria 40
9.2 Exclusion Criteria 40
9.3 Vulnerable Subjects 40
9.4 Strategies for Recruitment and Retention 41
9.4.1 Study Recruitment Strategy and Sampling 41
9.4.2 Retention Strategy 42
9.5 Duration of Study Participation 43
9.6 Total Number of Subjects and Sites 43
9.7 Participant Withdrawal or Termination 43
9.7.1 Reasons for Withdrawal or Termination 43
9.7.2 Handling of Participant Withdrawals or Termination 44
9.7.3 Premature Termination or Suspension of Study 44

10 STUDY SCHEDULE 44

10.1 Overview of study schedule 44
10.2 Enrollment/Baseline 46
10.3 Intermediate Visits (Tiers 2 and 3) 46
10.4 Final Study Visit 47
10.5 Withdrawal Visit 47
10.6 Unscheduled Visit 47

11 STUDY PROCEDURES/EVALUATIONS 47

11.1 Tiered phenotyping by sub-cohorts 47
11.2 Main PASC Pediatric Cohort Module (n=6000 infected and uninfected subjects, ages newborn-25 years) 48
11.3 Main PASC Pediatric Cohort Module Tier 1 Procedures 48
11.3.1 Main Cohort Tier 1 procedures ages newborn-5 years (not including congenital exposure) ...48
11.3.2 Main Cohort Tier 1 procedures ages 6-17 years 49
11.3.3 Main Cohort Tier 1 procedures ages 18-25 years 50
11.4 Main pediatric cohort acute Tier 2 assessments 50
11.4.1 Main Cohort Acute Tier 2 procedures ages newborn-5 years 51
11.4.2 Main Cohort Acute Tier 2 procedures ages 6-17 years 52
11.4.3 Main Cohort Acute Tier 2 procedures ages 18-25 years 52
11.5 Main Cohort Post-Acute Tier 2 Assessments 53
11.5.1 Main Cohort Post-Acute Tier 2 assessments ages newborn-5 years 53

11.5.2 Main Cohort Post-Acute Tier 2 remote assessments ages 6-17 years 54
11.5.3 Main Cohort Post-Acute Tier 2 remote assessments ages 18-25 years 56
11.6 Main Pediatric Cohort Tier 3 assessments 57
11.6.1 Main cohort Tier 3 assessments (ages 3-5 years) 57
11.6.2 Main cohort Tier 3 assessments (ages 6-25 years) 58
11.7 MIS-C Cohort (n=800, ages 3-25 years) 60
11.7.1 MIS-C cohort Tier 1 remote assessments 60
11.7.2 MIS-C cohort Tier 2 visits 60
11.7.3 MIS-C cohort Tier 3 visits 61
11.8 Post-vaccine Myocarditis Cohort (n=200, ages 3-25 years) .... Error! Bookmark not defined.
11.8.1 Post-vaccine myocarditis remote Tier 1 assessments .... **Error! Bookmark not defined.**
11.8.2 Post-vaccine myocarditis remote post-acute Tier 2 visits .... **Error! Bookmark not defined.**
11.9 Infants born to mothers with and without history of SARS-CoV-2 during pregnancy (n=2500, ages newborn-48 months) 61
11.9.1 Infants cohort Tier 1 assessments (remote) 62
11.9.2 Infants cohort Tier 2 assessments (on-site or remote) 62
11.10 ABCD cohort (n=10,000, ages 12-17 years) 63
11.10.1 ABCD Tier 1 assessments 63
11.11 Caregiver Assessments 64
11.11.1 Caregiver Tier 1 Assessments 64
11.11.2 Caregiver Tier 2 Assessments (remote time of Child/Young Adult visits at 12, 24, 36, and 48 months) 64
11.12 Other Biological Parent Assessments 64
11.13 Mobile Health Products and Devices Data Collection 65
11.14 Laboratory Procedures/Evaluations 65
11.14.1 Biospecimen collection overview 65
11.14.2 Tier 1 biospecimen collections 66
11.14.3 Tier 2 Acute biospecimen collections 66
11.14.4 Tier 2 Post-acute biospecimen collections 66
11.14.5 Tier 3 biospecimen collections 68
11.15 Study Specific Biospecimens 69
11.15.1 Specimen Collection Procedures 69
11.15.2 Specimen Preparation, Handling, and Storage 69
11.15.3 Specimen Shipment 69
11.16 Questionnaire Administration 70

12 SAFETY AND ADVERSE EVENTS 70

12.1 Definitions 70
12.2 Recording of Adverse Events 71
12.3 Reporting of Serious Adverse Events and Unanticipated Problems 71
12.3.1 Investigator reporting: notifying the IRB 71

13 STUDY OVERSIGHT 73

13.1 Monitoring Board 73
13.2 Data Safety Monitoring Plan 74

14 STATISTICAL CONSIDERATIONS 74

14.1 SAMPLE SIZE DETERMINATIONS **74**
14.2 STATISTICAL METHODS **75**
14.2.1 METHODS OF DATA COLLECTION 75
14.2.2 STRATEGIES FOR STUDY MODIFICATIONS 76
14.2.3 OVERVIEW OF ANALYTIC APPROACH TO AIMS 76
14.3 DATA MANAGEMENT PLAN **78**
14.3.1 DATA CATEGORIES 78
14.3.2 DATA TYPES 79
14.3.3 FUNCTIONAL DATA WORKFLOW SUMMARY 79
14.3.4 HARVARD MEDICAL SCHOOL (HMS) AWS CLOUD ENVIRONMENT 79
14.3.5 DATA STORAGE 79
14.3.6 DATA DESTRUCTION 79
14.3.7 DATA INTEGRITY 80
14.3.8 SECURITY MANAGEMENT 80
14.3.9 SOURCE DOCUMENTS AND ACCESS TO SOURCE DATA/DOCUMENTS 80

15 ETHICS/PROTECTION OF HUMAN SUBJECTS **80**

15.1 ETHICAL STANDARD **80**
15.2 INSTITUTIONAL REVIEW BOARD **80**
15.3 INFORMED CONSENT PROCESS **81**
15.3.1 CONSENT/ASSENT AND OTHER INFORMATIONAL DOCUMENTS PROVIDED TO PARTICIPANTS 82
15.3.2 CONSENT PROCEDURES AND DOCUMENTATION ..... **ERROR! BOOKMARK NOT DEFINED.**
15.3.3 POSTING OF CLINICAL TRIAL CONSENT FORM 83
15.4 PARTICIPANT AND DATA CONFIDENTIALITY **83**
15.4.1 REDCAP DATABASE SECURITY 84
15.4.2 DATA STORAGE SECURITY 84
15.4.3 STORAGE OF STUDY MATERIALS 84
15.4.4 HASHED IDENTIFIERS 84
15.4.5 DATAVANT TOKENIZATION AND PRIVACY PROTECTING RECORD LINKAGE (PPRL) TECHNOLOGY 84
15.4.6 LIMITED DATA SET 85
15.4.7 REPORTING OF INCIDENTAL FINDINGS 85
15.4.8 REPORTING OF GENETIC TESTING 85
15.4.9 CERTIFICATE OF CONFIDENTIALITY 86
15.4.10 RESEARCH USE OF STORED HUMAN SAMPLES, SPECIMENS, OR DATA 87
15.5 FUTURE USE OF STORED SPECIMENS **87**

16 DATA HANDLING AND RECORD KEEPING **88**

16.1 DATA COLLECTION AND MANAGEMENT RESPONSIBILITIES **88**
16.2 STUDY RECORDS RETENTION **88**
16.3 PROTOCOL DEVIATIONS **88**
16.4 PUBLICATION AND DATA SHARING POLICY **89**

17 STUDY FINANCES **89**

17.1 FUNDING SOURCE **89**
17.2 COSTS TO THE PARTICIPANT **89**

17.3 PARTICIPANT REIMBURSEMENTS OR PAYMENTS 89

18 STUDY ADMINISTRATION 89

18.1 STUDY LEADERSHIP 89

19 CONFLICT OF INTEREST POLICY 89

20 APPENDICES 91

20.1 APPENDIX A: ENROLLMENT ENTRY TIMEPOINTS 91
20.2 APPENDIX B. OVERVIEW SCHEDULE OF ASSESSMENTS BY RECRUITMENT POOL 92
20.3 PROPOSED SAMPLE SIZE BY TIER 99
20.4 APPENDIX D: TIER 1: DATA ELEMENTS 102
20.5 APPENDIX E: TIER 2 AND 3: CORE DATA ELEMENTS 109

21 REFERENCES 113

22 APPENDIX E. LIST OF STUDY SITES .... ERROR! BOOKMARK NOT DEFINED.

Statement of Compliance

This study will be conducted in accordance with the Code of Federal Regulations on the Protection of Human Subjects (45 CFR Part 46), any other applicable US government research regulations, and institutional research policies and procedures. The Principal Investigator will assure that no deviation from, or changes to the protocol will take place without prior agreement from the sponsor and documented approval from the Institutional Review Board (IRB), except where necessary to eliminate an immediate hazard(s) to the study participants. All personnel involved in the conduct of this study have completed Human Subjects Protection Training.

List of Abbreviations

ABCD study	Adolescent Brain Cognitive Development study
AE	Adverse Event/Adverse Experience
ATO	Authority to Operate
CBCL	Child Behavior Checklist
CDC	Centers for Disease Control
CFR	Code of Federal Regulations
CI	Confidence Interval
CLIA	Clinical Laboratory Improvement Amendments
COVID-19	Coronavirus Disease 2019
CRF	Case Report Form
DRC	Data Resource Core
EDTA	Ethylenediaminetetraacetic acid
EHR	Electronic Health Record
FISMA	Federal Information Security Modernization Act of 2002
HIPAA	Health Insurance Portability and Accountability Act of 1996
ICF	Informed Consent Form
IRB	Institutional Review Board
IRR	Incident Rate Ratio
MIS-C	Multisystem Inflammatory Syndrome In Children
MOP	Manual of Procedures
MRI	Magnetic Resonance Imaging
N	Number (typically refers to participants)
NHLBI	National Heart, Lung, and Blood Institute
NIH	National Institutes of Health
NYULH	New York University Langone Health
OSMB	Observational Study Monitoring Board
PASC	Post-Acute Sequelae of SARS-CoV-2
PBMC	Peripheral Blood Mononuclear Cells
PHI	Private Health Information
PI	Principal Investigator
PII	Personal Identifiable Information
Post-vax MC	Post-COVID vaccine myocarditis
RECOVER	Researching COVID to Enhance Recovery
REDCap	Research Electronic Data Capture
SST	Serum Separator Tube
US	United States
UUID	Universal Unique identifier
WGS	Whole Genome Sequencing
WHO	World Health Organization

1 Summary of Protocol Revisions

August 23, 2023: Version 3.2 to 3.3

Location and description of revision	Rationale for revision
Entire document	Post-vaccine myocarditis removed from RECOVER cohort following approval of all relevant governance bodies
Cover page	Updated PI information for Virginia Commonwealth University hub
6.1 Known Potential Risks - Tier 1 Procedures Risk	Updated age of saliva collection (2 years and older, optional 5 years and under) for internal document consistency and alignment with current approved ICFs
6.1 Known Potential Risks - Tier 2 Procedures Risk	Updated age of pulmonary function/spirometry collection (ages 5 and older) for internal document consistency and alignment with current approved ICFs
6.1 Known Potential Risks - Tier 3 Procedures Risk	Updated age of brain MRI (ages 6 and older) for internal document consistency and alignment with current approved ICFs
8.3.2 Categories of recruitment pools in the RECOVER meta-cohort, Recruitment targets by demographics, symptoms, and calendar time of infection.	Asymptomatic target lowered from 50% to 15-30% consistent with RECOVER Study Design committee and OSMB review and approval
9.7.2 Handling of Participant Withdrawals or Termination	Updated language concerning the End of Participation form used to record participant withdrawal or other study discontinuance
11.5.1 Main Cohort Post-Acute Tier 2 assessments ages Newborn-5 years	Removed Brief Infant Sleep Questionnaire (BISQ) from main cohort assessments, as it's only administered to congenitally exposed cohort
11.13.1 Biospecimen Collection Overview	Widened window for sites to review clinically obtained labs for recording in CRF from 30 days to 3 months
14.1 Sample size determinations	Asymptomatic target lowered from 50% to 15-30% consistent with RECOVER Study Design committee and OSMB review and approval
20.2 Appendix B Overview Schedule of Assessments by Recruitment Pool	Typo in Week 8 visit window updated from +7 months post-enrollment to +7 weeks post-enrollment
20.3 Appendix C. Proposed Sample Size by Tier	For Table 14: Promotion likelihood for participants with Medium probability of PASC increased from 50% to 100% following approval from RECOVER committees and PIs
20.6 Appendix F: Clinical and Laboratory Assessments	Clarified which Tier 2 Month 6 visit laboratory collections are processed locally versus centrally

March 28, 2023: Version 3.1 to 3.2

Location and description of revision	Rationale for revision
Document generally	The protocol as a whole has been updated for Section 508 accessibility compliance.

Location and description of revision	Rationale for revision
Section 6.1 Known Potential Risks	Consolidated descriptions of assessment risk. Collapsed age groupings to avoid contradiction with age-specific assessment schedules in Section 11.
Section 6.1.1 Tier 2 ages Newborn-5 years	Added language clarifying that head circumference is for participants <2 years old.
Section 6.1.1 Tier 2 ages 6-17 (child) and ages 18-25 (young adult)	Removed head circumference from assessments, as it does not apply to these ages.
9.1.10 Primary Caregiver Entry Criteria	Clarifying language was added for consenting and assenting procedures for caregivers below the legal age of majority.
9.2 Exclusion Criteria	Modified terminology about plan for adoption.
9.7.2: Handling of Participant Withdrawals or Termination	In the Handling of Participant Withdrawals or Termination section, details were added about the number of follow-up attempts and methods of outreach needed prior to classifying a participant as lost to follow-up.
10.1: Overview of study schedule	Expanded the window for the first post-acute follow-up assessments to begin earlier between 2 to 6 months. Added more details on how the clinical laboratory tests will be collected at each site and processed locally or sent to a central laboratory for testing.<br>Several references to “age-appropriate assent” edited to align with the submitted unified youth 7-17 assent.
10.2: Enrollment/Baseline	Clarifying language about the study design was added, related to the established algorithm for who will be promoted to tier 2. Changes also aimed to clarify procedures for the post-acute arm and the acute arm of the study.
Section 10.3: Intermediate Visits (Tiers 2 and 3)	Updated maximum number of visits for acute cohort participants from 10 visits to 9.
11.3 Main Cohort Module Tier 1 Procedures	Paper-based surveys are available for use in cases of limited access to electronic data capture systems.
11.3.1 Main Cohort Tier 1 procedures ages newborn-5 years (not including congenital exposure cohort)	Removed acute arm language in this section since Tier 1 is not associated with acute arm. Added clarifying language for cases of acute COVID after enrollment that their remote biospecimen collection can be delayed for 30 days following onset of infection.
11.3.2 Main Cohort Tier 1 procedures 6-17 years	Removed acute arm language in this section since Tier 1 is not associated with acute arm. Added clarifying language for cases of acute COVID after enrollment that their remote biospecimen collection can be delayed for 30 days following onset of infection.
11.3.3 Main Cohort Tier 1 procedures Tier 18-25 years	Removed acute arm language in this section since Tier 1 is not associated with acute arm. Added clarifying language for cases of acute COVID after enrollment that their remote biospecimen collection can be delayed for 30 days following onset of infection.

Location and description of revision	Rationale for revision
Section 11.4.1: Main Cohort Acute Tier 2 Procedures Ages Newborn to Five Years	Participants less than 2 years of age will have study visits conducted remotely and participants aged 2 to 5 years will have their week 8 visit conducted in person.<br>Added clarifying language on how their clinical data and biospecimen samples will be collected.<br>Added language clarifying that head circumference is for participants <2 years old.<br>Added clarifying language about the child electrocardiogram and child spirometry being conducted in person at the 8-week visit.<br>Remote biospecimen collection was removed from on site assessment batteries.
Section 11.4.2: Main Cohort Acute Tier 2 Procedures Ages 6-17 Years	Added clarifying language about the child electrocardiogram being conducted in person at the 8-week visit.<br>Removed head circumference from assessments, as it does not apply to these ages.<br>Remote biospecimen collection was removed from on site assessment batteries.
11.4.3 Main Cohort Acute Tier 2 procedures ages 18-25 years	Added clarifying language about the electrocardiogram being conducted in person at the 8-week visit.<br>Remote biospecimen collection was removed from on site assessment batteries.
Section 11.5: Main Cohort Post-Acute Tier 2 Assessments	Widened study window from two months to -2/+6 months for Tier 2 post-acute visits.<br>Further defined that some assessments are optional after first *successful* post-acute collection rather than simply any first post-acute visit.<br>Added language that optional after first post-acute collection within normal limits; if abnormal, assessment may be repeated at subsequent visits per site investigator's discretion.
Section 11.5.1: Main Cohort Post-Acute Tier 2 Assessments Ages Newborn to 5	Added neurocognitive assessments for on-site visits.<br>Added language clarifying that head circumference is for participants <2 years old.<br>Adjusted study window for first post-acute Tier 2 visit to align with Core Operations Group recommendation (between 2 to 6 months).<br>Removal of Bayley Scale of Infant Development 4 (child development) at age 24 months.
Section 11.5.2: Main Cohort Post-Acute Tier 2 Remote Assessments Ages 6-17	Added clarifying language that NIH Toolbox test, auditory verbal learning test (Rey) verbal memory, is for ages 8 years and up.<br>Added clarifying language that child spirometry is optional for participants less than 7 years per judgment of site investigator.<br>Added new survey for remote assessment to record data for the household social determinants of health,

Location and description of revision	Rationale for revision
11.6 Main Pediatric Cohort Post-Acute Tier 3 assessments	Added language on tier 3 visits to be conducted twice, first visit at 12-30 months and the second visit at 24-42 months after study entry.
11.6.1 Main cohort Tier 3 assessments (ages 3-5 years)	Removal of child brain MRI assessment.
Section 11.7.2: MIS-C Cohort Tier 2 visits	Updated age for the autonomic dysfunction assessment from ages 5 to 25 years to 6 years to 25 years.<br>Removed outdated language referring to biospecimen collection in this cohort.<br>Removal of Young Adult anthropometry and vital signs (including oximetry) for on site assessments.
11.7.3 MIS-C cohort Tier 3 visits	Added language on how tier 3 procedures conducted as part of COVID MUSIC study may be used in the RECOVER study as a Tier 3 measure.
11.9 Infants born to mothers with and without history of SARS-CoV-2 during pregnancy (n=2500, ages newborn-48 months)	Added language stating that the congenital cohort performs limited portions of assessments within RECOVER pediatric protocol which are listed in section 11.9.1, 11.9.2 and Appendix B table 13 which are all considered minimal risk.<br>Added language clarifying that Tier 1 assessments for this cohort are done remotely and Tier 2 refers to in-person or video-conferencing assessments.<br>Added Figures 3 and 4, illustrating cohort enrollment breakdown and assessment battery, respectively
11.14 Tier 2 Acute biospecimen collection	Added language for biospecimen collection kit contents to align with PASC Biorepository Core guidance.
11.14.4 Tier 2 Post-acute biospecimen collections	Added language for biospecimen collection kits for the congenitally exposed cohort 24-months.
Section 11.9.1 Infant cohort Tier 1 assessments (remote)	Added language clarifying that head circumference is for participants <2 years old.
Section 11.9.2: Infants Cohort Tier 2 Assessments (on site or remote)	Added growth assessment at 24 months for on-site visit and added language clarifying that head circumference is for participants <2 years old.<br>Added clarifying language on how blood collection will be conducted with at-home collection device (Tasso M-20).
Section 11.14.3: Tier 2 Acute Biospecimen Collections	Updated age from 2 to 6 years.
14 Statistical Consideration	Updated scientific aims of the study related to characteristics prevalence/incidence, characterization of PASC symptoms, risk/resiliency factors for PASC and defining pathophysiology of PASC.
14.1 Sample size determination	Clarified sample size discussion for Pediatric Main Cohort for the acute infected, uninfected and post-acute infected for Tier 1 and 2.<br>Clarified discussion about the sample size for participants enrolling into the study from ABCD and MIS-C cohort.

Location and description of revision	Rationale for revision
15.3 Informed Consent Process	Revised youth assent to reflect the requested move to a single document for obtaining informed assent from participants 7-17 years of age.
20.2: Appendix B. Overview Schedule of Assessments by Recruitment Pool	Widened post-acute Tier 2 visit windows to -2/+6 months<br>Harmonized assessments in schedule with CRFs in project REDCap.Table 8: Schedule of Assessments: Main Pediatric Cohort Tiers1-3 Ages 6-25 years has been updated and aligned with the changes mentioned in previous sections. Language was added for scheduling the 8 week in-person visit.<br>Table 9 : Schedule of Assessments: Main Cohort Tiers 1-3 Ages Newborn-5 years has been updated and aligned with the changes mentioned in previous sections. Language was added for scheduling the 8 week in-person visit.<br>Tables 10, 11, 12 and 13 have updated legends.
20.3 Appendix C.: Proposed Sample Size by Tier	For Table 14: Promotion likelihood language was added to clarify that all participants assigned to acute arm are automatically promoted to Tier 2.
20.4 Appendix D: Tiers 1 and 2 Data Elements	Added Table 15 to describe surveys and assessments administered in Tiers 1 and 2, including their sources, for the RECOVER Main Cohort
20.5 Appendix E: Tiers 2 and 3 Core Data Elements	Added Table 16 to describe Neurocognitive, Neurobehavioral, Well-Being and Mental Health Measures by Age in Tiers 2 and 3 for the Main Cohort

January 20, 2023: Version 3.0 to 3.1

Location and description of revision	Rationale for revision
8.2 Recruitment procedures	Added language corresponding to submission of Waiver of Authorization covering participant recruitment at all RECOVER sites

November 10, 2022: Version 2.4 to 3.0

Location and description of revision	Rationale for revision
Cover page: Principal Investigator, Clinical Science Core	Updated to include appointment of Dr. Rachel Gross as Study PI for the CSC.
Cover page: RECOVER site Principal Investigators	Updated to reflect Rhode Island Hospital PI Viren D'Sa
2 Protocol Summary: Number of participants	Updated participant enrollment numbers from up to 20,000 to up to 19,300 dyads. Also changed date from March 1, 2020 to January 1, 2020 in the definition of infection for individuals ages newborn-25 years meeting WHO criteria for suspected, probable or confirmed SARS-CoV-2 infection on or after January 1, 2020.
3 Key Roles: Principal Investigator, Clinical Science Core	Updated to include appointment of Dr. Rachel Gross as Study PI for the CSC.

Location and description of revision	Rationale for revision
4 Overview of Study Protocol Enrollment for RECOVER Meta-cohort	Updated participant enrollment numbers from up to 20,000 to up to 19,500 dyads.
6.1.1. Known Potential Risks: Tier 2 ages 6-17 years (child) and ages 18-25 years (young adult): Ziopatch section	Removed Ziopatch from protocol due to feasibility concerns.
8.3.2 Categories of recruitment pools in the RECOVER meta-cohort: Matched acute and post-acute uninfected participants in the main cohort.	Expanded language to clarify that there is no limitation on the number of siblings who can be enrolled across the study, and no limitation on the number of children who can enroll from within a single household.
9.1.1-ii Inclusion Criteria, Infected Cohort	Updated the inclusion criteria for the infected cohort from individuals who have suspected, probable, or confirmed SARS-CoV-2 infection within 24 months of enrollment to allow enrollment of individuals who have suspected, probable, or confirmed SARS-CoV-2 infection since January 1, 2020.
9.2 Exclusion Criteria	Added clarifying language to expand on the restriction of participants ages 18-25 years old at the time of enrollment from enrolling into both the pediatric cohort as a young adult participant and the adult cohort as a participant.
9.4.1 Study Recruitment Strategy and Sampling	Added new table to define the special populations.
9.6 Total Number of Participants and Sites	New Section Title – Formerly "Total Number of Subjects and Sites"<br><br>Updated number of enrolled participants from up to 20,000 dyads to up to 19,500 dyads.
11.3.1 Main Cohort Tier 1 procedures ages newborn-5 years (not including congenital exposure)	Added more detail and created more alignment with REDcap surveys about the domains assessed under *child medical history* [including birth weight and length, gestational age, delivery type, problems during pregnancy), current health history (including current self-reported weight and height, disabilities, smoke exposure), special health care needs, and history of MIS-C, POTS and Long COVID], *COVID health consequences* [including child weight, diet, physical activity, screen time, sleep, educational services, parenting (e.g., discipline, parent-child relationship quality, cognitive stimulation [StimQ])], and *household and child social determinants of health* (housing, financial, and food insecurity, health literacy, other childhood stressors, neighborhood stress); Additionally, clarified the window for Tier 1 assessment completion (3 months), including biospecimen collection.

Location and description of revision	Rationale for revision
11.3.2 Main Cohort Tier 1 procedures ages 6-17 years	Added more detail and created more alignment with REDcap surveys about the domains assessed under *child medical history* [including birth weight and length, gestational age, delivery type, problems during pregnancy), current health history (including current self-reported weight and height, disabilities, smoke exposure), special health care needs, and history of MIS-C, POTS and Long COVID], *COVID health consequences* [including child weight, diet, physical activity, screen time, sleep, educational services, parenting (e.g., discipline, parent-child relationship quality, cognitive stimulation [StimQ])], and *household and child social determinants of health* (housing, financial, and food insecurity, health literacy, other childhood stressors, neighborhood stress, DSM-5 cross-cutting measure)
11.3.3 Main Cohort Tier 1 procedures ages 18-25 years	Added more detail and created more alignment with REDcap surveys about the domains assessed under *child medical history* [including birth weight and length, gestational age, delivery type, problems during pregnancy), current health history (including current self-reported weight and height, disabilities, smoke exposure), special health care needs, and history of MIS-C, POTS and Long COVID], *COVID health consequences* [including child weight, diet, physical activity, screen time, sleep, educational services], and *household and child social determinants of health* (housing, financial, and food insecurity, health literacy, other childhood stressors, neighborhood stress, DSM-5 cross-cutting measure)
11.5.1 Main Cohort Post-Acute Tier 2 assessments ages newborn-5 years	Deleted Differential Ability Scales-II and Developmental Profile-4 for the main cohort, this will remain for the congenital exposure cohort only. Main cohort children 3 years and older will use the NIH toolbox instead. Clarified that growth will be assessed in person; Clarified that Brief infant sleep questionnaire is administered remotely via redcap.
11.5.2. Main Cohort Post-Acute Tier 2 Remote Assessments ages 6-17 years: Remote or on-site Assessments	Removed PROMIS life satisfaction, PROMIS Meaning and Purpose, PROMIS Social Isolation, PROMIS Family Relationships scales and PROMIS Sleep-Related Disturbances. Clarified the COMPASS-31 is administered remotely via redcap. Added child emotional and mental health measures (PROMIS positive affect (Short form), PROMIS Emotional Distress – Anger (Short form), PROMIS Psychological Stress Experiences (Short form), Revised Children's Anxiety and Depression Scale-25 (RCADS-25) – parent reported; and Strengths and Difficulties Questionnaire (conduct and hyperactivity scales only).

Location and description of revision	Rationale for revision
11.5.2 Main Cohort Post-Acute Tier 2 Remote Assessments ages 6-17 years On-site assessments	Added Active standing test; Removed Ziopatch from On-site Assessments. Updated child neurocognitive assessments from PROMIS scales to NIH Toolbox assessments: NIH toolbox: Picture Vocabulary Test (in person): Language Development – Receptive; NIH toolbox: Flanker Inhibitory Control and Attention Test (in person): Executive Functions - Inhibition and Attention; NIH toolbox: List Sort Working Memory Test (in person): Verbal Working Memory; NIH toolbox: Dimensional Change Card Sort Test (in person): Executive Functions - Flexibility and Switching of Cognitive Tasks; NIH toolbox: Pattern Comparison Processing Speed Test (in person): Processing Speed; NIH toolbox: Picture Sequence Memory Test (in person): Visual Memory; NIH toolbox: Oral Reading Recognition Test: Reading Ability; NIH toolbox: Auditory Verbal Learning Test (Rey) (in person): Verbal Memory
11.5.3 Main Cohort Post-Acute Tier 2 remote assessments ages 18-25 years: Remote or on-site Assessments	Removed PROMIS life satisfaction, PROMIS Meaning and Purpose, PROMIS Social Isolation, PROMIS Family Relationships scales and PROMIS Sleep-Related Disturbances. Clarified the COMPASS-31 is administered remotely via redcap. Added child emotional and mental health measures (PROMIS positive affect (Short form), PROMIS Emotional Distress – Anger (Short form), PROMIS Psychological Stress Experiences (Short form), and ASEBA-PC Adult Self-Report.
11.5.3 Main Cohort Post-Acute Tier 2 remote assessments ages 18-25 years: On-site Assessments	Added Active standing test; Removed Ziopatch from On-site Assessments. Updated child neurocognitive assessments from PROMIS scales to NIH Toolbox assessments: NIH toolbox: Picture Vocabulary Test (in person): Language Development – Receptive; NIH toolbox: Flanker Inhibitory Control and Attention Test (in person): Executive Functions - Inhibition and Attention; NIH toolbox: List Sort Working Memory Test (in person): Verbal Working Memory; NIH toolbox: Dimensional Change Card Sort Test (in person): Executive Functions - Flexibility and Switching of Cognitive Tasks; NIH toolbox: Pattern Comparison Processing Speed Test (in person): Processing Speed; NIH toolbox: Picture Sequence Memory Test (in person): Visual Memory; NIH toolbox: Oral Reading Recognition Test: Reading Ability; NIH toolbox: Auditory Verbal Learning Test (Rey) (in person): Verbal Memory
11.5.4 Monitoring Plan for Mental Health Responses	Added a new section to indicate that there is a detailed SOP that details the process for responding to mental health alerts.
11.7.2 MIS-C cohort Tier 2 visits	Harmonized MUSIC tier 2 measures with the main cohort changes listed above.
11.9 Infants born to mothers with and without history of SARS-CoV-2 during pregnancy (n=2500, ages newborn-48 months)	Added new section to clarify the timeline and delivery methods for the assessments for the infant cohort.

Location and description of revision	Rationale for revision
11.9.1 Infant cohort Tier 1 assessments (remote)	Added more detail and created more alignment with REDcap surveys about the domains assessed under National Survey of Children's Health Questionnaire: sociodemographics, child birth history, health status, special health care needs, PROMIS global health, COVID and vaccine history, health consequences [including child weight, diet, physical activity, screen time, sleep, educational services, parenting (e.g., discipline, parent-child relationship quality, cognitive stimulation [StimQ]), social determinants of health. Clarified that these assessments which align with the main cohort assessments will occur at 12, 18, 24, 36, and 48 months old.
11.10.1 ABCD Tier 1 assessments	Harmonized ABCD tier 1 measures with the main cohort changes listed above.
11.11.1 Caregiver Tier 1 Assessments	Added more detail and created more alignment with REDcap surveys about the domains assessed under caregiver COVID health consequences, caregiver social determinants of health, and caregiver wellbeing forms. Caregiver COVID health consequences form includes weight, diet, physical activity, screen time, sleep. Caregiver social determinants of health form includes employment history, health insurance, health care utilization, positive childhood experiences, perceived discrimination, social support (RAND-MOS). Caregiver wellbeing form includes depressive symptoms (patient health questionnaire-9); anxiety symptoms (generalized anxiety questionnaire-7); stress (perceived stress scale); DSM-5 cross-cutting measure (assessment of anger, mania, psychosis, repetitive thoughts and behaviors, dissociation, personality functioning).
11.14.1 Biospecimen Collection Overview:	Added language stating that inadequate Tasso M-20 collections will be handled per the Manual of Procedures.
11.14.1 Biospecimen Collection Overview	Added language to indicate only one Tasso-M20 redraw is recommended per participant.
11.14.1 Biospecimen Collection Overview	Included language to outline that acute and post-acute participants without a blood draw will be eligible for Tier 2 promotion as determined by the promotion algorithm. Uninfected participants without a blood draw will not be eligible for Tier 2 promotion.
11.14.2 Tier 1 Biospecimen Collections	Added language stating that in participants with new, acute infections (within the last 30 days), sites will delay the collection of blood and saliva samples until 30 days after the onset of this new infection.
12 Safety and Adverse Events	Added language to bring protocol in line with new guidance on Unanticipated Problems, Adverse Events, and Reportable New Information
12.3 Reporting of Serious Adverse Events and Unanticipated Problems	Removed known manifestations of SARS-CoV-2 infection from reporting requirements given that these symptoms and conditions will be captured throughout the RECOVER protocol.

Location and description of revision	Rationale for revision
14 Statistical Considerations	Updated participant enrollment numbers from up to 20,000 to up to 19,500 dyads.
20.2 Appendix B. Overview Schedule of Assessments by Recruitment Pool	Added language to state that while the preferred target for acute Tier 2 visits at 8 weeks is 7 to 9 weeks post-study entry, these visits can be scheduled up to 12 weeks from study entry.<br><br>Extended post-acute Tier 2 visit window from 2 months to 3 months.
20.2 Appendix B. Overview Schedule of Assessments by Recruitment Pool	Added language to indicate that acute visits at Weeks 2 and 4 that fall greater than 36 days post-infection will not be conducted.
20.3 Proposed Sample Size by Tier	Updated enrolled participant numbers from 20,000 to 19,500; Figure 4 removed, as its contents were no longer current<br><br>Added description of the tier 2 promotion algorithm, which will use three PROMIS questions as well as the number of PASC symptoms (major and minor).
20.4 Appendix D: Tier 1: Data Elements	Added more detail and created more alignment with REDcap surveys
20.5 Appendix E. Tier 2 and 3: Core Data Elements - Tier 2: Neurological/child developmental assessments: Ages 3 to 18 – NIH Toolbox-PROMIS Measures	Updated the acute assessment schedule to weeks 2, 4, and 8 weeks. Updated neurocognitive assessments from PROMIS measures to reflect proper assessment battery (NIH Toolbox)

October 20, 2022: Version 2.3 to 2.4

Location and description of revision	Rationale for revision
Section 6.1.1: Defined age 24 to <72 month (2-5 years) blood collection to reflect volume-by-weight calculation of 2 mL/kg for maximum of <5% total blood volume	Provide validated, study-wide rationale for blood collection in very young RECOVER participants
Section 10.1.1: Defined age 24 to <72 month (2-5 years) blood collection to reflect volume-by-weight calculation of 2 mL/kg for maximum of <5% total blood volume	Provide validated, study-wide rationale for blood collection in very young RECOVER participants
Section 11.5.1: Defined age 24 to <72 month (2-5 years) blood collection to reflect volume-by-weight calculation of 2 mL/kg for maximum of <5% total blood volume	Provide validated, study-wide rationale for blood collection in very young RECOVER participants
Section 11.9.2: Defined age 24 to <72 month (2-5 years) blood collection to reflect volume-by-weight calculation of 2 mL/kg for maximum of <5% total blood volume	Provide validated, study-wide rationale for blood collection in very young RECOVER participants
Section 11.14.1: Updated table to define blood collection for participants 24 to <72 months as derived using volume-by-weight calculation of 2 mL/kg for maximum of <5% total blood volume; Added link to blood volume-by-weight calculator	Provide validated, study-wide rationale for blood collection in very young RECOVER participants; Link to blood volume calculator will assist RECOVER personnel in determining
Section 11.14.3: Updated contents of Tier 2 Acute blood collection kits and corresponding assays	Clarify kit contents for use in Tier 2
Section 11.14.4: Updated contents of Tier 2 Post-Acute blood collection kits and corresponding assays	Clarify kit contents for use in Tier 2

March 1, 2022: Version 2.2 to 2.3

Location and description of revision	Rationale for revision
Section 8.2: Added description of IRB-approved eligibility screener for recruitment	To standardize screening procedures at enrolling sites
Section 8.3: Added description of randomization of uninfected control participants for Tier 2 longitudinal follow-up	To maintain harmonization with the updated statistical analysis plan
Section 10.3: Changed description of intermediate visits after newly identified infection	To clarify study procedures and harmonize with the updated acute Tier 2 visit schedule described in revised section 11.4
Section 11.4 and Appendix B: Reduced number of acute Tier 2 remote visits	To reduce participant burden and increase feasibility of the acute Tier 2 procedures
Section 11: Added text to indicate that EHR and mobile health platform may be used to collect health status and symptom data during longitudinal follow up in acute and post-acute Tier 2	Clarification
Section 12.3: Changed the event reporting requirements	To align with NYU IRB reporting requirements
Remove Appendix F	To eliminate redundancy with other sections of the protocol

January 31, 2022: Version 2.1 to 2.2

Location and description of revision	Rationale for revision
Section 9 clarifications	Clarified entry criteria for primary caregiver, other biological parent, and children/young adults with history of MIS-C.
Section 10 new study visit procedure	Added description of intermediate visit procedures in case of a newly discovered SARS-CoV-2 infection or re-infection after study entry.
Section 11 minor changes in study procedures and biospecimen collection	1.Simplified post-acute Tier 2 visit schedule for pediatric main cohort to harmonize with pediatric statistical analysis plan revision. Visits will be timed from date of study entry instead of date of COVID infection.<br>2. Added description of Tasso M-20 blood collection device for home Tier 1 blood spot collection.<br>3. Added Tier 2 and Tier 3 optional blood collection for MIS-C cohort
Sections 9 and 15 clarification of study recruitment procedures	Clarification of use of supporting materials to enhance understanding of the study as part of the informed consent process.

December 24, 2021: Version 2.0 to 2.1

Location and description of revision	Rationale for revision
Title Page	Addition of clinicaltrials.gov registration number; updating contact information
Section 6 and 15	Clarifications of procedures and risks for return of new information from incidental findings and genetic testing

Location and description of revision	Rationale for revision
Section 11	Clarifications of timing of study visits for post-acute Tier 2 main cohort, MIS-C and post-vaccine myocarditis sub-cohorts. Reformatting and minor edits to the Tier 3 panel of neurocognitive testing.
Appendix F	Updated to maintain consistency with the revised protocol sections

December 10, 2021: Version 1.0 to 2.0:

Location and description of revision	Rationale for revision
Section 1 added	To provide summary of protocol revisions
Section 4 Figure 1 Overview of Study Protocol Enrollment changed	More detailed overview of the study protocol enrollment to be harmonized with the pediatric statistical analysis plan
Section 5 Background updated	Added relevant citations published since last version
Section 6 Risks updated	Clarify risk mitigation by age of participant
Section 8 Study Design updated and re-organized	Clarify description of cohort types, recruitment strategies and recruitment pools
Section 9 Study enrollment updated	Clarify study entry criteria
Section 10 Study Schedule updated	Clarify scheduling of study visits
Section 11 Study Procedures re-organized	Clarify study procedures by age of participant and sub-cohort
Section 11 Study Procedures revised	To reduce participant burden
Section 14 Statistical considerations updated; Data management plan updated	Clarify plan for subgroup analysis by age group; data management plan harmonized with RECOVER DRC manual of operations
Section 15 Ethics/Protection of human subjects updated; Data security section updated	Clarify informed consent procedures; added additional information on data security procedures to harmonize with RECOVER DRC manual of operations
Appendix figures revised	Updated schedule of assessment figures and tables

2 Protocol Summary

Title	A Multi-Center Observational Study: The RECOVER Post-Acute Sequelae of SARS-CoV-2 (PASC) Pediatric Cohort Study
Short Title	Understanding the long-term impact of COVID on children and families
Brief Summary	This is a combined retrospective and prospective, longitudinal, observational meta-cohort of individuals ages newborn-25 years who will enter the cohort with and without SARS-CoV-2 infection at varying stages before and after infection. Individuals with and without SARS-CoV-2 infection and with or without PASC symptoms will be followed to identify risk factors and occurrence of PASC. This study will recruit participants from inpatient, outpatient, and community-based settings in the United States. Study data including age, demographics, social determinants of health, medical history, vaccination history, details of acute SARS-CoV-2 infection, overall health and physical function, and PASC symptoms will be reported by participants or collected from the electronic health record using a case report form at specified intervals. Biologic specimens will be collected at specified intervals, with some tests performed in local clinical laboratories and others performed by centralized research centers or banked in the Biospecimen Repository. Advanced clinical and radiologic examinations will be performed at local study sites with cross-site standardization.
Objectives	1. Characterize the incidence and prevalence of sequelae of SARS-CoV-2 infection occurring >30 days after study entry.<br>2. Characterize the spectrum of clinical symptoms, subclinical organ dysfunction, natural history, and distinct phenotypes identified as sequelae of SARS-CoV-2 infection occurring >30 days after study entry.<br>3. Define the biological mechanisms underlying pathogenesis of the sequelae of SARS-CoV-2 infection occurring >30 days after study entry.
Methodology	Ambidirectional longitudinal meta-cohort study (combined retrospective and prospective) with nested case-control studies.
Endpoints	Primary Endpoint: Presence of candidate PASC symptoms over time.<br>Secondary Endpoints: Clinical and biological recovery trajectories from SARS-CoV-2 infection; clinical and subclinical organ injury; incident post-SARS-CoV-2 clinical disease.
Study Duration	Four years
Participant Duration	Up to four years after study entry

Population	Infected: Individuals ages newborn-25 years meeting WHO criteria for suspected, probable or confirmed SARS-CoV-2 infection on or after January 1, 2020, including participants with history of MIS-C, and infants born to a mother meeting the same infected criteria during pregnancy.<br><br>Uninfected: Individuals ages newborn-25 years who have never met any of the WHO criteria for suspected, probable or confirmed SARS-CoV-2 infection, including infants born to a mother meeting the same uninfected criteria during pregnancy.<br><br>The primary caregiver of the child (or children) enrolled in the RECOVER pediatric cohort may provide consent to participate in prospective data and biological sample collection as specified in the protocol procedures as a member of a caregiver/child dyad.<br><br>If the identified primary caregiver is a biological parent of the child, the other biological parent may provide consent to participate in prospective biospecimen sample collection.
Study Sites	Phase 2 PASC Consortium Pediatric Cohort Sites (Please see Appendix E)
Number of participants	Up to 19,300 dyads (children and young adults with and without history of SARS-CoV-2 infection and their primary caregiver), including 800 children with MIS-C. Up to 19,300 caregivers and 19,300 other biological parents are anticipated to enroll.
Statistical Analysis	A flexible study design is proposed to allow modifications to PASC case definition, tiered phenotyping assessments, comparator groups, and/or statistical plan to optimize public health and scientific impact of study findings. Modifications in study design may be based on analyses of structured cohort data, unstructured cohort EHR data, other cohort EHR data, or other new knowledge of PASC acquired after study initiation.

3 Key Roles

Principal Investigators<br>Clinical Science Core	Rachel S. Gross, MD, MS, FAAP<br>Assistant Professor of Pediatrics and Population Health<br>Department of Pediatrics<br>New York University Grossman School of Medicine<br>Bellevue Hospital Center<br>462 First Avenue, 8th Floor<br>New York, NY 10016<br>(212) 263-8974
	Stuart Katz, MD MS<br>Helen L. and Martin S. Kimmel Professor of Advanced Cardiac Therapeutics<br>NYU Grossman School of Medicine<br>530 First Avenue, Skirball 9R<br>New York, NY 10016<br>(833) 422-6819
	Andrea B. Troxel, ScD<br>Professor and Director, Division of Biostatistics<br>Department of Population Health<br>NYU Grossman School of Medicine<br>180 Madison Avenue, Suite 5-55<br>New York, NY 10016<br>(833) 422-6819
	Leora Horwitz, MD<br>Associate Professor, Department of Population Health at NYU Grossman School of Medicine<br>Director, Division of Healthcare Delivery Science<br>550 1st Avenue, Suite 1803<br>New York, NY 10016
Contact Principal Investigator<br>Data Resource Core	Andrea S Foulkes, ScD<br>Director, Biostatistics, Massachusetts General Hospital<br>Professor of Medicine, Harvard Medical School<br>Professor, Department of Biostatistics, Harvard TH Chan School of Public Health<br>50 Staniford Street, Suite 560 | Boston, MA 02114 | +1 (617) 724-8208

4 Overview of Study Protocol Enrollment for RECOVER Meta-cohort

Figure 1: Overview of study protocol enrollment for RECOVER meta-cohort.

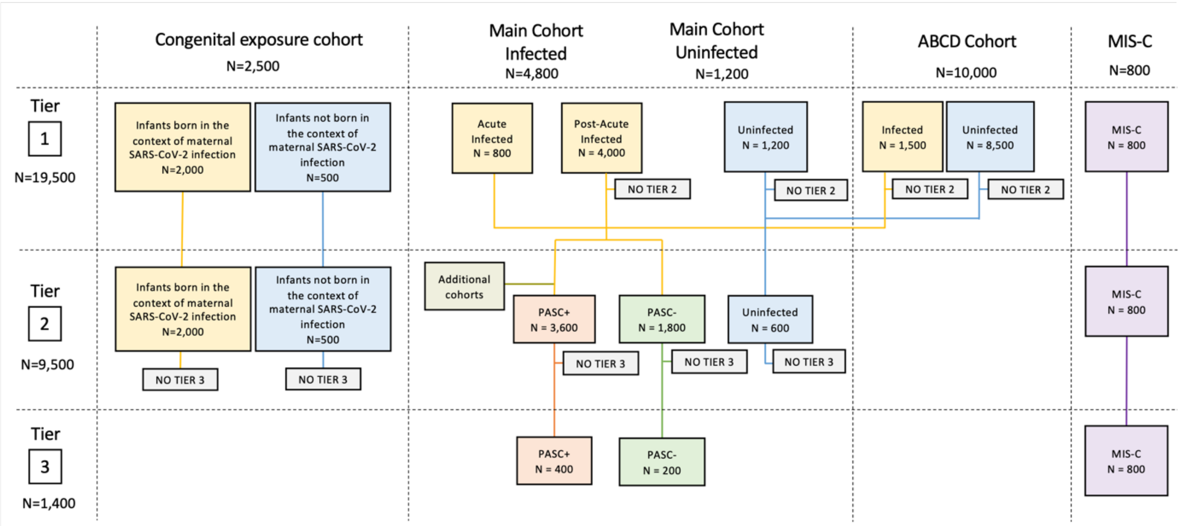

Figure 1 Key Points:

1. Up to 19,300 children and young adults ages newborn through25 years will be enrolled as a meta-cohort, including 2500 infants born to mothers with and without SARS-CoV-2 infection during pregnancy (congenitally exposed infant cohort), 6,000 children and young adults age newborn through25 years from the main RECOVER cohort with and without SARS-CoV-2 infection, up to 10,000 adolescents from the Adolescent Brain Cognitive Development (ABCD) study, and 800 children and young adults ages 3-25 years with Multisystem Inflammatory Syndrome in Children (MIS-C).
2. Each enrolled subject will undergo a tailored tiered testing approach based on history of SARS-CoV-2 infection, presence of PASC symptoms, age range, and other considerations specific to each sub-cohort of the RECOVER meta-cohort. Subjects will be selected for participation in Tier 2 based on their history of COVID exposure and PASC symptoms as determined in Tier 1 for post-acute participants and uninfected controls, and at the week 8 visit for acute Tier 2 participants. Subjects will be selected for participation in Tier 2 and Tier 3 to achieve the enrollment targets as summarized in Figure 1 and to achieve pre-specified targets for age distribution and diversity.

5 Introduction, Background Information and Scientific Rationale

5.1 Background Information and Relevant Literature

The severe acute respiratory syndrome coronavirus 2 (SARS-CoV-2) is a novel coronavirus strain that was first detected at the end of 2019. This strain primarily spreads through aerosols in expiratory gases in infected individuals with and without symptoms, resulting in the highly contagious coronavirus disease 2019 (COVID-19) and a global pandemic. As of June 2021, approximately 177 million people were infected with COVID-19, with at least 3.8 million deaths globally (1). COVID-19 positive cases are identified with a SARS-CoV-2 polymerase chain reaction test or an antigen test using saliva, nasopharyngeal or bronchial samples (2). Fever, chills, cough, shortness of breath, fatigue, muscle aches, loss of taste and/or smell, nausea, diarrhea, and other symptoms are typical of the acute phase of the disease (3,4). The spectrum of symptoms in acute SARS-CoV-2 infection in children is similar to adults, but typically less severe, and with minimal or no reported symptoms in almost half of all cases (5). Chest pain or burning, labored breathing, disorientation, and delirium are less common emergent symptoms in both adults and children associated with increased risk

of hospitalization and death. Increased age, (6) male sex, obesity, and co-morbidities such as diabetes, cardiac disease, or cancer (3,7) are associated with increased risk of severe COVID-19 disease and death in adults. Additionally, non-White people have experienced much higher rates of infection, severe disease, and death when compared with non-Hispanic Whites (8). Case fatality rates in children with acute COVID-19 are lower than adults. In hospitalized children with COVID-19, age < 2 years, indigenous ethnicity, and presence of ≥2 pre-existing conditions were associated with increased risk of death (9).

Multi-organ injury and dysfunction in acute COVID-19 have been reported in both adults and children (7,9-11). One distinct manifestation of PASC in children became recognized in April 2020, with the first reports from Europe of what is now called Multisystem Inflammatory Syndrome in Children, or MIS-C (13,14). MIS-C was initially defined as a multisystem hyperinflammatory syndrome in children and adolescents temporarily related to COVID-19, with features overlapping with Kawasaki disease and toxic shock syndrome. The case definition was revised by CDC as disease of children <21 years of age characterized by fever, laboratory evidence of inflammation, and clinically severe illness with multisystem organ involvement requiring hospitalization within 4 weeks of a laboratory confirmed SARS-CoV-2 infection (15). MIS-C is considered a subset of PASC because it occurs 4-6 weeks after the acute SARS-CoV-2 infection. More than 5,000 children and adolescents in the US have been diagnosed with MIS-C. Acute COVID-19 in children may also manifest as severe disease with multi-organ involvement but with clinical and laboratory patterns that are distinct from those observed in children with MIS-C (16).

After recovery from acute COVID-19, 30-70% of adults infected report a diverse array of persistent mild to severe symptoms lasting >4 weeks. The persistent symptoms after acute have been termed Long COVID or post COVID-19 condition. The term post-acute sequelae of SARS-CoV-2 (PASC) has been proposed to encompass a broader array of post-COVID-19 disease including evidence of organ dysfunction or emergence of new diseases that may occur independently of symptoms. There is no established definition for any of these terms; the WHO has proposed a working clinical case definition of post COVID-19 condition by a Delphi consensus based on a global consensus of a diverse stakeholders.(12) The WHO working case definition is based on an established history of COVID-19, and presence of symptoms in 12 identified domains that persist over time without alternative explanation.

The underlying pathophysiology of persistent symptoms after SARS-CoV-2 infection is unknown but has been proposed to be attributable to viral persistence, reactivation of other viruses such as Epstein-Barr virus, vascular endothelial damage, small fiber autonomic nerve damage, neuroinflammation in the central nervous system, immune dysregulation including activation of auto-immunity, and organ damage caused by hyper-inflammatory response during the acute phase of the disease (8). Myocarditis has been reported after SARS-CoV-2 infection in young adults and rarely in children after administration of mRNA-based COVID-19 vaccines (13,14); this observation suggests that immunological response to the SARS-CoV-2 spike protein may be contributing to cardiac manifestations of PASC in children with and without evidence of MIS-C. Other contributing causes include complications from critical illness related to prolonged intubation, malnutrition, and prolonged bed rest, and effects of pandemic-related stressors, including disruption of school, disruption of family dynamics by COVID-19 in multiple family members, household financial stress, and disruptions of health care access.

Commonly reported persistent symptoms after SARS-CoV-2 infection include fatigue, post-exertional malaise, dyspnea, cough, chest pain, palpitations, cognitive dysfunction and neuropsychiatric symptoms, gastrointestinal symptoms, extreme thirst, hair loss, and persistent loss of taste or smell (8,15-19). Persistent respiratory symptoms may occur independently of demonstrable abnormalities in lung structure or function (8,15,19). The severity of the acute COVID-19 manifestation has consistently been found to be directly proportional to the severity of post-COVID manifestation, but severe post-acute symptoms have been reported in subjects with mild or asymptomatic acute COVID-19 (15).

The clinical manifestations of non-MIS-C PASC in children are less well characterized when compared with adults with wide-ranging estimates of incidence across available studies. The WHO working definition may not be applicable in children. A study from Italy examined health data in 129 children under the age of 18 obtained via a questionnaire between September 2020 and 1 January 2021. 53% of the group experienced COVID-19 symptoms more than 120 days after their diagnosis. Symptoms included chest tightness and pain, nasal congestion, tiredness, difficulty concentrating and muscle pain (20). Among 55 children hospitalized for

COVID-19 in Sweden, 22% were found to have persistent symptoms four months after hospitalization, with fatigue being the most common symptoms (21). An analysis of private health claims data indicated that pain and difficulty breathing were the two most commonly documented medical problems after acute SARS-CoV-2 infection in children ≤18 years of age (33). In 518 hospitalized children age ≤18 years in Russia, 24% reported persistent symptoms after median 256 days after hospital discharge (22). Fatigue, sleep disturbance and sensory disturbance were the most commonly reported symptoms. Increased age and history of allergic disease were associated with increased risk of persistent symptoms. In a community-based sample of school aged children from the United Kingdom, 4.4% of the children with symptomatic laboratory-confirmed SARS-CoV-2 infection had symptoms lasting ≥28 days. The most frequently reported persistent symptoms were fatigue, headache and anosmia (23). A seroprevalence study of Swiss school children reported overall prevalence of prolonged symptoms <5%, and no difference between children with and without antibodies to SARS-CoV-2 (24). An analysis of electronic health records in 11,950 children/adolescents in Germany, demonstrated increased incidence rate ratio of overall health problems after laboratory COVID-19 diagnosis when compared with uninfected controls (IRR=1.30, 95%-CI=[1.25-1.35]). Malaise/fatigue/exhaustion was the symptom complex with the highest IRR versus controls (2.28, 95%-CI=[[1.71-3.06]) (25).

The goal of this study is to identify, evaluate, and characterize the clinical course of PASC symptoms and gain insight into underlying mechanisms in children, adolescents and young adults ages newborn to 25 years with previous SARS-CoV-2 infections with and without a history of PASC, and in infants born in the context of maternal SARS-CoV-2 infection, and the risk and resiliency factors associated with the severity of the clinical course of PASC. This ambidirectional (combined retrospective and prospective) longitudinal observational cohort study will focus on the long-term effects of SARS-CoV-2 infection, while explicitly considering sex and racial and ethnic disparities in risks and outcomes. Data acquired from this study will provide accurate and quantifiable measures for PASC symptoms in selected case and control populations to allow for comparisons among groups and provide insights into mechanisms related to PASC progression and recovery. Additionally, it is hoped that such findings will identify predictive and prognostic factors for PASC that will inform study design and enrich participant selection for future clinical trials of the prevention and/or treatment of PASC.

5.2 Rationale

PASC stands to pose a profound public health crisis in future years, with anticipated increased risk of morbidity, mortality, and disability in both adults and children. As of August 2021, there were over 37 million diagnosed cases of COVID-19 in the United States; as of the end of October 2021, nearly 25% of new cases were in the pediatric age group (26). The true US prevalence of SARS-CoV-2 infection is likely much greater when accounting for asymptomatic disease, limited access to testing and underreporting, particularly in children (27,28). Given the large number of cases of COVID-19 in the United States, even a low incidence of PASC might affect millions with immense impact on health care resource utilization, and public health measures of morbidity, mortality and disability. The long-term public health impact of PASC may be greater in the pediatric population when compared with adults despite less severe acute disease in children. The incidence, prevalence, phenotypes, risks, and etiology of PASC in US children are currently unknown, limiting opportunities for prevention and treatment (27). Therefore, prospective longitudinal studies in the pediatric population are urgently needed. The proposed study will enroll child or young adult/caregiver dyads to characterize indirects of effects of the SARS-CoV-2 pandemic on study endpoints, and if possible DNA samples from the child or young adult and both biological parents for future studies of genetic risks of long-term effects of SARS-CoV-2 infection. This pediatric study will enhance knowledge of children's recovery from SARS-CoV-2 infections and define and categorize the clinical spectrum and risk factors for PASC and elucidate potential mechanisms to inform future preventive and treatment studies.

6 Potential Risks & Benefits

6.1 Known Potential Risks

All study procedures are considered minimal risk, with exception of Tier 3 exercise testing and sputum induction, which are considered to be a minor increase over minimal risk. All study procedures will be performed when clinically appropriate based on age and investigator judgment. Participation in the study is

associated with a small risk of breach of confidentiality. Study procedures to reduce risk of breach are described below.

Tier 1 Procedures Risk

- Questionnaires are tailored to the caregiver as the primary respondent with optional input from the child. Questionnaires may include questions that make people feel uncomfortable, anxious, angry, or sad. Questionnaires can also cause fatigue. To minimize risk, study personnel will explain that all questions are optional, and that a question can be skipped if the participant prefers not to answer. The platform for the questionnaires will allow participants to take a break and come back to where they left off. All questionnaires are approved by the IRB prior to use.
    - For participants aged 18-25 years, questionnaires are tailored to the young adult as the primary respondent with optional input from the caregiver if available.
    - For caregivers, caregiver questionnaires are tailored to the caregiver adult as the primary respondent. If the primary caregiver is below the age of majority, the caregiver's parent or legal guardian will be the primary respondent, with input from the minor caregiver.
- Saliva collection is an optional biospecimen collection for children ages 2-5 years. Saliva collection has no known risks. Collection might be messy. Written and oral instructions are provided for saliva collection to children, young adults, and caregivers where applicable.
- Blood collection (ages 2 years and older). Blood collection at home is associated with risk of pain, bruising, lightheadedness and fainting. Specialized FDA-approved lancet devices designed to create a small drop of blood from the shoulder region, or other areas of the body (e.g., back or thigh) with reduced density of pain receptors will be used for blood collection for Tier 1 post-acute participants.

Tier 2 Procedures Risks

Procedures in Tier 2 will only be performed when clinically appropriate based on age and investigator judgment.

- Questionnaires are tailored to the caregiver as the primary respondent, with optional input from the child. Questionnaires may include questions that make persons feel uncomfortable, anxious, angry, or sad. Questionnaires can also cause fatigue. To minimize risk, study personnel will explain that all questions are optional, and that a question can be skipped if the participant prefers not to answer. The platform for the questionnaires will allow participants to take a break and come back to where they left off. All questionnaires will be approved by the IRB prior to use.
    - For participants aged 18-25 years, questionnaires are tailored to the young adult as the primary respondent with optional input from the caregiver.
    - For caregivers, questionnaires are tailored to the caregiver adult as the primary respondent. If the primary caregiver is below the age of majority, the caregiver's parent or legal guardian will be the primary respondent, with input from the minor caregiver.
- Anthropometry and vital signs including pulse oximetry: Child blood pressure; heart rate; oxygen saturation; oral, axillary or skin temperature; height (length); weight; head circumference (<2 years); waist circumference; and skin fold thickness will be measured. These procedures have no known risk, but could make some participants feel uncomfortable. All examination and vital sign procedures will be performed in a private examination room, in the company of the caregiver, with trained research personnel and chaperones if needed or requested.
- Home pulse oximetry. There are no known risks associated with use of an FDA-approved pulse oximeter sized to age of the child. The use of an oximeter is optional for children <7 years of age per judgment of the site investigator and caregiver.
- The electrocardiogram procedure (ages 3 years and older) is optional for children <5 years old per judgment of the site investigator and caregiver. A 12-lead or 15-lead electrocardiogram will be performed based on site practice with FDA-approved equipment. Self-sticking pediatric size electrodes will be placed on the extremities and chest for the electrocardiogram recording and will be removed after the recording. The electrocardiogram will be completed in approximately 5 minutes. Electrocardiography is considered a minimal risk procedure. The adhesive on the gel electrodes may cause transient minor skin irritation.

- Phlebotomy for blood collection (ages 2 years and older) is considered a minimal risk procedure. For participants newborn to <72 months (ages 0-5 years), maximal single phlebotomy will be derived using a blood volume-by-weight calculation of 2 mL/kg for <5% total blood volume. Maximal single phlebotomy volume for ages 72 months to <10 years (6-years to under 10 years) 25 mL; maximal single phlebotomy volume for ages 10-25 years is 38 mL. Blood collection may be associated with minor temporary discomfort. There is a risk of bruising and a very small amount of bleeding associated with the blood collection. There is also a very small risk of infection at the site of blood collection. Standard of care phlebotomy procedures will be used to mitigate these risks.
- Child and young adult spirometry testing (ages 5 years and older): The spirometry procedure is optional in children <7 years of age per judgment of the site investigator and caregiver. An FDA-approved hand-held spirometry system will be used to measure lung function. Subjects will be asked to blow into a single-use tube to capture exhaled gases. Three exhalations will be assessed. Spirometry is considered a minimal risk procedure. Blowing into a tube may cause transient fatigue or lightheadedness.

Tier 3 Procedures Risk

Procedures in Tier 3 will only be performed when clinically appropriate based on age and investigator judgment.

- Echocardiogram (age 3-25 years). An echocardiogram will be performed according to standard clinical protocols and is optional for age <7 years per judgement of the site investigator and caregiver. This is considered a minimal risk procedure. There is no known risk to cardiac ultrasound imaging. Gel electrodes for electrocardiogram monitoring during the test may cause skin irritation. The hand-held probe may cause a sensation of pressure, and rarely pain on the chest wall. Echocardiograms will be performed by trained technicians with pediatric experience.
- Cardiac MRI (ages 5-25 years). A cardiac MRI will be performed according to standard clinical protocols in children who do not require sedation and is optional for age <9 years per judgment of site investigator and caregiver. This is considered a minimal risk procedure. Participants may experience anxiety or claustrophobia in the MRI scanner. These symptoms will be mitigated by the use of entertainment/distraction whenever possible. Children with metallic implants will not be permitted to participate. Children who require sedation for the MRI will not be allowed to participate. No intravenous contrast agent will be administered for research purposes. To minimize risk, the MRI will be conducted by trained technicians under supervision of pediatric cardiologists or licensed radiologists at each study site.
- Pulmonary function tests including diffusing capacity (ages 5-25 years). Pulmonary function testing will be completed according to clinical protocols and is optional for age <9 years per judgment of the site investigator and caregiver. Pulmonary function tests are considered minimal risk procedures, but can be associated with transient fatigue and lightheadedness.
- Child sputum induction (ages 10-25 years). A standard of care clinical protocol for sputum induction will be used and is optional for children <12 years per judgment of the site investigator and caregiver. Sputum induction is considered a minor increase over minimal risk procedure due to small risk of bronchospasm, and small risks of use of levalbuterol to reduce the risk of bronchospasm. Participants will inhale nebulized hypertonic saline solution (3%) for 5-15 minutes to liquify airway secretions, promote coughing and allow expectoration of respiratory secretions. Coughing may be an uncomfortable feeling. If a participant is uncomfortable from coughing, the mist will be stopped. The mist may also induce a transient sore throat. Hypertonic saline can induce bronchospasm, with estimated risk of symptomatic bronchospasm in non-asthmatic children of 0.1% (29,30). To minimize risk of bronchospasm, children with asthma will be excluded and all children will receive pre-treatment with a bronchodilator (levalbuterol). Levalbuterol is an analog of endogenous catecholamines with established safety profile in studies of >600 children (31). Oxygen saturation will be monitored throughout the procedure. Handheld spirometry will be used to monitor subjects at 5-minute intervals during the inhalation procedure. The inhalation will be stopped if the $FEV_{1.0}$ decreases by more than 20% from pre-testing baseline or if the participant has symptoms of wheezing or shortness of breath, or if oxygen saturation decreases to <94%. The laboratory will be supervised by a licensed pediatric pulmonologist and emergency bronchodilator medications will be available in the room used for sputum induction.

- Symptom-limited cardiopulmonary exercise testing (ages 10-25 years). Participants will perform symptom-limited graded exercise on a treadmill or stationary bicycle ergometer according to clinical protocols in children and is optional for children <12 years per judgment of local site investigator and caregiver. Cardiopulmonary exercise testing is considered to be a minor increase over minimal risk procedure. Appropriately sized gel electrodes will be placed to measure the electrocardiogram before, during and after exercise. Exercise testing mimics exertion experienced during normal activities of living and is associated with a very small risk of arrhythmia or low blood pressure during or after exercise (32). A trained exercise technician in an experienced center that performs pediatric cardiopulmonary exercise testing will be present throughout the exercise test to monitor the participant; cardiac monitoring of the rhythm and serial blood pressure measurements will be performed throughout the course of the exercise test and during recovery. Participants with history of exercise intolerance will be carefully monitored and testing procedures may be modified if symptoms emerge. Children and young adults with known heart disease, including history of pericarditis, myocarditis, or MIS-C with cardiac involvement within the prior 6 months, abnormal resting electrocardiogram, or evidence of active myocardial inflammation on cardiac MRI findings will not undergo exercise testing.
- Abdominal ultrasound (ages 3-25 years). An abdominal ultrasound examination will be performed by a trained technician according to clinical protocols and is optional for age <7 years per judgment of the site PI and caregiver). Abdominal ultrasound is considered a minimal risk procedure. The liver, pancreas, kidneys, and bladder will be imaged. There is no known risk to abdominal ultrasound. Local pressure of the handheld probe may cause discomfort or pain.
- Brain MRI (ages 6-25 years): A 3T or lower field strength brain MRI will be performed according to standard clinical protocols in children who do not require sedation and is optional for age <9 years per judgment of the site investigator and caregiver. Brain MRI is considered a minimal risk procedure. Children with metallic implants will not be permitted to participate. Children who require sedation for the MRI will not be allowed to participate. No intravenous contrast agent will be administered. To minimize risk, the MRI will be conducted by trained technicians under supervision of licensed pediatricians and/or radiologists at each study site.
- Awake EEG (ages 3-25 years): An electroenchephalogram (EEG) will be performed according to clinical protocols with FDA approved equipment and is optional for age <9 years per judgement of the site investigator and caregiver. An EEG is considered a minimal risk procedure. A trained technician will apply scalp electrodes and record the EEG for up to 60 minutes. There is no known risk to EEG. The scalp electrodes may cause skin irritation. The test can be terminated early if necessary to relieve irritation.
- Neurocognitive testing (ages 3-25 years). Neurocognitive testing will be performed under the supervision of a child psychologist according to clinical protocols with use of standard of care validated instruments for assessment of selected age-appropriate domains of neurocognitive function. Neurocognitive testing is considered a minimal risk procedure. These tests may require 4 hours total for completion. Neurocognitive testing may ask questions that make persons feel uncomfortable, anxious, angry, or sad. Questionnaires can also cause fatigue. To minimize risk, study personnel will explain that all questions are optional, and that a question can be skipped if the participant prefers not to answer. The platform for the questionnaires will allow participants to take a break and come back to where they left off.
- Blood collection for clinical laboratory and biorepository: maximal single phlebotomy volume for newborn to <72 months (0-5 years) will be will be derived using a blood volume-by-weight calculation of 2 mL/kg for <5% total blood volume; maximal single phlebotomy volume for ages 6 years to under 10 years is 25 mL; maximal single phlebotomy volume for ages 10 years to under 25 years is 38 mL. Phlebotomy for blood collection is considered a minimal-risk procedure. Clinical standard of care phlebotomy procedures will be used. Phlebotomy is associated with minor temporary discomfort. There is a risk of bruising and a very small amount of bleeding associated with the blood collection. There is also a very small risk of infection at the site.
- Microbiome biospecimen collection (ages 3-25 years). There is no known risk to specimen collection. Collection of urine and stool may be embarrassing and messy and can be performed at home and is optional for age <7 years per judgment of site investigator and caregiver. Nasal swabs can cause local discomfort or pain.

Risks of use of mobile health technology

Commercial products or devices made by third party companies, including wearable fitness trackers, wearable sleep monitors, mobile apps for use on smartphone and tablets, websites and web apps, and types of computer software that permit screen sharing, record keystrokes, gain access to device files and/or use location tracking technology may be used to collect study data. These devices will be used in accord with the Terms of Service and/or the End User License Agreements (EULA) provided by the product or device vendor. Use of such products and devices is associated with loss of privacy and risk of breach of confidentiality. These products and devices will only be used to collect study data in ages 6-25 years with IRB approval and if the participant has agreed to all applicable Terms of Service and EULAs.

Risks of Incidental findings
Biospecimen test results determined in CLIA-certified clinical laboratories and imaging and other clinical testing results determined by licensed medical professionals that are analytically valid may be provided to their local site research team or recorded in the participant medical record and will be reviewed by the Principal Investigator or other designated licensed medical professional at each site. If the Principal Investigator or licensed designee determine that the result is clinically significant or medically actionable, the participant will be contacted by telephone or in-person to explain the test findings within one week of the return of the test results. The participant will also be advised to follow up with their primary care physician. Any additional testing ordered by the primary care physician will be for clinical indication and will be paid by the participant or their insurance company. Disclosure of such incidental findings can cause or increase anxiety, and may result in increased clinical care costs to the participant.

Test results determined in research laboratories that cannot be validated in CLIA-certified clinical laboratories will not be recorded in the medial record and will not be returned to the participant.

Risks of Genetic Testing
A known risk of whole genome sequencing (WGS), as with all next generation sequencing techniques, is the incidental discovery of potentially pathogenic genetic variants during the course of the planned research. The WGS studies for the RECOVER study will be performed in a CLIA-certified laboratory with results interpreted by a board-certified clinical molecular geneticist or molecular genetic pathologist or equivalent. Analytically valid replicated results from the CLIA-certified laboratory that are defined as clinically actionable according to the standards and guidelines defined by the American College of Medical Genetics and Genomics and the Association for Molecular Pathology (ACMG-AMP) guidelines may be disclosed to the participants if all of the following criteria are met:(33-35)

- The genetic finding has important health implications for the participant and the associated risks are established and substantial.
- The genetic finding is actionable, that is, there are established therapeutic or preventive interventions or other available actions that have the potential to change the clinical course of the disease.
- The test is analytically valid and the disclosure plan complies with all applicable laws.
- During the informed consent process or subsequently, the study participant has opted to receive his/her individual genetic results.

All disclosure of clinically actionable genetic results will be guided by the RECOVER WGS Core Laboratory in collaboration with the RECOVER Clinical Science Core. The consent form will inform participants of the potential for return of actionable results from WGS and the potential risks associated with the disclosure of the genetic information. For participants who elect to be informed of their clinically actionable genetic results, the validated, replicated result will be shared with the site PI or designated study personnel, who will use their local process and policies for re-identification of the participant and referral if needed for evaluation and counseling, which may include involvement of their local genetics team and/or the participant's cardiologist or other healthcare providers. Participants who reach the legal age of majority during the study will be re-consented and given the opportunity to opt-in or opt-out of return of genetic information.

There is a risk when performing genetic testing on participants and their parents that the genetic testing may detect cases of non-paternity (i.e., where the father of the child someone other than who it was thought to be). Non-paternity will be kept in the strictest confidence and will not be shared with the participant, parent(s), or family members.

Disclosure of individual genetic test results can cause or increase anxiety, damage family relationships, and/or compromise future insurability and employability. Some people involved in genetic studies feel anxious about the possibility of carrying (or their child carrying) an altered gene that may place them at risk or that might be passed on to subsequent generations. Genetic counseling will be provided by their clinical health care providers. Even without disclosure of individual results, genetic findings may be used to support harmful stereotypes, stigmatize, or discriminate against members of a socially defined group such as race or ethnicity; which could further impact employability as well as marital, adoption, and child-custody opportunities.

6.2 Known Potential Benefits

For most participants and caregivers in this observational study, there will be no direct benefit. It is possible that results of certified clinical laboratory testing available in the medical record will provide primary care physicians of the participants with clinically useful and medically actionable information. Although an individual participant may not benefit from participation, the results of the study will make important contributions to the understanding of healthcare providers, caregivers, parents, and patients about the long-term outcomes after SARS-CoV-2 infection.

Currently, there is no known direct benefit from the participation of the participant in the RECOVER biorepository. However, future biospecimen analyses may help physicians provide better answers to families' questions regarding causes, risk, and recurrence risks. It may also provide clues to future interventions and/or treatments.

At the end of the study, participants and/or parents will be sent a description of the aggregate study results in lay language. In this study, families will be given the contact information for the study PI and/or coordinator, in the event the patient wishes to discuss the results or has questions. If desired, a separate notification will also be provided to the treating/referring physician (cardiologist, etc.) describing overall study results.

7 Objectives and Purpose

The objective of this proposed study is to enhance knowledge of children's recovery from SARS-CoV-2 infections in order to support development of novel diagnostic and therapeutic interventions. Overarching scientific objectives are as follows:

1. Characterize the incidence and prevalence of sequelae of SARS-CoV-2 infection occurring >30 days after study entry across the early life spectrum from age newborn-25 years.
2. Characterize the spectrum of clinical symptoms, subclinical organ dysfunction, natural history, and distinct phenotypes identified as sequelae of SARS-CoV-2 infection occurring >30 days after study entry infection across the early life spectrum from age newborn-25 years.
3. Define the biological mechanisms underlying pathogenesis of the sequelae of SARS-CoV-2 infection occurring >30 days after study entry across the early life spectrum from age newborn-25 years.

7.1 Specific Aims

The following aims are proposed to support achievement of the scientific objectives of the study:

Aim 1. Characterize the incidence, prevalence, and long-term sequelae, including clinical and biological features, severity, and distinct sub-phenotypes following SARS-CoV-2 infection across the early life spectrum from ages newborn-25 years.

1a. Define PASC phenotypes, sub-phenotypes, and severity based on clinical and biological features across the early life spectrum from ages newborn-25 years.

1b. Estimate the incidence of PASC phenotypes (new onset or exacerbation) among children with SARS-CoV-2 infection or born in the context of maternal SARS-CoV-2 infection during pregnancy compared with uninfected controls followed over the same time interval.

1c. Estimate the incidence and prevalence of clinical and subclinical organ injury/disease after SARS-CoV-2 infection.

Aim 2. Characterize the clinical course and recovery of acute and post-acute sequelae over time and determine associated risk factors for PASC among SARS-CoV-2 infected individuals compared to SARS-CoV-2 infected individuals without PASC and compared to uninfected individuals across the early life spectrum from ages newborn-25 years.

2a. Characterize the patterns of outcomes of acute and post-acute sequelae of SARS-CoV-2 infection over time.

2b. Determine whether pre-infection and peri-infection risk and resiliency factors (e.g., social determinants of health, family dynamics, demographic, behavioral, and biological factors, preexisting clinical and subclinical co-morbidities and acute infection severity and treatment) are associated with sequelae of SARS-CoV-2 infection and their resolution over time.

2c. Compare the prognostic significance of subclinical organ injury/disease for incident clinical disease over time among SARS-CoV-2 infected versus uninfected individuals.

Aim 3. Define the pathophysiology of and mechanisms associated with the development of acute and post-acute sequelae, including the direct and indirect effects, of SARS-CoV-2 infection on symptom onset and potential modifiers across the early life spectrum from ages newborn-25 years.

3a. Evaluate the direct and indirect effects of SARS-CoV-2 infection on the development of acute and post-acute sequelae, including potential mediation by severe disease (e.g., ICU stay and/or intubation), treatment (e.g., steroids), and pandemic-related stressors.

3b. Determine whether SARS-CoV-2 infection modifies the trajectory of prior organ dysfunction, and/or the risk of developing new organ injury compared with pre-pandemic status, and identify possible pathophysiological mechanisms.

3c. Compare the long-term outcomes of children with MIS-C and SARS-CoV-2 infected children without history of MIS-C, and elucidate the mechanisms and pathobiology behind these events.

3d. Characterize the impact of clinical manifestations of PASC on measures of child development in caregiver-child dyads comparing:

i. Children with SARS-CoV-2 infection with PASC to those with SARS-CoV-2 infection without PASC, and those without SARS-CoV-2 infection.
ii. Children with and without caregiver history of SARS-CoV-2 infection including infants born to mothers with SARS-CoV-2 infection during pregnancy.

8 Study Design and Endpoints

8.1 Overall Study Design

This is a combined retrospective and prospective, longitudinal, observational meta-cohort of individuals who will enter the cohort with and without SARS-CoV-2 infection and at varying stages before and after infection (see Study Overview Figure 1). Individuals with and without SARS-CoV-2 infection and with or without PASC symptoms will be followed to identify risk factors and occurrence of PASC. This study will be conducted in the United States and participants will be recruited through inpatient, outpatient, and community-based settings. No recruitment materials will be used without first being approved by the IRB.

Each enrolled subject will undergo a tailored tiered testing approach based on history of SARS-CoV-2 infection, presence of PASC symptoms, age range, and other considerations specific to each sub-cohort of the RECOVER meta-cohort. Subjects will be identified for participation in post-acute Tier 2 based on their history of COVID exposure and PASC symptoms as determined in Tier 1 for post-acute participant and

uninfected controls, and at the week 8 visit for acute Tier 2 participants. Subjects will be selected for participation in Tier 2 and Tier 3 to achieve the enrollment targets as summarized in Figure 1 and to achieve pre-specified targets for age distribution and diversity.

8.2 Recruitment procedures

Recruitment into the pediatric protocol will occur through direct contact with potential participants in clinical settings, electronic health record query, social media platforms, websites, outreach to community-based organizations, and conventional mass media (radio and print publications). Flyers, post-cards, business cards, narrated slide shows, animations, and videos may be used across different media platforms to provide information about study participation. All materials used in recruitment activities will be IRB-approved, and any recruitment information sent by email will use a secure encrypted email platform.

If the potential subject has provided prior consent to be contacted for research at their study site institution, the site study team may create a query in the local electronic health record system to identify potential subjects based on study entry criteria (based on age, diagnostic codes, SARS-CoV-2 testing results, or other information in the electronic health record found to be related to the PASC phenotype). A secure email will be sent to potential subjects to solicit interest in the study, with instructions for contact of the study team if interested to participate. Once contact has been established with a potential subject, an IRB-approved eligibility script and screener may be used by the enrolling site. The study Principal Investigator or designated study staff members may provide additional IRB-approved information to the potential subject as described below and may schedule a study visit. Other PHI or medical information will not be available to the study team. The electronic health record query may be repeated throughout the duration of the 4-year study. All query responses will be de-identified and retained. A waiver of authorization is in place at participating sites so study teams can review patient medical records to identify potential subjects who meet eligibility criteria. If a potential subject requests information regarding opting out of further recruitment for all research, subjects will be directed to site Principal Investigator or designated study team member.

Once potential subjects have been identified, the study team may need to notify the treating physician that they have patients eligible to participate. If notification to the treating physician is necessary, one or more of the following methods will be used to notify the treating physician:

1. The treating physician may be given a list, advertisement, letters or oral script to use when contacting potential subjects
2. The treating physician and site Principal Investigator may send a letter to all potential subjects (letter must have both names)
3. If the treating physician agrees, the study team may directly contact potential subjects on behalf of the treating physician by letter, phone, email, or an electronic medical record patient portal.

Once contact is made, approved recruitment language will be used to communicate the reason they are being contacted and potential subjects will be asked if they are interested in participating in this specific study. An IRB-approved eligibility script and screener may be used by the enrolling site. Should the potential subjects agree, the study team will provide the subjects with information regarding the next steps for participation.

8.3 Cohort and Case-Noncase Study Recruitment Pools

The SARS-CoV-2 Pediatric Recovery Cohort PASC Study will enroll individuals ages newborn through 25 years with and without SARS-CoV-2 infection, defined as probable, suspected or confirmed according to the WHO criteria, and at varying stages before and after infection in a meta-cohort design.
Figure 2: Schematic of Meta-Cohort Recruitment Cohorts

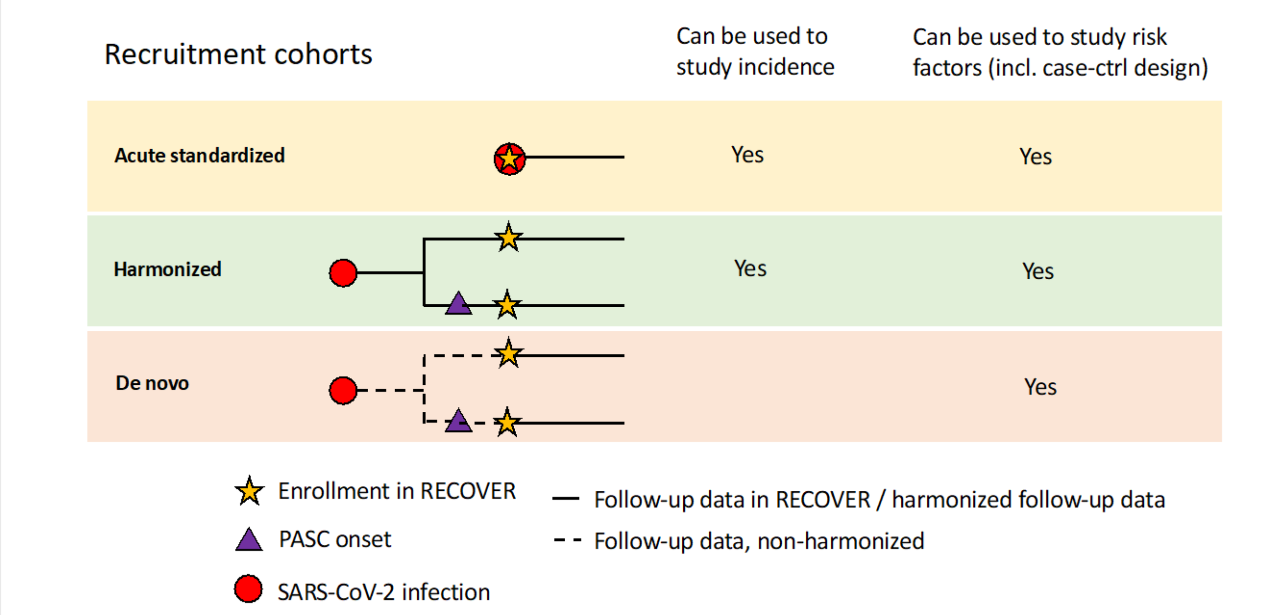

Figure 2 Key Points

- Three different types of cohorts are included:

1. "Acute standardized cohort," which includes participants with a new, acute COVID infection;
2. "Harmonized cohorts," which include existing cohorts that contribute data that is both previously collected and newly collected; and
3. "De novo cohorts," which are new cohorts collecting data from the past and data going forward

- The acute standardized and the harmonized cohorts can be used to study the incidence of COVID infections in children and young adults, or the rate of new infections that occur.
- All three cohort types can be used to study risk factors associated with developing the long-term effects of COVID

8.3.1 Overview of cohort types contributing to the RECOVER meta-cohort

Extant, clinical and de novo (newly enrolled in RECOVER) cohorts:

- SARS-CoV-2 infected children and young adults with and without current or prior PASC-like symptoms, including infected individuals with history of MIS-C, and infants born in the context of maternal SARS-CoV-2 infection during pregnancy (congenitally exposed).
- SARS-CoV-2 uninfected children, young adults, and infants born to uninfected mothers
- Enrollment procedures and RECOVER data collection procedures will be harmonized with the extant research protocol for participants enrolled in other studies; separate RECOVER consent will be signed.

Acute cohort:

- Newly SARS-CoV-2 infected individuals (≤30 days since onset of symptoms or positive laboratory testing)
- Contemporaneous SARS-CoV-2 uninfected individuals selected from the same population as newly SARS-CoV-2 infected individuals
- Eligibility for the post-acute phase of Tier 2 in the acute cohort will be determined at the 8-week acute Tier 2 visit as described in Section 11.

Post-acute cohort:

- Post-acute infected individuals (>30 days after initial symptoms or positive laboratory testing) in the extant, clinical and de novo cohorts, including infected individuals with history of MIS-C, and infants

born in the context of maternal SARS-CoV-2 infection during pregnancy (congenitally exposed), will be enrolled after initial SARS-CoV-2 infection.

- If available, relevant retrospective data prior to enrollment will be extracted from the electronic health record or existing research and clinical cohort data.
- Uninfected individuals will be derived from a similar population with respect to age, sex, race and ethnicity, geographic origin, sociodemographics, and time of enrollment as the infected individuals.
- Individuals in Tier 1 will be classified as post-acute infected with or without history of prior laboratory testing or symptoms of SARS-CoV-2 infection. An index date for these individuals may be the time of a past known family or school exposure, past symptoms, or at study entry if no history of exposure or symptoms is known.

8.3.2 Categories of recruitment pools in the RECOVER meta-cohort

Based on these types of cohorts available within the RECOVER meta-cohort, the RECOVER study population will include 9 categories of participants:

1. Acute infected participants in the main cohort (ages newborn-25 years, symptomatic and asymptomatic),
2. Post-acute infected participants in the main cohort (ages newborn-25 years, symptomatic and asymptomatic),
3. Uninfected participants who will serve as comparisons for the acute and post-acute infected participants in the main cohort,
4. Infected participants recruited from the Adolescent Brain Cognitive Development (ABCD) study,
5. Uninfected participants recruited from the ABCD study,
6. Infants and toddlers (≤3 years of age) born in the context of maternal SARS-CoV-2 infection during pregnancy (congenitally exposed)
7. Uninfected control infants and toddlers (≤3 years of age) not born in the context of maternal SARS-CoV-2 infection during pregnancy, and
8. Children and young adults with history of MIS-C

Additional procedures participant enrollment within these categories is described below. Primary caregivers of participants will also be enrolled in the study for limited data collection.

Acute and post-acute infected participants in the main cohort. Participants with SARS-CoV-2 infection will be recruited at or within 30 days after infection (acute) or more than 30 days after infection (post-acute).

Symptomatic acute infected participants will meet the WHO criteria for suspected, probable, or confirmed infection and may include both vaccinated and unvaccinated participants. For participants with confirmed SARS-CoV-2 infection, the index date is the infection date, i.e., the date at which a participant meets test result inclusion criteria; for participants with probable or suspected infection, infection date is an approximate date at which infection occurred (by self-report or documented by chart review). For participants with asymptomatic infection and no known exposure history to determine data of infection, no index date will be assigned. All symptomatic acute participants with and without PASC symptoms will be selected to be followed longitudinally beyond the initial 8-week acute phase for the duration of the study.

Symptomatic post-acute infected participants in the main cohort will be recruited based on meeting WHO criteria for a suspected, probable, or confirmed case of SARS-CoV-2 infection. Participants with history of MIS-C who have not been previously enrolled in another research cohort may be enrolled in the pediatric main cohort. Participants with history of MIS-C may be identified in RECOVER PASC consortium EHR cohorts, active surveillance of the EHR at RECOVER PASC consortium pediatric cohort sites, existing patient advocacy social networks for MIS-C, referral from other health care providers at the RECOVER PASC consortium pediatric cohort health care systems, and self-referral. Recruitment from PASC clinics or other referral sources based on the presence or absence of post-acute symptoms (rather WHO criteria) will be minimized. Self-referral patients will be accepted but the combination of participants recruited based on the presence of PASC symptoms and self-referral patients with reported PASC symptoms cannot account for more than 50% of enrolled patients within any single racial/ethnic group. Participants recruited based on the presence of post-acute symptoms and self-referral patients with post-acute symptoms will be excluded from prevalence/incidence calculations due to selection bias.

Asymptomatic acute and post-acute infected participants may be identified by positive PCR or antigen screening test in the medical history or by positive nucleocapsid antibody test in the medical history or from results of Tier 1 nucleocapsid antibody testing, or symptomatic infected participants will meet WHO criteria for suspected, probable or confirmed infection that are not symptom-based. For asymptomatic infected participants, the index date is the historical date of exposure if known, or the date of study entry. Asymptomatic acute infected participants will be selected to be followed longitudinally beyond the initial 8-week acute phase for the duration of the study, as determined by the presence of suspected PASC symptoms.

Acute and post-acute uninfected participants in the main cohort. Uninfected subjects must meet the following conditions at the time of RECOVER screening/enrollment:

1. Never met the WHO criteria for a suspected, probable, or confirmed case of SARS-CoV-2 infection,
2. No known history of SARS-CoV-2 test from a respiratory specimen (PCR or antigen testing) and
3. No known history of positive SARS-CoV-2 nucleocapsid protein antibody (if vaccinated) and spike protein antibody test (if not vaccinated).

Uninfected participants will be recruited contemporaneously with acute participants and will be frequency matched on sex, age, race/ethnicity, and vaccination status. For uninfected participants, the *index date* is defined as the date of RECOVER enrollment. A subset of uninfected participants will be followed longitudinally for the duration of the study. Uninfected control subjects will be recruited from screening test records (negative tests due to household or school contacts with no symptoms at the time) and from well-child ambulatory clinics, vaccination clinics, or other non-COVID related clinical encounters contemporaneous with an identified acute or post-acute case from the same clinic setting. Acute and post-acute uninfected participants must have no known history of positive screening SARS-CoV-2 test and no known history of a positive antibody test (spike and nucleocapsid protein) from medical records or from Tier 1 testing. The uninfected subject may be the sibling or classmate of another participant in RECOVER. There is no limitation on the number of siblings who can be enrolled across the study and no limitation on the number of children who can be enrolled from a single household. When possible, at recruitment, the uninfected and infected participants may also be matched on important covariates such as sex, age, race / ethnicity, hospitalization, and vaccination status. The date of a negative SARS-CoV-2 test (if available) or the date of a well-child visit from the same recruitment pool contemporaneous with an infected case will be considered the index date. A proportion of post-acute uninfected participants will be selected based on the presence or absence of PASC symptoms to be followed longitudinally for the duration of the study (n=600, Figure 1). Uninfected control participants selected for longitudinal follow-up in Tier 2 will be randomly assigned to Tier 2 procedures matching the acute infected main cohort (n=200) or Tier 2 procedures matching the post-acute infected main cohort (n=400).

Infected and uninfected children recruited from the Adolescent Brain Cognitive Development (ABCD) study (ages 12-17 years). The Adolescent Brain Cognitive Development (ABCD) Study is an NIH-funded study of brain development and child health. Participants belonging to this extant cohort include both adolescents who were infected with SARS-CoV-2 prior to RECOVER enrollment and those who were not. Most ABCD cohort participants will contribute Tier 1 data only and will not be followed longitudinally; however, a small proportion of ABCD Study participants may contribute longitudinal Tier 2 data to RECOVER depending in part on the presence of suspected PASC symptoms and geographic proximity to other pediatric cohort sites. Infected post-acute cases and non-infected controls will be identified according to the same criteria as the main cohort. Index date determination for these participants will follow the index determination for post-acute infected and uninfected participants.

Children (≤3 years of age) born in and out of the context of maternal SARS-CoV-2 infection during pregnancy. Children ≤3 years of age born to a childbearing parent with history of SARS-COV-2 infection during pregnancy will be enrolled in the study from existing research cohorts. Children ≤3 years of age born to a childbearing parent without history of SARS-COV-2 infection during pregnancy will also be enrolled from the same existing research cohorts. These participants will all be followed longitudinally until the age of 48 months. It is anticipated that these infants will mostly be recruited between the ages of 9 and 18 months. Caregiver dyads are not being enrolled for this cohort. The mothers of these children may participate in the RECOVER adult cohort.

Children (ages newborn-5 years) from the pediatric cohorts who were infected with COVID after birth (excluding those with only in utero exposure by being born to a childbearing parent with history of SARS-COV-2 infection during pregnancy) and uninfected controls will be enrolled in the study. These participants will be a part of the main cohort and will complete age-appropriate assessments.

Children and young adults with history of MIS-C in extant cohorts. MIS-C is considered a subset of PASC. Participants with history of MIS-C according to the CDC case definition (36) will be recruited from RECOVER sites participating in existing research cohort studies. These participants will all be followed longitudinally until 2 years after study enrollment (up to 5 years after index infection). The existing research cohort data will be harmonized with RECOVER prospective data collection.

Primary caregivers of participants. A primary caregiver is the adult responsible for the care of the enrolled child and resides in the same household as the child. For children ages newborn to 17 years, the caregiver will be the primary respondent for child health questionnaires. Caregivers will answer questions about their own SARS-CoV-2 exposure and experience with COVID-19, if any, as well as provide information about household social determinants of health and limited biological specimens.

Other biological parents of participants. If the primary caregiver is a biological parent of the child or young adult who is willing to participate in the study, the other biological parent, if available, may be enrolled to provide a home sample of saliva for DNA analysis.

Recruitment targets by demographics, symptoms, and calendar time of infection. Recruitment will occur across all pediatric sites in the RECOVER consortium. Within each site, recruitment of infected individuals will be monitored to ensure balance across strata as follows:

- 50% of participants will be symptomatic and between 15-30% will be asymptomatic during the 30 days after SARS-CoV-2 infection.
- 50% of participants will be female,
- 53% of participants will be non-Hispanic Whites, 16% non-Hispanic Blacks, 27% Hispanic/Latinx, and 4% Asian Americans, Native Hawaiians, Pacific Islanders, American Indians, and Native Alaskan.

Uninfected participants will be recruited from the same communities and sources as infected participants. Recruitment will be stratified to match the SARS-CoV-2 infected group as follows:

- 50% of participants will be female,
- 53% of participants will be non-Hispanic Whites, 16% non-Hispanic Blacks, 27% Hispanic/Latinx, and 4% Asian Americans, Native Hawaiians, Pacific Islanders, American Indians, and Native Alaskan.

All participants (acute, post-acute and uninfected controls) will contribute to aims according to their SARS-CoV-2 infection/PASC history and clinical course during prospective follow-up. Patients without SARS-CoV-2 infection may include individuals with or without hospitalization in the prior three months. A proportion of individuals with prior or acute SARS-CoV-2 infection will be selected to be followed prospectively from enrollment, based on presence or absence of suspected PASC symptoms, with collection of relevant retrospective data when available. Selection for prospective follow-up will be determined by the Tier 2 targets for symptomatic vs. asymptomatic infection, PASC positive vs. PASC negative symptom status, and race and ethnicity as described above.

9 Study Enrollment and Withdrawal

9.1 Inclusion Criteria

In order to be eligible to participate in this study, an individual must meet all of the following criteria:

9.1.1 Infected Cohort:

Patients will be eligible for inclusion according to the following criteria:

i. Ages newborn-25 years
ii. Infected individuals will have suspected, probable, or confirmed SARS-CoV-2 infection as defined by WHO criteria since January 1, 2020 or have been born to a mother meeting these criteria during pregnancy (congenitally exposed)
iii. Children/young adults with or without history of MIS-C are eligible
iv. Children/young adults with or without history of SARS-CoV-2 vaccination are eligible
v. Children/young adults with evidence of past SARS-CoV-2 infection based on serum antibody profile are eligible (with or without history of acute symptoms)
vi. Children/young adults with recurrent SARS-CoV-2 infections and those with post-vaccination (breakthrough) infections are eligible to participate
vii. Participants are eligible without exclusion related to sex, race/ethnicity, geography, nationality, severity of disease, or underlying health conditions

9.1.2 Children/Young Adults with Suspected SARS-Cov-2 Infection

a) Children/young adults who meet these clinical criteria:

At least one of these clinical criteria:

i. Acute onset of fever and cough OR
ii. Acute onset of ANY THREE OR MORE of the following signs or symptoms: fever, cough, general weakness /fatigue, headache, myalgia, sore throat, coryza, dyspnea, anorexia/nausea/vomiting, diarrhea, altered mental status.

AND at least one of these epidemiological criteria:

i. Residing or working in an area with a high risk of transmission of virus: closed residential, school or camp settings anytime within the 14 days before symptom onset; OR
ii. Residing or travel to an area with community transmission anytime within the 14 days before symptom onset; OR
iii. Any known household contact or any member of the household working in any health care setting, including within health facilities or within the community; anytime within the 14 days before symptom onset.

b) A patient with history of **severe acute respiratory illness (SARI):**
SARI: acute respiratory infection with history of fever or measured fever of ≥ 38 C°; and cough; with onset within the last 10 days; and requires hospitalization

c) An asymptomatic person not meeting epidemiologic criteria with a positive SARS-CoV-2 Antigen-RDT.

9.1.3 Children/Young Adults with Probable SARS-Cov-2 Infection

a) A patient who meets clinical criteria above AND is a contact of a probable or confirmed case or linked to a COVID-19 cluster; OR
b) A suspect case with chest imaging showing findings suggestive of COVID-19 disease; OR
c) A person with recent onset of anosmia (loss of smell) or ageusia (loss of taste) in the absence of any other identified cause

9.1.4 Children/Young Adults with Confirmed SARS-Cov-2 Infection

a) A person with a positive Nucleic Acid Amplification Test (NAAT); OR
b) A person with a positive SARS-CoV-2Antigen-RDT AND meeting either the probable case definition or suspect criteria A OR B; OR
c) An asymptomatic person with a positive SARS-CoV-2 Antigen-RDT who is a contact of a probable or confirmed case

9.1.5 Children/Young Adults with Asymptomatic SARS-CoV-2 Infection

a) A person without history of acute COVID-19 symptoms who has one or more of the epidemiological exposures for suspected infection and who also meets criteria b or c for suspected or probable infection, or who meets any of the criteria for confirmed infection

b) A person without history of acute COVID-19 symptoms who has positive nucleocapsid antibody test result in medical history or Tier 1 testing with or without NAAT or RDT testing or known contact to a probable or confirmed case.

9.1.6 Non-Infected Cohort

A person who meets the following criteria will qualify for enrollment as a non-infected control subject:

a) Does not meet WHO criteria for a suspected, probable, or confirmed case of SARS-CoV-2 infection AND
b) Does not have serological evidence of past asymptomatic SARS-CoV-2 infection in medical history or Tier 1 testing, AND
c) Lives in the same communities or recruited from the same sources as those in the SARS-CoV-2 infected cohort, AND
d) Either not hospitalized for any reason in prior 3 months, or hospitalized (with or without ICU stay) within the prior 3 months
e) Uninfected individuals may participate independent of their vaccination status
f) Uninfected individuals who develop SARS-CoV-2 infection during the study period will be reassigned to the SARS-Cov-2 infected group and will be considered to have been enrolled prior to SARS-CoV-2 infection.

9.1.7 *Children (≤3 years of age) born in and out of the context of maternal SARS-CoV-2 infection during pregnancy.*

- Children ≤3 years of age born to a childbearing parent with history of suspected, probable, or confirmed SARS-COV-2 infection during pregnancy (according the same criteria listed for the infected child cohort) will be enrolled in the study from existing research cohorts at the maternal fetal medicine sites in the RECOVER. network.
- Children ≤3 years of age born to a childbearing parent without history of SARS-COV-2 infection during pregnancy (according to the same criteria listed for the non-infected child cohort) will also be enrolled from the same existing research cohorts at maternal fetal medicine sites in the RECOVER network.

9.1.8 Children with MIS-C

Children/young adults with SARS-CoV-2 infection who have history of MIS-C meeting the CDC definition:

- An individual aged <21 years presenting with fever*, laboratory evidence of inflammation**, and evidence of clinically severe illness requiring hospitalization, with multisystem (≥2) organ involvement (cardiac, renal, respiratory, hematologic, gastrointestinal, dermatologic or neurological); AND
- No alternative plausible diagnoses; AND
- Positive for current or recent SARS-CoV-2 infection by RT-PCR, serology, or antigen test; or exposure to a suspected or confirmed COVID-19 case within the 4 weeks prior to the onset of symptoms.

*Fever ≥38.0°C for ≥24 hours, or report of subjective fever lasting ≥24 hours
**Including, but not limited to, one or more of the following: an elevated C-reactive protein (CRP), erythrocyte sedimentation rate (ESR), fibrinogen, procalcitonin, d-dimer, ferritin, lactic acid dehydrogenase (LDH), or interleukin 6 (IL-6), elevated neutrophils, reduced lymphocytes and low albumin

Young adults with past history of MIS-C with current ages 22-25 years are eligible to participate.

9.1.9 Primary Caregiver Entry Criteria

a) A primary caregiver is defined as an individual, such as a family member (biological or nonbiological) or legal guardian, who is responsible for the care of the enrolled child and resides in the same

household as the child. When possible, the primary caregiver identified at study entry will remain in the same role throughout the study.

b) The designated primary caregiver is the family member (biological or nonbiological) or legal guardian who spends the most time with the child or young adult, has substantial responsibility for taking care of her/him on a daily basis, and is most knowledgeable about her/him.
c) If two or more persons share equally in the caregiver responsibilities for the child or young adult, the person selected by the family to fill out study forms both about themselves and the child will be designated the primary caregiver.
d) If a primary caregiver has not reached the legal age of majority in their jurisdiction, the minor caregiver's parent/legal guardian will provide consent for participation, with assent provided by the minor caregiver.
e) A nonbiological primary caregiver or legal guardian serving as the designated primary caregiver must be above the legal age of majority in their jurisdiction.
f) The designated primary caregiver cannot be a babysitter or other childcare provider who receives money to care for the child.

9.1.10 Biological Parent Entry Criteria

a) If the designated primary caregiver who is participating in the study is a biological parent of the enrolled child or young adult, the other biological parent may be enrolled to provide a home sample of saliva for DNA analysis.

9.2 Exclusion Criteria

An individual who meets any of the following criteria will be excluded from participation in this study:

a) Any child, young adult, designated caregiver, or other biological parent who in the opinion of the site investigator may be at increased risk of adverse events during participation in the study, or who may not be able to complete study procedures due to co-morbid disease or disability.
b) Any young adult above the age of majority who lacks capacity to provide consent
c) Nonviable neonates and neonates of uncertain viability as determined by the treating physician
d) Any child, young adult, or designated caregiver with co-morbid illness with expected survival <2 years
e) Any child with a plan for adoption or for whom the state is the legal guardian
f) Any young adult, designated caregiver or other biological parent who is incarcerated, or who lacks capacity to provide consent
g) Currently enrolled in the study: Understanding the Long-term Impact of COVID-19 in Adults.
    - This applies only to Pediatric participants aged 18-25 years old who are eligible to enroll as a young adult on the pediatric protocol. Young adult participants cannot be enrolled in both the Pediatric and Adult protocols.
    - This does not apply to Caregivers in the Pediatric Protocol.
    - Individuals of child-bearing potential enrolling as a pregnant participant on the Adult protocol cannot also enroll as a young adult or Caregiver on the Pediatric protocol.

9.3 Vulnerable Subjects

This observational study is conducted in subjects ages newborn-25 years, their primary caregivers, and optionally the other biological parent if the designated primary caregiver is a biological parent. Some of the participants may be women who are pregnant or breastfeeding. Some of the caregivers may be under the age of majority in their local jurisdiction. Study participation in caregivers is limited to questionnaires and collection of blood and saliva biospecimens. For children and young adults enrolled in the study, the study procedures are considered to be minimal risk (Tiers 1 and 2), or a minor increase over minimal risk (some of the procedures in Tier 3).

For Tier 3 procedures, symptom-limited exercise testing and sputum induction are considered to include a minor increase over minimal risk. This means that the increase in the probability and magnitude of harm is only slightly more than minimal risk, and that any potential harms associated with the procedure will be transient and reversible in consideration of the nature of the harm, and there is no or an extremely small probability that subjects will experience significant pain, discomfort, stress or harm. Exercise is a normal

activity in children, and the level of exercise will be stopped as directed by the child symptoms. Monitoring of electrocardiogram, heart rate, blood pressure, and symptoms by a trained professional will reduce risk of harm. Sputum induction is considered as a minor increase over minimal risk due to a small risk of bronchospasm (0.1%) and possible side effects with levalbuterol pre-treatment. To minimize risk, participants with history of bronchospasm will be excluded. Oxygen saturation and spirometry will be monitored during the procedure. Trained technicians under supervision of a licensed medical professional will be present with access to emergency bronchodilator medicines if needed.

Inclusion of exercise testing and sputum induction for collection of lung microbiome biospecimens is vitally important to the understanding of the long-term effects of SARS-CoV-2 infection in children. There is clear and significant evidence that these procedures will provide important information to further the understanding of the etiologies, prevention, diagnosis, pathophysiology, or alleviation or treatment of PASC in children.

Inclusion of pregnant subjects is vitally important to the understanding of the long-term effects of SARS-CoV-2 infection in pregnant women and their child. The proposed study procedures do not require radiation exposure or other procedures that pose risk to pregnant or breastfeeding research subjects. If a participant younger than the age of majority is pregnant, or becomes pregnant, during the course of the study, the consent process may be modified if the participant is allowed under state and local regulation to provide consent for themselves. Otherwise, consent from two parents will be obtained and participant assent will be sought.

There is possible direct medical benefit to participants in the study related to return of medical information to the participant and their primary care physician, and potential future benefit related to discoveries about PASC from study data and biospecimens.

9.4 Strategies for Recruitment and Retention

9.4.1 Study Recruitment Strategy and Sampling

Recruitment of children and young adults with and without SARS-CoV-2 infection or born to a mother with SARS-CoV-2 infection during pregnancy may be stratified to ensure adequate representation by age, sex, race/ethnicity.

Children and young adults with acute SARS-CoV-2 infection will be recruited from extant research and clinical cohorts not selected for the study of PASC, including:

- Acute case surveillance testing in PASC consortium pediatric cohort health care systems
- Community engagement cohorts for surveillance of acute cases
- Acute case surveillance in extant populations enrolled in aim 1
- State and local public health resources
- PASC consortium EHR cohorts
- Participant self-referral

Recruitment for acute SARS-CoV-2 infection may use CDC data to target regions with higher case rates.

Children and young adults with post-acute SARS-CoV-2 infection including children born to a mother with SARS-CoV-2 infection (>4 weeks after infection) will be recruited from:

- Post-acute case surveillance in PASC consortium pediatric cohort health care systems, including surveillance of infants born to a mother with SARS-CoV-2 infection
- Community engagement surveillance for post-acute cases
- Post-acute cases identified in extant populations
- COVID vaccine clinics
- Existing research and clinical cohorts of post-acute children (including MIS-C research cohorts) and infants born to a mother with SARS-CoV-2 infection
- PASC consortium EHR cohorts
- Existing patient advocacy groups and social networks of PASC patients

- Referral from other health care providers at the PASC consortium pediatric cohort health care systems
- Participant self-referral

Children and young adults with SARS-CoV-2 infection will be sampled and recruited from an unbiased denominator of SARS-CoV-2 infected children, including a diversity of sites of care (e.g., not only patients seeking medical care at a post-COVID clinic or only patients cared for in academic medical centers) and severity of illness (i.e., not only from hospitalized patients).

For children and young adults without SARS-CoV-2 infection, participants will be sampled and recruited from similar communities, demographics, and sites and dates of care as those being recruited into the SARS-CoV-2 positive cohort. Screening testing after household or school contacts will be used when possible to identify uninfected control subjects. Uninfected control subjects may also be recruited from well-child ambulatory clinic visits or COVID vaccine clinics that are contemporaneous with acute and post-acute cases (within 6 months of acute index date). Recruitment may be stratified to match the SARS-CoV-2 positive group in terms of racial/ethnic diversity and severity of acute SARS-CoV-2 illness based on history of hospitalization and history of treatment in an intensive care unit.

Participants with history of MIS-C will be recruited from existing research cohorts, PASC consortium EHR cohorts, active surveillance of the EHR at PASC consortium pediatric cohort sites, existing patient advocacy social networks for MIS-C, referral from other health care providers at the PASC consortium pediatric cohort health care systems, and self-referral.

Caregivers will be recruited at the time of child enrollment when feasible. Caregiver participation is optional; eligible child and young adult participants can be enrolled with or without caregiver participation. The other biological parent of the child/young adult will be recruited at the time of caregiver recruitment, if the primary caregiver is a biological parent. Participation by the other biological parent is optional.

Sites are encouraged to recruit from special populations, defined as those individuals meeting at least one of the following:

Table 1: Special Populations with definitions and application scenarios

Non-hospitalized subjects with acute COVID-19	• <4 weeks since time of symptoms or positive testing. This only applies to acute cases being enrolled (**infection within last 30 days**)
Rural participants	• Individuals meeting criteria who reside in a rural area as defined by the Health Resources and Services Administration (https://data.hrsa.gov/tools/rural-health)
Medically underserved area	• Individuals meeting criteria who reside in a medically underserved area as defined by HRSA (https://data.hrsa.gov/tools/shortage-area/by-address)<br>• Defined as an area with too few primary care providers, high infant mortality, high poverty, or a high elderly population
Non-English speaking participant	Individuals meeting criteria who speak a primary language other than English; *To qualify for this designation, all subject-facing documents, including the consent form, will be translated into a subject's native language and approved by the IRB before non-English-speaking subjects are enrolled.

9.4.2 Retention Strategy

Subject retention will be promoted by promoting self-efficacy and self-monitoring behaviors with a home pulse oximetry device provided by the study site, by promoting family engagement with educational materials related to PASC symptom management, by creation of a patient web portal to provide access to study personnel for questions, and by using feedback received from subjects to enhance participant experience.

Additional efforts will be made to minimize attrition: 1) reminder calls for assessments, rescheduled if missed; 2) maintaining contact information (e.g., updating at each contact, obtaining alternate contacts, re-connecting in primary care), and ongoing contact with all subjects during the study by email (with encrypted messaging if PHI included) and by text (with subject consent); 3) compensation for subject participation in study procedures.

Contacts with participants may include: reminders for completion of study surveys, reminders for study appointments, a post-visit thank-you card or call, a quarterly newsletter, a birthday or greeting card, and a holiday or end-of-year card. We aim to design both culturally and religiously appropriate contact documents. Therefore, these contacts will be initiated by each site and will be conducted in the language of choice of the participant. In addition, because some religions (e.g., Jehovah's Witnesses) may not celebrate birthdays or holidays, specialized cards will be designed to accommodate these participants. All newsletters and cards sent to RECOVER respondents will be targeted for a 5th grade reading-level.

Subject response burden will be monitored in real-time during the study. If burden is found to be excessive, it will be reduced by altering the data collection strategy, such as by increasing the interval of assessments; reducing the number of data elements collected (eliminating rare symptoms); pre-filling prior responses to reduce data entry time for subjects; tightening criteria for Tier 2 and Tier 3 data collection; increasing the availability of home-based Tier 2/3 assessments; and/or increasing subject reimbursement. All such modifications will be approved by the IRB before implementation.

Recruitment and retention data will be monitored on an ongoing basis to compare target versus actual recruitment rates by site (stratified by age, race/ethnicity, sex); compare the number of expected surveys completed and biospecimens collected to target; and subject retention reports indicating the number of participants active, completed, and lost to follow-up.

9.5 Duration of Study Participation

Up to four years from the time of study entry, or from birth in the case of infants born to mothers with SARS-CoV-2 during pregnancy.

9.6 Total Number of Participants and Sites

Figure 1 provides an overview of the number of study subjects. Up to 19,300 dyads (children and young adults with and without history of SARS-CoV-2 infection and their primary caregiver), including 2,500 infants born to mothers with and without SARS-CoV-2 infection during pregnancy (congenitally exposed), and 800 children with MIS-C19,300.

Participants will be recruited from the enrolling sites that comprise the RECOVER Pediatric PASC Investigator Consortium. The RECOVER Pediatric and Prenatal/Infancy PASC Investigator Consortium study sites are listed in Appendix A.

9.7 Participant Withdrawal or Termination

Subjects are free to withdraw from participation in the study at any time upon request. The participant will provide a written notice of withdrawal to the study site PI.

Upon withdrawal, the subject's data will be excluded from data distribution for future analyses. Patient data will not be excluded from completed analyses.

Once the subjects withdraw participation, no more information will be collected. However, in cases when the data removal will affect the integrity of the study, all previously collected data will not be removed once the subjects withdraw from the study and subjects will be informed during the consenting process.

9.7.1 Reasons for Withdrawal or Termination

Participants may be prematurely terminated from the study in the following situations:

- Consent is withdrawn
- Participant is no longer actively participating in study activities
- Any clinical adverse event (AE), laboratory abnormality, or other medical condition or situation occurs such that continued participation in the study would not be in the best interest of the participant
- The participant meets an exclusion criterion (either newly developed or not previously recognized) that precludes further study participation
- The study sponsor, the principal investigator, or other body responsible for monitoring the safety of the study has decided to stop the study.

9.7.2 Handling of Participant Withdrawals or Termination

Subjects may withdraw at any time and for any reason by contacting the PI at the study site. If a subject chooses to withdraw from the study, or is otherwise terminated from the study, there will be an effort to obtain permission to record vital status data up to the protocol-described end of follow-up for that subject. Subjects who withdraw will be asked to provide a written communication via mail or email to the study site with their preferences about future contact and use of collected data and biospecimens. Upon withdrawal and receipt of written request from the participant, a subject's samples in storage at the Mayo Clinic Biorepository will be destroyed and collected study data will not be used in future research according to subject request. No future samples will be collected. After a short period of time to allow for validation of the withdrawal with the study site, the RECOVER Participant ID will be unlinked from fully identifying information. It will not be possible to retreivedata that have already been distributed. This includes the limited data set available in the RECOVER de-identified data analysis portal managed by the Data Resource Core at Massachusetts General Hospital.

An *End of Participation* form will be used to record cases of participant withdrawal, termination, or study discontinuance. The specifics of and study site expectations underlying each selection are detailed in the RECOVER CSC MOP.

There will be no planned replacement for study subjects lost to follow-up. However, enrollment targets may be adjusted based on planned interim analyses.

9.7.3 Premature Termination or Suspension of Study

This study may be temporarily suspended or prematurely terminated if there is sufficient reasonable cause. Written notification, documenting the reason for study suspension or termination, will be provided by the suspending or terminating party to the NIH Sponsor and site investigators. If the study is prematurely terminated or suspended, the site PI will promptly inform the IRB and will provide the reason(s) for the termination or suspension.
Circumstances that may warrant termination or suspension include, but are not limited to:

- Determination of unexpected, significant, or unacceptable risk to participants
- Emergence of new information from external sources that merits premature termination of the study
- Determination of futility for achievement of study aims that would warrant premature termination
- Insufficient compliance to protocol requirements
- Data that are not sufficiently complete and/or evaluable

Study may resume once concerns about safety, protocol compliance, and data quality are addressed and satisfy the sponsor and/or IRB.

10 Study Schedule

10.1 Overview of study schedule

A tiered phenotyping approach will be implemented for structured data collection at all study visits. The tiered phenotyping approach will be tailored across the child/young adult lifespan: early childhood including infancy,

toddlerhood and pre-school age (ages newborn-5 years), middle childhood (ages 6-11 years), adolescence (ages 12-17 years) and young adulthood (ages 18-25 years). All participants will provide informed consent (and assent) at the first Tier 1 study contact before participating in any study procedures.

Tier 1 procedures will include baseline questionnaires for collection of sociodemographic information, social determinants of health, medical history and core data elements related to past SARS-CoV-2 infection, possible PASC symptoms, and quality of life that will be collected remotely at study entry in all participants with or without history of SARS-CoV-2 infection (both caregiver and child/young adult subjects). Tier 1 will also include home collection of limited blood biospecimens including dried blood spot for detection of antibodies to SARS-CoV-2, and for storage in the PASC Consortium RECOVER biorepository for future analyses, and home collection of saliva for extraction of DNA for future analyses. If the primary caregiver is a biological parent of the child or young adult, the other biological parent, if available, may also be enrolled to provide a home sample of saliva for DNA analysis.

Tier 2 and Tier 3 procedures will include longitudinal questionnaire data and biospecimen collection conducted remotely and at on-site study visits to provide more detailed longitudinal characterization of PASC for up to four years in subjects with a history of SARS-CoV-2 infection, a matched sample of subjects without history of SARS-CoV-2 infection, and caregivers. Common data elements, adapted across the child and young adult lifespan, will be used for all Tiers when available. For acute participants there is an acute phase of Tier 2 (first 8 weeks), and for all Tier 2 participants there is a post-acute phase extending from 2-6 months to 48 months after study entry.

The Tier numbering represents progressively increased detail and complexity in phenotyping procedures rather than a temporal relationship. The study visit time windows are designed to allow overlap in the timing of Tier 1-3 procedures depending on the whether the enrollment occurs at the time of an acute or post-acute COVID-19 infection, the individual sub-cohort, the individual participant history of COVID-19, and other logistics related to scheduling.

Collection of blood biospecimens in children and young adults will be conducted in accordance with Federal regulations (the lesser of 50 mLs or 3 mL/kg in an eight-week period and collection will not occur more frequently than 2 times per week). For this protocol, we will be conservatively drawing a maximum of 2mL/kg for pediatric participants ages 24 through <72 months. Clinical laboratory tests will be collected at each site and processed locally or sent to a central laboratory for testing. Biospecimens for future analysis will be collected in collaboration with the PASC RECOVER Biorepository Core at the Mayo Clinic in Rochester Minnesota. For stored specimens, the biorepository staff will utilize test-specific standard operating procedures for biospecimen collection and transport, and will supply each site with necessary collection supplies for each study visit.

In addition to structured data collection, EHR data from pre- and post-pandemic time intervals, and historical data from extant cohorts may be incorporated into the research record when feasible. A mobile health platform may be developed for remote unstructured data collection including sensor data from wearable devices, and remote collection of structured and unstructured participant reported outcomes. Mobile health data will not be collected until IRB approval of a future modified protocol is obtained. Biospecimens derived from clinical procedures may be collected when feasible, including cerebrospinal fluid, other body fluids, bronchoalveolar lavage specimens, procedural biopsies, and surgical pathology specimens. Identified clinical biospecimens will be tracked and either transferred from the cohort site to the PASC central RECOVER biorepository or linked by the study UUID to the site biorepository for future access.

Schedules of Events by sub-cohort and age are included in Appendix B. Research procedures listed in the Schedule of Events will occur based on the elapsed time from study entry except for the MIS-C cohort, for which the testing will occur based on elapsed time from their index hospitalization, and the cohort of infants born to mothers with SARS-CoV-2 infection during pregnancy, for which the testing will occur based on elapsed time from birth. For the main pediatric cohort post-acute visits will occur at 6 months, 12 months, 24 months, 36 months, and 48 months after study entry.

Scheduled procedures will be conducted at each visit according to the subject age at the time of the visit. The questionnaire format and data granularity vary across age groups, but the domains remain constant to allow

roll up of data for longitudinal comparison across age groups for each participant. For young adults who attain age >25 years after enrollment, participation in the study will continue with the procedures as described for the 18-25 years age group.

10.2 Enrollment/Baseline

The study design is a longitudinal observational cohort study (forming a meta-cohort of five distinct cohort types), which enrolls subjects in an ambidirectional time frame relative to the presence of a SARS-CoV-2 infection (or a control subject without known infection selected from the same time frame). The study meta-cohort figure (Figure 1) represents the ambidirectional time frames for the different components of the meta-cohort. Post-Acute Tier 1 subjects may be enrolled from extant research cohorts, extant clinical cohorts, children born to mothers with SARS-CoV-2 infection during pregnancy, and post-acute cohorts with history of past SARS-CoV-2 infection in extant post-acute research cohorts or clinical post-acute cohorts (Figure 2). All subjects will undergo the same Tier 1 study procedures as described below.

Acute participants are enrolled directly into Tier 2 but will be required to complete the same baseline assessment forms as required for participants enrolling into the Post-Acute Tier 1 arm. For acute participants, participants complete a brief baseline assessment at enrollment and then the majority of the baseline assessment forms are completed during the week 8 visit.

Subjects will be selected for post-acute Tier 2 study procedures and post-acute Tier 3 study procedures based on history of SARS-CoV-2 infection and post-acute symptoms consistent with the sequelae of SARS-CoV-2 infection (Figure 1 Overview of Study Procedures).

Enrollment/Baseline Visit (Post-Acute Tier 1 and Acute Tier 2)

- Obtain and document consent from participants on study IRB-approved informed consent form, and assent form if applicable
- Verify SARS-CoV-2 infection status based on history
- Verify and document that enrollment criteria are met
- Obtain identify information for generation of UUID
- Obtain information via interview and questionnaires as described for Tier 1 below.
- Collect biospecimens as described for Tier 1 in Section 11.14.

10.3 Intermediate Visits (Tiers 2 and 3)

- Intermediate visits will be planned to occur according to age and sub-cohort per the descriptions in Section 11 and the schedules of assessments in Appendix B. Study procedures will be conducted for post-acute Tier 2 and Tier 3 as described below.
- Subjects will be selected for participation in post-acute Tiers 2 based on their history of COVID exposure and PASC symptoms as determined in Tier 1 for post-acute participant and uninfected controls, and at the week 8 visit for acute Tier 2 participants. Subjects will be selected for participation in post-acute Tiers 2 and Tiers 3 to achieve the enrollment targets as summarized in Figure 1.
- Scheduling of intermediate visits will differ by sub-cohort as follows:
    - For main pediatric cohort, each subject will be asked participate in visits according to the elapsed time from study entry and continue to end of the funding period.
    - For infants born to mothers with and without SARS-CoV-2 infection, each subject will be asked to participate in visits according to their elapsed time from birth.
    - For MIS-C patients, each subject will be asked to participate in visits according to their elapsed time from their index hospitalization for MIS-C.
- The maximum number of visits for an acute cohort subject enrolled at the start of the funding period would be 9, including 4 visits in Acute Tier 2 (3 remote and 1 on-site) and 5 visits on-site Post-Acute Tier 2 as described below.
- Intermediate visits may be conducted on-site at the study center and remotely. There will be telephone contact with the subjects during the study window for intermediate visits to assist in the completion of remote questionnaires. Subjects will receive email links for remote online completion of

required study questionnaires. Any email message with PHI will be encrypted prior to sending. Patient may opt-in to receive text communications with study staff.

- In the case of newly identified SARS-CoV-2 infection or re-infection after study entry (newly positive antigen test result >90 days after a known prior acute infection or newly positive antibody test result), additional intermediate visits will be scheduled as follows:
  - For identified acute infection or re-infection with SARS-CoV-2 (≤30 days), the participant will undergo 2, 4, and 8-week remote survey assessments according to Tier 2 acute procedures for participant age. Subsequent post-acute Tier 2 visits will be conducted without change in schedule. Participants initially enrolled as uninfected controls will be reclassified as part of the infected cohort.
  - For identified post-acute infection or re-infection (>30 days), one additional remote assessment according to Tier 2 post-acute procedures for participant age will be scheduled if the infection becomes known outside of a scheduled study visit window. Remote post-acute Tier 2 visit procedures will be conducted according to participant age. Subsequent post-acute Tier 2 visits will be conducted without change in schedule. Participants initially enrolled as uninfected controls will be reclassified as part of the infected cohort.
- Tier 2 and Tier 3 visits may be scheduled contemporaneously or separately according to the specified visit time windows per discretion of the site investigators, and participant preference.

10.4 Final Study Visit

The final study visit will occur 30 days before the end of the funding period according to the schedule of events in Appendix B. Any ongoing AE/SAE at the time of the final visit will be followed for an additional 30 days or until resolution, whichever is shorter. Subjects will be provided with instructions for further follow up of ongoing adverse events, sharing of study results, and for continuation of care with their primary care physician. Aggregate study results will be shared with participants within 6 months of the final study visit.

10.5 Withdrawal Visit

In the event that a subject withdraws early, or has study participation terminated by the investigator, an effort will be made to schedule a final study visit according to the next scheduled visit as listed in appendix B. The study procedures in the final visit may be abbreviated depending on subject preference and safety considerations.

10.6 Unscheduled Visit

In the event of an unanticipated adverse event as described in Section 12, or occurrence of a SAE, subjects may be contacted for an unscheduled remote or onsite visit in order to obtain necessary information for monitoring subject safety and reporting to the IRB and OSMB.

11 Study Procedures/Evaluations

11.1 Tiered phenotyping by sub-cohorts

Tiered phenotyping is customized for each of the sub-cohorts contributing to the RECOVER meta-cohort structure. Within each cohort, study procedures are customized based on age of the subject. Subjects participation over time will progress from Post-Acute Tier 1 or Acute Tier 2 to Post-Acute Tier 2 to Post-Acute Tier 3 according to sub-cohort criteria within study time windows, but participation in Post-Acute Tier 1 or Acute Tier 2 does not obligate participation in Post-Acute Tier 2, and participation in Post-Acute Tier 2 does not obligate participation in Post-Acute Tier 3. Details of the tiered phenotyping procedures are provided by sub-cohorts in the following paragraphs.

11.2 Main Cohort Module (n=6000 infected and uninfected subjects, ages newborn-25 years)

The following sections describe procedures for the different tiers for the main cohort.

11.3 Main Cohort Module Tier 1 Procedures

Post-Acute Tier 1 assessments will be conducted in participants selected from unbiased sources to determine history of COVID-19 infection and history or persistent symptoms post-COVID in order to estimate the incidence rate ratio of PASC. Tier 1 visits will be conducted remotely by caregiver/young adult self-report (electronic-based, phone-based, or paper-based where access to eCRFs may be limited) or by research staff-assisted data collection (telephone, videoconference) at the time of study entry. Tier 1 questionnaires are adapted according to subject age (newborn-5 years, 6-11 years, 12-17 years, and 18-25 years).

11.3.1 Main Cohort Tier 1 procedures ages newborn-5 years (not including congenital exposure cohort)

For subjects ages newborn to 5 years, the caregiver will be the primary respondent with limited input from the child. Tier 1 data collection may be conducted remotely and/or at an on-site study visit according to participant preference. It is anticipated that completion of all Tier 1 questionnaires will require approximately 2 hours. All Tier 1 assessments including the initial biospecimen collection will be completed within 3 months.

If Tier 1 enrollment occurs at time of acute COVID-19 or the patient is too ill to participate in the complete baseline assessment, patient-reported elements may be deferred until recovery.

Structured data collection will include the following domains:

- Obtain and document consent from participant on study IRB-approved informed consent form
- Child sociodemographic data and contact information including next of kin
- Child medical history, including birth history (including birth weight and length, gestational age, delivery type, problems during pregnancy), current health history (including current self-reported weight and height, disabilities, smoke exposure), special health care needs, and history of MIS-C, POTS and Long COVID.
- Child vaccination history
- Child health status
  - Early Childhood PROMIS Pediatric Global Health measure
- Child history of acute SARS-CoV-2 infection
- Child history of PASC symptoms
- COVID family infection history
- Child COVID health consequences (including child weight, diet, physical activity, screen time, sleep, educational services, parenting (e.g., discipline, parent-child relationship quality, cognitive stimulation [StimQ]).
- Household and child social determinants of health
  - Housing and address stability
  - financial stability
  - food insecurity
  - access to health care
  - caregiver health literacy
  - perceived discrimination (Everyday Discrimination Scale)
  - other childhood stressors
  - COVID guidelines (mask wearing, social distancing)
  - community cohesion (Neighborhood Collective Efficacy Questionnaire) and neighborhood factors

The Tier 1 study visit(s) will also include remote biospecimen collection (ages 24 months through 5 years) as described in Section 11.14. In cases of acute COVID-19 *after* enrollment, Tier 1 remote biospecimen collection will be delayed 30 days following onset of infection. Selected Tier 1 child participants without history of SARS-CoV-2 infection who reside in regions with higher rates of SARS-CoV-2 infection and who have no

detectable antibodies on the first test may contribute up to two additional biospecimens for SARS-CoV-2 antibody at 3-month intervals if the test remains negative (total 3 tests over total of 6 months).

Unstructured data collection from the participant EHR may also be recorded for Tier 1 assessments when feasible.

11.3.2 Main Cohort Tier 1 procedures ages 6-17 years

For subjects aged 6 to 17 years, the caregiver will be the primary respondent with optional input from the child. Tier 1 data collection may be conducted entirely remotely and may also occur at an on-site study visit according to participant preference. It is anticipated that completion of all Tier 1 assessments will require approximately 2 hours. All Tier 1 assessments including biospecimen collection will be completed within 3 months.

If Tier 1 enrollment occurs at time of acute COVID-19 or the patient is too ill to participate in the complete baseline assessment, patient-reported elements may be deferred until recovery .

Structured data collection will include the following domains:

- Obtain and document consent from participant on study IRB-approved informed consent form, and assent form if applicable
- Child sociodemographic data and contact information including next of kin
- Child medical history, including birth history (including birth weight and length, gestational age, delivery type, problems during pregnancy), current health history (including current self-reported weight and height, disabilities, smoke exposure), special health care needs, and history of MIS-C, POTS and Long COVID.
- Child vaccination history
- Child health status
    - Child/Young Adult PROMIS Pediatric Global Health measure
- Child history of acute SARS-CoV-2 infection
- Child history of PASC symptoms
- Child COVID health consequences (including child weight, diet, physical activity, screen time, sleep, educational services, parenting (e.g., discipline, parent-child relationship quality, cognitive stimulation [StimQ]).
- Household social determinants of health
    - Housing and address stability
    - financial stability
    - food insecurity
    - access to health care
    - perceived discrimination (Everyday Discrimination Scale)
    - other childhood stressors
    - COVID guidelines (mask wearing, social distancing)
    - community cohesion (Neighborhood Collective Efficacy Questionnaire) and neighborhood factors
    - DSM-5 cross-cutting measure (assessment of inattention, depression, anger, mania, anxiety, psychosis, repetitive thoughts and behaviors)

The tier 1 study visit(s) will also include remote biospecimen collection as described in Section 11.14. In cases of acute COVID-19 *after* enrollment, Tier 1 remote biospecimen collection will be delayed 30 days following onset of infection. Selected Tier 1 child participants without history of SARS-CoV-2 infection who reside in regions with higher rates of SARS-CoV-2 infection and who have no detectable antibodies on the first test may contribute up to two additional biospecimens for SARS-CoV-2 antibody at 3-month intervals if the test remains negative (total 3 tests).

Unstructured data collection from the participant EHR may also be recorded for Tier 1 assessments when feasible.

11.3.3 Main Cohort Tier 1 procedures ages 18-25 years

For subjects ages 18-25 years, the young adult will be the primary respondent with optional input from the caregiver. Tier 1 data collection may be conducted entirely remotely and may also occur at an on-site study visit according to participant preference. It is anticipated that completion of all Tier 1 assessments will require approximately 2 hours. All Tier 1 assessments including biospecimen collection will be completed within 3 months.

If Tier 1 enrollment occurs at time of acute COVID-19 or the patient is too ill to participate in the complete baseline assessment, patient-reported elements may be deferred until recovery .

Structured data collection will include the following domains:

- Obtain and document consent from participant on study IRB-approved informed consent form
- Young adult sociodemographic data and contact information including next of kin
- Young adult medical history, (including birth weight and length, gestational age, delivery type, problems during pregnancy), current health history (including current self-reported weight and height, disabilities, smoke exposure), special health care needs, and history of MIS-C, POTS and Long COVID.
- Young adult vaccination history
- Young adult health status
    - Young adult PROMIS10 Quality of Life
- Young adult history of acute SARS-CoV-2 infection
- Young adult history of PASC symptoms
- Young adult COVID health consequences (including weight, diet, physical activity, screen time, sleep, educational services).
- Household social determinants of health
    - Housing and address stability
    - financial stability
    - food insecurity
    - access to health care
    - perceived discrimination (Everyday Discrimination Scale)
    - stress (Perceived Stress Scale)
    - social support (RAND-MOS)
    - community cohesion (Neighborhood Collective Efficacy Questionnaire)
      DSM-5 cross-cutting measure (assessment of depression, anger, mania, anxiety, psychosis, repetitive thoughts and behaviors, dissociation, personality functioning)

The tier 1 study visit(s) will also include remote biospecimen collection a as described in Section 11.14. In cases of acute COVID-19 *after* enrollment, Tier 1 remote biospecimen collection will be delayed 30 days following onset of infection. Selected Tier 1 young adult participants without history of SARS-CoV-2 infection who reside in regions with higher rates of SARS-CoV-2 infection and who have no detectable antibodies on the first test may contribute up to two additional biospecimens for SARS-CoV-2 antibody at 3-month intervals if the test remains negative (total 3 tests).

Unstructured data collection from the participant EHR may also be recorded for Tier 1 assessments when feasible.

11.4 Main pediatric cohort acute Tier 2 assessments

Acute Tier 2 assessments will be conducted in participants with acute SARS-CoV-2 infection (≤30 days), and a sample of uninfected control subjects selected contemporaneously from same recruitment pool. The acute Tier 2 assessments will be conducted remotely when feasible. Questionnaires are customized for age groups

with primary respondent determined by the age of the child/young adult as described for Tier 1. All acute Tier 2 clinical assessments are optional based on the severity of illness, age of the participant and the judgment of the investigator and caregiver. Minimum ages are provided for each procedure. The schedule of assessments for all acute Tier 2 visits will be based on the elapsed time from study entry. Tier 1 biospecimens in acute COVID-19 patients will be collected at the 8-week visit of acute Tier 2.

11.4.1 Main Cohort Acute Tier 2 procedures ages Newborn-5 years

For infants, toddlers, and pre-school age children with acute post-natal SARS-CoV-2 infection (≤30 days) and uninfected control subjects, Acute Tier 2 assessments will occur at weeks 2, 4, and 8 after study entry. Questionnaires customized for age group newborn-5 years with caregiver as primary respondent will be administered as described for Tier 1. For participants < 2 years old, all study visits will be conducted remotely. For participants aged 2 to 5 years, the visit at Week 8 will be conducted on-site at the enrolling site facility if possible and will include clinical assessments and biospecimen collection. If the patient is too ill to participate in the scheduled visits, efforts will be made to capture as much data as possible remotely or onsite when participant condition allows. Structured data collection at study visits during this 8-week period will include the following domains:

- Child Acute COVID testing information
- Child exposure information
- Child Health care resource utilization
- Child Acute COVID medications
- Child Acute COVID symptoms
- Child PASC symptoms
- Child anthropometry and vital signs including pulse oximetry (week 8, ages 2-5 years): Child blood pressure, heart rate, oxygen saturation, oral, axillary or skin temperature, height (length), weight, waist circumference and skin fold thickness will be measured as appropriate for age.
- Child electrocardiogram (Week 8): ages 3-5 years if feasible based on judgment of site investigator and caregiver, optional for ages <5 years. A 12-lead or 15-lead electrocardiogram will be performed with FDA-approved equipment. Self-sticking pediatric size electrodes will be placed on the extremities and chest for the electrocardiogram recording and will be removed after the recording. The electrocardiogram will be completed in approximately 5 minutes.
- Child Spirometry testing (Week 8): An FDA-approved hand-held spirometry system will be used to measure lung function for ages 5-25 years (optional for participants < 7 years per judgement of the site investigator and caregiver). Subjects will be asked to blow into a single-use tube to capture exhaled gases. Three exhalations will be assessed.

A pulse oximeter for home use may be shipped from the study site to acute Tier 2 subjects ages 3-5 years and uninfected control subjects when feasible. The caregiver and child participant will complete a diary or oxygen percent saturation and pulse rate with daily measurements for one week, weekly measurements for the next 3 weeks, and additional measurements at the time of reported symptoms of palpitations or shortness of breath. The diary will be returned to the site for REDCap data entry.

Child biospecimen collection during on-site study visit at week 8 after start of the SARS-CoV-2 infection as described in Section 11.14.

Unstructured data collection from the participant EHR and from mobile health applications may also be recorded to monitor participant health events and symptoms status during the acute Tier 2 follow-up when feasible. The mobile health applications will not be used until their use is approved by the IRB. If a subject is hospitalized during the acute phase of infection, the electronic health record may be abstracted to capture length of stay, type of hospital unit, use of supplemental oxygen and ventilatory support, presence of MIS-C, and COVID-19 treatments.

No caregiver data will be collected during the acute Tier 2 assessments.

11.4.2 Main Cohort Acute Tier 2 procedures ages 6-17 years

For participants with acute SARS-CoV-2 infection (≤30 days) and uninfected control subjects, Tier 2 assessments will occur at weeks 2, 4, and 8 after study entry. Weeks 2 and 4 will include remote questionnaire assessments. Questionnaires customized for age groups 6-11 and 12-17 years with caregiver as primary respondent will be administered as described for Tier 1. The visit at Week 8 will be conducted on-site at the enrolling site facility if possible and will include clinical assessments and biospecimen collection. If the patient is too ill to participate in the scheduled visits, efforts will be made to capture as much data as possible remotely or onsite when patient condition allows. Structured data collection at study visits during this 8-week period will include the following domains:

- Child Acute COVID testing information
- Child exposure information
- Child Health care resource utilization
- Child Acute COVID medications
- Child Acute COVID symptoms
- Child PASC symptoms
- Child anthropometry and vital signs including oximetry (Week 8): Child blood pressure, heart rate, oxygen saturation, oral, axillary or skin temperature, height (length), weight, waist circumference, and skin fold thickness will be measured as appropriate for age.
- Child electrocardiogram (Week 8): A 12-lead or 15-lead electrocardiogram will be performed with FDA-approved equipment. Self-sticking pediatric size electrodes will be placed on the extremities and chest for the electrocardiogram recording and will be removed after the recording. The electrocardiogram will be completed in approximately 5 minutes.
- Child Spirometry testing (Week 8): An FDA-approved hand-held spirometry system will be used to measure lung function for ages 5-25 years (optional for participants < 7 years per judgement of the site investigator and caregiver). Subjects will be asked to blow into a single-use tube to capture exhaled gases. Three exhalations will be assessed.

A pulse oximeter for home use may be shipped from the study site to acute Tier 2 subjects and uninfected control subjects when feasible. The caregiver and child participant will complete a diary or oxygen percent saturation and pulse rate with daily measurements for one week, weekly measurements for the next 3 weeks, and additional measurements at the time of reported symptoms of palpitations or shortness of breath. The diary will be returned to the site for REDCap data entry.

Child biospecimen collection will occur at an on-site study visit at week 8 after start of the SARS-CoV-2 infection as described in Section 11.14.

Unstructured data collection from the participant EHR and from mobile health applications may also be recorded to monitor participant health events and symptoms status during the acute Tier 2 follow-up when feasible. The mobile health applications will not be used until their use is approved by the IRB. If a subject is hospitalized during the acute phase of infection, the electronic health record will be abstracted to capture length of stay, type of hospital unit, use of supplemental oxygen and ventilatory support, presence of MIS-C, and COVID-19 treatments.

No caregiver data will be collected during the acute Tier 2 assessments.

11.4.3 Main Cohort Acute Tier 2 procedures ages 18-25 years

For participants with acute SARS-CoV-2 infection (≤30 days) and uninfected control subjects, Tier 2 assessments will occur at weeks 2, 4, and 8 after study entry. Weeks 2 and 4 will include remote questionnaire assessments. Questionnaires customized for age group 18-25 years with young adult as primary respondent will be administered as described for Tier 1. The visit at week 8 will be conducted on-site at the enrolling site facility if possible and will include clinical assessments and biospecimen collection. If the patient is too ill to participate in the scheduled visits, efforts will be made to capture as much data as possible remotely or onsite when patient condition allows. Structured data collection at study visits during this 8-week period will include the following domains:

- Young adult Acute COVID testing information
- Young adult exposure information

- Young adult Health care resource utilization
- Young adult Acute COVID medications
- Young adult Acute COVID symptoms
- Young adult PASC symptoms
- Young adult anthropometry and vital signs including oximetry (week 8): Blood pressure, heart rate, oxygen saturation, oral, axillary or skin temperature, height (length), weight, waist circumference, and skin fold thickness will be measured as appropriate for age.
- Young adult electrocardiogram (week 8): A 12-lead or 15-lead electrocardiogram will be performed with FDA-approved equipment. Self-sticking pediatric size electrodes will be placed on the extremities and chest for the electrocardiogram recording and will be removed after the recording. The electrocardiogram will be completed in approximately 5 minutes.
- Young adult Spirometry testing (week 8): An FDA-approved hand-held spirometry system will be used to measure lung function. Subjects will be asked to blow into a single-use tube to capture exhaled gases. Three exhalations will be assessed.

A pulse oximeter for home use may be shipped from the study site to acute participants and uninfected control participants when feasible following their enrollment. The young adult participant – with optional assistance from the caregiver – will complete a diary of oxygen percent saturation and pulse rate with daily measurements for one week, weekly measurements for the next 3 weeks, and additional measurements at the time of reported symptoms of palpitations or shortness of breath. The diary will be returned to the site for REDCap data entry at the week 8 visit.

Young adult biospecimen collection will be at on-site study visit at week 8 after start of the SARS-CoV-2 infection as described in Section 11.14.

Unstructured data collection from the participant EHR and from mobile health applications may also be recorded to monitor participant health events and symptoms status during the acute Tier 2 follow-up when feasible. The mobile health applications will not be used until their use is approved by the IRB. If a subject is hospitalized during the acute phase of infection, the electronic health record will be abstracted to capture length of stay, type of hospital unit, use of supplemental oxygen and ventilatory support, presence of MIS-C, and COVID-19 treatments.

No caregiver data will be collected during the acute Tier 2 assessments.

11.5 Main Cohort Post-Acute Tier 2 Assessments

Post-Acute Tier 2 assessments will be conducted in patients with post-acute SARS-CoV-2 infection (>30 days), and a sample of patients without history of SARS-CoV-2 infection who have completed Tier 1 assessments. The post-acute Tier 2 assessments will be conducted remotely when feasible. Questionnaires are customized for age groups with primary respondent determined by the age of the child/young adult as described for Tier 1. All clinical assessments are optional based on the age of the participant and the judgment of the investigator and caregiver. Minimum ages are provided for each procedure. The schedule of assessments for all Tier 2 visits will be based on the elapsed time from study entry, and wide study windows for Tier 2 visits will be implemented to accommodate child and caregiver needs and facilitate completion of all scheduled assessments. For the first post-acute Tier 2 visit, sites will target a completion window of 6 months post-enrollment with the ability to complete up to 9 months post-enrollment. Thus, it is expected that, relative to the Month 6 target, the first post-acute Tier 2 visit will be scheduled within - 4 to +3 months. Visits thereafter will have a target window of +/- 3 months relative to study time point (See Schedule of Assessments in 20.2).

11.5.1 Main Cohort Post-Acute Tier 2 assessments ages Newborn-5 years

For infants, toddlers and pre-school age children with post-acute post-natal SARS-CoV-2 infection (>30 days) and uninfected control subjects, Post-acute Tier 2 assessments will occur five times at 6 months, 12 months, 24 months, 36 months and 48 months after study entry. Questionnaires customized for age group Newborn-5 years with caregiver as primary respondent will be administered as described for Tier 1. Study procedures in this age group will be conducted remotely for ages newborn to less than 2 years and will be conducted using a combination of remote or on-site visits for ages 2-5 years.

Remote or on-site Assessments

- Child interim medical history
- Child interim vaccination history
- Child interim history of SARS-CoV-2 infection
- Ages and Stages Questionnaire child development): age-appropriate version for ages newborn through 5 years old
- Modified Checklist for Autism in Toddlers (screening test for autism): given for ages 18 through 24 months old
- Ages and Stages Questionnaire Social Emotional (child development): age-appropriate version for ages newborn through 17 months old.
- Child Behavior Checklist for Behavioral Problems (child development): given for ages 18 months through 5 years old
- Household social determinants of health **(sent from Caregiver project)**

On-site assessments

- Growth (weight, height, head circumference [<2 years], skinfolds; measured)
- Child vital signs, including pulse oximetry
- Child electrocardiogram (ages 3-5 years if feasible based on judgment of site investigator and caregiver; optional for ages <5 years; optional after first post-acute collection *within normal limits*; if abnormal, assessment may be repeated at subsequent visits at investigator's discretion)
- Child spirometry (ages 5 years and older if feasible based on judgment of site investigator and caregiver; optional in participants < 7 years old; optional after first post-acute collection *within normal limits*; if abnormal, assessment may be repeated at subsequent visits at investigator's discretion)
- Child Beighton Scale for joint flexibility. This scale assesses joint hypermobility for the fifth digits of the hand, thumbs, elbows, knees, and spine with simple maneuvers (ages 3-5 years, optional after first post-acute collection *within normal limits*; if abnormal, assessment may be repeated at subsequent visits at investigator's discretion)
- Child detailed cognitive development (ages 3-5 years)
    - NIH Toolbox: Picture Vocabulary Test: Language Development – Receptive
    - NIH Toolbox: Flanker Inhibitory Control and Attention Test: Executive Functions - Inhibition and Attention
    - NIH Toolbox: Dimensional Change Card Sort Test: Executive Functions - Flexibility and Switching of Cognitive Tasks
    - NIH Toolbox: Picture Sequence Memory Test: Visual Memory

- Child post-acute Tier 2 biospecimen collection for ages 24 months to <72 months (2-5 years) may be collected using a validated volume-by-weight calculation of 2 mL/kg or <5% total blood volume at 6, 12, 24, 36, and 48 months after study entry on-site at the pediatrics cohort study locations or by a home phlebotomy service as described in Section 11.14.

Unstructured data collection from the participant EHR and from mobile health applications may also be recorded to monitor participant health events and symptoms status during longitudinal post-acute Tier 2 follow-up when feasible. The mobile health applications will not be used until their use is approved by the IRB.

11.5.2 Main Cohort Post-Acute Tier 2 assessments ages 6-17 years

For children ages 6-17 years with post-acute SARS-CoV-2 infection (>30 days) and uninfected control subjects, post-acute Tier 2 assessments will occur five times at 6 months, 12 months, 24 months, 36 months and 48 months after study entry. Questionnaires customized for age groups 6-11 years and 12-17 years with caregiver as primary respondent will be administered as described for Tier 1. Study procedures in this age group will be conducted remotely and on-site with structured data collection at study visits:

Remote or on-site Assessments

- Child interim medical history
- Child interim vaccination history
- Child interim history of SARS-CoV-2 infection

- Child interim COVID health consequences
- Child interim history of PASC symptoms
- Child interim well-being
  - PROMIS Pediatric Global Health measure
  - PROMIS Fatigue
  - PROMIS Physical Activity
- Child diet
- Child autonomic dysfunction symptoms assessment (Composite Autonomic Symptom Score, COMPASS-31, optional after first successful post-acute collection)
- Child education status
  - Academic performance
  - Attendance
  - Grade level (months 12, 24, 36, and 48 only)
  - Accommodations
- Child detailed emotional/mental health development
  - PROMIS Positive Affect (Short form)
  - PROMIS Emotional Distress – Anger (Short form)
  - PROMIS Psychological Stress Experiences (Short form)
  - Revised Children's Anxiety and Depression Scale-25 (RCADS-25) – parent reported
  - Strengths and Difficulties Questionnaire (conduct and hyperactivity scales only)
- Household social determinants of health **(sent from Caregiver project)**

On-site Assessments

- Child anthropometry and vital signs, including pulse oximetry
- Active standing test: This test will include 10 minutes of standing with frequent heart rate and blood pressure measurements at the first post-acute visit as tolerated. If participant meets criteria for postural orthostatic tachycardia syndrome (POTS), assessment may be repeated at subsequent post-acute visits at site investigator’s discretion. We will have measurements at supine baseline, then 1, 3, 5, 7, and 10 minutes as tolerated standing (pulse rate, systolic and diastolic blood pressure). If a child needs to stop earlier than the 10 minutes, the reason will be documented.
- Child electrocardiogram (optional after first post-acute collection *within normal limits*; if abnormal, assessment may be repeated at subsequent visits per site investigator’s discretion)
- Child spirometry (optional for participants < 7 years per judgment of site investigator; optional after first post-acute collection *within normal limits*; if abnormal, assessment may be repeated at subsequent visits per site investigator’s discretion)
- Child Beighton Scale for joint flexibility. This scale assesses joint hypermobility for the fifth digits of the hand, thumbs, elbows, knees, and spine with simple maneuvers (optional after first post-acute collection *within normal limits*; if abnormal, assessment may be repeated at subsequent visits per site investigator’s discretion).
- Child detailed cognitive development
  - NIH Toolbox: Picture Vocabulary Test: Language Development – Receptive
  - NIH Toolbox: Flanker Inhibitory Control and Attention Test: Executive Functions - Inhibition and Attention
  - NIH Toolbox: List Sort Working Memory Test: Verbal Working Memory **(Ages 7 years and older)**
  - NIH Toolbox: Dimensional Change Card Sort Test: Executive Functions - Flexibility and Switching of Cognitive Tasks
  - NIH Toolbox: Pattern Comparison Processing Speed Test: Processing Speed **(Ages 7 years and older)**
  - NIH Toolbox: Picture Sequence Memory Test: Visual Memory
  - NIH Toolbox: Oral Reading Recognition Test: Reading Ability **(Ages 7 years and older)**
  - NIH Toolbox: Auditory Verbal Learning Test (Rey): Verbal Memory **(Ages 8 years and older)**

- Child Post-Acute Tier 2 biospecimen collection may be conducted at 6, 12, 24, 36, and 48 months after study entry on-site at the pediatrics cohort study locations or by home phlebotomy service as described in Section 11.14.

Unstructured data collection from the participant EHR and from mobile health applications may also be recorded to monitor participant health events and symptoms status during longitudinal post-acute Tier 2 follow-up when feasible. The mobile health applications will not be used until their use is approved by the IRB.

11.5.3 Main Cohort Post-Acute Tier 2 assessments ages 18-25 years

For young adults ages 18-25 years with post-acute SARS-CoV-2 infection (>30 days) and uninfected control subjects, Tier 2 assessments will occur five times at 6 months, 12 months, 24 months, 36 months and 48 months after study entry. Questionnaires customized for age 18-25 years with young adult as primary respondent will be administered as described for Tier 1. Study procedures in this age group will be conducted remotely and on-site with structured data collection at study visits:

Remote or on-site Assessments

- Young Adult interim medical history
- Young Adult interim vaccination history
- Young Adult interim history of SARS-CoV-2 infection
- Young Adult interim COVID health consequences
- Young Adult interim history of PASC symptoms
- Young Adult interim well-being
- PROMIS 10 Global Health measure
- PROMIS Fatigue
- PROMIS Physical Activity
- Young Adult diet
- Young Adult sleep
- Young Adult autonomic dysfunction symptoms assessment (Composite Autonomic Symptom Score, COMPASS-31 (optional after first successful post-acute collection)
- Young adult detailed emotional/mental health development
    - PROMIS Positive Affect (Short form)
    - PROMIS Emotional Distress – Anger (Short form)
    - PROMIS Perceived Stress
    - ASEBA-PC Adult Self-Report

On-site Assessments

- Young Adult anthropometry and vital signs including oximetry
- Active standing test: This test will include 10 minutes of standing with frequent heart rate and blood pressure measurements at the first post-acute visit as tolerated. If participant meets criteria for postural orthostatic tachycardia syndrome (POTS), assessment may be repeated at subsequent post-acute visits at site investigator's discretion. We will have measurements at supine baseline, then 1, 3, 5, 7, and 10 minutes as tolerated standing (pulse rate, systolic and diastolic blood pressure). If a young adult needs to stop earlier than 10 minutes, the reason will be documented.
- Young Adult electrocardiogram (optional after first post-acute collection *within normal limits*; if abnormal, assessment may be repeated at subsequent visits per site investigator's discretion)
- Young Adult spirometry (optional after first post-acute collection *within normal limits*; if abnormal, assessment may be repeated at subsequent visits per site investigator's discretion)
- Young Adult Beighton Scale for joint flexibility. This scale assesses joint hypermobility for the fifth digits of the hand, thumbs, elbows, knees, and spine with simple maneuvers (optional after first post-acute collection *within normal limits*; if abnormal, assessment may be repeated at subsequent visits per site investigator's discretion)
- Young adult detailed cognitive development
    - NIH Toolbox: Picture Vocabulary Test: Language Development – Receptive
    - NIH Toolbox: Flanker Inhibitory Control and Attention Test: Executive Functions - Inhibition and Attention
    - NIH Toolbox: List Sort Working Memory Test: Verbal Working Memory
    - NIH Toolbox: Dimensional Change Card Sort Test: Executive Functions - Flexibility and Switching of Cognitive Tasks

- NIH Toolbox: Pattern Comparison Processing Speed Test: Processing Speed
- NIH Toolbox: Picture Sequence Memory Test: Visual Memory
- NIH Toolbox: Oral Reading Recognition Test: Reading Ability
- NIH Toolbox: Auditory Verbal Learning Test (Rey): Verbal Memory

- Young Adult Post-Acute Tier 2 biospecimen collection may be conducted at 6, 12, 24, 36, and 48 months after study entry on-site at the pediatrics cohort study locations or by home phlebotomy service as described in Section 11.14.

Unstructured data collection from the participant EHR and from mobile health applications may also be recorded to monitor participant health events and symptoms status during longitudinal post-acute Tier 2 follow-up when feasible. The mobile health applications will not be used until their use is approved by the IRB.

11.5.4 Monitoring Plan for Mental Health Responses

A detailed SOP for how the RECOVER CSC will direct sites to engage with participants around concerning responses to well-being questions across ages and assessments is contained in the CSC MOP ("Safety Monitoring of Mental Health Questionnaires").

11.6 Main Pediatric Cohort Post-Acute Tier 3 assessments

For participants with history of SARS-CoV-2 infection with or without PASC symptoms, all of the listed Post-Acute Tier 3 procedures will be performed if appropriate for age and there is no contraindication to a specific procedure.

All Tier 3 testing is optional based on the age of the participant and the judgment of the investigator and caregiver. Tier 3 visits will be conducted twice. For Main Cohort participants first visit will occur 12-30 months after study entry and second about one year later, 24-42 months after study entry.

Participants will be recruited to participate in Tier 3 testing based on sampling from Tier 2 participants to maintain diversity in the study population, and in all subjects with history of MIS-C. Wide study windows for Tier 3 will be implemented to accommodate child/young adult and caregiver needs and facilitate completion of all scheduled assessments. It is anticipated that completion of Tier 3 procedures might require up to 3 separate visits (total 12 hours) and may be prioritized based on symptoms reported by the participant. The schedule of assessments for all Tier 3 visits will be based on the elapsed time from study entry.

11.6.1 Main cohort Tier 3 assessments (ages 3-5 years)

For pre-school age participants with history of SARS-CoV-2 infection with or without PASC symptoms, a limited set of Tier 3 assessment may be performed. All Tier 3 testing is optional based on the age of the participant and the judgment of the investigator and caregiver. Tier 3 visits will be conducted twice, first at 12-30 months after study entry, and second about one year later, 24-42 months after study entry.

Structured data collection at Tier 3 study visits may include all or some of the following testing:

- Child Echocardiogram (ages 3-5 years). An echocardiogram will be performed according to standard clinical protocols and is optional for age <7 years per judgment of the site investigator and caregiver. Three gel electrodes will be placed on the torso to measure electrocardiogram. Ultrasound gel will be applied to the chest; a hand-held Doppler ultrasound transducer will be applied by a licensed pediatric ultrasound technician to the chest wall to obtain standard images of the cardiac chambers, cardiac valves, Doppler-derived blood flow velocities, and global longitudinal strain. The echocardiogram requires 60 minutes.
- Child Abdominal ultrasound (ages 3-5 years). An abdominal ultrasound examination will be performed by a trained technician according to clinical protocols and is optional for age <7 years per judgment of the site PI and caregiver). The liver, pancreas, kidneys, and bladder will be imaged. Total time required is 60 minutes.
- Awake EEG (ages 3-5 years): An electroencephalogram (EEG) will be performed according to clinical protocols with FDA approved equipment and is optional for age <9 years per judgement of the site

investigator and caregiver. A trained technician will apply scalp electrodes and record the EEG for up to 60 minutes.

- Child neurocognitive testing. These tests will be conducted by a Child Psychologist or their trained assistant. The main set of measures for the neurocognitive battery come from the Woodcock-Johnson batteries, which is a battery of measures with strong psychometric properties, that can be used with subjects between the ages of 3-5 years (valid for wide age range of 2 to 90 years old). These will include Cognitive measures, Language measures, Achievement measures, and behavioral and psychiatric testing. There are Spanish versions of parts of the Cognitive and Achievement subtests. There are norms developed based upon a large nationally representative sample.

Table 2: Tier 3 Neurocognitive Testing for Ages 3 to 5 years

Test category	Test
General Intelligence-Verbal	WJ-IV Oral Language
General Intelligence-Nonverbal	WJ-IV Spatial Reasoning
Executive Function - Attention	NIMH Toolbox - Flanker Test
Executive Function - Inhibitory Control	NIMH Toolbox - Inhibitory Control
Episodic Memory	NIMH Toolbox - Picture Sequence Memory
Language - Receptive	WJ-IV - Understanding Directions
Language - Expressive	WJ-IV - Picture Vocabulary & Rapid Picture Naming
Working Memory - Verbal	WJ-IV Numbers Reversed
Processing Speed	WJ-IV - Pair Cancellation
Verbal Memory	WJ-IV Story Memory
Visual-Motor Integration	Beery Buktenica Test
Motor Speed	Purdue Pegboard
Reading	WJ-IV - Letter Word Identification
Spelling	WJ-IV - Spelling
Mathematics	WJ-IV - Number Sense and Calculation
Emotional and Behavioral Adjustment	CBCL, Strengths and Difficulties Questionnaire
Psychiatric Disorder	K-CAT

- Microbiome biospecimen collection (ages 3-5 years). Collection of skin swabs, nasal swabs, oral swabs, urine and stool will be performed and is optional for age <7 years per judgment of site investigator and caregiver. Soft-tipped, sterile cotton swabs will be used for the sample collection. Microbiome swab collection will require 15 minutes. Stool will be collected at home with remote collection kits mailed to the participant home.

Child Tier 3 biospecimen collection (<15 ml) will be conducted on-site at the pediatrics cohort study locations or by home phlebotomy service as described in Section 11.14.

11.6.2 Main cohort Post-Acute Tier 3 assessments (ages 6-25 years)

For child/young adult participants with history of SARS-CoV-2 infection with or without PASC symptoms, a panel of Tier 3 assessment may be performed. All Tier 3 testing is optional based on the age of the participant and the judgment of the investigator and caregiver. Tier 3 visits will be conducted twice. For Main Cohort participants first visit will occur 12-30 months after study entry and second about one year later, 24-42 months after study entry. Structured data collection at Tier 3 study visits may include all or some of the following testing:

- Echocardiogram (ages 6-25 years). An echocardiogram will be performed according to standard clinical protocols and is optional for age <7 years per judgment of the site investigator and caregiver. Three gel electrodes will be placed on the torso to measure electrocardiogram. Ultrasound gel will be applied to the chest; a hand-held Doppler ultrasound transducer will be applied by a licensed pediatric ultrasound technician to the chest wall to obtain standard images of the cardiac chambers, cardiac valves, Doppler-derived blood flow velocities, and global longitudinal strain. The echocardiogram requires 60 minutes.
- Child/Young Adult cardiac MRI (ages 6-25 years). A cardiac MRI will be performed according to standard clinical protocols in children who do not require sedation and is optional for age <9 years per judgment of site investigator and caregiver. Children with metallic implants will not be permitted to

participate. The MRI will be conducted by trained technicians under supervision of licensed pediatric cardiologists and/or radiologists at each study site. The MRI requires 60 minutes.

- Child/Young Adult pulmonary function tests including diffusing capacity (ages 7-25 years). Pulmonary function testing will be completed according to clinical protocols and is optional for age <9 years per judgment of the site investigator and caregiver. Participants will be asked to breath in prescribed patterns to complete the measurements of lung capacity and gas diffusion. Pulmonary function tests require 60 minutes.
- Child/Young Adult sputum induction (10-25 years). A standard of care clinical protocol for sputum induction will be used and is optional for children <12 years per judgment of the site investigator and caregiver. Participant will inhale nebulized hypertonic saline solution (3%) for 5-15 minutes. The saline mist liquefies airway secretions, promotes coughing and allows expectoration of respiratory secretions. Subjects with history of bronchospasm will be excluded. To minimize risk of bronchospasm, children with asthma will be excluded and all children will receive pre-treatment with a bronchodilator (levalbuterol). Levalbuterol is an analog of endogenous catecholamines with established safety profile in studies of >600 children (31). Oxygen saturation will be monitored throughout the procedure. A handheld spirometer will be used to monitor Forced Expiratory Volume at 1 second (FEV1.0) during the procedure at 5-minute intervals. The procedure will be stopped if the FEV1.0 decreased >20% from pre-testing baseline or if the subject has symptoms of shortness of breath. Sputum induction will be performed by an experienced technician under the supervision of a licensed medical professional. Bronchodilator rescue therapy will be readily available if needed.
- Child/Young Adult symptom-limited cardiopulmonary exercise testing (ages 10-25 years). Participants will perform symptom-limited graded exercise on a treadmill or stationary bicycle ergometer according to clinical protocols in children and is optional for children <12 years per judgment of local site investigator and caregiver. Trained technicians under supervision of licensed pediatricians will oversee the test. Pediatric size gel electrodes will be placed to measure the electrocardiogram before, during and after exercise. A mouth tube or facemask will be used to collect expired gases before during and after exercise. Blood pressure will be measured at 1-3 minutes intervals before during and at 2-minute intervals for up to 10 minutes post-exercise. Total time required is 60 minutes.
- Child/Young Adult abdominal ultrasound (ages 6-25 years). An abdominal ultrasound examination will be performed by a trained technician according to clinical protocols and is optional for age <7 years per judgment of the site PI and caregiver). The liver, pancreas, kidneys, and bladder will be imaged. Total time required is 60 minutes.
- Child/Young Adult brain MRI (ages 6-25 years): A 3T or lower field strength brain MRI will be performed according to standard clinical protocols in children who do not require sedation and is optional for age <9 years per judgment of the site investigator and caregiver. The MRI will be conducted by trained technicians under supervision of licensed radiologists at each study site. The MRI requires up to 60 minutes.
- Awake EEG (ages 6-25 years): An electroencephalogram (EEG) will be performed according to clinical protocols with FDA approved equipment and is optional for age <9 years per judgement of the site investigator and caregiver. A trained technician will apply scalp electrodes and record the EEG for up to 60 minutes.
- Child/Young adult neurocognitive testing. These tests will be conducted by a Child Psychologist or their trained assistant. The main set of measures for the neurocognitive battery come from the Woodcock-Johnson batteries, which is a battery of measures with strong psychometric properties, that can be used with subjects between the ages of 6 to 25 years (valid for wide age range of 2 to 90 years old). These will include Cognitive measures, Language measures, Achievement measures and behavioral and psychiatric testing. There are Spanish versions of parts of the Cognitive and Achievement subtests. There are norms developed based upon a large nationally representative sample.

Table 3: Tier 3 Neurocognitive Testing for 6 to 25 Year Olds

**Test category**	**Test**
General Intelligence-Verbal	WJ-IV Oral Language
General Intelligence-Nonverbal	WJ-IV Spatial Reasoning
Executive Function - Attention	NIMH Toolbox - Flanker Test
Executive Function - Inhibitory Control	NIMH Toolbox - Inhibitory Control

Episodic Memory	NIMH Toolbox - Picture Sequence Memory
Language - Receptive	WJ-IV - Understanding Directions
Language - Expressive	WJ-IV - Picture Vocabulary & Rapid Picture Naming
Working Memory - Verbal	WJ-IV Numbers Reversed
Working Memory - Visual	WRAML2 - Picture Memory (5 to 18)
Processing Speed	WJ-IV - Pair Cancellation
Verbal Memory	WJ-IV Story Memory
Visual-Motor Integration	Beery Buktenica Test
Motor Speed	Purdue Pegboard
Reading	WJ-IV - Letter Word Identification
Spelling	WJ-IV - Spelling Y
Mathematics	WJ-IV - Number Sense and Calculation
Emotional and Behavioral Adjustment	Strengths and Difficulties Questionnaire
Psychiatric Disorder	K-CAT

- Microbiome biospecimen collection (ages 6-25 years). Collection of skin swabs, nasal swabs, oral swabs, urine and stool will be performed and is optional for age <7 years per judgment of site investigator and caregiver. Soft-tipped, sterile cotton swabs will be used for the sample collection. Microbiome swab collection will require 15 minutes. Stool will be collected at home with remote collection kits mailed to the participant home.

Child Tier 3 biospecimen collection will be conducted on-site at the pediatrics cohort study locations or by home phlebotomy service as described in Section 11.14.

11.7 MIS-C Cohort (n=800, ages 3-25 years)

Since the participants in the MIS-C cohort have existing data collected from participation in other NIH funded research, a limited set of Tier 1 procedures will be performed to avoid duplication with existing data. Visits will be scheduled according to time elapsed from the index date of hospitalization for MIS-C. The initial visit will be scheduled 3-30 months after the index hospitalization. The second study visit will be scheduled approximately one year later 15-42 months after the index hospitalization. Due to participant burden related to other studies, caregivers will not be enrolled but will be the primary respondents for questionnaires for subjects ages 3-17 years. Tier 1 questionnaires are adapted according to subject age (3-5 years, 6-17 years, and 18-25 years). For subjects ages 3-17 years, the caregiver will be the primary respondent with optional input from children ages 12-17 years. For subjects ages 18-25 years, the young adult will be the primary respondent with optional input from the caregiver.

11.7.1 MIS-C cohort Tier 1 remote assessments

- Sociodemographics and history of MIS-C extracted from research records or health records
- Household social determinants of health
    - address stability
    - financial stability
    - food insecurity
    - access to health care
    - perceived discrimination (Everyday Discrimination Scale)
    - stress (Perceived Stress Scale)
    - social support (RAND-MOS)
    - community cohesion (Neighborhood Collective Efficacy Questionnaire)
- Child/Young adult COVID health impact
- Child/Young adult history of PASC symptoms
- Child PROMIS Pediatric Global Health measure or young adult PROMIS10 Quality of Life

11.7.2 MIS-C cohort Tier 2 visits

Participants with MIS-C will undergo a subset of post-acute Tier 2 assessments at study entry and annually for two additional years:

Remote or on-site Assessments

- Child/Young Adult history of interim medical history, interim SARS-CoV-2 infection and vaccination history
- Child/Young Adult COVID health consequences
- Child/Young Adult interim history of PASC symptoms
- Child PROMIS Pediatric Global Health measure or young adult PROMIS10 Quality of Life
    - PROMIS- Physical Activity
    - PROMIS fatigue
- Young Adult autonomic dysfunction symptoms assessment (Composite Autonomic Symptom Score, COMPASS-31, ages 6-25 years, optional after the first post-acute visit)

Extant cohort data will be harmonized with RECOVER data collection when possible. Unstructured data collection from the participant EHR will also be recorded for Tier 2 assessments when feasible.

There is no electrocardiogram procedure, spirometry procedure, or blood biospecimen collection in the MIS-C cohort.

11.7.3 MIS-C cohort Tier 3 visits

MIS-C Tier 3 visits will be limited to selection of up to three of the Tier 3 procedures listed for the main cohort. The procedures will be selected based on age, past clinical manifestations and clinical testing for each subject. The procedures will be conducted according to the same procedures as listed for the main pediatric cohort (two Tier 3 visits approximately 1 year apart). Tier 3 procedures already conducted as part of the COVID MUSIC study (e.g., Echo, Cardiac MRI), may be used in the RECOVER study as a Tier 3 measure.

11.8 Infants born to mothers with and without history of SARS-CoV-2 during pregnancy (n=2500, ages newborn-48 months)

The congenitally-exposed cohort performs only a limited portion of assessments within the RECOVER pediatric protocol. These study procedures are outlined in sections 11.9.1, 11.9.2, and Appendix B table 13. This cohort completes no other measures beyond those outlined within those sections. All included assessments are considered minimal risk.

Tier 1 and Tier 2 procedures for this cohort listed below will be conducted at 12, 18, 24, 36, and 48 months after birth. There are no Tier 3 procedures. Tier 1 for this cohort refers to assessments that are done remotely. This is different from the main cohort Tier 1 in which Tier 1 is only the baseline assessment. Tier 2 for this cohort refers to in-person or video-conferencing assessments.

At enrollment, participants' baseline visit will comprise of remote surveys and age-specific measures for the appropriate visit timepoint (12, 18, 24, 36, and 48 months). These age-specific measures will populate in REDCap when applicable at the Baseline visit.

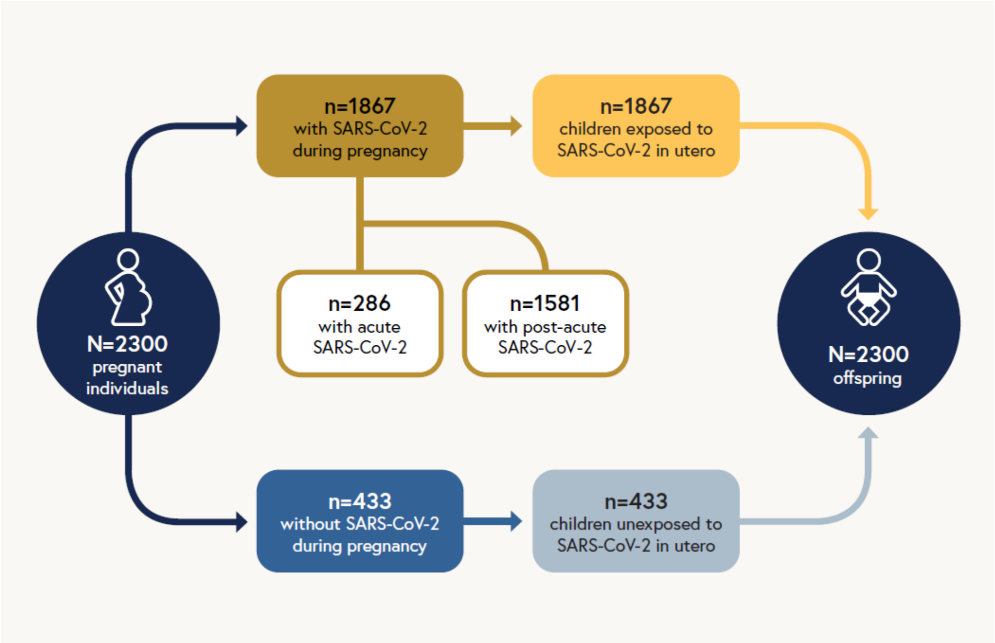

Figure 3: Congenitally-Exposed Cohort Enrollment Breakdown

11.8.1 Infant cohort Tier 1 assessments (remote)

- Child birth history
- National Survey of Children's Health Questionnaire
- Sociodemographics
- Health status
- Special health care needs
- PROMIS global health
- COVID and vaccine history
- COVID health consequences [including child weight, diet, physical activity, screen time, sleep, educational services, parenting (e.g., discipline, parent-child relationship quality, cognitive stimulation [StimQ])]
- Social determinants of health (child health)
- Growth at 12 and 18 months (weight, length, head circumference [<2 years]; self-report or EHR)
- Infant sleep at 24 months (Brief Infant Sleep Questionnaire)
- Child Developmental Assessments
    - Ages and Stages Questionnaire at 12, 24 and 36 months
    - Modified Checklist for Autism in Toddlers at 18 months (screening test for autism)
    - Ages and Stages Questionnaire Social Emotional at 18 months
    - Child Behavior Checklist for Behavioral Problems at 24 and 36 months
    - Developmental Profile-4 Time at 36 months

11.8.2 Infants cohort Tier 2 assessments (on-site or remote)

- Growth at 24, 36 and 48 months (weight, height, head circumference [<2 years], skinfolds)
- Bayley Scales of Infant Development-4 at 24 months (child development)
- Differential Ability Scales-II, Time at 36 and 48 months (child development)

Tier 2 biospecimens will be collected at age 24 months using an at-home collection device (Tasso M-20) with <5mL collected or by venipuncture using a validated volume-by-weight calculation of no more than 2 mL/kg for <5% total blood volume as described in Section 11.14.

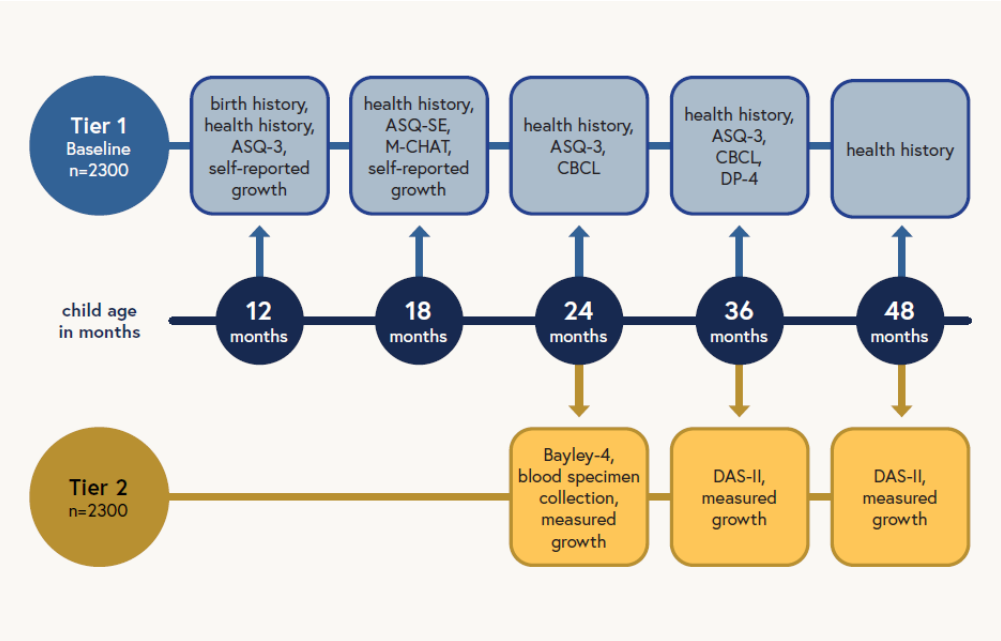

Figure 4: Congenitally-Exposed Cohort Assessments

11.9 ABCD cohort (n=10,000, ages 12-17 years)

11.9.1 ABCD Tier 1 assessments

The RECOVER procedures are harmonized to avoid duplication with other study data collection in the ABCD study. Due to participant burden related to the ABCD study, only Post-Acute Tier 1 assessments will be performed. For ABCD subjects who have evidence of possible PASC symptoms, referral to other RECOVER sites for participation in Tier 2 and Tier 3 assessments will be offered as an option, if geographically feasible.

Tier 1 visits will be conducted remotely by caregiver/child self-report (electronic-based, phone-based, or paper-based) or by research staff-assisted data collection (telephone, videoconference) at the time of study entry. Tier 1 questionnaires are adapted according to participant age for this cohort (12-17 years). For subjects ages 12-17 years, the caregiver will be the primary respondent with optional input from children ages 12-17 years.

- Child and caregiver sociodemographic data and contact information including next of kin
- Child and caregiver medical history, including birth history (including birth weight and length, gestational age, delivery type, problems during pregnancy), current health history (including current self-reported weight and height, disabilities, smoke exposure), special health care needs, and history of MIS-C, POTS and Long COVID.
- Child and caregiver vaccination history
- Child and caregiver health status
    - Caregiver PROMIS 10 Quality of Life
    - Child/Young Adult PROMIS Pediatric Global Health measure
- Child and caregiver history of acute SARS-CoV-2 infection
- Child and caregiver history of PASC symptoms
- Child and caregiver COVID health consequences (including child weight, diet, physical activity, screen time, sleep, educational services).
- Household social determinants of health
    - Housing and address stability
    - financial stability
    - food insecurity
    - access to health care
    - perceived discrimination (Everyday Discrimination Scale)
    - other childhood stressors

- COVID guidelines (mask wearing, social distancing)
- community cohesion (Neighborhood Collective Efficacy Questionnaire) and neighborhood factors
- DSM-5 cross-cutting measure (assessment of inattention, depression, anger, mania, anxiety, psychosis, repetitive thoughts and behaviors)

The Tier 1 study visit(s) will also include remote biospecimen collection of blood (for the child and caregiver) and saliva (for the caregiver) as described in Section 11.14 (no child saliva collection for DNA as DNA data already exists in the ABCD biobank). Selected Tier 1 child and young adult participants without history of SARS-CoV-2 infection who reside in regions with higher rates of SARS-CoV-2 infection and who have no detectable antibodies on the first test may contribute up to two additional biospecimens for SARS-CoV-2 antibody at 3-month intervals if the test remains negative (total 3 tests).

11.10 Caregiver Assessments

Caregivers will be identified at the time of child/young adult enrollment as described in the study entry criteria for the main cohort and the ABCD cohort. Caregivers will not be enrolled for the MIS-C cohort or the infants of mothers with history of SARS-CoV-2 during pregnancy (since the mothers will be enrolled within the RECOVER adult cohort).

11.10.1 Caregiver Tier 1 Assessments

- Caregiver medical history
- Caregiver vaccination history
- Caregiver health status (PROMIS-10 Quality of Life)
- Caregiver history of acute SARS-CoV-2 infection
- Caregiver history of PASC symptoms
- Caregiver COVID health consequences (including weight, diet, physical activity, screen time, sleep).
- Caregiver social determinants of health (including employment history, health insurance, health care utilization, positive childhood experiences, perceived discrimination, social support (RAND-MOS),
- Caregiver wellbeing: depressive symptoms (patient health questionnaire-9); anxiety symptoms (generalized anxiety questionnaire-7); stress (perceived stress scale); DSM-5 cross-cutting measure (assessment of anger, mania, psychosis, repetitive thoughts and behaviors, dissociation, personality functioning)

Caregiver Tier 1 biospecimen collection will be conducted remotely as described in section 11.14.

11.10.2 Caregiver Tier 2 Assessments (remote time of Child/Young Adult visits at 12, 24, 36, and 48 months)

- Caregiver interim medical history
- Caregiver interim vaccination history
- Caregiver interim health status (PROMIS-10 Quality of Life)
- Caregiver interim history of acute SARS-CoV-2 infection
- Caregiver interim history of PASC symptoms
- Caregiver interim COVID health impact

11.11 Other Biological Parent Assessments

If the caregiver is a biological parent, the other biological parent, if available, may enroll in the study to provide a saliva specimen for biorepository storage and future genetic analyses. PII will be collected to generate a UUID, as required.

11.12 Mobile Health Products and Devices Data Collection

Commercial products or devices made by third party companies, including wearable fitness trackers, wearable sleep monitors, mobile apps for use on smartphone and tablets, websites and web apps, and types of computer software that permit screen sharing, record keystrokes, gain access to device files and/or use location tracking technology may be used to collect study data. These devices may be owned by the participant or provided to the participant by the RECOVER mobile health platform core. These devices will be used in accord with the Terms of Service and/or the End User License Agreements (EULA) provided by the product or device vendor. These products and devices will only be used to collect study data with IRB approval and if the subject has agreed to all applicable Terms of Service.

11.13 Laboratory Procedures/Evaluations

11.13.1 Biospecimen collection overview

There are no screening laboratory assessments. All biospecimens are collected as outcome variables. Blood biospecimens may be collected in children/young adults ages 24 months to 25 years. Blood biospecimens may be collected either remotely (Tier 1 by self-administered dried blood spot collection kits) or via phlebotomy from an upper extremity vein performed at a study site or at home. All clinical laboratory testing will be performed at the local site or nationally accredited laboratory. Before collection of clinical laboratory testing in Tier 2 and Tier 3, the participant's electronic health record will be reviewed for clinical laboratory results available within the 3 months prior to the visit (where feasible). Only laboratory tests not available in the participant electronic health record in the 3-month period will be collected. Biorepository biospecimens will be processed and shipped as described below. Blood volume will be age adjusted according to the following table:

Table 4: Phlebotomy maximum volume by age group

Age	Maximum volume single visit blood draw (mL)	% Estimated Total Blood Volume
24 months – <72 months	2 mL per kg of body weight	< 5% total blood volume
72 months – <10 years	25	≤1.5
10 – 25 years	38	≤1.5

Pediatric Blood Volume Calculator: https://www.mdapp.co/pediatric-blood-volume-calculator-538/

For post-acute Tier 1, the home blood spot biospecimen collection will be conducted with Tasso M-20 blood collection kit. The Tasso-M20 has obtained CE mark certification and is registered with the FDA as a Class I product exempt from premarket notification [510(k)] requirements. This device uses a small lancet to collect 4 samples of 20µL ± 5% (total 80µL) from body regions with reduced density of pain receptors (shoulder for ages 6-25 years, shoulder, buttocks, or thigh for ages 2 to under 6 years). The device is applied to the skin area with light suction and completes collection of blood in about 2 minutes. The caregiver or young adult will receive the kit at home with instructions for home blood spot collection and a postage-paid pre-addressed envelope for return of the biospecimen to the biorepository core. Dried blood spot samples ship as an exempt human specimen (UN3373 exempt).

Biorepository specimens will be stored indefinitely, or until all of the sample is used up. Biospecimen collections for clinical laboratory testing and biorepository for children, young adults, and caregivers are listed by tier.

Tasso-M20 collections that are inadequate will be handled in accordance with the RECOVER Pediatric Cohort Manual of Procedures.

Tasso re-collections will be limited to one redraw.

If participant refuses blood collection at enrollment, sites will document non-completion of blood draw as a protocol deviation and will have the participant complete the remainder of Tier 1 procedures.

- Acute and Post-Acute infected participants without a blood draw will be eligible for Tier 2 promotion, as determined by the promotion algorithm.
- Uninfected participants without a blood draw **will not** be eligible for Tier 2 promotion.

11.13.2 Tier 1 biospecimen collections

Children and young adults ages 2-25 years (<5 mL, remote collection at study entry except for acute patients with collection at the 8-week visit)

- Saliva (oragene collection kit) for DNA (biorepository)
- Dried blood spot for SARS-CoV-2 spike and nucleocapsid antibody and biospecimen storage (<5 mL)
- If initial antibody test negative and subject lives in region with high COVID PCR positivity rate, phlebotomy for SARS-CoV-2 spike and nucleocapsid may be repeated up to two additional times at 3-month intervals

Caregivers (remote collection at study entry)

- Saliva (oragene collection kit) for DNA (biorepository)
- Dried blood spot for SARS-CoV-2 spike and nucleocapsid antibody and biospecimen storage

Other biological parent (optional remote collection at study entry)

- Saliva (oragene collection kit) for DNA (biorepository)

Note that in cases of a new, acute infection (within the last 30 days), sites will delay the collection of blood and saliva samples until 30 days after the onset of this new infection.

11.13.3 Tier 2 Acute biospecimen collections

Children ages 24 to <72 months (2 to <6 years) on-site or remote collection at week 8:

- Saliva (oragene collection kit) for DNA (biorepository)
- Serum: 5 mL Serum Separator Tube (SST) for storage in biorepository
  - 5 x 500uL Serum Aliquots for PBC
- Plasma: 10 mL EDTA tube for central lab and biorepository
  - 5 x 200uL
  - 8 x 500uL
  - 1 x WBC pellet
  - 3 x RBC pellets

Children and young adults ages 72 months and up (6 to 25 years) on-site or remote collection at week 8):

- Saliva (oragene collection kit) for DNA (biorepository)
- Serum: 5 mL Serum Separator Tube (SST) for storage in biorepository
  - 5 x 500uL Serum Aliquots for PBC
- Plasma: 10 mL EDTA tube for central lab and biorepository
  - 5 x 200uL
  - 8 x 500uL
  - 1 x WBC pellet
  - 3 x RBC pellets
- PBMC: Two x 4 mL Cell Preparation Tube for storage in biorepository
  - ~3 x 1mL PBMC Aliquots

11.13.4 Tier 2 Post-acute biospecimen collections

First post-acute Tier 2 visit (6 months)

Children ages 24 to <72 months (2 to under 6 years) on-site or remote collection at first post-acute Tier 2 visit:

- Serum: 5 mL Serum Separator Tube (SST) for storage in biorepository
  - 5 x 500uL Serum Aliquots
- Plasma: 10 mL EDTA tube for central lab and biorepository
  - 5 x 200uL

- 8 x 500uL
- 1 x WBC pellet
- 3 x RBC pellets

Children ages 72 months to <10 years (6 to under 10 years) on-site or remote collection at first post-acute (6 month) Tier 2 visit:

- Local labs (14 mL)
    - Sample types to be collected per local assay specifications to perform Tier 2 lab assessments as outlined in Appendix F.
- Central lab and PBC
    - Serum: Two 5 mL SST for central lab and biorepository
        - 1 x Intermediate Aliquot Container with ~1500uL for PBC to create:
            - 6 x 200uL
            - 1-2 x 500uL
        - 1 x 1000uL
        - 1 x 2000uL

Children and young adults aged 10 to 25 years on-site or remote collection at first post-acute (6 month) Tier 2 visit:

- Local labs (14 mL)
    - Sample types to be collected per local assay specifications to perform Tier 2 lab assessments as outlined in Appendix F.

- Central lab and PBC
    - 2 x 5 mL SST
        - 1 x 1000uL (serum)
        - 1 x 2000uL (serum)
    - 1 x Intermediate Container with ~1500uL of serum, shipped same day of collection, refrigerated to PBC
        - 1 x Intermediate Aliquot Container with ~1500uL for PBC to create:
            - 3 x 500uL
    - 1 x 10 mL EDTA
        - 5 x 200uL plasma
        - 8 x 500uL plasma
        - 1 x WBC pellet
        - 3 x RBC pellets
        - 1 x 100uL (plasma)
        - 2 x 200uL (plasma) aliquots
        - 2 x 500uL (plasma)
        - 2 x 1000uL (plasma)
        - 1 x ~1000uL WBC
        - 3 x 1000uL RBC pellet

Subsequent post-acute Tier 2 visits (12, 24, 36, and 48 months)

Children ages 3-5 years (total 15 ml, on-site or remote collection):

- Serum: 5 mL Serum Separator Tube (SST) for storage in biorepository
    - 5 x 500uL Serum Aliquots for PBC
- Plasma: 10 mL EDTA tube for central lab and biorepository
    - 5 x 200uL
    - 8 x 500uL
    - 1 x WBC pellet
    - 3 x RBC pellets

Children age 72 months to <10 years (6 to under 10 years) on-site collection at subsequent post-acute Tier 2 visits at months 12, 24, 36, and 48:

- Central lab and PBC
  - 1 x 5 mL SST
    - 5 x 500uL Serum Aliquots
  - 1 x 10 mL EDTA
    - 5 x 200uL
    - 8 x 500uL
    - 1 x WBC pellet
    - 3 x RBC pellets
  - 2 x 4mL CPT
    - ~3 x 1mL PBMC Aliquots

Children and young adults age 10 to 25 years on-site collection at subsequent post-acute Tier 2 visits at month 12, 24, 36, and 48)

- Central lab and PBC
  - 1 x 5 mL SST
    - 5 x 500uL Serum Aliquots
  - 1 x 10mL EDTA
    - 5 x 200uL
    - 8 x 500uL
    - 1 x WBC pellet
    - 3 x RBC pellets
  - 4 x 4 mL CPT
    - ~6 x 1mL PBMC Aliquots

Congenitally Exposed Cohort 24-month collection
Children enrolled in the congenitally exposed cohort will undergo biospecimen collection at the 24-month visit. In person collection is preferred but remote collection with a Tasso M-20 device can be used where venipuncture is not feasible.

Infant cohort in-person blood collection at 24 months
- Central lab and PBC (no more than 15mL according to blood collection parameters)
  - 1 x 5 mL SST
    - 1 x 1000uL of serum
    - 3 x 500uL of serum
  - 1 x 10 mL EDTA
    - 1 x 100uL
    - 2 x 200uL aliquots (plasma)
    - 2 x 500uL (plasma)
    - 2 x 1000uL (plasma)
    - 1 x ~1000uL WBC
    - 3 x 1000uL RBC pellet

Tasso collection for PBC (where venipuncture is not feasible)
  - Dried blood spot collection for SARS CoV-2 antibody and biorepository (~80uL)

11.13.5 Tier 3 biospecimen collections

Children and young adults age 3 to 25 years (total blood collection 13.1 ml, onsite collection two times approximately 1 year apart)

- Complete metabolic panel (1.6 ml SST)
- Complete blood count (0.5 ml pediatric EDTA tube)
- Lipid panel (2.0 ml pediatric SST)
- Hemoglobin A1c (0.5 ml pediatric EDTA tube)
- Urine protein:creatinine ratio

- D-Dimer (1 ml citrate plasma tube)
- High sensitivity Troponin (2.0 ml lithium heparin tube)
- High sensitivity C-reactive protein (0.5 ml lithium heparin tube)
- Procalcitonin (3 ml no additive tube)
- NT-pro-brain natriuretic peptide (0.5 ml pediatric EDTA tube)
- Insulin C-peptide (1.5 ml pediatric SST)
- Microbiome specimens: sputum, skin swabs, nasal swab, oral swab, urine, stool

11.14 Study Specific Biospecimens

11.14.1 Specimen Collection Procedures

Blood biospecimens may be collected in children/young adult participants aged 24 months and older. Blood biospecimens may be collected either remotely (Tier 1 by self-administered dried blood spot collection kits) or via phlebotomy from an upper extremity vein performed at a study site or at home. All on-site or home phlebotomy procedures will be performed by an individual with appropriate training and experience for pediatric population. Study staff will follow a standard operating procedure to verify subject identity and proper labeling at the time of phlebotomy.

Blood biospecimens for clinical testing will either be analyzed at the site local accredited laboratory or a national accredited laboratory provider. Blood biospecimens for biorepository will be processed locally as described below and shipped to the Mayo Clinic biorepository. Biorepository specimens will be stored using a UUID with no identifiers. The link to the UUID will be maintained at the local site and/or the central REDCap database. The biospecimens will be stored indefinitely, or until the sample is used up. Stored deidentified biospecimens may be used for future research as approved by the NIH Sponsor and RECOVER scientific leadership. Participants who withdraw from the study will have their stored biospecimens destroyed.

11.14.2 Specimen Preparation, Handling, and Storage

The Mayo Clinic Central biorepository will create blood collection kits for each visit. The kits will contain Blue Refrigerate Biohazard bag, tubes and aliquot tubes pre-labelled biorepository specimen ID collection for local freezing and storage, and return mailing container.

Samples collected in SST tubes will be cold centrifuged and aliquoted in 0.5 ml volumes, and placed in a -20C freezer for short-term storage. Frozen aliquoted serum specimens will be shipped on dry ice in containers provided by Mayo Clinic.

Samples collected in EDTA and CPT tubes will be placed in appropriate packaging for immediately shipment to Mayo clinic biorepository.

If standard phlebotomy is not feasible at Tier 2 visits, dried blood spot samples will be obtained by a lancet-based system and shipped to the biorepository.

11.14.3 Specimen Shipment

Biospecimens will be shipped to the Mayo Biorepository in Rochester Minnesota.

- Blood spot (Tasso m-20) and saliva collections obtained at home will be shipped directly to the biorepository by the participant.
- Frozen specimens collected at study sties will be batched and shipped once weekly on dry ice.
- CPT specimens collected at study sites will be shipped the same day as the study visit.

All specimens will be labeled according to the standard operating procedure and shipped in an appropriate biosafe container provided by Mayo Biorepository.

11.15 Questionnaire Administration

The names and domains covered by each of the questionnaires is listed above in the description of the Tier 1-3 procedures. Questionnaires will be administered either remotely via electronic online versions or paper versions, or during an onsite visit. Questionnaire have all been adapted for age ranges across the pediatric and young adult life spectrum. At each study visit the age-appropriate version of the questionnaire will be used. All the selected questionnaires have been previously validated with the exception of the symptom checklist for post COVID-19 symptoms. There is no validated version of this checklist due to the recent discovery of the post-acute sequelae of COVID-19.

12 Safety and Adverse Events

12.1 Definitions

Unanticipated Problems Involving Risk to Subjects or Others
Any event, incident, experience, outcome, or new information that meets all of the following criteria:

- unexpected in nature, severity, or frequency given the information provided in research-related documents and characteristics of the subject population being studied; and
- is related or possibly related to participation in the research; and
- suggests that the research caused harm to subjects or others or places subjects or others at increased risk of harm (including physical, psychological, economic, or social harm) than was previously known or recognized.

Please refer to 12.3.1 for reporting requirements.

Adverse Event
An **adverse event** (AE) is defined as any physical and psychological harm occurring to subjects during the course of participating in research, whether or not it is related to participation in the research (**excluding** symptoms, signs and co-morbidities already captured in the PASC symptom, co-morbidity, laboratory results or procedure results forms), or an adverse consequence of a study-related procedure. An AE can be any unfavorable or unintended event that is temporally related to the research.

Known manifestations of acute and post-acute SARS-CoV-2 infection will be recorded as endpoints on the PASC symptom, co-morbidity, laboratory results or procedure results forms in REDCap, rather than as AEs or SAEs, even if occurring in uninfected individuals.

Serious Adverse Event
Adverse events are classified as serious or non-serious. A **serious adverse event** is any AE that is:

- fatal
- life-threatening
- requires or prolongs hospital stay
- results in persistent or significant disability or incapacity
- an important medical event

Important medical events are those that may not be immediately life threatening, but are clearly of major clinical significance. They may jeopardize the participant, and may require intervention to prevent one of the other serious outcomes noted above. For example, drug overdose or abuse, a seizure that did not result in in-patient hospitalization, or intensive treatment of bronchospasm in an emergency department would typically be considered serious.

All adverse events that do not meet any of the criteria for serious should be regarded as **non-serious adverse events**.

Preexisting Condition

A preexisting condition is one that is present at the start of the study. A preexisting condition should be recorded as an adverse event if the frequency, intensity, or the character of the condition worsens during the study period.

Post-study Adverse Event
All unresolved adverse events will be followed by the investigator until the events are resolved, the subject is lost to follow-up, or the adverse event is otherwise explained. At the last scheduled visit, the investigator will instruct each subject to report any subsequent event(s) that the subject, or the subject's personal physician, believes might reasonably be related to participation in this study. The investigator will notify the study sponsor of any death or adverse event occurring at any time after a subject has discontinued or terminated study participation that may reasonably be related to this study. The sponsor will also be notified if the investigator should become aware of the development of cancer or of a congenital anomaly in a subsequently conceived offspring of a subject that has participated in this study.

12.2 Recording of Adverse Events

At each contact with the subject, the investigator will seek information on adverse events by specific questioning and, as appropriate, by examination. Information on all adverse events will be recorded immediately in the source document, and also in the appropriate adverse event module of the case report form (CRF). All clearly related signs, symptoms, and abnormal diagnostic procedures results will be recorded in the source document, though should be grouped under one diagnosis.

All adverse events occurring during the study period will be recorded. The clinical course of each event will be followed until resolution, stabilization, or until it has been determined that the study treatment or participation is not the cause. Serious adverse events that are still ongoing at the end of the study period will be followed until resolution. Any serious adverse event that occurs after the study period and is considered to be possibly related to study participation will be recorded and reported according to the same criteria as other serious adverse events.

12.3 Reporting of Serious Adverse Events and Unanticipated Problems

12.3.1 Investigator reporting: notifying the IRB

Federal regulations require timely reporting by investigators to their local IRB of unanticipated problems posing risks to subjects or others. The following describes the NYULMC IRB reporting requirements, though Investigators at participating sites are responsible for meeting the specific requirements of their IRB of record.

Federal regulations require timely filing of unanticipated problems posing risks to subjects or others to the local IRB. These events are:
- unexpected, AND
- related to study participation, AND
- serious or involve risks to subjects or others

This section also describes the NYULH IRB reporting requirements for other types of events, though investigators at participating sites are responsible for meeting any additional local requirements and/or those of the relevant sIRB. The following categories of events are considered reportable and require a submission to the IRB using the Reportable New Information e-submission form.

1. New or Increased Risk

Information arising from the study that indicates a new or increased risk or safety issue. For example:
- New information (e.g., an interim analysis, safety monitoring report, publication in the literature, sponsor report, or investigator finding) indicates an increase in the frequency or magnitude of a previously known risk or uncovers a new risk.
- Protocol violation that harmed subjects or others, or that indicates subjects or others might be at increased risk of harm.

- Complaint of a subject that indicates subjects or others might be at increased risk of harm or at risk of a new harm.

2. Unexpected Harm to a Subject or Other Individual

Any harm experienced by a subject or other individual(s) that, in the opinion of the investigator, is unexpected and related or possibly related to the research procedures. Harms can include psychological, economic, legal, and other non-physical harms.

- A harm is “unexpected” when its specificity or severity is inconsistent with risk information previously reviewed and approved by the IRB in terms of nature, severity, frequency, and characteristics of the study population.
- A harm is “probably related” to the research procedures if, in the opinion of the investigator, the research procedures more likely than not caused the harm.

Examples of harm include:

a. Death of a Research Subject. Investigators are required to report deaths of research participants to the IRB if the death was unanticipated and related or probably related to participation in the study.

b. Adverse Events. Only Unanticipated Adverse Events that are related to the research need to be reported to the IRB. As described above, RNI includes events that may increase risks or cause harm.

3. Non-Compliance

Non-compliance with federal regulations governing human research, NYU Langone Health’s HRP policies, or with IRB requirements or determinations, or allegations of such non-compliance.

4. Audits

External audits, inspections, or inquiries by a federal agency and any resulting reports (e.g., FDA Form 483).

5. Reports

Written reports of study monitors, reports to/from a study sponsor or other information that indicates a change to the risks or potential benefits of the research.

6. Researcher Error

Failure to follow the protocol due to the action or inaction of the investigator or research staff.

7. Breach of Confidentiality

Breach of subject or patient confidentiality, data breach, or data incident. Any unauthorized disclosure of subject’s personally identifiable information.

8. Unreviewed Change

Any change in the IRB-approved study protocol that was taken without prior IRB review to eliminate immediate hazard to subjects must be reported. This would include protocol violations and deviations.

A protocol violation refers to an accidental or unintentional change to the IRB-approved protocol that harmed subjects or others, or that indicates subjects or others may be at increased risk of harm. Examples: subject received the wrong dose of study medication.

9. Incarceration

Investigators must report to the IRB when a subject who is enrolled in a study that is not IRB-approved to involve prisoners becomes incarcerated and the study team plans to continue study activities with prisoners while incarcerated.

10. Complaint

Complaints made by a subject that are related to the study and either indicate increased risk and/or that cannot be resolved by the research team must be reported.

11. Suspension or Termination
Principal Investigators must report premature suspension or termination of the research by the sponsor, investigator, or institution.

12.3.2 When to Report Events

All reportable events, meaning those that are **unexpected** and meet the category of events above, must be reported to the NYU Langone Health IRB immediately but no later than ten (10) calendar days of becoming aware of the event.

If the reportable event is a subject's death that is unexpected and related to a study procedure, the site Principal Investigator should report such event to the IRB immediately but no later than five (5) calendar days of becoming aware.

If an event requires immediate intervention to prevent serious harm to subjects or others, the investigator may act accordingly to prevent harm and then must report the event and all interventions taken within five (5) days.

Investigators must report all other possible RNIs occurring at the local research site and non-local research sites to the IRB as soon as possible but no later than ten (10) business days from the date of the event or from the date the investigator is notified of the event.

The IRB will accept other reports when the Principal Investigator is unsure whether the event should be reported. The Principal Investigator should first contact IRB Operations by email or telephone to determine if the reporting is necessary under this Policy.

Events that do not meet the above criteria should be summarized and reported to the IRB at the time of continuing review.

12.3.3 Reporting Process

The reportable events noted above will be reported to the IRB using a Reportable New Information submission and will include a description of the event with information regarding its fulfillment of the above criteria, follow-up/resolution, and need for revision to consent form and/or other study documentation. Copies of each report and documentation of IRB notification and receipt will be kept in the Clinical Investigator's study file.

13 Study Oversight

13.1 Monitoring Board

Oversight of data and safety is provided by a RECOVER Observational Safety Monitoring Board appointed by NHLBI. The OSMB is composed of experts in longitudinal research methods, clinical experts in the manifestations of COVID-19 in adults, pregnant women, and children, biostatistics, bioethics, and patient/caregiver representatives. The OSMB may also appoint ad hoc members with subspecialty expertise in the diverse array of clinical manifestations of PASC.

A charter of the OSMB will be submitted for IRB review before starting enrollment. The OSMB will meet at least twice a year to review data on AEs, unanticipated events, patient-reported outcomes, data quality, and study recruitment as described in the committee charter, and make recommendations about study conduct to the NHLBI. As the RECOVER Pediatric PASC Investigator Consortium study does not involve any interventions, a pre-specified stopping rule for efficacy or futility is not indicated.

After each OSMB meeting, the OSMB determination letter and a summary report of adverse events will be prepared within 30 days and will be distributed by NHLBI staff to each principal investigator at the RECOVER Clinical Science Core for review. The summary report will contain the following information:

- A statement that a OSMB review of outcome data, adverse events, and information relating to study performance across all centers took place on a given date.
- A statement as to whether or not the frequency of adverse events exceeded what was expected and indicated in the informed consent.
- A statement that a review of recent literature relevant to the research took place.
- The OSMB's recommendation with respect to progress or need for modification of the protocol or informed consent. If the OSMB recommends changes to the protocol or informed consent, the rationale for such changes and any relevant data will be provided.
- A statement that if safety concerns are identified, the NHLBI Program Official will communicate these promptly to the investigators.

13.2 Data Safety Monitoring Plan

The Data and Safety Monitoring Plan for this trial will follow recommended monitoring principles for an observational study of a vulnerable population. Oversight of data and safety is provided by the RECOVER PASC Observational Safety Monitoring Board (OSMB) appointed by NHLBI. The OSMB will be composed of experts in longitudinal research (adult and pediatric populations), clinical experts in adult and pediatric manifestations of COVID-19, biostatistics, bioethics, and patient/caregiver representatives. The OSMB will also appoint ad hoc members with subspecialty expertise in the diverse array of clinical manifestations of PASC. The OSMB will meet at least twice a year as described in Section 13.1.

Each study site lead investigator will assume responsibilities for monitoring and reporting unanticipated events to the single IRB and the Clinical Science Core. The site lead investigator will provide requested follow-up information as requested by the IRB and Clinical Science Core. The Clinical Science Core will also perform routine monitoring of all sites in the RECOVER PASC consortium and issue queries for any protocol deviations. Not for cause audits for all sites in the RECOVER PASC consortium will also be conducted by an outside vendor with report to the IRB and Clinical Science Core.

14 Statistical Considerations

RECOVER-Pediatrics scientific aims are to:

- Characterize the prevalence and incidence of new onset or worsening symptoms related to PASC.
- Characterize the spectrum of clinical symptoms of PASC, including distinct phenotypes, and describe the clinical course and recovery.
- Identify risk and resiliency factors for developing PASC and recovering from PASC.
- Define the pathophysiology of PASC, including subclinical organ dysfunction, and identify biological mechanisms underlying the pathogenesis of PASC.

14.1 Sample size determinations

At least 6,000 participants will be recruited into the Pediatric Main, or *de novo*, cohort at Tier 1. This includes 800 acute infected participants, 1,200 uninfected participants (200 of whom will be randomized to the acute arm and 1000 of whom will be randomized to the post-acute arm), and 4,000 post-acute infected participants.

In the Pediatric Main Cohort at Tier 2, we will enroll 6,000 participants, which includes 5,400 infected and 600 uninfected participants. The uninfected sub-cohort includes all 200 participants who were initially randomized to the acute arm as well as 400 participants who are selected at random from the 1,000 participants who were initially randomized to the post-acute arm. The infected sub-cohort includes the post-acute infected participants who are promoted from Tier 1 as well as additional participants who are likely to have PASC who are recruited from Long COVID clinics and other subspecialty services. For the purposes of sample size calculations, we anticipate that at Tier 2, the infected participant population will include 3,600 participants with PASC and 1,800 without (a ratio of 2:1). 600 infected participants will progress to Tier 3; we assume, for the purposes of sample size calculations, that 400 have PASC and 200 do not.

Up to 10,000 participants will also be recruited from the ABCD cohort, who will complete only Tier 1 assessments. The pediatric cohort also includes 1,867 infants born in the context of maternal SARS-CoV-2 infection during pregnancy, 433 infants not born in the context of maternal SARS-CoV-2 infection during pregnancy, and 800 children in the MIS-C cohort.

Table 5: RECOVER Pediatrics Cohort sample size by infection status and type of enrollment

Cohort	Group	Sample size
Main (Tier 1)	Acute infected	800
Main (Tier 1)	Post-acute infected	4,000
Main (Tier 1)	Uninfected	1,200
ABCD		10,000
Congenital Exposure	Exposed	1,867
Congenital Exposure	Unexposed	433
MIS-C		800

Sample size determinations are based on a type 1 error rate of 0.01 and 90% power. In the analytic phase, a false discovery rate adjusted p-value will be used that appropriately accounts for the number of comparisons considered at that time.

In the infant cohort: Assuming a developmental milestone score is standardized to mean 0 and standard deviation 1, the minimum detectable difference between infants born in the context of maternal SARS-CoV-2 infection and infants not born in the context of maternal SARS-CoV-2 infection is 0.19.

In the main acute/post-acute cohort: Assuming the risk of PASC in the uninfected is 10%, the minimum detectable effect size for the difference in risk of PASC as measured at Tier 1 between infected and uninfected participants is 4.1%. Assuming the risk of PASC in the uninfected is 10%, and that ideally about 15-30% of acute/post-acute participants are asymptomatic during the acute phase of SARS-CoV-2 infection, the minimum detectable effect size for the difference in risk of PASC as measured at Tier 1 between asymptomatic infected and uninfected participants is 6.0%. Assuming the prevalence of a given binary Tier 2 feature is 50% among participants with PASC, the minimum detectable effect size for the difference in proportion with the feature between PASC+ and PASC- participants is 5.6%. Assuming the prevalence of a given binary Tier 3 feature is 50% among participants with PASC, the minimum detectable effect size for the difference in proportion with the feature between PASC+ and PASC- participants is 16.4%.

In the MIS-C cohort: Assuming the prevalence of cardiac manifestations (or any other Tier 1/2/3 feature) is 50% among participants with MIS-C, the precision of a prevalence estimate as described by a 95% confidence interval is ± 4.6%.

14.2 Statistical Methods

14.2.1 Methods of Data Collection

Structured data elements will be collected remotely by telephone with study personnel, by home visit by study personnel, through a secure encrypted mobile, email, or web-based platform, or (if no other option) by return of printed questionnaire by postal mail. Text messaging may be used with subject permission for collection of data without PHI. If preferred by the participant and participant caregiver, structured data may be collected in person at a study site.

Biospecimen collection will be handled in two ways, depending on stability of the sample:

- Samples for analytes that require rapid freezing will be processed locally and sent to the central RECOVER biorepository on dry ice.
- Other samples will be sent directly to the centralized RECOVER biorepository for processing.

- Off-protocol clinically obtained samples including cerebrospinal fluid, bronchoalveolar lavage specimens, procedural biopsies, and surgical pathology specimens will be tracked and either transferred from study site biorepository to the central RECOVER biorepository or linked by the UUID to the institutional RECOVER biorepository for future access.

14.2.2 Strategies for Study Modifications

This protocol is designed to be pragmatic and flexible in design. A governance structure will evaluate internal and external sources of data on a regular basis and make recommendations to the NIH Executive Committee and OSMB for any proposed protocol modifications. We may undertake the following procedures to guide protocol modifications over time:

1) The frequency of PASC will be monitored in real-time during the study. If the incidence or prevalence is found to be higher or lower than planned, recruitment strategies may be altered to deliberately undersample/oversample PASC cases.
2) Participant response burden will be monitored in real-time during the study. If burden is found to be excessive, it may be reduced by altering the data collection strategy, such as by increasing the assessment interval; reducing the number of data elements collected; increasing the availability of home-based assessments; and/or increasing participant reimbursement.
3) Free text responses to interval assessments will be monitored in real-time during the study. If a new symptom or outcome is being reported at a frequency >15% by participants, the symptom may be added to the data collection tool.
4) Data elements that are not NIH recommended CDE may be modified based on ongoing analysis by DRC; data elements that are not informative to PASC definition models may be removed, with substitution by new data elements.
5) PASC definition may be revised in an iterative manner based on existing PASC data, medical literature, and feedback from patient representatives, participants, and the scientific community. Updated PASC definitions may be used to implement a strategy to modify deeper phenotyping.
6) Tier 2 and Tier 3 assessments may be evaluated for futility at pre-specified intervals; protocol assessments will be adjusted accordingly and may include elimination of some assessments and introduction of other new assessments.

14.2.3 Overview of Analytic Approach to Aims

Full details of the analytic approach are included in the pediatric statistical analysis plan. The statistical analysis plan will include the following key elements.

Aim 1 is to characterize the incidence and prevalence of long-term sequelae, including clinical and biological features, severity and distinct sub-phenotypes, following SARS-CoV-2 infection. This will be achieved by estimating the incidence of PASC phenotypes among participants with SARS-CoV-2 infection or born to a mother with SARS-CoV-2 infection who are free of PASC-like symptoms and/or diagnoses prior to SARS-CoV-2 infection, compared with uninfected individuals free of PASC-like symptoms prior to the pandemic followed over the same time interval. These analyses will be performed in subgroups as detailed in Table 6 below. To identify PASC phenotypes, sub-phenotypes and severity based on clinical and biologic features, we will statistically compare the incidence among infected and uninfected individuals. Supervised and unsupervised approaches will be applied to characterize sub phenotypes.

Aim 2 is to characterize the clinical course and recovery of acute and post-acute sequelae over time from the time of study entry and to determine associated risk factors for PASC among SARS-CoV-2 infected, PASC positive individuals compared to infected PASC negative individuals and compared to uninfected individuals. We will characterize the patterns of outcomes of acute and post-acute sequelae over time using longitudinal data methods (e.g., general estimating equations and generalized linear mixed models) and functional principal component analysis. Longitudinal trajectories will be compared statistically between infected and uninfected individuals. We will additionally estimate and test the association of pre-infection and peri-infection risk and resiliency factors (e.g., social determinants of health, family dynamics, demographic, behavioral, biological factors and preexisting clinical and subclinical disease) prior to and following SARS-CoV-2 infection with the presence, severity and time to resolution of acute and post-acute sequelae using standard longitudinal and time-to-event models. These analyses will be performed in subgroups as detailed in Table 6

below. We will also estimate the incidence and prevalence of subclinical organ injury/disease after SARS-CoV-2 infection and compare the prognostic significance of subclinical organ injury/disease for incident clinical disease among SARS-CoV-2 infected versus uninfected individuals.

Aim 3 will define the pathophysiology of and mechanisms associated with the development of acute and post-acute sequelae including MIS-C, including the direct and indirect effects of SARS-CoV-2 infection on symptom onset and potential modifiers. We will estimate the direct and indirect effects of SARS-CoV-2 infection on the development of acute and post-acute sequelae, including potential mediation by post-traumatic responses (e.g., severe disease) and caregiver characteristics. We will also determine whether the occurrence of MIS-C after SARS-CoV-2 exposure modifies the trajectory of prior organ dysfunction, and/or risk of developing new organ injury compared with pre-pandemic injury, and identify possible pathophysiological mechanisms using mediation analysis based on longitudinal models (e.g., generalized linear mixed models) including interactions.

For all aims, additional analyses of pre-specified subgroups based on age, demographics, region of index SARS-CoV-2 infection if known, and COVID-19 health status of primary caregiver will be performed as sample sizes allow as detailed in the following Table.

Table 6: Planned subgroup analyses overall and by infection status at time of RECOVER enrollment

Subgroup	*Overall (N=)*	*SARS-CoV-2 Infected (N=)*	*SARS-CoV-2 Uninfected (N=)*	*Unadjusted Infected vs. Uninfected*
**Demographics**				
Female sex – count/total (%)	count/total (%)	count/total (%)	count/total (%)	*p-value*
Age – Median (IQR)	Median (IQR)	Median (IQR)	Median (IQR)	*p-value*
Age new born-2 years (infancy/toddlerhood)	count/total (%)	count/total (%)	count/total (%)	*p-value*
Age 3-5 years (early childhood)	count/total (%)	count/total (%)	count/total (%)	
Age 7-11 years (middle childhood)	count/total (%)	count/total (%)	count/total (%)	
Age 12-17 years (adolescence)	count/total (%)	count/total (%)	count/total (%)	
Age 18-25 years (young adulthood)	count/total (%)	count/total (%)	count/total (%)	
Race/Ethnicity				
White/non-Hispanic	count/total (%)	count/total (%)	count/total (%)	*p-value*
Black/non-Hispanic	count/total (%)	count/total (%)	count/total (%)	
Hispanic	count/total (%)	count/total (%)	count/total (%)	
Asian	count/total (%)	count/total (%)	count/total (%)	
Native Hawaiian/Pacific Islander	count/total (%)	count/total (%)	count/total (%)	
American Indian/Alaska Native	count/total (%)	count/total (%)	count/total (%)	
Other	count/total (%)	count/total (%)	count/total (%)	
Geographic region*				
HHS Region 1	count/total (%)	count/total (%)	count/total (%)	*p-value*
HHS Region 2	count/total (%)	count/total (%)	count/total (%)	
HHS Region 3	count/total (%)	count/total (%)	count/total (%)	
HHS Region 4	count/total (%)	count/total (%)	count/total (%)	
HHS Region 5	count/total (%)	count/total (%)	count/total (%)	
HHS Region 6	count/total (%)	count/total (%)	count/total (%)	
HHS Region 7	count/total (%)	count/total (%)	count/total (%)	
HHS Region 8	count/total (%)	count/total (%)	count/total (%)	
HHS Region 9	count/total (%)	count/total (%)	count/total (%)	
HHS Region 10	count/total (%)	count/total (%)	count/total (%)	
Rural	count/total (%)	count/total (%)	count/total (%)	*p-value*
**Caregiver information (at study entry)**				
History of SARS-CoV-2 infection	count/total (%)	count/total (%)	count/total (%)	count/total (%)

* https://www.hhs.gov/about/agencies/iea/regional-offices/index.html

14.3 Data Management Plan

14.3.1 Data Categories

RECOVER study data may be divided into two broad categories: Structured and Unstructured. Structured data can be simple (e.g., surveys/lab tests) or complex (e.g., sleep studies). REDCap will be used to capture structured data electronically. REDCap may include PII and PHI data to enable centralized coordination of biospecimen collection information.

14.3.2 Data Types

RECOVER study data may be divided into at least 12 different operational data types.

1. Patient questionnaires (in-person or submitted online; Structured)
2. Clinical site historical data (extant data; Structured→Complex)
3. Clinical site historical data (extant data; Unstructured)
4. EHR repository data (Structured→Complex)
5. Lab test results (Structured→Complex)
    i. Hospital
    ii. Commercial
    iii. Home
6. RECOVER biorepository inventory data (Biospecimens, slides; Structured→Complex)
7. Neuropsychological assessment data (Complex/Structured)
8. Functional assessment data (e.g., exercise testing, pulmonary/liver/kidney function; Structured→Complex)
9. Mobile health/wearable devices and computer software (e.g., fitness trackers, sleep monitors, Zio patch)
10. Advanced imaging data (CT and MRI; Unstructured)
11. Vaccination status data (Structured)
12. Vital signs and physiological testing data (e.g., PFTs, tilt table; Structured→Complex)

14.3.3 Functional data workflow summary

The RECOVER program studies the long-term effects of SARS-CoV-2 based on the study of several cohorts: Pediatric, Adult, Autopsy, and Pregnancy/Pediatric. In all cases, study participants are consented, samples are collected, and data is stored in central REDCap instance as described in the next section. Data may be entered into REDCap Central via four mechanisms: 1) direct entry into REDCap Central by CRCs at the clinic sites and 2) entry of data into surveys by participants in the study (and, in the case of the Pediatric cohort, their caregivers), 3) entry into an instance of REDCap by CRCs at the study sites, with subsequent data transfer to REDCap central, and 4) data transfer from existing research data repositories. The data are validated for quality using SAS within the FISMA envelope that hosts REDCap Central and then flows into a central i2b2 database ("I2b2 Data Hub") as a limited dataset for analysis. The data is made available to investigators via a front-end built into the i2b2 Data Hub.

14.3.4 Harvard Medical School (HMS) AWS Cloud Environment

RECOVER systems, including REDCap Central (all cohorts), the i2b2 Data Hub, and statistical analysis tools such as R, SAS, SQL Server, SHRINE, and Gitlab will reside on a FISMA Moderate compliant infrastructure. i2b2 will be the primary software component used to centrally store all data and provide investigator tools for querying, reporting and extraction of analysis data sets.

The REDCap Central environment is managed by HMS. It is developed on an existing fully authorized FISMA Moderate environment in Amazon Web Services (AWS) currently in use to support the NHLBI BioData Catalyst (BDC) project, as authorized by the NHLBI in March, 2021. This environment leverages all the management and security systems, controls, change control methodologies, training, documentation, and 3rd party security testing (e.g., Penetration Testing) and assessments (e.g., 3PAO reviews) in place for the HMS BDC project.

14.3.5 Data Storage

Data will be stored in a cloud infrastructure. To comply with the government's Cloud Smart policy, all PHI will live within a FISMA Moderate cloud environment that has received an ATO from NHLBI based on review by NHLBI's cybersecurity office.

14.3.6 Data Destruction

When participants withdraw from the study, their data will be destroyed in REDCap using standard REDCap functionality. The record, including its participant ID, will persist, as will records of ICFs that were signed, and the withdrawal record. All other data will be destroyed from REDCap. It will not be possible to restore this data once it is destroyed.

Per the study protocols, data that are stored in the i2b2 Data Hub will not be destroyed at the time of withdrawal.

When the study ends, all data will be de-identified in REDCap Central. The data in the i2b2 Data hub will persist in its existing de-identified form.

14.3.7 Data Integrity

Detailed Quality Control programs will be designed and deployed by the DRC to ensure and audit data integrity

14.3.8 Security Management

Data will be stored in cloud infrastructure. To comply with the government's Cloud Smart policy, all PHI will live within a FISMA Moderate cloud environment that has received an ATO from NHLBI, which includes network firewalls and systems for access control, change control, continuous monitoring, and training. A System Security Plan, which will be reviewed and approved by NHLBI as part of the ATO, describes the cybersecurity and IT management plan in detail.

14.3.9 Source Documents and Access to Source Data/Documents

Source data are all information, original records of clinical findings, observations, or other activities in a study necessary for the reconstruction and evaluation of the study. Source data are contained in source documents. Examples of these original documents, and data records include: hospital records, clinical and office charts, laboratory notes, memoranda, subjects' diaries or evaluation checklists, pharmacy dispensing records, recorded data from automated instruments, copies or transcriptions certified after verification as being accurate and complete, microfiches, photographic negatives, microfilm or magnetic media, x-rays, subject files, and records kept at the pharmacy, at the laboratories, and at medico-technical departments involved in the study.

The study case report form (CRF) is the primary data collection instrument for the study. All data requested on the CRF must be recorded. All missing data must be explained. If a space on the CRF is left blank because the procedure was not done or the question was not asked, write "N/D". If the item is not applicable to the individual case, write "N/A".

Access to study records will be limited to IRB-approved members of the study team. The investigator will permit study-related monitoring, audits, and inspections by the IRB/EC, the sponsor, government regulatory bodies, and University compliance and quality assurance groups of all study related documents (e.g., source documents, regulatory documents, data collection instruments, study data etc.). The investigator will ensure the capability for inspections of applicable study-related facilities (e.g., pharmacy, diagnostic laboratory, etc.).

Participation as an investigator in this study implies acceptance of potential inspection by government regulatory authorities and applicable University compliance and quality assurance offices.

15 Ethics/Protection of Human Subjects

15.1 Ethical Standard

The investigator will ensure that this study is conducted in full conformity with Regulations for the Protection of Human Subjects of Research codified in 45 CFR Part 46.

15.2 Institutional Review Board

The protocol, informed consent form(s), recruitment materials, and all participant materials will be submitted to the IRB for review and approval. Approval of both the protocol and the consent form must be obtained before any participant is enrolled. Any amendment to the protocol will require review and approval by the IRB before the changes are implemented to the study. All changes to the consent form will be IRB approved; a determination will be made regarding whether previously consented participants need to be re-consented.

NYU will serve as the sIRB for sites without prior reliance agreements. The single IRB (sIRB) model has been adapted for the RECOVER initiative to permit reliance on existing central IRBs in some of the site networks. For site networks with existing reliance agreements, each central IRB will execute a memorandum of understanding with the NYU sIRB to standardize protocol document handling and IRB review processes across all sites and maintain harmonization with the local requirements for each existing central IRB. This hybrid model has been reviewed and approved by NIH for the RECOVER initiative.

15.3 Informed Consent Process

Informed consent will be obtained and documented in writing before participation in study procedures. Study sites will identify potential participants in their available recruitment pools (extant cohorts, clinical cohorts, acute cohorts, and/or post-acute cohorts). The research study will be explained in lay terms to each potential research participant/parent/legal guardian in their preferred language. The overall common consent document will include:

1) consent for participation in RECOVER Post-Acute Tier 1, Acute Tier 2 and optional participation in Post-Acute Tier 2 activities;
2) separate consent for participation in RECOVER Tier 3 activities
3) consent for recontact for future participation in research;
4) consent to obtain and link data from electronic health records, regional health information exchanges, non-financial claims data and the National Death Index;
5) consent for broad sharing of deidentified data and specimens through RECOVER databases and specimen repositories (in addition to other NIH-designated repositories).
6) optional return of genetic information and other research laboratory findings, including option to change decision for minor reaching age of majority
7) Subjects who reach the age of majority during study participation will be re-consented.

The potential participant (if above age of majority) or a parent/legal guardian above the age of majority will provide written documentation of informed consent before undergoing any study procedures. Informed assent will also be obtained. The consent process may be conducted by telephone, secure video conference platform approved for exchange of PHI, or in person. The investigator or suitable designated delegate will conduct a meeting with the study candidate and legally authorized representative (if younger than the age of majority) to review all the required elements of informed consent and to address all questions about the study. Adjunct education materials including flyers, booklets, slide presentations, animations, videos may be made available to the participant in print or electronic media formats to enhance comprehension of the informed consent process and scope of the study. All such materials will be approved by the IRB prior to use. Secure encryption will be used for email delivery of any of these materials. Comprehension of the study procedures and risks will be confirmed with standardized questions to the participant and/or parent/legal guardian. A standardized teach back method will be implemented as needed to ensure understanding of the key aspects of participation before enrollment. Subjects will be provided information on how to contact an appropriate individual for pertinent questions about the research and their rights and whom to contact in the event that they sustain a research-related injury.

Participants and their caregivers will only be approached for consent during periods of clinical stability. If required, the treating physician will be notified prior to the consent process. Consents may be conducted remotely using telephone or electronic audio and audiovisual platforms or in person. The child and caregiver will be at home or other private location during the consent process.

Documentation of consent will be recorded electronically via REDCap. For participants below the age of majority, the consent process will be conducted with the child and the parent/legal guardian. The parent/legal guardian will sign the consent form; the child may sign the age-appropriate assent form. For participants above the age of majority, the consent process will be conducted with the young adult; the young adult will sign the consent form.

The parent/legal guardian or young adult above the age of majority will be sent a link to the REDCap consent form via encrypted email, and potential subjects will be given the phone number of a study team member to call after they have reviewed the consent. The study team member will then explain the consent to the parent/legal guardian or young adult, and answer all questions. The study team member will then administer standardized questions with scoring rubric to assess understanding of key elements of the protocol. The study team member will re-teach the protocol details for any incorrect responses to the standardized questions, and will repeat the questions to re-assess understanding. The consent process will be temporarily suspended If the parent/legal guardian or young adult continues to offer incorrect responses after 3 attempts of re-teaching. Additional teaching and educational will be provided at a later date. Only parent/legal guardians or young adults who demonstrate understanding of the key elements of the protocol will electronically sign the informed consent document in REDCap. Study personnel will verify identification before sanctioning an individual's electronic signature. An electronic or printed signed copy will be provided to the participant and a copy of the participant's consent to participate will be kept on a password-protected and secure drive at each study site.

For children, the IRB-approved assent document will be made available to the child for review with their parent/legal guardian. The study team will explain the assent document to the child and parent/legal guardian and answer all questions. Child understanding of the key elements of the assent document will be assessed by the study team and parent/legal guardian. The child will be given the opportunity to sign the assent document or provide verbal assent.

The REDCap eConsent link will be sent to the IRB of record before use in the study. Language consistency with the IRB-approved consent will be reviewed and approved by the IRB of record before eConsent is initiated.

If a parent/legal guardian is unable to provide an electronic signature during a remote visit, he or she will be required to sign a paper copy of the informed consent in the presence of a witness. The signature and date of the witness will also be required on the paper copy. A separate record of the required elements of the ICF process will be documented in the participant's study record.

A participant older than the age of majority will provide written informed consent; for participants younger than the age of majority, informed consent will be provided by the legal authorized representative, with review of the youth assent document by the participant. If a participant attains the age of majority during participation in the study, the participant will be approached for re-consent. Caregivers and other biological parents older than the age of majority will provide written informed consent; for primary caregivers and other biological parents younger than the age of majority, the legally authorized representative for the caregiver or other biological parent will provide written informed consent, with review of the assent document by the caregiver/other biological parent.

15.3.1 Consent/Assent and Other Informational Documents Provided to Participants

Consent forms describing in detail the study intervention, study procedures, and risks are given to the participant and written documentation of informed consent is required prior to starting intervention. The following consent materials are submitted with this protocol:

- Informed consent form Caregiver
- Informed consent form children ages newborn-5 years congenitally exposed (Tiers 1 and 2)
- Informed consent form children ages newborn-5 years post-natally exposed (Tiers 1 and 2)
- Informed consent form children ages 6-17 years (Tiers 1 and 2)
- Informed consent form children ages 6-17 years (Tier 3)
- Informed consent form children ages 3-5 years (Tier 3)
- Informed consent form young adults ages 18-25 years (Tiers 1 and 2)
- Informed consent form young adults ages 18-25 years (Tier 3)
- Informed consent form children ages 3-17 years with history of MIS-C (Tiers 1, 2, and 3)
- Informed consent form young adults ages 18-25 years with history of MIS-C (Tiers 1, 2, and 3)
- Informed consent form other biological parent
- Assent for children 7-17 years of age (Tiers 1 and 2)
- Assent for children 7-17 years of age (Tier 3)

15.3.2 Consent Procedures and Documentation

Informed consent is a process that is initiated prior to the individual's agreeing to participate in the study and continues throughout the individual's study participation. Extensive discussion of risks and possible benefits of participation will be provided to the participants and their families. Consent forms will be IRB-approved and the participant will be asked to read and review the document.

For parents/legal guardians of child participants and young adult participants above the age of majority, the investigator will explain the research study and contents of the consent form and answer any questions that may arise. All parents/legal guardians and young adults will receive a verbal explanation in terms suited to their comprehension of the purposes, procedures, and potential risks of the study and of their or their children's rights as research participants. Parents/legal guardians of child participants and young adult participants will have the opportunity to carefully review the written consent form and ask questions prior to signing. The parents/legal guardians and young adult participants should have the opportunity to discuss the study with their surrogates and carefully consider their decision prior to agreeing to participate. The parent/legal guardian of the child participant or young adult participant will sign the informed consent document prior to any procedures being done specifically for the study. The parent/legal guardian or young adult may withdraw consent at any time throughout the course of the study. A copy of the signed informed consent document will be given to the parent/legal guardian or young adult participant for their records. The rights and welfare of the participants will be protected by emphasizing to them that the quality of their medical care will not be adversely affected if they decline to participate in this study.

For children, the IRB-approved assent document will be made available to the child for review with their parent/legal guardian. The study team will explain the assent document to the child and parent/legal guardian and answer all questions. Child understanding of the key elements of the assent document will be assessed with standard questions with re-teaching as needed. The child will be given the opportunity to sign the assent document or provide verbal assent.

A copy of the signed informed consent document and any signed assent document will be stored in the subject's research record. The consent process, including the name of the individual obtaining consent, will be thoroughly documented in the subject's research record. Any alteration to the standard consent process (e.g., use of a translator, consent from a legally authorized representative, consent document presented orally, etc.) and the justification for such alteration will likewise be documented.

15.3.3 Posting of Clinical Trial Consent Form

The proposed study consent form will be posted on clinicaltrials.govand a public website at recovercovid.org.

15.4 Participant and Data Confidentiality

Information about study subjects will be kept confidential and managed according to the requirements of the Health Insurance Portability and Accountability Act of 1996 (HIPAA). Those regulations require a signed subject authorization informing the subject of the following:

- What protected health information (PHI) will be collected from subjects in this study?
- Who will have access to that information and why?
- Who will use or disclose that information?
- The rights of a research subject to revoke their authorization for use of their PHI.

In the event that a subject revokes authorization to collect or use PHI, the investigator, by regulation, retains the ability to use all information collected prior to the revocation of subject authorization. For subjects that have revoked authorization to collect or use PHI, attempts will be made to obtain permission to collect at least vital status (i.e., that the subject is alive) at the end of their scheduled study period.

Investigators in this research will take all reasonable measures to protect the confidentiality of the medical records of patients and their families. Multiple measures to protect confidentiality are described in the following paragraphs.

15.4.1 REDCap database security

The REDCap instance will be hosted and managed by Harvard Medical School (HMS) in their FISMA Moderate-compliant AWS cloud infrastructure. The security of the HMS infrastructure is governed by alignment with 18 security control families, as defined by FISMA at the Moderate level. In general, as per FISMA, only HMS technical staff and delegates will have access to infrastructure, networking, systems administration, access control, monitoring, and security testing systems. Approved DRC personnel can have applications administrative access to modify REDCap forms, undertake data quality research, and other data processing tasks. Investigators will not be able to have direct access to study data outside of the data entered by their sites. The mechanisms for this access have not yet been determined.

15.4.2 Data storage security

The RECOVER i2b2 Data Hub (the Hub) is the central location where all RECOVER data will exist or be indexed. It will contain the eCRF and some eConsent data from REDCap and some biospecimen data from the Biorepository, as well as all other data, such as EHR data, that is collected on study participants. The i2b2 data hub will live within a FISMA-moderate cloud environment. Only DRC staff will have direct access to the i2b2 Data Hub infrastructure. DRC staff will be responsible for the creation of staff user access. Data in the i2b2 Data Hub will exist as a Limited Data Set with no direct identifiers. There will be a web-based query tool that will be available to privileged study investigators, with proper site credentials and human research participants training, for aggregate queries only.

15.4.3 Storage of Study Materials

Investigators will take all reasonable measures to protect the confidentiality of the study participants through the measures used in all PASC studies, including storage of study materials in locked, secure locations accessible only to study investigators, knowledge of the subject's name only for the minimal time needed for coordination of study logistics, use of a UUID with no personal identifiers to maintain confidentiality and elimination of all PHI in the final study database, and use of secure password protected computer access and encrypted transmission of patient information.

15.4.4 Hashed identifiers

A unique subject hashed identifier (UUID) will be assigned to each study participant. The hashed identifier is a universal subject ID that allows researchers to share data specific to a study participant without exposing personally identifiable information (PII) and at the same time be able to match participants across labs, databases, or research studies. Personal information will not be included in the final research database, but rather we will only generate a unique set of encrypted codes that can then be decrypted to determine if the subject already exists within a data repository. The hashed identifier will allow data from this study to be combined with data from other research studies or databases in an effort to improve outcomes in children and adolescents who have had SARS-CoV-2 infection with or without PASC.

15.4.5 Datavant Tokenization and Privacy Protecting Record Linkage (PPRL) Technology

An important goal of RECOVER is linking data from disparate sources. To achieve this goal, the DRC will guide the implementation of Privacy Preserving Record Linkage (PPRL) using Datavant technology to securely connect the same individuals across different data sources while maintaining the participants' privacy.

Datavant's de-identification technology replaces private patient information with an encrypted "token" that can't be reverse engineered to reveal the original information. The technology can create the same patient-specific tokens in any data set, so that different data sets can be combined using the patient tokens to match corresponding records without ever sharing the underlying patient information. The process can be thought of in two distinct steps: token creation and privacy protecting record linkage.

15.4.5.1 Tokenization

Tokens are created from PII and they can be built from many different combinations of PII elements. The specific tokens to be created and PII elements required are specified below.

The processes to be used for RECOVER token creation are still being designed. The token creation may occur locally at the sites or centrally at the DRC. In the first case (and most popular among the site PIs), the site installs Datavant software locally to create the participant tokens using PII. The PII used to create the tokens stays at the local site and does not get sent to the DRC. Only the resulting token is sent to the DRC. In the second case, the site would rely on the DRC to create the token, which requires that the site send PII needed to create the tokens to the DRC. Both processes involve working with Datavant to create the site-specific encrypted tokens.

15.4.5.2 Linkage

Datavant tokens allow corresponding patient records to be matched across data sets without ever sharing PHI. The matching or linkage will occur at the DRC or at the enrolling site using Datavant software, which will live inside the FISMA-moderate cloud infrastructure. The consortium sites will be trained in the proper implementation of the Privacy Preserving Record Linkage “Hash tokens” consistent with the model being applied to other NIH repositories and programs

15.4.6 Limited Data Set

The data stored in i2b2 Data Hub is a limited data set of identifiable patient information as defined in the Privacy Regulations issued under the Health Insurance Portability and Accountability Act (HIPAA). A limited data set does not include any of the following information:

- Names
- Street addresses or postal address information with the exception of town/city, state and zip code
- Phone/Fax numbers
- E-mail addresses
- Social Security numbers
- Medical records numbers
- Health plan beneficiary numbers
- Other account numbers
- Certificate and license numbers
- Vehicle identifiers and serial numbers, including license plates
- Device identifiers and serial numbers
- URLs and IP addresses
- Biometric identifiers such as fingerprints, retinal scans and voice prints
- Full face photos and comparable images

15.4.7 Reporting of Incidental Findings

Test results determined by CLIA-certified clinical laboratories and imaging and other clinical testing results determined by licensed medical professionals that are analytically valid will be recorded in the participant medical record and will be reviewed by the Principal Investigator or other designated licensed medical professional at each site. If the Principal Investigator or licensed designee determine that the result is clinically significant or medically actionable, the participant will be contacted by telephone or in-person to explain the test findings within one week of the return of the test results. The participant will also be advised to follow up with their primary care physician. Any additional testing ordered by the primary care physician will be paid by the participant or their insurance company.

Test results determined in research laboratories that cannot be validated in CLIA-certified clinical laboratories will not be recorded in the medial record and will not be returned to the participant.

15.4.8 Reporting of Genetic Testing

The Whole Genome Sequencing (WGS) studies for the RECOVER study will be performed in a CLIA-certified laboratory with results interpreted by a board-certified clinical molecular geneticist or molecular genetic pathologist or equivalent. Analytically valid replicated results from the CLIA-certified laboratory that are defined as clinically actionable according to the standards and guidelines defined by the American College of Medical Genetics and Genomics and the Association for Molecular Pathology (ACMG-AMP) guidelines may be disclosed to the participants if all of the following criteria are met:(33-35)

- The genetic finding has important health implications for the participant and the associated risks are established and substantial.
- The genetic finding is actionable, that is, there are established therapeutic or preventive interventions or other available actions that have the potential to change the clinical course of the disease.
- The test is analytically valid and the disclosure plan complies with all applicable laws.
- During the informed consent process or subsequently, the study participant has opted to receive his/her individual genetic results.

All disclosure of clinically actionable genetic results will be guided by the RECOVER WGS Core Laboratory in collaboration with the RECOVER Clinical Science Core. The consent form will inform participants of the potential for return of actionable results from WGS and the potential risks associated with the disclosure of the genetic information. For participants who elect to be informed of their clinically actionable genetic results, the validated, replicated result will be shared with the site PI or designated study personnel, who will use their local process and policies for re-identification of the participant and referral if needed for evaluation and counseling, which may include involvement of their local genetics team and/or the participant's cardiologist or other healthcare providers. Participants who reach the legal age of majority during the study will be re-consented and given the opportunity to opt-in or opt-out of return of genetic information.

Clinical genetic testing targeting known disease-associated variants will not be performed. There is a reasonable possibility that no findings will result from this research effort. If variants are detected, it may be years before any clinical utility of these findings is realized. Further, if samples are "anonymized" prior to release to other investigators for future research, it may not be possible to trace the results back to the participant.

15.4.9 Certificate of Confidentiality

Participant confidentiality is strictly held in trust by the participating investigators, their staff, and the sponsor(s) and their agents. This confidentiality is extended to cover testing of biological samples and genetic tests in addition to the clinical information relating to participants. Therefore, the study protocol, documentation, data, and all other information generated will be held in strict confidence. No information concerning the study or the data will be released to any unauthorized third party without prior written approval of the sponsor.

The study monitor, other authorized representatives of the sponsor, or representatives of the IRB may inspect all documents and records required to be maintained by the investigator, including but not limited to, medical records (office, clinic, or hospital) and pharmacy records for the participants in this study. The clinical study site will permit access to such records.

The study participant's contact information will be securely stored at each clinical site for internal use during the study. At the end of the study, all records will continue to be kept in a secure location for as long a period as dictated by local IRB and Institutional regulations.

In accord with NIH policy (https://grants.nih.gov/policy/humansubjects/coc/coc-nih-funded.htm), a Certificate of Confidentiality will be issued automatically as a term of this NIH-funded award, and no physical certificate will be issued. With this Certificate, the researchers of this study cannot be forced to disclose information that may identify a participant, even by a court subpoena, in any federal, state, or local civil, criminal, administrative, legislative, or other proceeding. The Certificate cannot be used to resist a request for information from the United States government when it is used for evaluating federally funded study projects or for information that must be disclosed to meet the requirements of the Food and Drug Administration (FDA). A Certificate of Confidentiality does not prevent a participant or his/her family from voluntarily releasing

information about the participant's involvement in this research. If an insurer, employer, or other person obtains a participant's or family's written consent to receive research information, then the researchers will not use the Certificate to withhold that information.

A genome-wide association study (GWAS) performed with samples collected in this study will comply with the NIH Policy for Sharing of Data Obtained in NIH Supported or Conducted GWAS, which calls for investigators funded by the NIH for GWAS to 1) share de-identified genetic (genotypic and phenotypic) data through a centralized NIH data repository; and 2) submit documentation that describes how the institutions have considered the interests of the research participants, such as privacy and confidentiality. Submission of data to the NIH GWAS repository will be consistent with the permissions and limitations delineated on the study consent signed by study participants. Information from DNA analyses and clinical studies or medical records may be placed into a central data repository in the future, such as the National Center for Biotechnology Information repository. Data and samples will be de-identified before submission to this or any other central repository.

15.4.10 Research Use of Stored Human Samples, Specimens, or Data

- Intended Use: Samples and data collected under this protocol may be used to study mechanisms and clinical manifestations of SARS-CoV-2 infection and other disease states in the future. It is anticipated that DNA testing may be performed in the future.
- Storage: Access to stored samples will be limited with policies and procedures requiring multiple reviews prior to release of any samples for analysis. Samples and data will be stored using UUID codes assigned by the investigators until the aliquots are used up. Data will be stored in REDCap. Only investigators authorized by Mayo Clinic and the RECOVER Scientific Leadership will have access to the samples and data.
- Tracking: Data will be tracked using the central research database at the RECOVER PASC Investigator Consortium Data Resource Core at Massachusetts General Hospital. Each specimen will be labeled and tracked with a UUID.
- Disposition at the completion of the study: All stored samples will be sent to the RECOVER PASC biorepository at Mayo Clinic. Study participants who request destruction of samples will be notified of compliance with such request and all supporting details will be maintained for tracking.

15.5 Future Use of Stored Specimens

Biospecimens are defined as any tissues, bodily fluids (such as blood [Tasso m-20 and venipuncture], saliva), and excreta (such as stool and urine) that are collected as part of the RECOVER cohort protocols. With the participant's approval and as approved by the NYU sIRB and consortium central IRBs, de-identified biological coded biospecimen samples will be stored at the RECOVER PASC biorepository at Mayo Clinic. Participants may be permitted to restrict certain uses of biospecimens. These samples may be used for research into the causes of PASC, complications of PASC, understanding risk factors for PASC, and to develop diagnostic tests and treatments for PASC. These samples may also be used in other areas of research not directly related to PASC, including research conducted by business entities. Whole genome sequencing may be performed, but "true" clinical genetic testing targeting known disease-associated variants will not be performed. The RECOVER PASC biorepository at Mayo Clinic may transfer biospecimens to other biorepositories in the future as designated by the NIH Sponsor. The RECOVER PASC biorepository at Mayo Clinic may also be provided with a UUID that will allow linking the biological specimens with the phenotypic data from each participant, maintaining the masking of the identity of the participant. Only the RECOVER investigators at enrolling sites and the Data Resource Core will have access to the information linking the coded subject ID and subject identity.

During the conduct of the study, an individual participant can choose to withdraw consent to have biological specimens stored for future research. Subjects who reach the age of majority after study entry will have the opportunity to opt-out of storage of biospecimens at that time. However, withdrawal of consent with regard to biosample storage will not be possible after the study is completed, at which time the linking information between the subject coded ID and subject identity has been destroyed and only anonymized samples remain.

Access to stored biospecimens will be provided through the RECOVER PASC biorepository at Mayo Clinic or other designated biorepository as determined by the policies and procedures of the NIH Sponsor.

16 Data Handling and Record Keeping

16.1 Data Collection and Management Responsibilities

Data collection is the responsibility of the study staff at the site under the supervision of the site PI. The investigator is responsible for ensuring the accuracy, completeness, legibility, and timeliness of the data reported.

All source documents should be completed in a neat, legible manner to ensure accurate interpretation of data. Black ink is required to ensure clarity of reproduced copies. When making changes or corrections, cross out the original entry with a single line, and initial and date the change. Do not erase, overwrite, or use correction fluid or tape on the original.

Copies of the electronic CRF (eCRF) will be provided for use as source documents and maintained for recording data for each participant enrolled in the study. Data reported in the eCRF derived from source documents should be consistent with the source documents or the discrepancies should be explained and captured in a progress note and maintained in the participant's official electronic study record.

Research data will be entered into a central REDCap database, and then de-identified and stored as a limited data set in the central data capture system provided by the RECOVER PASC Consortium Data Resource Core at Massachusetts General Hospital. A central instance of REDCap will be used for capture of structured data from enrolling sites. The data capture system meets Federal data security requirements and includes password protection and internal quality checks, such as automatic range checks, to identify data that appear inconsistent, incomplete, or inaccurate. Research data will be entered directly from the source documents or transferred from existing research databases at the enrolling sites. At the end of the study, all identifiers will be removed from the central REDCap database. Identifiers may remain in the local site research database if the participant has provided consent for contact for future research.

16.2 Study Records Retention

Study documents will be retained for the longer of 3 years after close-out or 5 years after final reporting/publication. These documents should be retained for a longer period, however, if required by local regulations. No records will be destroyed without the written consent of the sponsor, if applicable. It is the responsibility of the sponsor to inform the investigator when these documents no longer need to be retained.

16.3 Protocol Deviations

A protocol deviation is any noncompliance with the study protocol and study manual of operations requirements. The noncompliance may be either on the part of the participant, the investigator, or the study site staff. As a result of deviations, corrective actions are to be developed by the site and implemented promptly.

It is the responsibility of the site to use continuous vigilance to identify and report deviations within 5 working days of identification of the protocol deviation. All deviations associated with change in risk to participants or compromise of scientific integrity of the study must be addressed in study source documents, reported to RECOVER program scientific directors at NIH, the Clinical Science Core Principal Investigators at NYU Langone Health, and the RECOVER PASC DRC Principal Investigators at Massachusetts General Hospital, the IRB or record, and the RECOVER OSMB. Protocol deviations that do not impact risk or scientific integrity must be recorded on note to file and reported to the OSMB at 6-month intervals. Protocol deviations must be reported to the local IRB per their guidelines. The site PI/study staff is responsible for knowing and adhering to their IRB requirements. Further details about the handling of protocol deviations will be included in the MOP.

16.4 Publication and Data Sharing Policy

This study will comply with the NIH Public Access Policy, which ensures that the public has access to the published results of NIH funded research. It requires scientists to submit final peer-reviewed journal manuscripts that arise from NIH funds to the digital archive PubMed Central upon acceptance for publication.

17 Study Finances

17.1 Funding Source

This study is financed through a grant from the Other Transactional Authority (OTA) of the US Federal Government. The study is overseen by the National Institutes of Health, NHLBI

17.2 Costs to the Participant

There are no costs to the participant related to participation in the study. The OTA grant will pay for all study related procedures and costs.

17.3 Participant Reimbursements or Payments

Sites will offer patients a nominal reimbursement for participation in the remote interval assessments and more substantial reimbursement for participation in the more complex or time-consuming Tier 2 and Tier 3 testing commensurate with their time and effort.

18 Study Administration

18.1 Study Leadership

The scientific leadership for the study and oversight of sites participating in the study is provided by the RECOVER Clinical Science Core (CSC) at the NYU Grossman School of Medicine. The RECOVER CSC collaborates with the RECOVER Data Resource Core at Massachusetts General Hospital for data management and data storage at the RECOVER biorepository at Mayo Clinic for biospecimen storage. The activity of the RECOVER cohort studies is overseen by a Steering Committee composed of the RECOVER Principal Investigators and NIH program leadership, an Executive Committee composed of NIH Institute leadership, and an OSMB composed of experts in longitudinal observation studies, epidemiology, bioethics, and biostatistics. The Steering Committee, Executive Committee and OSMB will meet at a minimum of twice yearly.

Protocol modifications may be proposed by the RECOVER Clinical Science Core based on interim analysis of study data, safety data, data derived from other RECOVER cohorts, and new information in the medical literature. A study design committee will make recommendations to the RECOVER Steering Committee. The recommendation will be further reviewed by the data safety monitoring board and the RECOVER Executive Committee. If a protocol modification is approved by the Executive Committee and safety monitoring board, a modified proposal, and associated modified consent forms and other protocol documents will be submitted to the NYU sIRB for review. There will be no changes in study procedures until the modification is approved by the NYU sIRB. Urgent changes in study procedures may be implemented to maintain subject safety. In this instance, the NYU sIRB will be notified of the change within 72 hours.

19 Conflict of Interest Policy

All recipient institutions and investigators in the PASC consortium will comply with the requirements of 42 CFR 50, Subpart F, "Responsibility of Applicants for Promoting Objectivity in Research for which PHS Funding is Sought" (FCOI Regulation), as implemented in the 2011 Final Rule for grants and cooperative agreements.

The requirements promote objectivity in research by establishing standards that provide a reasonable expectation that the design, conduct, or reporting of research funded under PHS grants or cooperative agreements will be free from bias resulting from any conflicting financial interest of an investigator. An "investigator" is someone defined as the PD/PI and any other person, regardless of title or position who is responsible for the design, conduct, or reporting of research funded by PHS, or proposed for such funding which may include, for example, collaborators or consultants.
Each Institution shall maintain an up-to-date, written, enforced policy on financial conflicts of interest that complies with the regulation and make the policy available via a publicly accessible Web site.

These FCOI requirements do not apply to Federal employees or Federal agencies. Federal agencies have their own set of rules governing financial conflicts of interest for employees.

When submitting a grant application, the signature of the Authorized Organization Representative (AOR) will certify each PASC Consortium applicant institution's compliance with the requirements of 42 CFR 50, Subpart F, including that:

- There is in effect at the Institution an up-to-date, written and enforced administrative process to identify and manage Financial Conflicts of Interest (FCOI) with respect to all research projects for which NIH funding is sought or received;
- The Institution shall promote and enforce Investigator compliance with the regulation's requirements including those pertaining to disclosure of Significant Financial Interests;
- The Institution shall identify and manage FCOIs and provide initial and ongoing FCOI reports to the NIH consistent with this subpart;
- When requested, the Institution will promptly make information available to the NIH/HHS relating to any Investigator disclosure of financial interests and the Institution's review of, and response to, such disclosure, whether or not the disclosure resulted in the Institution's determination of an FCOI;
- The Institution shall fully comply with the requirements of the regulation.

20 APPENDICES

20.1 Appendix A: Enrollment Entry Timepoints

Table 7: Enrollment Entry Timepoints

Time point	Status	Cohort	Outcomes
Prior to SARS-CoV-2 infection	Develops SARS-CoV-2 infection	SARS-CoV-2 infected cohort	Define PASC based on excess incidence compared to referent population.<br><br>Determine clinically interpretable sub-types using supervised or unsupervised learning approach.
	No SARS-CoV-2 infection	Uninfected control cohort	
Current, acute SARS-CoV-2 infection	Active SARS-CoV-2 infection	SARS-CoV-2 infected cohort	To characterize the clinical features, pathophysiology, and mechanisms that influence PASC symptoms and their progression over time and across the childhood lifespan.
Post SARS-CoV-2 infection	Prior SARS-CoV-2 infection	PASC**+** Cases<br>PASC**-** Cases	To characterize the clinical course and recovery of PASC over time

20.2 Appendix B. Overview Schedule of Assessments by Recruitment Pool

All study visits for the main pediatric cohort are timed from study entry. All Post-Acute Tier 1 assessments, including initial biospecimen collection, will be completed within 3 months of enrollment. All Acute baseline assessments are expected to be completed by the start of the Week 8 visit window (+7 weeks post-enrollment). Tier 2 acute visits will have a time window of ± 7 days and are optional in the event of severe acute illness. Though the preferred target for the Acute Tier 2 8-week visit is 7 to 9 weeks post-study entry, it can be scheduled up to 12 weeks from study entry. For the first post-acute Tier 2 visit, sites will target a completion window of 2-6 months post-enrollment with the ability to complete up to 9 months post-enrollment. Thus, it is expected that, relative to the Month 6 target, the first post-acute Tier 2 visit will be scheduled within -4 to +3 months. Visits thereafter will have a target window of +/- 3 months relative to study time point. Post-acute Tier 2 visits will include an on-site component and a remote component for completion of questionnaires. Tier 3 will be conducted over an interval of 3 visits about one year apart, between months 12-30 after study entry and repeated between months 24-42 after study entry.

Table 8: Schedule of Assessments: Main Pediatric Cohort Tiers 1-3 Ages 6-25 years

Tier 1 and Tier 2 Assessments	BL		Acute Phase				Post Acute Phase				
Visit Window	<3m after enroll*		±7 days	±7 days	±7 days**		-4 /+3 months	-3 /+3 months			
Identity	B										
Demographics	B										
Child Birth History					B						
Child Current Health Status					B		B	B	B	B	B
Special Health Care Needs Screener					B		B	B	B	B	B
PROMIS global health scale	B				B		B	B	B	B	B
Current COVID Infection History	B										
First COVID Infection History	B										
Weekly COVID Infection History			B	B	B						
COVID Infection History (Followup)							B	B	B	B	B
Related Conditions					B		B	B	B	B	B
COVID Testing History					B						
COVID Family Infection					B		B	B	B	B	B
COVID Symptoms	B		B	B	B		B	B	B	B	B
COMPASS-31							B	B	B	B	B
COVID Vaccine History					B		B	B	B	B	B
COVID Health Consequences					B		B	B	B	B	B
Social Determinants of Health					B		B	B	B	B	B
Child Wellbeing							B	B	B	B	B
Tier 1 Biospecimen	O										
Anthropometry & vitals											
Electrocardiogram					R		R	R	R	R	R
Spirometry (Pulmonary Function)					R		R	R	R	R	R
Pulse Oximetry					R						
Tier 2 Acute Biospecimen					O						
Clinical Laboratory Post-Acute Tier 2											
Active Standing Test							R	R	R	R	R
Joint Flexibility (Beighton Scale)							R	R	R	R	R
Neurocognitive Development (NIH Toolbox)							R	R	R	R	R
Emotional/Mental Health							R	R	R	R	R
Post-Acute Tier 2 biospecimen							O	O	O	O	O
Tier 3 Assessments											
Cardiac ECHO								R	R		
Cardiac MRI								R	R		
Pulmonary Function Tests								R	R		
Lung microbiome (sputum induction)								R	R		
Cardiopulmonary Exercise Testing								R	R		
Abdominal organ structure								R	R		
Brain MRI								R	R		
Brain EEG								R	R		
Neurocognitive testing								R	R		
Tier 3 biospecimen collection for microbiome and clinical laboratory								O	O		
Visit Timepoint:	BL	…	2w	4w	8w	…	6m	12m	24m	36m	48m

*Target visit window for Baseline is +3 months for Post-Acute and up to the start of the Week 8 visit (+7 weeks) for Acute
**Target visit window for 8 week visit is ± 7 days (7-9 weeks) but can be scheduled up to 12 weeks post study entry

Table 8 Legend: Assessment type indicated by both color and letter as follows: Blue (B)=Questionnaires; Orange (O)=Biospecimen Collection; Red (R)=Clinical Assessments

Table 8 Notes:
1. Visits scheduled based on time elapsed from study entry
2. Visit assessments by age described in Sections 11.5
3. Biospecimen collection described in Section 11.14
4. Electrocardiogram, spirometry and clinical laboratory collection required at first post-acute Tier 2 visit and optional at subsequent post-acute Tier 2 visits if successfully collected

Note: Acute-schedule visits at Week 2 and Week 4 will not be completed if the participant is greater than 36 days post-infection, Please refer to the Manual of Procedures for further information.

Table 9: Schedule of Assessments: Main Pediatric Cohort Tiers 1-3 Ages Newborn-5 years

Tier 1 and Tier 2 Assessments	BL		Acute		Phase			Post	Acute	Phase	
Visit Window	<3m after enroll*		±7 days	±7 days	±7 days**		-4 /+3 months		-3 /+3	months	
Identity	B										
Demographics	B										
Child Birth History					B						
Child Current Health Status					B		B	B	B	B	B
Special Health Care Needs Screener					B		B	B	B	B	B
PROMIS	B				B		B	B	B	B	B
Current COVID Infection History	B										
First COVID Infection History	B										
Weekly COVID Infection History			B	B	B						
COVID Infection History (Followup)							B	B	B	B	B
Related Conditions					B		B	B	B	B	B
COVID Testing History					B						
COVID Family Infection					B		B	B	B	B	B
COVID Symptoms	B		B	B	B		B	B	B	B	B
COMPASS-31							B	B	B	B	B
COVID Vaccine History					B		B	B	B	B	B
COVID Health Consequences					B		B	B	B	B	B
Social Determinants of Health					B		B	B	B	B	B
Child Wellbeing							B	B	B	B	B
Child Behavior Checklist							B	B	B	B	B
ASQ^							B	B	B	B	B
ASQ Social/Emotional^							B	B	B	B	B
MCHAT^							B	B	B	B	B
Tier 1 Biospecimen	O										
Anthropometry & vitals					R		R	R	R	R	R
Electrocardiogram					R		R	R	R	R	R
Spirometry (Pulmonary Function)					R		R	R	R	R	R
Pulse Oximetry					R						
Tier 2 Acute Biospecimen					O						
Clinical Laboratory Post-Acute Tier 2											
Joint Flexibility (Beighton Scale)							R	R	R	R	R
Neurocognitive Development (NIH Toolbox)							R	R	R	R	R
Emotional/Mental Health							R	R	R	R	R
Post-Acute Tier 2 biospecimen							O	O	O	O	O
Tier 3 Assessments											
Cardiac ECHO								R	R		
Pulmonary Function Tests								R	R		
Abdominal organ structure								R	R		
Brain EEG								R	R		
Neurocognitive testing								R	R		
Tier 3 biospecimen collection for microbiome and clinical laboratory								O	O		
Visit Timepoint:	BL	…	2w	4w	8w	..	6m	12m	24m	36m	48m

*Target visit window for Baseline is +3 months for Post-Acute and up to the start of the Week 8 visit (+7 weeks) for Acute
**Target visit window for 8 week visit is ± 7 days (7-9 weeks) but can be scheduled up to 12 weeks post study entry
^Measures have age-range limitations outlined in section 11.5.1

Table 9 Legend: Assessment type indicated by both color and letter as follows: Blue (B)=Questionnaires; Orange (O)=Biospecimen Collection; Red (R)=Clinical Assessments

Table 9 Notes:

1. Visits scheduled based on time elapsed from study entry
2. Visit assessments described in Sections 11.5
3. Biospecimen collection described in Section 11.14
4. Electrocardiogram, spirometry and clinical laboratory collection required at first post-acute Tier 2 visit and optional at subsequent post-acute Tier 2 visits if successfully collected

Note: Acute-schedule visits at Week 2 and Week 4 will not be completed if the participant is greater than 36 days post-infection, Please refer to the Manual of Procedures for further information.

Table 10: Caregiver Schedule of Assessments: Main Pediatric Cohort

**Tier 1 and Tier 2 Assessments**	**BL**		**Post Acute Phase**				
Visit Window	<3m after enroll*		-4 /+3 months	-3 /+3 months			
Identity	B						
Demographics	B						
PROMIS	B			B	B	B	B
Current Health Status	B			B	B	B	B
First COVID infection history	B						
Most recent COVID infection history	B						
COVID infection history (followup)				B	B	B	B
COVID testing history	B						
COVID vaccine history	B			B	B	B	B
COVID symptoms	B			B	B	B	B
COVID health consequences	B			B	B	B	B
Household social determinants of health	B		B	B	B	B	B
Caregiver social determinants of health	B			B	B	B	B
Caregiver wellbeing	B			B	B	B	B
Biospecimens	O						
Visit Timepoint:	BL	…	6m	12m	24m	36m	48m

*Target visit window for Baseline is +3 months for Post-Acute and up to the start of the Week 8 visit (+7 weeks) for Acute
**Target visit window for 8 week visit is ± 7 days (7-9 weeks) but can be scheduled up to 12 weeks post study entry

Table 9 Legend: Assessment type indicated by both color and letter as follows: Blue (B)=Questionnaires; Orange (O)=Biospecimen Collection

Table 11: Schedule of Assessments: MIS-C Cohort

Tiers 1 and 2 Assessments	BL		Post Acute		
Demographics	B				
Social determinants of health	B		B		B
Medical history	B		B		B
COVID health impact	B		B		B
Vaccination status	B		B		B
PASC Symptoms	B		B		B
Anthropometry & vitals			R		R
PROMIS			B		B
Diet			B		B
Activity			B		B
Tier 3 Assessments					
Cardiac ECHO			R		R
Cardiac MRI			R		R
Pulmonary Function Tests			R		R
Lung microbiome (sputum induction)			R		R
Cardiopulmonary exercise testing			R		R
Abdominal organ structure			R		R
Brain MRI			R		R
Brain EEG			R		R
Neurocognitive testing			R		R
Tier 3 Microbiome biospecimen collection			O		O
**Visit Timepoint:**	BL	…	12m		24m

Table 11 Legend: Assessment type indicated by both color and letter as follows: Blue (B)=Questionnaires; Orange (O)=Biospecimen Collection; Red (R)=Clinical Assessments

Table 11 Notes:

1. Visits scheduled base on time elapsed from index date of hospitalization
2. Tier 2 and 3 assessments may be scheduled contemporaneously within specified time windows
3. Biospecimen collection described in Section 11.14
4. MIS-C participants will undergo selection of up to three Tier 3 assessments according to participant medical history and symptoms

Table 12: Schedule of Assessments: ABCD Cohort

Tier 1 Assessments	BL
**Demographics**	B
**Child Birth History**	B
Child Current Health Status	B
Special Health Care Needs Screener	B
PROMIS	B
COVID Infection History	B
Related Conditions	B
COVID Testing History	B
COVID Family Infection	B
COVID Symptoms	B
COVID Vaccine History	B
COVID Health Consequences	B
Social Determinants of Health	B
Child Wellbeing	B
Tier 1 biospecimen collection	O

Table 12 Legend: Assessment type indicated by both color and letter as follows: Blue (B)=Questionnaires; Orange (O)=Biospecimen Collection

Table 12 Notes:

1. Biospecimen collection described in Section 11.14
2. ABCD cohort will participate in Tier 1 assessments; selected participants may be referred to another RECOVER site for participation in Tier 2 and Tier 3 procedures if geographically feasible

Table 13: Schedule of Assessments: Congenitally Exposed Infants ages newborn-5 years (born to mothers with and without SARS-CoV-2 infection during pregnancy)

**Tiers 1 and 2 Assessments**	**BL**		**Visits**				
**Identity**	B						
Demographics	B						
Child Birth History	B						
Child Current Health Status			B	B	B	B	B
Special Health Care Needs Screener			B	B	B	B	B
PROMIS			B	B	B	B	B
COVID Infection History	B		B	B	B	B	B
Related Conditions			B	B	B	B	B
COVID Family History			B	B	B	B	B
COVID Symptoms			B	B	B	B	B
COVID Vaccine History			B	B	B	B	B
COVID Health Consequences			B	B	B	B	B
Social Determinants of Health	B		B	B	B	B	B
Brief Infant Sleep Questionnaire					B		
Medical history (NSCH)			B	B	B	B	B
ASQ			B		B	B	
Growth/Exam			B	B	R	R	R
MCHAT				B			
ASQ Social/Emotional				B			
CBCL Behavioral Problems					B	B	
Developmental Profile-4						B	
Tier 2 biospecimen collection					O		
Bayley Scales					R		
Differential Ability Scales-II						R	R
Visit Timepoint:	BL	…	12m	18m	24m	36m	48m

Table 13 Legend: Assessment type indicated by both color and letter as follows: Blue (B)=Questionnaires; Orange (O)=Biospecimen Collection; Red (R)=Clinical Assessments

Table 13 Notes:

1. Visits scheduled base on time elapsed from birth
2. Visit assessments described in Section 11.9.1
3. Biospecimen collection described in Section 11.14

20.3 Appendix C: Proposed Sample Size by Tier

Up to 19,300 children and young adults (age newborn through 25 years) may be enrolled. Up to 19,50019,300ivers may be optionally enrolled with the child or young adult. Based on past history of SARS-CoV-2 exposure and post exposure history, there are 3 distinct modules for participation:

Main PASC Cohort Module. The total number of children and young adults (age newborn through 25 years) enrolled will be adjusted to achieve a total of 5400 participants with SARS-CoV-2 infection in Tier 2 of the main PASC Cohort Module (3600 with PASC and 1800 without PASC). This total will include 1000 participants with acute SARS-CoV-2 infection enrolled in Tier 1. The main PASC Cohort Module of Tier 2 will also include 600 participants without SARS-CoV-2 infection. These 600 uninfected control participants will be randomly assigned to Tier 2 follow-up schedule to match the acute infected cohort (n=200) or the post-acute infected cohort (n=400). Approximately 10% of the infected participants in this cohort will participate in Tier 3.

The children and young adults followed longitudinally in Tier 2 of the main cohort are selected based on a sampling scheme that prioritizes the acute arm as well as children and youth in the post-acute arm with a greater likelihood of having PASC. Promotion to Tier 2 occurs as follows: 1) All children/young adults in the acute arm with or without history of infection will be promoted; 2) children/young adults in the post-acute arm with a history of infection will be promoted at a rate dependent on their likelihood of PASC based on prior Long COVID diagnoses, Tier 1 PROMIS global health measure responses, and symptoms screener survey responses. 40% of children/young adults without known infection in the post-acute arm, selected at random, will be promoted. In addition to promoting children and young adults from Tier 1, children and young adults will also be recruited from Long COVID clinics and subspecialty services to achieve the target sample size in Tier 2 of 6,000. These children and young adults will complete both Tier 1 and Tier 2 assessments.

Promotion to Tier 2: The promotion algorithm relies on three items from the PROMIS-10 Global Health, each scored on a 5-point Likert scale of 1-Poor, 2-Fair, 3-Good, 4-Very Good, 5-Excellent:

- “In general, would you say [your/child’s name] health is?”
- “In general, how would you rate [your/child’s name] physical health?”
- “In general, how would you rate [your/child’s name] mental health, including mood and ability to think?”

The algorithm also relies on the presence of major and minor symptoms that started during or after a COVID infection that lasted for more than 4 weeks.

Promotion occurs based on the likelihood of screening positive for PASC:

Table 13: Promotion Algorithm Used in the de novo RECOVER-Pediatrics Cohort for Selecting Children and Young Adults for the Longitudinal Follow-Up (Tier 2)

Type of participant	Subgroup	Subgroup criteria	Promotion Rate to Tier 2
“Acute infected”: Children and young adults who reported having a COVID infection <30 days prior to enrollment	All	None	100%

"Post-acute infected": Children and young adults who reported having a COVID infection >30 days prior to enrollment	High probability of PASC[a]	Any of the following:<br><br>1. Prior diagnosis of Long COVID or MIS-C based on study site medical record review or referral by a health care provider<br>2. Recruitment from a Long COVID clinic<br>3. ≥1 fair/poor responses on the global health PROMIS scale[b] and ≥1 major or minor symptom reported[c]<br>4. ≥1 good/fair/poor responses on the global health PROMIS scale[b] and ≥ 1 major symptom reported[c]<br>5. ≥1 good/fair/poor responses on the global health PROMIS scale[b] and ≥2 minor symptoms reported[c]	100%
	Medium probability of PASC[a]	Any of the following:<br><br>1. ≥1 good responses on the global health PROMIS scale[b] and ≥1 major or minor symptom reported[c]<br>2. ≥1 very good/good/fair/poor responses on the global health PROMIS scale[b] and ≥1 major symptom reported[c]<br>3. ≥1 very good/good/fair/poor responses on the global health PROMIS scale[b] and ≥2 minor symptoms reported[c]	100%
	Low probability of PASC[a]	Does not meet high or medium probability of PASC criteria	20%
"Uninfected": Children or Young adults without a known history of a COVID infection	Acute arm	At enrollment, 17% of the "uninfected" group were randomly assigned to participate in the acute arm of the study.	100%
	Post-acute arm	At enrollment, 83% of the "uninfected" group were randomly assigned to participate in the post-acute arm of the study. 40% of this group will be randomly assigned to participate in Tier 2	40%

Note on Table 13: All participants assigned to the Acute arm are automatically promoted to Tier 2. Thus, the **Lower** likelihood of 20% applies only to participants in the Post-Acute arm.

[a] Responses to the PROMIS Global Health Scales and the presence of major and minor symptoms are used to categorize participants who are post-acute infected as high, medium, or low probability of PASC.

[b] The PROMIS Global Health Scales are self-reported or caregiver-reported measures of overall, physical, and mental health for young adults and children, respectively. The three questions from the caregiver-reported version that are used in the algorithm, include:

1) "In general, would you say your child's health is?;
2) "In general, how would you rate your child's physical health?"; and
3) "In general, how would you rate your child's mental health, including mood and ability to think?"

Responses include: Excellent, Very Good, Good, Fair, or Poor.

[c]Not all symptoms are asked of all participants, as many are age-specific (e.g., fewer symptoms assessed for younger children) and sex-specific (e.g., menses related symptoms).

Pregnancy Cohort Module. 2000 infants (age 0-2 years) born to mothers infected with SARS-CoV-2 during pregnancy (congenitally exposed) and 500 infants born to mothers not infected with SARS-CoV-2 during pregnancy will be enrolled and will participate in a separate Tier 1 and Tier 2 assessment schedule.

ABCD Cohort Module. 10,000 adolescents enrolled in the ABCD study will be enrolled for determination of COVID exposure and PASC symptoms in Tier 1 assessments. A subset of subjects will be offered participation in Tiers 2 and 3 based on geographic location.

MIS-C. 800 children and young adults with history of MIS-C will participate in a subset of Tier 2 and Tier 3 procedures.

20.4 Appendix D: Tiers 1 and 2 Data Elements

Table 14. Survey Topics in Tiers 1 and 2 Questionnaires

Survey Instrument	Topic	Asked in Tier 2 follow-up surveys	Source of survey if not developed for RECOVER
Household-Level and Child-Level Surveys			
Demographics	Name and contact information	✓	
Demographics	Alternate contacts	✓	
Demographics	Date of birth		
Demographics	Sex assigned at birth		All of Us Research Program
Demographics	Gender identity		All of Us Research Program
Demographics	Race and ethnicity		All of Us Research Program
Demographics	Languages spoken		California Health Interview Survey
Demographics	Country of origin		American Community Survey (ACS)
Demographics	Educational attainment (grade, school type)		National Health and Nutrition Examination Survey (NHANES)
Child birth history	Birth mother age		National Survey of Children's Health
Child birth history	Child birth weight		National Survey of Children's Health
Child birth history	Child birth length		National Survey of Children's Health
Child birth history	Prematurity/ gestational age		National Survey of Children's Health
Child birth history	Delivery type		
Child birth history	NICU admission		
Child birth history	Pregnancy complications		
Child birth history	Breastfeeding		
Child current health status	Child current length or height	✓	National Survey of Children's Health
Child current health status	Child current weight	✓	National Survey of Children's Health
Child current health status	Biological parents' height and weight		
Child current health status	Child menses	✓	
Child current health status	Child disabilities		National Survey of Children's Health
Child current health status	Household smoking exposure	✓	
Special Health Care Needs Screener	Special Health Care Needs Screener	✓	Children with Special Health Care Needs (CSHCN) Screener
Special Health Care Needs Screener	Asthma	✓	National Survey of Children's Health

Survey Instrument	Topic	Asked in Tier 2 follow-up surveys	Source of survey if not developed for RECOVER
Special Health Care Needs Screener	Cerebral Palsy	✓	National Survey of Children's Health
Special Health Care Needs Screener	Diabetes	✓	National Survey of Children's Health
Special Health Care Needs Screener	Epilepsy or seizure disorder	✓	National Survey of Children's Health
Special Health Care Needs Screener	Heart problem	✓	National Survey of Children's Health
Special Health Care Needs Screener	Frequent or severe headaches, including migraines	✓	National Survey of Children's Health
Special Health Care Needs Screener	Tourette's syndrome or tics	✓	National Survey of Children's Health
Special Health Care Needs Screener	Anxiety (feeling nervous or anxious)	✓	National Survey of Children's Health
Special Health Care Needs Screener	Depression (feeling very sad)	✓	National Survey of Children's Health
Special Health Care Needs Screener	Down syndrome	✓	National Survey of Children's Health
Special Health Care Needs Screener	Blood disorders	✓	National Survey of Children's Health
Special Health Care Needs Screener	Cystic fibrosis	✓	National Survey of Children's Health
Special Health Care Needs Screener	Other genetic or inherited condition	✓	National Survey of Children's Health
Special Health Care Needs Screener	Problems with behavior	✓	National Survey of Children's Health
Special Health Care Needs Screener	Developmental delay	✓	National Survey of Children's Health
Special Health Care Needs Screener	Intellectual disability	✓	National Survey of Children's Health
Special Health Care Needs Screener	Speech or other language disorder (problems with talking or understanding words)	✓	National Survey of Children's Health
Special Health Care Needs Screener	Learning disability (problem with learning)	✓	National Survey of Children's Health
Special Health Care Needs Screener	Autism or Autism Spectrum Disorder (ASD)	✓	National Survey of Children's Health
Special Health Care Needs Screener	Attention Deficit Disorder (ADD) or Attention Deficit/Hyperactivity Disorder (ADHD)	✓	National Survey of Children's Health
Special Health Care Needs Screener	Eating disorders (like Anorexia or Binge eating disorder)	✓	National Survey of Children's Health
Special Health Care Needs Screener	Other health problems	✓	
Global Health	Self-reported or caregiver-reported overall, physical and mental health	✓	Early Childhood Parent Report Global Health 8a; PROMIS Parent Proxy Scale v1.0 – Global Health 7; PROMIS-10 v1.2
COVID infection history	Infection date	✓	

Survey Instrument	Topic	Asked in Tier 2 follow-up surveys	Source of survey if not developed for RECOVER
COVID infection history	How family learned about COVID infection	✓	
COVID infection history	Presence of symptoms	✓	
COVID infection history	Duration of symptoms	✓	
COVID infection history	Symptom severity	✓	
COVID infection history	Health care utilization during COVID infection	✓	
COVID infection history	COVID treatments	✓	
Related conditions	Multisystem Inflammatory Syndrome in Children (MIS-C)	✓	
Related conditions	POTS (Postural Orthostatic Tachycardia Syndrome) or other form of dysautonomia or autonomic dysfunction	✓	
Related conditions	Long COVID diagnosis	✓	
COVID Testing History	Testing history		
COVID Testing History	Testing access		
COVID Family Infection	COVID infection		
COVID Family Infection	COVID-related hospitalization		
COVID Family Infection	COVID-related death	✓	
COVID Symptoms	General symptoms or problems	✓	
COVID Symptoms	Symptoms or problems in the eyes, ears, nose, and throat	✓	
COVID Symptoms	Symptoms or problems involving the heart and lungs	✓	
COVID Symptoms	Symptoms or problems involving the belly	✓	
COVID Symptoms	Symptoms or problems involving the skin, hair, and nails	✓	
COVID Symptoms	Symptoms or problems involving the bones and muscles	✓	
COVID Symptoms	Symptoms or problems involving the brain and nerves	✓	
COVID Symptoms	Symptoms or problems involving feelings or behavior	✓	
COVID Symptoms	Symptoms or problems involving periods	✓	
COMPASS-31	Symptoms associated with dysautonomia	✓	COMPASS-31
COVID vaccine history	Child COVID vaccine history	✓	
COVID vaccine history	Birth mother COVID vaccine history while pregnant		

Survey Instrument	Topic	Asked in Tier 2 follow-up surveys	Source of survey if not developed for RECOVER
COVID vaccine history	Birth mother COVID vaccine history while breastfeeding		
COVID vaccine history	COVID vaccine intentions		
COVID Health Consequences	Perceived weight status	✓	Youth Risk Behavior Survey
COVID Health Consequences	Child diet	✓	Youth Risk Behavior Survey
COVID Health Consequences	Physical activity	✓	Youth Risk Behavior Survey
COVID Health Consequences	Outdoor play	✓	
COVID Health Consequences	Screen time	✓	Youth Risk Behavior Survey
COVID Health Consequences	Sleep	✓	Youth Risk Behavior Survey
COVID Health Consequences	School disruption	✓	
COVID Health Consequences	Grades	✓	
COVID Health Consequences	Developmental services (Early intervention, Individualized Education Programs, home visiting)	✓	
COVID Health Consequences	Discipline	✓	Quick Parenting Assessment
COVID Health Consequences	Caregiver-child relationship quality	✓	Adult Child Relationship Scale
COVID Health Consequences	Cognitive stimulation (reading, teaching, playing, talking)	✓	StimQ cognitive home environment questionnaire (self-report version of infant/toddler; preschool; elementary school age)
Social Determinants Of Health	Household composition		
Social Determinants Of Health	Birth order		
Social Determinants Of Health	Housing		
Social Determinants Of Health	Marital status		
Social Determinants Of Health	Health care utilization	✓	National Survey of Children's Health
Social Determinants Of Health	Health literacy		Brief health literacy screener
Social Determinants Of Health	Health insurance	✓	
Social Determinants Of Health	Unmet needs	✓	
Social Determinants Of Health	Health care access	✓	

Survey Instrument	Topic	Asked in Tier 2 follow-up surveys	Source of survey if not developed for RECOVER
Social Determinants Of Health	COVID-related guidelines (e.g., masking, social distancing)		
Social Determinants Of Health	Financial difficulties	✓	
Social Determinants Of Health	Financial assistance programs	✓	
Social Determinants Of Health	Food insecurity	✓	USDA Core Food Security Module
Social Determinants Of Health	Perceived neighborhood safety	✓	
Social Determinants Of Health	Neighborhood cohesion	✓	
Social Determinants Of Health	Discrimination	✓	Everyday Discrimination Scale
Social Determinants Of Health	Early childhood experiences		Other Childhood Stressors
Social Determinants Of Health	Mental health	✓	DSM-5 Cross-Cutting Symptom Measure
Caregiver-Level Surveys			
Identity	Caregiver relationship to child		
Demographics	Caregiver date of birth		
Demographics	Caregiver sex assigned at birth		All of Us Research Program
Demographics	Caregiver gender identity		All of Us Research Program
Demographics	Caregiver race and ethnicity		All of Us Research Program
Demographics	Caregiver languages spoken		California Health Interview Survey
Demographics	Caregiver country of origin		American Community Survey (ACS)
Global Health	Caregiver overall, physical and mental health	✓	PROMIS global health scale
Current health status	Caregiver disabilities		
COVID infection history	Caregiver infection date	✓	
COVID infection history	How caregiver learned about their own COVID infection	✓	
COVID infection history	Caregiver presence of symptoms	✓	
COVID infection history	Caregiver duration of symptoms	✓	
COVID infection history	Caregiver symptom severity	✓	
COVID infection history	Caregiver health care utilization during COVID infection	✓	
COVID infection history	Caregiver COVID treatments	✓	

Survey Instrument	Topic	Asked in Tier 2 follow-up surveys	Source of survey if not developed for RECOVER
COVID Testing History	Caregiver testing history		
COVID vaccine history	Caregiver COVID vaccine history	✓	
COVID Symptoms	Caregiver general symptoms or problems	✓	
COVID Symptoms	Caregiver symptoms or problems in the eyes, ears, nose, and throat	✓	
COVID Symptoms	Caregiver symptoms or problems involving the heart and lungs	✓	
COVID Symptoms	Symptoms or problems involving the belly	✓	
COVID Symptoms	Caregiver symptoms or problems involving the skin, hair, and nails	✓	
COVID Symptoms	Caregiver symptoms or problems involving the bones and muscles	✓	
COVID Symptoms	Caregiver symptoms or problems involving the brain and nerves	✓	
COVID Symptoms	Caregiver symptoms or problems involving feelings or behavior	✓	
COVID Symptoms	Caregiver symptoms or problems involving periods	✓	
COVID Health Consequences	Caregiver perceived weight status	✓	Behavioral Risk Factor Surveillance System (BRFSS)
COVID Health Consequences	Caregiver diet	✓	Behavioral Risk Factor Surveillance System (BRFSS)
COVID Health Consequences	Caregiver physical activity	✓	Behavioral Risk Factor Surveillance System (BRFSS)
COVID Health Consequences	Caregiver screen time	✓	Behavioral Risk Factor Surveillance System (BRFSS)
COVID Health Consequences	Caregiver sleep	✓	Behavioral Risk Factor Surveillance System (BRFSS)
Social Determinants Of Health	Caregiver work	✓	
Social Determinants Of Health	Caregiver health insurance	✓	
Social Determinants Of Health	Caregiver health care utilization	✓	
Social Determinants Of Health	Caregiver positive childhood experiences (PCEs)		Positive Childhood Experiences (PCEs)
Social Determinants Of Health	Caregiver discrimination	✓	Everyday Discrimination Scale
Social Determinants Of Health	Caregiver social support	✓	RAND Social Support Survey
Caregiver wellbeing	Caregiver depressive symptoms	✓	Patient Health Questionnaire-9

Survey Instrument	Topic	Asked in Tier 2 follow-up surveys	Source of survey if not developed for RECOVER
Caregiver wellbeing	Caregiver anxiety symptoms	✓	Generalized Anxiety Disorder-7
Caregiver wellbeing	Caregiver stress	✓	Perceived Stress Scale
Caregiver wellbeing	Caregiver mental health	✓	DSM-5 Cross-Cutting Symptom Measure

20.5 Appendix E: Tiers 2 and 3 Core Data Elements

Table 15. Neurocognitive, Neurobehavioral, Well-Being and Mental Health Measures by Age in Tiers 2 and 3 for the Main Cohort

Study Tier	Neurocognitive and Developmental Assessments	Neurobehavioral, Well-Being and Mental Health Assessments
**Infancy and Toddlerhood:**	**Birth through 2 years old**	
Tier 2	Ages and Stages Questionnaire—3rd Edition (ASQ-3)<br>Modified Checklist for Autism in Toddlers Revised with Follow up (MCHAT-RF)	Ages and Stages Questionnaires: Social-Emotional, 2nd Edition (ASQ:SE-2)<br>Child Behavior Checklist
Tier 3	N/A	N/A
Preschool-Age:	3 years old through 5 years old	
Tier 2	Ages and Stages Questionnaire—3rd Edition (ASQ-3)<br>NIH Toolbox Cognitive Measures	Ages and Stages Questionnaires: Social-Emotional, 2nd Edition (ASQ:SE-2)<br>Child Behavior Checklist<br>Patient-Reported Outcomes Measurement Information System (PROMIS®) Parent Proxy Anger Scale<br>PROMIS® Parent Proxy Psychological Stress Experiences Scale<br>PROMIS® Parent Proxy Positive Affect Scale
Tier 3	*Cognitive*: Woodcock Johnson Cognitive Battery subtests<br>*Language*: Woodcock-Johnson Oral Language Battery subtests<br>*Verbal Memory*: Woodcock Johnson subtests<br>*Visual Memory*: Wide Range Assessment of Memory and Learning<br>*Visual-Motor Drawing*: Beery Buktenica<br>*Visual Motor Speed*: Purdue Pegboard<br>*Pre-Academics*: Woodcock-Johnson Achievement Battery subtests	Kiddie SADS computer completed by caregiver
School-age and Adolescence:	6 years old through 17 years old	
Tier 2	NIH Toolbox Cognitive Measures	Patient-Reported Outcomes Measurement Information System (PROMIS®) Parent Proxy Anger Scale<br>PROMIS® Parent Proxy Psychological Stress Experiences Scale<br>PROMIS® Parent Proxy Positive Affect Scale<br>Revised Children's Anxiety and Depression Scale (RCADS-25)<br>Strengths and Difficulties Questionnaire (Hyperactivity/Inattention and Conduct Problems Subscales)

**Study Tier**	**Neurocognitive and Developmental Assessments**	**Neurobehavioral, Well-Being and Mental Health Assessments**
Tier 3	*Cognitive*: Woodcock Johnson Cognitive Battery subtests<br>*Language*: Woodcock-Johnson Oral Language Battery subtests<br>*Verbal Memory*: Woodcock Johnson subtests<br>*Visual Memory*: Wide Range Assessment of Memory and Learning<br>*Visual-Motor Drawing*: Beery Buktenica<br>*Visual Motor Speed*: Purdue Pegboard<br>*Pre-Academics*: Woodcock-Johnson Achievement Battery subtests	Kiddie SADS computer completed by caregiver
Young Adults:	18 years through 25 years old	
Tier 2	NIH Toolbox Cognitive Measures	Patient-Reported Outcomes Measurement Information System (PROMIS®) Parent Proxy Anger Scale<br>PROMIS® Parent Proxy Psychological Stress Experiences Scale<br>Achenbach Adult Self Report
Tier 3	*Cognitive*: Woodcock Johnson Cognitive Battery subtests<br>*Language*: Woodcock-Johnson Oral Language Battery subtests<br>*Verbal Memory*: Woodcock Johnson subtests<br>*Visual Memory*: Wide Range Assessment of Memory and Learning<br>*Visual-Motor Drawing*: Beery Buktenica<br>*Visual Motor Speed*: Purdue Pegboard	SADS Structured Psychiatric Interview

20.6 Appendix F: Clinical and Laboratory Assessments

Table 16: Clinical and Laboratory Assessments Across the Tiers in the Main Cohort RECOVER-Pediatrics Cohort.

Category	Assessment	Tier 1	Tier 2	Tier 3
Clinical Assessment	Weight		✓	
Clinical Assessment	Height or length		✓	
Clinical Assessment	Waist circumference		✓	
Clinical Assessment	Skin fold thickness (triceps and subscapular)		✓	
Clinical Assessment	Temperature		✓	
Clinical Assessment	Heart rate		✓	
Clinical Assessment	Respiratory rate		✓	
Clinical Assessment	Oxygen saturation		✓	
Clinical Assessment	Blood pressure		✓	
Clinical Assessment	10 Minute Active Standing Test (Assessing blood pressure and heart rate after 5 minutes supine, then after standing for 1, 3, 5, 7 and 10 minutes)		✓	
Clinical Assessment	Electrocardiogram		✓	
Clinical Assessment	Spirometry		✓	
Clinical Assessment	Beighton Scale for Joint Hypermobility		✓	
Clinical Assessment	NIH toolbox		✓	
Clinical Assessment	Echocardiogram			✓
Clinical Assessment	Cardiac MRI without contrast			✓
Clinical Assessment	Pulmonary Function Tests (PFTs)			✓
Clinical Assessment	Lung Microbiome (Sputum Induction)			✓
Clinical Assessment	Cardiopulmonary Exercise Testing			✓
Clinical Assessment	Abdominal ultrasound			✓
Clinical Assessment	Brain MRI without contrast			✓
Clinical Assessment	Brain EEG			✓
Clinical Assessment	Neurocognitive testing			✓
Laboratory study	SARS-CoV-2 spike and nucleocapsid antibody **(Central)**	✓	✓	
Laboratory study	Complete metabolic panel **(Local)**		✓	
Laboratory study	Complete blood count **(Local)**		✓	
Laboratory study	Anti nuclear antibody (ANA) **(Central)**		✓	
Laboratory study	Anti-cyclic citrullinated peptide antibodies (Anti-CCP) **(Central)**		✓	
Laboratory study	Anti dsDNA antibody **(Central)**		✓	
Laboratory study	Rheumatoid factor (RF) **(Central)**		✓	
Laboratory study	Lipid Panel **(Local)**		✓	
Laboratory study	Hemoglobin A1c **(Local)**		✓	
Laboratory study	Thyroid stimulating hormone (TSH) **(Local)**		✓	
Laboratory study	Free T4 **(Local)**		✓	
Laboratory study	25-hydroxyvitamin D **(Local)**		✓	
Laboratory study	Serum calcium **(Local)**		✓	

Category	Assessment	Tier 1	Tier 2	Tier 3
Laboratory study	EBV anti early antigen IgG, viral capsid IgM, viral capsid IgG, nuclear antigen IgG **(Central)**		✓	
Laboratory study	D-Dimer			✓
Laboratory study	High sensitivity Troponin			✓
Laboratory study	High sensitivity C-reactive protein			✓
Laboratory study	Procalcitonin			✓
Laboratory study	N-terminal pro-brain natriuretic peptide			✓
Laboratory study	Insulin C-peptide			✓
Laboratory study	Microbiome specimens: sputum, skin swabs, nasal swabs, oral swabs, urine and stool			✓

21 References

1. Worldometer. Coronavirus Cases. 2021.
2. Carbone M, Lednicky J, Xiao S-Y, Venditti M, Bucci E. Coronavirus 2019 Infectious Disease Epidemic: Where We Are, What Can Be Done and Hope For. J Thorac Oncol 2021;16:546-571.
3. Nasserie T, Hittle M, Goodman SN. Assessment of the Frequency and Variety of Persistent Symptoms Among Patients With COVID-19: A Systematic Review. JAMA Network Open 2021;4:e2111417-e2111417.
4. Higgins V, Sohaei D, Diamandis EP, Prassas I. COVID-19: from an acute to chronic disease? Potential long-term health consequences. Critical Reviews in Clinical Laboratory Sciences 2020:1-23.
5. Sah P, Fitzpatrick MC, Zimmer CF et al. Asymptomatic SARS-CoV-2 infection: A systematic review and meta-analysis. Proc Natl Acad Sci U S A 2021;118.
6. Wiersinga WJ, Rhodes A, Cheng AC, Peacock SJ, Prescott HC. Pathophysiology, Transmission, Diagnosis, and Treatment of Coronavirus Disease 2019 (COVID-19): A Review. JAMA 2020;324:782-793.
7. Gupta A, Madhavan MV, Sehgal K et al. Extrapulmonary manifestations of COVID-19. Nature Medicine 2020;26:1017-1032.
8. Nalbandian A, Sehgal K, Gupta A et al. Post-acute COVID-19 syndrome. Nature Medicine 2021;27:601-615.
9. Wadman M, Couzin-Frankel J, Kaiser J, Matacic C. A rampage through the body. Science (New York, NY) 2020;368:356-360.
10. Barth RF, Xu X, Buja LM. A Call to Action: The Need for Autopsies to Determine the Full Extent of Organ Involvement Associated With COVID-19. Chest 2020;158:43-44.
11. Hannah E. Davis GSA, Lisa McCorkell, Hannah Wei, Ryan J. Low, Yochai Re'em, Signe Redfield, Jared P. Austin, Athena Akrami. Characterizing Long COVID in an International Cohort: 7 Months of Symptoms and Their Impact. medRxiv 2020.
12. WHO. A clinical case definition of post COVID-19 condition by a Delphi consensus, 6 October 2021. 2021.
13. Dionne A, Sperotto F, Chamberlain S et al. Association of Myocarditis With BNT162b2 Messenger RNA COVID-19 Vaccine in a Case Series of Children. JAMA Cardiol 2021.
14. Mevorach D, Anis E, Cedar N et al. Myocarditis after BNT162b2 mRNA Vaccine against Covid-19 in Israel. N Engl J Med 2021.
15. Al-Aly Z, Xie Y, Bowe B. High-dimensional characterization of post-acute sequelae of COVID-19. Nature 2021;594:259-264.
16. Dani M, Dirksen A, Taraborrelli P et al. Autonomic dysfunction in 'long COVID': rationale, physiology and management strategies. Clinical medicine (London, England) 2021;21:e63-e67.

17. Davis HE, Assaf GS, McCorkell L et al. Characterizing long COVID in an international cohort: 7 months of symptoms and their impact. EClinicalMedicine 2021;38:101019.

18. Hayes LD, Ingram J, Sculthorpe NF. More Than 100 Persistent Symptoms of SARS-CoV-2 (Long COVID): A Scoping Review. Front Med (Lausanne) 2021;8:750378.

19. Group TWCftCS. Four-Month Clinical Status of a Cohort of Patients After Hospitalization for COVID-19. JAMA 2021;325:1525-1534.

20. Buonsenso D, Munblit D, De Rose C et al. Preliminary evidence on long COVID in children. Acta Paediatr 2021;110:2208-2211.

21. Sterky E, Olsson-Akefeldt S, Hertting O et al. Persistent symptoms in Swedish children after hospitalisation due to COVID-19. Acta Paediatr 2021;110:2578-2580.

22. Osmanov IM, Spiridonova E, Bobkova P et al. Risk factors for long covid in previously hospitalised children using the ISARIC Global follow-up protocol: A prospective cohort study. Eur Respir J 2021.

23. Molteni E, Sudre CH, Canas LS et al. Illness duration and symptom profile in symptomatic UK school-aged children tested for SARS-CoV-2. Lancet Child Adolesc Health 2021.

24. Radtke T, Ulyte A, Puhan MA, Kriemler S. Long-term Symptoms After SARS-CoV-2 Infection in Children and Adolescents. JAMA 2021.

25. Roessler M, Tesch F, Batram M et al. Post COVID-19 in children, adolescents, and adults: results of a matched cohort study including more than 150,000 individuals with COVID-19. medRxiv 2021:2021.10.21.21265133.

26. AAP: https://www.aap.org/en/pages/2019-novel-coronavirus-covid-19-infections/children-and-covid-19-state-level-data-report/. 2021.

27. Stokes EK ZL, Anderson KN, et al. Coronavirus Disease 2019 Case Surveillance — United States,. MMWR Morb Mortal Wkly Rep 2020;69:759–765 CDC, January 22–May 30, 2020.

28. CDC. United States COVID-19 Cases, Deaths, and Laboratory Testing (NAATs) by State, Territory, and Jurisdiction. COVID cases, Deaths and Testing, June 26th 2021.

29. Grant LR, Hammitt LL, Murdoch DR, O'Brien KL, Scott JA. Procedures for collection of induced sputum specimens from children. Clin Infect Dis 2012;54 Suppl 2:S140-5.

30. Pizzichini E, Pizzichini MM, Leigh R, Djukanovic R, Sterk PJ. Safety of sputum induction. Eur Respir J Suppl 2002;37:9s-18s.

31. Lindquist DE, Cooper AA. Safety of Levalbuterol Compared to Albuterol in Patients With a Tachyarrhythmia. Journal of Pharmacy Technology 2014;30:13-17.

32. Paridon SM, Alpert BS, Boas SR et al. Clinical stress testing in the pediatric age group: a statement from the American Heart Association Council on Cardiovascular Disease in the Young, Committee on Atherosclerosis, Hypertension, and Obesity in Youth. Circulation 2006;113:1905-20.

33. Fabsitz RR, McGuire A, Sharp RR et al. Ethical and practical guidelines for reporting genetic research results to study participants: updated guidelines from a National

Heart, Lung, and Blood Institute working group. Circ Cardiovasc Genet 2010;3:574-80.

34. Richards CS, Bale S, Bellissimo DB et al. ACMG recommendations for standards for interpretation and reporting of sequence variations: Revisions 2007. Genet Med 2008;10:294-300.

35. Richards S, Aziz N, Bale S et al. Standards and guidelines for the interpretation of sequence variants: a joint consensus recommendation of the American College of Medical Genetics and Genomics and the Association for Molecular Pathology. Genet Med 2015;17:405-24.

36. CDC: https://www.cdc.gov/mis/mis-c/hcp/index.html?CDC_AA_refVal=https%3A%2F%2Fwww.cdc.gov%2Fmis%2Fhcp%2Findex.html.
