# Supplementary material for: Researching COVID to enhance recovery (RECOVER) pediatric study protocol: Rationale, objectives and design
Source: PLoS One. 2024 May 7;19(5):e0285635. doi: 10.1371/journal.pone.0285635 (PMC11075869; doi:10.1371/journal.pone.0285635)
Supplement: S2 Text — (PDF) [file pone.0285635.s012.pdf]

ASSENT FORM (ages 12-14 years – Part 1)

Short Title of the Research Study: Understanding the long-term impact of COVID on children and families

Why we would like to speak with you

We want to talk with you about being a part of a research study. A research study is when people try to get information to learn more about solving a problem. The people who do this are called researchers.

Before saying yes to being in this research study, it is important that you read this form and speak with the researchers about the study. This form tells you why we are doing the research, what you will be doing in this research study, and what the risks may be. It also tells you about your right to say "No" to being a part of this research study.

Before you can be in this research study, we will to tell you about what you will be doing in this research study. You can ask questions at any time before, during or after we talk. You will also be given time to read this form. After we tell you about this research study, we will ask you if you would like to be in this research study or not. If you decide to be in this research study, you will be asked to sign this paper and you can take a copy of it home with you.

This research study is not about getting a medicine or another treatment. Your regular doctor is in charge of helping you take care of your health, whether or not you agree to be part of this research study.

It's okay to say 'No' if you don't want to be in the study. It is also okay to be in the study now and leave it at any time. You should speak with your parent or person who takes care of you (guardian) about what you decide.

Why are we doing this research study?

We are doing this research study to learn more about children who have had COVID-19 or Coronavirus. COVID-19 is caused by an infection with a virus called SARS CoV-2. The reason we are doing this study is to understand why some children who got COVID-19 are still sick many months after being infected, and how this affects the body. This is sometimes called "Long COVID."

As part of the study, we would like to get information from you and your family, and put that together with information from other children and families across the country. This will make it easier for us to understand:

- *How many* children and families are getting Long COVID?
- *Why* some children and families are getting Long COVID?
- *How long* does Long COVID last in children?
- *What can be done to help* children and families with Long COVID?

We will be getting information from lots of children like you. In this research study, there will be about 20,000 children.

What will happen to you if you are in the research study?

There are a few different parts of the study. We will only talk about the first part of the study today. If you are asked to participate in the other parts of the research study, we will tell you more about the other parts, and we will ask you if you would like to be the other parts or not.

This is what you will be asked to do for the first part of the study:

- Answer questions about you and your home
- Have your blood and saliva (spit) taken at your home

The first part of the study can be done at home and will take about 1 hour. Here is more information about what we will ask you to do:

Approved For Period: 12/4/2022 - 8/31/2023

Answer questions. You, your parent or the person who cares for you will answer questions about their health and your health. We will ask about:

- any medications you take
- your health history
- any testing you have done for COVID-19 and if you have gotten the COVID-19 vaccine
- how COVID-19 may have affected you, and whether you are still having any health problems because of past infection with the virus that causes COVID-19

Have your blood and saliva (spit) taken. Your parent or guardian will help collect the blood at home with a small device and collect saliva by having you spit into a tube. We will do tests on your blood and saliva. One of the tests is for antibodies to the virus that causes COVID. Antibodies are made by your body to help you get rid of germs. We will call your parent or guardian to explain the results of your tests. Depending on the test results, you may be asked to visit the research study center (hospital or office building) to repeat the antibody blood the test after 3 and 6 months.

We will keep some of your blood and saliva to test later on. You can choose to learn the results of these tests in a few years when we are done with this study.

What side effects might happen while you are on the research study?

Th needle used to get blood will pinch your skin and can hurt for a little while, just like getting a blood test or a shot at the doctor. If you feel sick or hurt, it is very important that you tell your parents and the researchers.

What are your other choices?

If you choose not to be in this research study, it won’t affect the type of regular treatment you will receive. Your doctor will recommend that treatment for you. You will get treatment for COVID-19 or any other health problem, whether or not you decide to be in this study. We will let your doctor know if you are in the study, and they will make sure you get the best treatments for what you need.

Do you have to be in **the research study?**

No, and no one will be upset with you if you do not want to be in this research study. If you don’t want to be in this research study, just tell us. If you do want to be in the research study, tell us that. And, remember, you can say yes now and change your mind later. It’s up to you.

Please talk this over with your parents or guardian before you decide whether or not to be in the research study. Your parents have said that it is okay with them if you want to be in the research study. Even though your parents have said it is okay with them, you can still say 'No'.

What else do you need to know?

The results of this research may be presented at medical meetings or in research study publications, but your name would never be used. Research study records with your name or other information about you will be kept confidential or private as required by law.

What about your privacy?

The researchers will talk about you and the research study with your parent/guardian, but will not talk about it with anyone else except the people working on the study and your doctor.

What will this cost?

There is no cost to you or your parents for being in this research study. Your parents or legal guardian will receive money for your time spent doing this research study.

What if you have questions?

You can ask us questions at any time. You can ask now or later. Your parent or guardian knows how to reach us even after you go back home. If you have any questions about your rights while in the research study, you may

Approved For Period: 12/4/2022 - 8/31/2023

call the New York University Grossman School of Medicine Institutional Review Board (IRB) at (212) 263-4110. The IRB is a department at NYU that reviews research studies to protect your rights and monitor your safety and concerns.

Agreeing to participate in this research study

You can say yes or no. If you say yes, remember:

- You can stop being in the study any time you want to
- You can call the researcher any time you have any questions
- Besides your parents/guardian, your information will only be shared with the people working on this study and your doctor

If you sign this paper, it means that

- you have read this form
- you have talked with the research team and your parents/guardian about it
- you have had all your questions answered and
- you want to be in the research study

By signing below you are agreeing to participate in part 1 of this research study and you will receive a signed copy of this form.

____

Signature of Child *Date*

____

Signature of Person Getting Assent/Consent *Date*

Approved For Period: 12/4/2022 - 8/31/2023
