# Supplementary material for: Researching COVID to enhance recovery (RECOVER) pediatric study protocol: Rationale, objectives and design
Source: PLoS One. 2024 May 7;19(5):e0285635. doi: 10.1371/journal.pone.0285635 (PMC11075869; doi:10.1371/journal.pone.0285635)
Supplement: S5 Text — (PDF) [file pone.0285635.s015.pdf]

ASSENT FORM (ages 12-14 years – Part 2)

Short Title of the Research Study: Understanding the long-term impact of COVID on children and families

Why we would like to speak with you

Thank you for being in our study so far. When we last talked about the study, we told you that this study has other parts. There are three parts to this study. You have finished the first part of the study, and we would like to tell you about the second part of the study.

We are doing a research study to understand child health problems that come from a virus called COVID-19. A virus is a kind of germ that can make children feel sick. After we tell you about the next part of the research study, we will ask you if you would like to be in this second part of the research study or not. If you decide to be in this second part of the research study, you will be asked to sign this paper and you can take a copy of it home with you.

If you have any questions as we are talking about this research study, you can ask them. Don't worry about waiting until the end. You can stop me at any time and ask your questions. It's okay to say 'NO' if you don't want to be in the study. It is also okay to be in the study now and leave it at any time. You should talk with your parent or person who takes care of you (guardian) about what you decide.

This research study is not about getting a medicine or another treatment. Your regular doctor is in charge of helping you take care of your health, whether or not you agree to be part of this research study.

Why are we doing this research study**?**

The reason we are doing this study is to understand why some children who get COVID-19 feel better fast and other children who get COVID-19 feel sick for a long time. Feeling sick for a long time after COVID-19 is sometimes called "Long COVID."

As part of the study, we would like to get information from you and your family, and put that together with information from other children and families across the country. This will make it easier for us to understand:

- *How many* children and families are getting Long COVID?
- *Why* some children and families are getting Long COVID?
- *How long* does Long COVID last in children?
- *What can be done to help* children and families with Long COVID?

We will be getting information from lots of children like you. In part 2 of this research study, there will be about 10,000 children.

What will happen to you if you are in the research study?

In part 1 of the study, you were asked to do these things at home:

- Answering questions
- Having your blood and saliva (spit) taken

For part 2 of the study, some of the things you will be asked to do are done at home and some of the things that you will be asked to do are done at the research study center (the office where the researchers work). Being in part 2 of the study can take 2-4 years with up to 6 visits to the research study center.

If you agree to be in part 2 of the study, you will be asked to:

- **Answer questions.** Some of the questions will be similar to the questions you answered in part 1 and can be done at home. You will also have some new questions in part 2 about how you feel, and how you think and learn. Some of these new questions will be done at the research study center.

Approved For Period: 12/4/2022 - 8/31/2023

- **Have tests at the research study center.** The tests will include a check-up to measure how you are growing, and may also include tests to measure how your heart is working (called an electrocardiogram or electrical activity of the heart) and how your lungs are working (called a spirometry or pulmonary function test). These tests may not be done at all visits, or in all children in the study. The researchers will tell you if you will have these tests.
- **Have tests of blood and urine (pee).** The researchers will use a needle to collect blood from your arm and ask you to pee in a cup to collect urine. These tests may not be done at all visits, or in all children in the study. The researchers will tell you if you will have these tests.

Some of the blood will be used to test for the COVID-19 antibody, just like part 1. We will keep some of your blood and pee to test later on. You can choose to learn the results of these tests in a few years when we are done with this study.

Will it hurt?

When your blood is taken, it might hurt. That is because the needle will pinch your skin, just like getting a blood test or a shot at the doctor. If you feel sick or hurt, it is very important that you tell your parents and the researchers.

Do you have to be in this research study?

No, and no one will be upset with you if you do not want to be in this research study. If you don't want to be in this research study, just tell us. If you do want to be in the research study, tell us that. And, remember, you can say yes now and change your mind later. It's up to you.

Please talk this over with your parents or guardian before you decide whether or not to be in the research study. Your parents have said that it is okay with them if you want to be in the research study. Even though your parents have said it is okay with them, you can still say 'No'.

What if you have questions?

You can ask us questions at any time. You can ask now or later. Your parent or guardian knows how to reach us even after you go back home.

What about your privacy?

The researchers will talk about you and the research study with your parent/guardian, but will not talk about it with anyone else except the people working on the study and your doctor.

Saying Yes or No to being in this research study

You can say yes or no. If you say yes, remember:

1. You can stop being in the study any time you want to
2. You can call the researcher any time you have any questions
3. Besides your parents/guardian, your information will only be shared with the people working on this study and your doctor

If you sign this paper, it means that you have read this and you have talked with the researchers and your parents/guardian about it. It also means you have had all your questions for today answered and you want to be in part 2 of the research study.

If you do not want to be in the study, do not sign this paper.

Being in the study is up to you, and no one will be upset if you don't sign this paper or if you change your mind later.

Approved For Period: 12/4/2022 - 8/31/2023

____
Signature of Child *Date*

____
Signature of Person Getting Assent/Consent *Date*

Approved For Period: 12/4/2022 - 8/31/2023
