# Supplementary material for: Researching COVID to enhance recovery (RECOVER) pediatric study protocol: Rationale, objectives and design
Source: PLoS One. 2024 May 7;19(5):e0285635. doi: 10.1371/journal.pone.0285635 (PMC11075869; doi:10.1371/journal.pone.0285635)
Supplement: S6 Text — (PDF) [file pone.0285635.s016.pdf]

ASSENT FORM (ages 15-17 years – Part 2)

Short Title of the Research Study: Understanding the long-term impact of COVID on children and families

Agreeing to participate in this research study

Thank you for being in our study so far. When we last talked about the study, we told you that this study has other parts. There are three parts to this study. You have finished the first part of the study, and we would like to tell you about the second part of the study.

Your parent or person who cares for you (guardian) knows about this part of our study and wants you to be in part 2 of the study if you agree. If you sign below it means that you are saying yes and do want to be in part 2 of the study. If you are asked to be in the other parts of the study you will be asked to sign another one of these forms in the future.

You can say yes or no. If you say yes, remember:

- You can stop being in the study any time you want to
- You can call the researcher any time you have any questions
- Besides your parents/guardian, your information will only be shared with the people working on this study and your doctor

If you sign this paper, it means that

- you have read this form and the informed consent form
- you have talked with the research team and your parents/guardian about it
- you have had all your questions answered and
- you want to be in the research study

By signing below you are agreeing to participate in part 2 of this research study and you will receive a signed copy of this form.

____

Signature of Child *Date*

____

Signature of Person Getting Assent/Consent *Date*

Approved For Period: 12/4/2022 - 8/31/2023
