# Supplementary material for: Researching COVID to enhance recovery (RECOVER) pediatric study protocol: Rationale, objectives and design
Source: PLoS One. 2024 May 7;19(5):e0285635. doi: 10.1371/journal.pone.0285635 (PMC11075869; doi:10.1371/journal.pone.0285635)
Supplement: S3 File — (PDF) [file pone.0285635.s019.pdf]

NYU Grossman School of Medicine

Rachel S. Gross, MD, MS
Associate Professor of Pediatrics and Population Health
Pediatric Research Director, RECOVER Clinical Science Core

Dear Editorial Team,

Re:

MS ID#: PONE-D-23-10495R1

MS Title: Researching COVID to enhance recovery (RECOVER) pediatric study protocol: Rationale, objectives and design

The following individuals should be recognized as co-authors on this manuscript, we kindly request for them to be added to the author list.

Tamara Bradford
Maryanne Chrisant
Audrey Dionne
Stephanie Handler
Keren Hasbani
Camden Hebson
Kimberly McHugh
Julie Miller
Elizabeth C. Mitchell
Onyekachukwu Osakwe
Michael A. Portman
S. Kristen Sexson Tejtel
Shubika Srivastava
Felicia Trachtenberg

Kind Regards,

____

Rachel Gross, MD, MS

Corresponding Author

NYU RECOVER Clinical Science Core
462 First Avenue, 8th Floor New York, NY 10016 | Rachel.Gross@nyulangone.org

NYU Grossman School of Medicine

Co-Authors

Author	Signature	Date Signed
Shifa Ahmed	Shifa Ahmed	2/16/24
Almary Akerlundh	Almary Akerlundh	2/16/24
Akram N. Alshawabkeh	Akram N. Alshawabkeh	2/16/24
Brett R. Anderson	Brett R. Anderson	2/16/24
Judy L. Aschner	Judy L. Aschner	2/16/24
Andew M. Atz	Andew M. Atz	2/16/24
Robin L. Aupperle	Robin L. Aupperle	2/16/24
Fiona C. Baker	Fiona C. Baker	2/16/24
Venkataraman Balaraman	Venkataraman Balaraman	2/16/24
Dithi Banerjee	Dithi Banerjee	2/16/24
Deanna M. Barch	Deanna M. Barch	2/16/24
Arielle Baskin-Sommers	Arielle Baskin-Sommers	2/16/24
Sultana Bhuiyan	Sultana Bhuiyan	2/16/24
Marie-Abele C. Bind	Marie-Abele C. Bind	2/16/24
Amanda L. Bogie	Amanda L. Bogie	2/16/24
Tamara Bradford	Tamara Bradford	2/16/24
Natalie C. Buchbinder	Natalie C. Buchbinder	2/16/24
Elliott Bueler	Elliott Bueler	2/16/24

NYU Grossman School of Medicine

Author	Signature	Date Signed
Hülya Bükülmez	Hülya Bükülmez	2/16/24
B.J. Casey	B.J. Casey	2/16/24
James Chan	James Chan	2/16/24
Linda Chang	Linda Chang	2/16/24
Lori B. Chibnik	Lori B. Chibnik	2/16/24
Maryanne Chrisant	Maryanne Chrisant	2/16/24
Mine S. Cicek	Mine S. Cicek	2/16/24
Duncan B. Clark	Duncan B. Clark	2/16/24
Rebecca G. Cliffton	Rebecca G. Cliffton	2/16/24
Katharine N. Clouser	Katharine N. Clouser	2/16/24
Lesley Cottrell	Lesley Cottrell	2/16/24
Kelly Cowan	Kelly Cowan	2/16/24
Viren D'Sa	Viren D'Sa	2/16/24
Mirella Dapretto	Mirella Dapretto	2/16/24
Soham Dasgupta	Soham Dasgupta	2/16/24
Walter Dehority	Walter Dehority	2/16/24
Audrey Dionne	Audrey Dionne	2/16/24
Benard P. Dreyer	Benard P. Dreyer	2/16/24
Kirsten B. Dummer	Kirsten B. Dummer	2/16/24

Author	Signature	Date Signed
Matthew D. Elias	Matthew D. Elias	2/16/24
Amy J. Elliott	Amy J. Elliott	2/16/24
Shari Esquenazi-Karonika	Shari Esquenazi-Karonika	2/16/24
Danielle N. Evans	Danielle N. Evans	2/16/24
E. Vincent S. Faustino	E. Vincent S. Faustino	2/16/24
Alexander G. Fiks	Alexander G. Fiks	2/16/24
Valerie J. Flaherman	Valerie J. Flaherman	2/16/24
Daniel Forsha	Daniel Forsha	2/16/24
Andrea S. Foulkes	Andrea S. Foulkes	2/16/24
John J. Foxe	John J. Foxe	2/16/24
Naomi P. Friedman	Naomi P. Friedman	2/16/24
Greta Fry	Greta Fry	2/16/24
Margot Gage Witvliet	Margot Gage Witvliet	2/16/24
Richard Gallagher	Richard Gallagher	2/16/24
Sunanda Gaur	Sunanda Gaur	2/16/24
Dylan G. Gee	Dylan G. Gee	2/16/24
Maria Laura Gennaro	Maria Laura Gennaro	2/16/24
Kevin M. Gray	Kevin M. Gray	2/16/24
Rachel S. Gross	Rachel S. Gross	2/15/24

NYU Grossman School of Medicine

Author	Signature	Date Signed
Stephanie Handler	Stephanie Handler	2/16/24
Ashraf S. Harahsheh	Ashraf S. Harahsheh	2/16/24
Keren Hasbani	Keren Hasbani	2/16/24
Andrew C. Heath	Andrew C. Heath	2/16/24
Camden Hebson	Camden Hebson	2/16/24
Mary M. Heitzeg, Ph.D.	Mary M. Heitzeg, Ph.D.	2/16/24
Christina M. Hester	Christina M. Hester	2/16/24
Sophia Hill	Sophia Hill	2/16/24
Laura Hobart-Porter	Laura Hobart-Porter	2/16/24
Travis K.F. Hong	Travis K.F. Hong	2/16/24
Carol R. Horowitz	Carol R. Horowitz	2/16/24
Daniel S. Hsia	Daniel S. Hsia	2/16/24
Matt Huentelman	Matt Huentelman	2/16/24
Kathy D. Hummel	Kathy D. Hummel	2/16/24
Katherine Irby	Katherine Irby	2/16/24
Joanna Jacobus	Joanna Jacobus	2/16/24
Vanessa L. Jacoby	Vanessa L. Jacoby	2/16/24
Terry L. Jernigan	Terry L. Jernigan	2/16/24
Pei-Ni Jone	Pei-Ni Jone	2/16/24

NYU Grossman School of Medicine

Author	Signature	Date Signed
David C. Kaelber	David C. Kaelber	2/16/24
Elizabeth W. Karlson	Elizabeth W. Karlson	2/16/24
Tyler J. Kasmarcak	Tyler J. Kasmarcak	2/16/24
Stuart D. Katz	Stuart D. Katz	2/15/24
Patricia A. Kinser	Patricia A. Kinser	2/16/24
Lawrence C. Kleinman	Lawrence C. Kleinman	2/16/24
Matthew J. Kluko	Matthew J. Kluko	2/16/24
Jessica S. Kosut	Jessica S. Kosut	2/16/24
Angela R. Laird	Angela R. Laird	2/16/24
Michelle F. Lamendola-Essel	Michelle F. Lamendola-Essel	2/16/24
Jeremy Landeo-Gutierrez	Jeremy Landeo-Gutierrez	2/16/24
Sean M. Lang	Sean M. Lang	2/16/24
Christine L. Larson	Christine L. Larson	2/16/24
Peter Paul C. Lim	Peter Paul C. Lim	2/16/24
Krista M. Lisdahl	Krista M. Lisdahl	2/16/24
Brian W. McCrindle	Brian W. McCrindle	2/16/24
Russell J. McCulloh	Russell J. McCulloh	2/16/24
Kimberly McHugh	Kimberly McHugh	2/16/24
Alan L. Mendelsohn	Alan L. Mendelsohn	2/16/24

NYU Grossman School of Medicine

Author	Signature	Date Signed
Torri D. Metz	Torri D. Metz	2/16/24
Julie Miller	Julie Miller	2/16/24
Elizabeth C. Mitchell	Elizabeth C. Mitchell	2/16/24
Joshua D. Milner	Joshua D. Milner	2/16/24
Sindhu Mohandas	Sindhu Mohandas	2/16/24
Lerraughn M. Morgan	Lerraughn M. Morgan	2/16/24
Praveen C. Mudumbi	Praveen C. Mudumbi	2/15/24
Eva M. Müller-Oehring	Eva M. Müller-Oehring	2/16/24
Erica R. Nahin	Erica R. Nahin	2/16/24
Michael C. Neale	Michael C. Neale	2/16/24
Manette Ness-Cochinwala	Manette Ness-Cochinwala	2/16/24
Jane W. Newburger	Jane W. Newburger	2/16/24
Sheila M. Nolan	Sheila M. Nolan	2/16/24
Carlos R. Oliveira	Carlos R. Oliveira	2/16/24
Onyekachukwu Osakwe	Onyekachukwu Osakwe	2/16/24
Matthew E. Oster	Matthew E. Oster	2/16/24
R. Mark Payne	R. Mark Payne	2/16/24
Michael A. Portman	Michael A. Portman	2/16/24
Hengameh Raissy	Hengameh Raissy	2/16/24

NYU Grossman School of Medicine

Author	Signature	Date Signed
Isabelle G. Randall	Isabelle G. Randall	2/16/24
Suchitra Rao	Suchitra Rao	2/16/24
Harrison T. Reeder	Harrison T. Reeder	2/16/24
Kyung E. Rhee	Kyung E. Rhee	2/16/24
Johana M. Rosas	Johana M. Rosas	2/16/24
Erika B. Rosenzweig	Erika B. Rosenzweig	2/16/24
Mark W. Russell	Mark W. Russell	2/16/24
Arash A. Sabati	Arash A. Sabati	2/16/24
Amy L. Salisbury	Amy L. Salisbury	2/16/24
Yamuna Sanil	Yamuna Sanil	2/16/24
Alice I. Sato	Alice I. Sato	2/16/24
Michael S. Schechter	Michael S. Schechter	2/16/24
Rangaraj Selvarangan	Rangaraj Selvarangan	2/16/24
S. Kristen Sexson Tejtel	S. Kristen Sexson Tejtel	2/16/24
Divya Shakti	Divya Shakti	2/16/24
Kavita Sharma	Kavita Sharma	2/16/24
Jessica N. Snowden	Jessica N. Snowden	2/16/24
Lindsay M. Squeglia	Lindsay M. Squeglia	2/16/24
Shubhika Srivastava	Shubhika Srivastava	2/16/24

Author	Signature	Date Signed
Cheryl R. Stein	Cheryl R. Stein	2/16/24
Michelle D. Stevenson	Michelle D. Stevenson	2/16/24
Melissa S. Stockwell	Melissa S. Stockwell	2/16/24
Jackie Szmuszkovicz	Jackie Szmuszkovicz	2/16/24
Maria M. Talavera-Barber	Maria M. Talavera-Barber	2/16/24
Kelan G. Tantisira	Kelan G. Tantisira	2/16/24
Ronald J. Teufel, II	Ronald J. Teufel, II	2/16/24
Deepika Thacker	Deepika Thacker	2/16/24
Felicia Trachtenberg	Felicia Trachtenberg	2/16/24
Tanayott Thaweethai	Tanayott Thaweethai	2/16/24
Moriah E. Thomason	Moriah E. Thomason	2/16/24
Dongngan T. Truong	Dongngan T. Truong	2/16/24
Mmekom M. Udosen	Mmekom M. Udosen	2/16/24
David Warburton	David Warburton	2/16/24
Megan R. Warner	Megan R. Warner	2/16/24
Sara E. Watson	Sara E. Watson	2/16/24
Alan Werzberger	Alan Werzberger	2/16/24
Jordan C. Weyer	Jordan C. Weyer	2/16/24
John C. Wood	John C. Wood	2/16/24

Author	Signature	Date Signed
Marion J. Wood	Marion J. Wood	2/15/24
H. Shonna Yin	H. Shonna Yin	2/16/24
William T. Zempsky	William T. Zempsky	2/16/24
Emily Zimmerman	Emily Zimmerman	2/16/24
