# Supplementary material for: Researching COVID to enhance recovery (RECOVER) pediatric study protocol: Rationale, objectives and design
Source: PLoS One. 2024 May 7;19(5):e0285635. doi: 10.1371/journal.pone.0285635 (PMC11075869; doi:10.1371/journal.pone.0285635)
Supplement: S5 File — (PDF) [file pone.0285635.s021.pdf]

[Insert logo here]

Research Subjects Ages 18 to 25 Years Old
Informed Consent Form (Parts 1 & 2)

**Title of Study:**<br><br><br><br>**Study Site Leader:**	NIH RECOVER: A Multi-site Observational Study of Post-Acute Sequelae of SARS-CoV-2 Infection in Pediatric Populations<br>Study #: s21-01231<br><br>Name<br>Title<br>Institution<br>Address 1<br>Address 2<br>City, State, Zip Code<br>Telephone
**Study Leader:**	Stuart Katz, MD, MS<br>Helen L. and Martin S. Kimmel Professor of Advanced Cardiac Therapeutics<br>NYU Grossman School of Medicine<br>530 First Avenue, Skirball 9R<br>New York, NY 10016<br>Tel 1-833-422-6819
**Research Information Leader:**	Andrea Foulkes, Sc.D.<br>Director Biostatistics, Massachusetts General Hospital<br>Professor of Medicine, Harvard Medical School<br>50 Staniford Street, Suite 560<br>Boston, MA 02114<br>Tel (617) 724-8208
**Emergency Contact:**	Insert Emergency Contact<br>Insert Phone Number/Pager, etc.
**For questions or concerns about the study, please call:**	Stuart Katz, MD, MS<br>Helen L. and Martin S. Kimmel Professor of Advanced Cardiac Therapeutics<br>NYU Grossman School of Medicine<br>530 First Avenue, Skirball 9R<br>New York, NY 10016<br>Tel 1-833-422-6819

1. About volunteering for this research study

You are being invited to take part in a research study. You can choose whether or not you want to take part in this study.

Before you decide if you want to be part of this study, you will need to know what the study is about, what you will have to do in this study, the good things that may come out of being part of the study, and the possible risks (or harms) from being in this study. You may also want to talk about this study and this form with your family, friends, or doctor. If you have any questions about the study or about this form, please ask us. If you decide to take part in this study, you must sign this form. We will give you a copy of this form to keep once you have signed it.

Approved For Period: 12/4/2022 - 8/31/2023

2. Why are we doing this study?

This study is part of a research project paid for by the National Institutes of Health (NIH) called RECOVER (Researching COVID to Enhance Recovery). We are doing this study to understand how COVID, the infection caused by a virus called SARS-CoV-2 affects the body, and why some people who got COVID are still sick many months after being infected. Taking a long time to recover from COVID is called “Long COVID” or “PASC”, which stands for Post-Acute Sequelae of SARS-CoV-2.

Children and young adults who have and have not had COVID will be part of the study. For some parts of the study, the main person taking care of these children or young adults, a parent or caregiver, is also being invited to take part in this study. We are asking you to take part in this research study to understand why some young adults with COVID-19 infection get more sick or have different parts of the body affected, or get better faster.

This consent form talks about the study visits for people from 18-25 years of age who will be in Parts 1 or 2 of the research study. An adult is someone who has reached the age of majority, as defined by the local regulations of your state. Once you reach the age of majority (become an adult), you will be asked to sign this consent form before doing parts 1 and 2 surveys and tests, even if you were already in the study before you reached the age of majority. “I”, “you” and “your” always refers to the “young adult” (the person who is taking part in the study).

3. How long will you be in the study? How many other people will be in the study?

We plan to have up to 20,000 children and young adults up to age 25 years old be part of this study across the country.

You are being asked to participate in Part 1 and Part 2 studies. There will be about 19,5000 children and young adults in Part 1 and about 10,000 children and young adults in Part 2.

4. What will you be asked to do in the study?

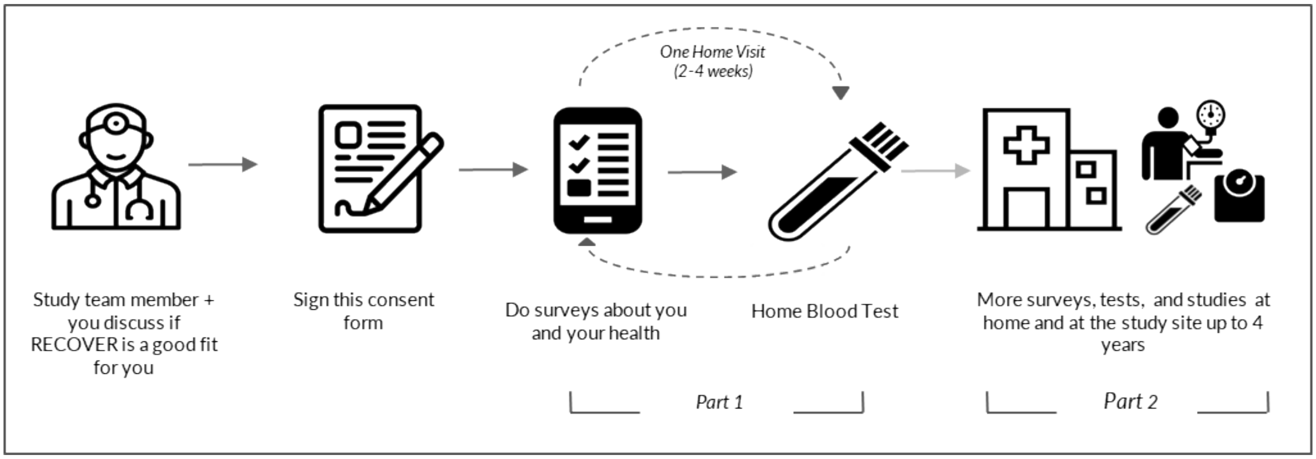

The picture shows the first two parts of the study. All the young adults who want to take part in the study will sign the consent form and get the Part 1 surveys and tests. Only about half of the young adults who do the Part 1 surveys and tests will be asked to do Part 2 surveys and tests. You can choose to be in only Part 1, and not be in Part 2. You are being asked to read and sign this consent because you have been asked to be in Part 1, and may be asked to be in Part 2. If you choose to be in this study, we will ask you to sign this consent form before any of the Part 1 or Part 2 surveys and tests.

Approved For Period: 12/4/2022 - 8/31/2023

Part 1 Surveys and Tests

Part 1 Surveys and Tests will be done at home. The surveys and tests will be done by telephone, by computer, tablet or smartphone, or by using mailed paper copies of the surveys and tests. The surveys and tests will take about 1 hour and can be done all together at one time, or can be done separately over a longer time, even different days over a week.

For Part 1, we will ask you questions about you, your health, health behaviors, health problems and any medicines you may be taking. We will ask you questions about your home, and the neighborhood you live in. In addition, we will ask you about COVID-related issues including any testing, if you have gotten the COVID vaccine, and any health problems you had because of COVID or the COVID vaccine. We will look at your health records if you had to stay in the hospital or had any other health care visits because of COVID or the COVID vaccine. If we can't look at your health records, we will ask you questions about your hospital stay or other health care visits instead.

While you are in the study, we will check national registries (places where information about people across the country is kept) and your medical records to find out about your health and about any tests you have had outside the research study. Information from your health records may be included in the study to help the team understand how you were affected by COVID or the COVID vaccine.

For Part 1, you will also get kits sent to your home with instructions on how to collect a little of your blood and saliva (spit) for the study at home, and how to mail these samples to us. Blood will be drawn using an at-home collection device (Tasso m-20) through a needle prick to the skin of their arm. If you have just gotten sick with COVID in the past month, we will wait 8 weeks before getting the Part 1 blood and saliva (spit) from you. Some children and young adults who did not have COVID will also wait 8 weeks before getting the Part 1 blood and saliva. The total amount of blood collected for Part 1 of the study is less than one teaspoon (5 ml). One of the tests is for antibodies to the virus that causes COVID-19. Antibodies are what your body's immune system makes when it sees a new germ. You will get the results of the test, and the research staff will help explain to you what the results mean. The rest of the blood and the saliva will be used for tests in the future.

If the antibody test shows no evidence of a SARS-CoV-2 infection, we may ask you to give a little blood up to two more times for repeat antibody testing about three months apart. This blood will be collected at home or at the study site for testing. You will get the results of the testing.

We may notify your primary care doctor about your participation in this study. Your doctors will choose your treatments based on your needs. Participation in the study does not change your treatment.

Part 2 Surveys and Tests

The Part 2 study visits include the surveys and tests listed below. Counting from the time you enter the study, up to 10 Part 2 visits may be scheduled over 4 years. We will tell you how many visits you will have based on your history related to COVID. Some of the Part 2 visits are surveys that you can do at home, and some you will need to come to the study site (a clinic or hospital with a room for the study visit).

At each Part 2 visit, you will also be asked to fill out surveys at home with questions about any changes in your health, including any testing for COVID, COVID vaccination, medicines you take, and how your health is overall. You will be asked questions about how COVID and the COVID vaccine may have affected you, your household, and whether you have had any health problems because of past infection with the virus that causes COVID or the COVID vaccine. We will look over your health records so we can get information such as test results, any visits to the hospital or a doctor's office, and what kinds of medicines you got.

If you have just gotten sick with COVID in the past month, you will be asked to complete surveys at home for up to three of the first Part 2 visits scheduled about once every two weeks. You will be asked to fill out short surveys about your symptoms by telephone, by computer, tablet or smartphone, or by using mailed paper copies of the surveys and will take less than 30 minutes. If you are not feeling well, you can skip a

Approved For Period: 12/4/2022 - 8/31/2023

survey. One more Part 2 visit will be scheduled about eight weeks later at the study site. At this visit you will have a check-up exam, an electrocardiogram, a breathing test, collection of saliva(spit) and a blood test. These tests are described in the table on the next page. Some children and young adults who did not have COVID will also have these short surveys and the Part 2 visit scheduled about eight weeks after joining the study.

If you have just gotten sick with COVID in the past month, we will send you a measurement tool called a pulse oximeter to your home. A pulse oximeter uses a red light to measure the oxygen in the blood. Oxygen is part of the air we breathe. To measure the oxygen, the oximeter is put over a finger so that the red light shines on the skin of the fingertip. The staff will explain to you exactly how to use the oximeter at home. The oximeter will show how fast your heart is beating (pulse), and the amount of oxygen in the blood. We will ask you to measure the oxygen with the oximeter once in the morning and once in the evening for 7 days at home, and then once a week in the morning for three more weeks at home. We will give you a piece of paper to write down the measurements.

If you had COVID more than a month ago, five Part 2 visits will be scheduled at about 6 months and 1, 2, 3, and 4 years after you enter the study. The Part 2 visits are a mix of surveys that may be done at home and tests that will be done at the study site. These Part 2 surveys and tests will take about one hour at home and 1-2 hours at the study site for each visit. The following table has a description of the Part 2 tests that are done at the study site. Some of the tests may not be done depending on how you are feeling.

**Name of test**	**Where survey or test will take place**	**Information about what will happen**
Check-up or physical exam	Study site	Study staff will measure how you breathe, your height, weight, and other body measurements
Electrocardiogram (ECG)	Study site	An electrocardiogram is a test that records the timing and strength of the electrical signals that make the heart beat. You will be asked to lie down on a bed or stretcher. You will have sticky pads put on your chest, arms and legs. Wires from a computer will be attached to the sticky pads. This test takes about 15 minutes.
Breathing tests (Spirometry)	Study site	Breathing tests use different ways of measuring how well the lungs are working. You will be asked to breathe into a tube as hard and fast as you can. This test takes about 30 minutes.
Getting blood	Study site (or home)	2-3 tablespoons of blood (up to 38 ml) will be taken from a vein in your arm with a thin needle. This will be used to look at how well the heart is working and signs of infection or other problems in the blood (for example inflammation). This will be sent to be stored for testing in the future.
Joint flexibility test (Beighton Scale)	Study site	You will be asked to move parts of your body so we can see how well your arms and legs, spine, elbows, knees and fingers move. This will take about 5 minutes.

Approved For Period: 12/4/2022 - 8/31/2023

Name of test	Where survey or test will take place	Information about what will happen
Tests to see how your brain thinks and learns (Neurocognitive testing)	Study site	This test uses special questionnaires to measure how the brain works. We are interested in the way your brain thinks and learns. These tests are given by a specially trained person (a psychologist or a trained assistant). These tests may take a couple of hours to do.

The tests listed in the table may be repeated at Part 2 visits up to 4 years after you enter the study.

Visit	Week 2	Week 4	Week 8	Month 6	Year 1	Year 2	Year 3	Year 4
Surveys	X*	X*	X	X	X	X	X	X
Examination			X	X	X	X	X	X
ECG			X	X	X	X	X	X
Spirometry			X	X	X	X	X	X
Blood Collection			X	X**	X	X	X	X

Weeks 2, 4, and 8 are only scheduled if you have just gotten sick with COVID in the past month. Some children and young adults who did not have COVID will also do surveys and tests on weeks 2, 4, and 8.

Squares marked with X* means that these visits will include short home surveys done at home taking less than 30 minutes. Other visits will have longer surveys done at home or at the study site taking about one hour.

Squares marked with X** means that 2-3 tablespoons of blood (38 mL) will be collected at this visit. Other visits will be less than three tablespoons of blood (31 mL). The most amount of blood that could be collected for all the Part 2 visits over 4 years is a little more than one-half cup (158 mL). The amount of blood collected for you may be less, depending on your history of COVID-19.

In addition to the study visits listed in the table, we will ask you and check your medical record about any tests or procedures that your doctor has ordered while you are in the study. We will keep track of any samples of biopsies, blood, or body fluids that might have been collected by your doctor, and may transfer some or all of these samples for future use.

Other ways to keep track of your health for the study

We may ask you to share information with us about your health and activity level by using a product or device made by a company. Products or devices are things like fitness trackers, mobile apps that can be used on a smartphone or tablet, websites and web apps, or types of computer software. If you don't already have the product or device, we may give you one to use for the RECOVER study. If we give you a product or device to use, you must agree to the company's rules before you can use it, just as if you bought the product or service for yourself. The researchers of the RECOVER study do not control these rules. We will help you understand these rules in the "Terms of Service" or the "End User License Agreement" papers that come with the product or device. Please read these papers with rules carefully. These rules may ask you to agree to certain things, like not to sue the company if something goes wrong with the product or device. These rules may also allow the company to get, keep, or give others a copy of your information that comes from the product or device. Although the RECOVER study will protect the copy of your information that you give us, we cannot protect or control what the company does with the copy that goes to them. If you do not agree to the company's rules, you do not have to take and use the product or device. You can say no to taking the product or device and still be in other parts of the RECOVER study.

Communicating with the Research Team

Approved For Period: 12/4/2022 - 8/31/2023

The research team will contact you by phone, email or text messages, depending on what you prefer. When the research team sends email messages that include information about your health that is linked to who you are (identifiable), they will help keep your personal information confidential by “encrypting” the message. There is no way to encrypt the messages sent by text. This means that information you send or receive by text message could be looked at by someone who was not supposed to see it, or by your mobile/cell phone provider or company. When text messages are sent, there may be risks related to your privacy. Please indicate whether you agree to receive text messages from the research team:

______ Yes, I agree to receive texts from the research team. ________ *Initial here*

______ No, I do not agree to receive texts from the research team. ______ *Initial here*

Please make sure to keep the research team updated if your address, email, or mobile/cell phone number changes during the study.

Future Use of Survey and Test Results Data

Information that identifies you, such as your name and address, will be removed from the questionnaires and test results. After this happens, information may be used for future research studies or shared with other researchers without a risk of loss of confidentiality to you and we will not request additional informed consent from you to use these results.

Future Use of Blood, Saliva, and Other Things From Your Body (called Biospecimens)

Biospecimens (blood and saliva(spit)) will be sent to a storage place called the RECOVER Research Biorepository at Mayo Clinic, MN to be stored for future research tests. These are called “study samples”.

Blood and saliva in this storage place will be used mostly for research on COVID and the long-term effects of COVID, but they may also be used for research on other health problems.

Your study information and study samples may be shared with researchers around the world, including those working as part of businesses. However, the group in charge of this study (leaders of the RECOVER project at the NIH) controls who can get the information and study samples. To use your information and study samples, researchers must ask the leaders of the RECOVER study if they can be shared. Your name or other information that identifies who you will not be provided to other researchers. Samples that are stored will be given a number (called a code). Only the researchers where you signed up to be part of this research (your study site) and the researchers who are in charge of keeping study information will have the key that links, or connects, the code number given to study samples with personal information. Researchers must also agree to not try to figure out who you are. If you change your mind about sharing your samples, you can take back your study samples and study information by telling the Study Site Leader listed at the beginning of this form.

At the end of the study, your personal information will be taken out of the RECOVER database, and there will no longer be a key to link your information with the study samples. After taking away the key, the data and study samples we got from you for this study may be used for future research studies or shared with other researchers. We will not ask for consent from you to use these data and study samples. After the linking key is removed, you will no longer be able to request to take back your study samples.

5. What are the possible risks or discomforts?

Being in this study may involve some added risks or discomforts from the surveys and tests being done as a part of the study. In addition to the risks listed below, there may be risks that have not appeared

Approved For Period: 12/4/2022 - 8/31/2023

before. You should contact the Study Site Leader as soon as possible if you are concerned about anything while you are part of the study.

Possible loss of privacy (confidentiality)

When we share your information and study samples, there is a small risk that people may get to see it who are not supposed to. Researchers will do their best to protect your information by keeping study information and study samples in safe places and separate from your name and contact information. Researchers using your study samples must agree not to try to find out who you are. However, there is a small chance they may be able to find out who you are.

The research team may communicate with you electronically, such as over email or text message. While the researchers will take steps to protect your privacy and confidentiality, there is a risk that people may see the messages who are not supposed to. The researchers will only send limited information in these messages.

When you use apps or software made by companies, such as wearable fitness trackers, wearable sleep monitors, or other mobile or web apps, there is a small risk that people outside the research study may get to see your information who are not supposed to.

Risk of doing survey questions

Answering the survey questions can take a long time. This may make you tired, uncomfortable, or frustrated. You can stop or take a break if you need to.

Risk of having blood taken

Blood will be drawn through a needle placed into a vein in your arm. Having blood taken may be uncomfortable or make you feel dizzy or faint (pass out). Tell the staff right away if you feel like you might pass out. Redness, pain, bruising, bleeding, or infection may also happen where the needle goes into the skin during blood collection.

Other testing risks

Electrocardiography: The sticky pads (electrodes) that are placed on the chest may be uncomfortable, and cause skin redness or itching. If we need to take off hair from the chest before we stick on the pads, irritation from shaving may also occur.

Breathing (also called pulmonary) tests can make you feel tired, lightheaded or dizzy. These symptoms are short-lasting with no long-term risks.

Collection of other specimens including saliva(spit) may be unpleasant or embarrassing. Those who work for the study will make sure you are given a private space to give these samples.

Findings about Your Genes that Were Not Planned or Not Part of the Main Study

It is possible that during the study, we may do tests on your blood or saliva that find out things out about your genes. You can decide whether you want to be told about these things. There can be a risk in knowing these results. For example, researchers may look at the full set of genes in your body, including looking at the exact order of DNA, the building block of the genes. This is called whole genome sequencing. New information may be found that show that you have genes that make it more likely that you will have certain health problems. Knowing this information can be stressful, lead to worry and affect your relationship with your family. There may also be good things (benefits) about knowing about how genes might affect your health; sometimes you can change your habits or have screening tests that will help you from getting a disease or help you treat a disease earlier.

Group Risks: Although we will not give researchers your name, we will give them basic information such as your race, ethnic group, and sex. This information helps researchers learn whether the factors that lead to health problems are the same in different groups of people. It is possible that such findings could one day help people of the same race, ethnic group, or sex as you. However, they could also be used to

Approved For Period: 12/4/2022 - 8/31/2023

support harmful stereotypes, stigmatize, or discriminate against members of a socially defined group such as race or ethnicity.

6. Can you be in the study if you are pregnant or breastfeeding?

You can be part of this study if you are pregnant or breastfeeding. Being part of this study does not involve any tests in which there is radiation exposure, or any tests that would not be safe during pregnancy or for an infant that is drinking your breast milk.

7. What if new information becomes available?

During this study we may find information that could be important to you. This includes information that might cause you to change your mind about being in the study. We will let you know as soon as possible if this kind of information is found.

New findings of study tests
In this part of the form, we are telling you about new information that may be discovered from study tests.

As part of this study, study test results from certified clinical laboratories (blood tests) and study tests from licensed doctors (body scans and other tests of your heart, lung, belly, and brain) may be shared with you. The study doctors will evaluate the study test results to determine whether or not there is any possible impact on your health. If something that could affect your health is found on the study test, the study doctors will speak to you in person or on the telephone to explain the new information within 10 days after finding the new information. The test findings may be included in your medical record, which means that anybody who is allowed to see your medical record, including your health care providers outside of the study, will be able to see the test results. Your health care providers may order more tests or treatments that are not part of the study testing. You will be financially responsible for any tests or treatments ordered by your health care provider that are not part of the study.

New findings from genetic research
In this part of the form, we are telling you about new information that may be discovered in genetic research tests that will be done in this study. DNA is the material that makes up your genes. Researchers plan to do research on the DNA in your genes while you are in the study, and also in the future after you have finished all of the study visits.

The genetic testing for the study is being done for research, but the study doctors might find a change in your gene DNA that may increase risk of certain diseases or health conditions for you and your family. You can choose whether or not you would like to know about the results of the genetic testing.
If a change in your gene DNA that may increase risk for certain diseases or health conditions is found, the study doctor will double-check this result in a certified clinical laboratory to determine whether or not there is any possible impact on the health of yourself or your family. There is no cost to you for double-checking the result in a certified clinical laboratory. If a result that could affect your health is found on the certified clinical laboratory test, the study doctors will speak to you in person or on the telephone to explain the new information within 10 days after finding the new information. If you choose to know about these genetic test results, it is important for you to know that results from a certified clinical laboratory may also be included in your medical record, which means that anybody who is allowed to see your medical record, including your health care providers outside of the study, will be able to see the test results. Your health care providers may order more tests or treatments that are not part of the study, and may ask you to meet with a genetic counselor to help you understand the results of the genetic test. You will be financially responsible for any tests, treatments, and counseling ordered by your health care provider that are not part of the study.

It is possible that you may have other changes in gene DNA that we do not come across as part of this research. If you would like to have a complete review of your genetic information, we recommend that

Approved For Period: 12/4/2022 - 8/31/2023

you undergo further genetic testing in a certified laboratory and seek genetic counseling to help you understand the results of the testing.

Please initial one of the options below to confirm whether you would like to be informed of findings from study gene testing:

Please initial next to your choice below:
_____ Yes, I would like to be told about gene test results that might affect my health.
Initials here
_____ No, I would not like to be told about gene test results that might affect my health.
Initials here

8. What are the possible good things (benefits) from being part of the study?

We do not expect that your health will get better from being a part of this study. Being part of the study may help you and your doctor better understand problems that are due to COVID. The results of the study will be important in helping patients, caregivers, and parents understand how COVID affects the body long-term.

9. What else can I do if I decide not to be part of the study?

You do not have to be part of this research study. You do not have to take part in this research study to be treated for infection with the virus that causes COVID-19 (SARS-CoV-2 infection). If you decide not to participate in the study, what you decide will not affect your health care in the future, how you pay for health care, or if you can get health insurance.

10. Will I be paid for being in this study?

People who take part in Part 1 and 2 visits will be given money for their time and the cost of travel during the study. You may need to provide travel receipts to the study staff.

Because you will be getting money to be part of this research study, you will need to give the study staff either your Social Security number or your Alien Registration number. You will also be asked to fill out a form called the IRS W9. This is because NYU Langone is required by laws of the United States to report to the Internal Revenue Service (IRS) any amounts that are paid to research participants that are equal to or greater than $600. You may need to pay taxes on payments for research that are greater than $600. If you do not have either of these numbers or are not willing to fill out the IRS W9 form, you can be in the study but will not get any money.

You must keep a record of all the money given to you for being part of this research study and any other research study for each year (from January to December) that you are part of research studies. You must let us know right away when the total amount of money you get for being part of research studies is the same as or likely to be greater than $600 total (not including money to pay for the cost of travel) in any one year (from January to December).

The use of your personal or medical information and study samples may lead to new tests or drugs, or other things that may be sold to make money. A patent or license may be gotten for these things to keep other people from making, using, or selling these things. There are no plans to give any money to you if this happens.

Approved For Period: 12/4/2022 - 8/31/2023

11. Will I have to pay for anything?

There are no costs to you related to your being part of the study. Money from the National Institutes of Health (NIH) will cover the costs of your being part of the study. You or your insurance company will not be asked to pay for the costs of your visits related to the study. You and/or your health insurance may be billed for the costs of health care during this study if these costs would have happened even if you were not in the study. If your insurance does not cover these costs or you do not have insurance, these costs will be up to you to take care of.

12. What happens if you are hurt from being in the study?

For emergencies, call 911. If you think you have been hurt because of being part of this research study, tell the Study Site Leader as soon as you can. The Study Site Leader's name and phone number are listed at the top of page 1 of this consent form.

If you are hurt because of being part of this research, your study site staff can help you find a doctor to give you treatment if you want. We may ask your insurance company, or someone else, if appropriate, to pay for the costs of the treatment due to your being hurt, but you may also need to pay for some of this cost.

There are no plans for the [study site] or NYU Grossman School of Medicine or NYU Langone Health to pay you or give you anything else for being hurt. You do not give up the rights you have under the law by signing this form.

13. When is the study over? Can I leave the study before it ends?

This study will last for 4 years. This study may be stopped early. It is also possible that you may be taken out of the study early for the following reasons:

- The researchers in charge of the study feel it is important to do this for your health or safety.
- You have not followed study instructions.
- The group funding the study, the main researchers in charge, or other group whose job it is to be in charge of looking at the safety of the study decide to stop the study.
- More information about Long COVID is known so that the study is no longer needed.

At any time, if you do not wish to continue to be in the study, you are free to leave the study. Leaving the study will not affect you or your care, how your health care is paid for, or what kind of health insurance you can get.

14. How will we protect your confidentiality (privacy)?

Your medical information is also called protected health information, or "PHI", because it is protected by federal and state laws, such as the Health Insurance Portability and Accountability Act, or HIPAA. This includes information in your research record as well as information in your health record at the place where you signed up to be part of your research (your study site (XXX)). In following the rules of your study site and with HIPAA, only those people who have a reason to look at your health information because of their job can look at this information.

Medical information created by this research study may become part of your medical record. We may include your research information in your medical record for several reasons, including for the billing of services provided in connection with the study, to securely document any medical services you receive, and so that other members of the NYU Langone Health community who may treat you have access to important information about your health.

Approved For Period: 12/4/2022 - 8/31/2023

You have a right to look at the information in your health record. In some cases, when it is needed to make sure the research is done in the best way possible so that results can be trusted, you may not be able to see or make a copy of certain information about the study while the study is going on, but you will have the right to see and copy the information once the study is over in line with your research center's policies and the law.

Certificate of Confidentiality
To help us further protect your privacy (confidentiality), this research is covered by a Certificate of Confidentiality from the National Institutes of Health (NIH). This adds special protection for the research information (data, documents, or biospecimens) that may identify you.

Research information protected by this Certificate of Confidentiality cannot be disclosed to anyone else who is not connected with the research, without your consent. With this Certificate of Confidentiality, the researchers may not disclose or use research information that may identify you in any federal, state, or local civil, criminal, administrative, legislative, or other action, suit, or proceeding, or be used as evidence, for example, if there is a court subpoena, without your consent. However, disclosure, without your consent, is still necessary if there is a federal, state, or local law that requires disclosure (such as to report child abuse or diseases caused by germs that can be spread to others).

The Certificate of Confidentiality cannot be used to refuse a request for information from appropriate government agencies responsible for the project.

By agreeing to be in this research and signing below, you are giving your consent (permission) to share research information with others at NYU Langone Health and other researchers who are working together with NYU Langone Health who are listed in the next section. This means that your research information, including lab results and body pictures, may be included in your medical record.

15. HIPAA Authorization

As noted in the Confidentiality part of this consent form above, federal laws in the U.S. have been made so that we, the researchers working with us, health care providers, and the people who care for you protect the privacy of information that identifies you (used to know who you are) and relates to your past, present, and future physical and mental health problems. We are asking for your permission (authorization) to use and share your health information with others related to this study, in other words, in order for this research to happen, including doing and watching over the study.

Your treatment outside of this study, payment for your health care, and your health care benefits will not be affected even if you do not give permission (authorize) the use and sharing of your information for this study.

What information may be used or shared with others related to this study?
All information in your research record for this study may be used and shared with those people who are in the list at the end of this part of the consent form. Also, information in your health record that the research team believes may be important to the study may be looked at by those in the list. This includes, for example, results from your study visits, laboratory tests, body pictures, scans, other tests, surveys, and diaries.

Who can use and share information in connection with this study?
The following individuals may use, share, or get your information for this research study:

- The research team, including the Study Site Leader and other people helping with the study or who are in charge of watching over the study at the place where you signed up to be part of the research study
- The researchers at NYU Grossman School of Medicine, who are in charge of helping with and watching over the study at all the places across the country where the study is happening

Approved For Period: 12/4/2022 - 8/31/2023

- Non-research staff who need this information to do their jobs (such as for treatment, payment, or health care operations)
- The researchers at Massachusetts General Hospital, who are in charge of storing the information for this study, the researchers at Mayo Clinic who are in charge of the research biorepository, and other RECOVER study centers or national centers in charge of storing research information
- The group that funded the study: National Institutes of Health (National Heart Lung and Blood Institute)
- The ethical review board (also called institutional review board or IRB) that oversees the research and the research quality improvement programs
- The group that is watching over the safety of patients and families in the study (called the Observational Study Monitoring Board). The National Institutes of Health decides who will be in this expert group
- A company hired to provide at-home blood collection devices for the study, for purposes of arranging and shipping the device(s) to your address (Tasso Inc.).
- A company hired to oversee the quality of the RECOVER research information (Biomedical Research Alliance of New York)
- People or groups that we hire to do work for the study, such as fitness tracker companies, smartphone application companies, data storage companies, insurers, and lawyers
- Governmental agencies in charge of watching over the research (for example, the US Department of Health and Human Services)
- Health care providers, including your doctors and others who care for you related to this study, and laboratories or other people who are looking at your health information as part of this study

Your information may be re-disclosed (shared) or used for other reasons if the person who gets your information is not required by law to protect the privacy of the information.

What if you do not want to give permission to use and share your information for this study?
Signing this form is voluntary. You do not have to give us permission to use and share your information, but if you do not, you will not be able to be part of this study.

Can you change your mind and withdraw (take back) permission to use or share your information?
Yes, you may take back your permission to use and share your health information at any time for this research study. If you choose to take back permission, you can choose to stop collection of new information, or request that previously collected information, including biological samples, be removed from the study. If you take back your permission, we will not be able to take back information that has already been used or shared with others. To take back your permission, send a written notice by mail or email to the Study Site Leader noted at the top of page 1 of this form. If you take back your permission, you will no longer be able to stay in this study.

How long can your information be used or shared?
Your permission to use or share your personal health information for this study will never end unless you take it back.

16. What is an Electronic Medical Record?

An Electronic Medical Record (EMR) is an electronic version of your medical chart, or health record. An EMR is simply a computer version of a paper health record.

Having this EMR will let researchers keep information about you related to your being part of this research study. You must agree to make this EMR if you want to be part of this study. In order to make your EMR, the study team will need to get basic information about you that would be similar to the information you would provide the first time you visit a hospital or other place you get health care, for example, your name, the name of your main doctor, the type of insurance you have, your date of birth and other health-related information.

Approved For Period: 12/4/2022 - 8/31/2023

What information may be put in the EMR?
Information related to your being part of the research (like laboratory tests, research-related notes, imaging studies (studies to look at parts of the body), and other study tests, etc.) will be put in your EMR at your study site listed on the first page.

This information can be seen by people who work at your study site who are not part of the research team. Information that is in your EMR may also be shared with others who your study site has decided should be able to look at your EMR (for example, health insurance company, disability provider, etc.).

Will you be able to look at research-related information within the Electronic Medical Record?
A law called the "21st Century Cures Act" makes it easier for patients to look at their EMR.

As part of this research study, some research-related information will be put in your EMR and you will be able to see it right away. You may not be able to see some other research-related information until the end of the study.

17. The Institutional Review Board (IRB) and how it protects you

The Institutional Review Board (IRB) reviews all research involving people before it can be started and then as long as the study continues. The main concern of the IRB is to protect the people who are part of the study. For questions about your rights while you are part of the study, contact the NYU IRB Office number on (212) 263-4110. The NYU School of Medicine's IRB and other IRBs that may be part of this study are made up of doctors, nurses, scientists, and people from the community.

18. Who can you call with questions, or if you are worried about your rights as a research subject?

You can call the IRB with your questions or concerns. Our telephone numbers are listed below. Stuart Katz, MD is the person in charge of this research study. His name and phone number are listed on the first page of this form. If you want to speak with someone not directly involved in this research study, please contact [insert name of contact or IRB]. You can call them at [insert contact information]. You can talk to them about:

- Your rights as a research participant
- Your concerns about the research
- A complaint about the research. Also, if you feel pressured to take part in this research study, or to continue with it, they want to know and can help.

Approved For Period: 12/4/2022 - 8/31/2023

When you sign this form, you are agreeing to take part in this research study as described to you. This means that you have read the consent form, your questions have been answered, and you have decided to volunteer.

Name of Participant (Print)	Signature of Participant	Date
Name of Person Obtaining Consent (Print)	Signature of Person Obtaining Consent	Date

Approved For Period: 12/4/2022 - 8/31/2023

Witness to Consent Process for Non-English-Speaking Subjects (using a translated consent form OR “Short Form” in Subject’s Spoken Language)

Statement of Witness
As someone who understands both English and the language spoken by the subject, I listened to and heard the research team member talk about the information in the English version of the consent form with the subject in the subject’s own language, and that the subject was given the chance to ask questions.

Name of Witness (Print)	Signature of Witness	Date

Witness to Consent of a Subject Who Cannot Read or Write

Statement of Witness
I represent that the consent form was presented orally to the subject in the subject’s own language, that the subject was given the opportunity to ask questions, and that the subject has shown their consent and authorization for being part of the study by (check box that applies).

☐ Subject making his/her own “X” above in the subject signature line

☐ Subject showed approval for being part of the study in another way; describe:

Name of Witness (Print)	Signature of Witness	Date

Approved For Period: 12/4/2022 - 8/31/2023
