# Supplementary material for: Researching COVID to enhance recovery (RECOVER) pediatric study protocol: Rationale, objectives and design
Source: PLoS One. 2024 May 7;19(5):e0285635. doi: 10.1371/journal.pone.0285635 (PMC11075869; doi:10.1371/journal.pone.0285635)
Supplement: S6 File — (PDF) [file pone.0285635.s022.pdf]

[Insert logo here]

Research Subjects Ages Newborn-5 Years Old Informed Consent Form (Parts 1 & 2)

**Title of Study:**<br><br><br><br>**Study Site Leader:**	NIH RECOVER: A Multi-site Observational Study of Post-Acute Sequelae of SARS-CoV-2 Infection in Pediatric Populations<br>Study #: s21-01231<br><br>Name<br>Title<br>Institution<br>Address 1<br>Address 2<br>City, State, Zip Code<br>Telephone
**Study Leader:**	Stuart Katz, MD, MS<br>Helen L. and Martin S. Kimmel Professor of Advanced Cardiac Therapeutics<br>NYU Grossman School of Medicine<br>530 First Avenue, Skirball 9R<br>New York, NY 10016<br>Tel 1-833-422-6819
**Research Information Leader:**	Andrea Foulkes, Sc.D.<br>Director Biostatistics, Massachusetts General Hospital<br>Professor of Medicine, Harvard Medical School<br>50 Staniford Street, Suite 560<br>Boston, MA 02114<br>Tel (617) 724-8208
**Emergency Contact:**	Insert Emergency Contact<br>Insert Phone Number/Pager, etc.
**For questions or concerns about the study, please call:**	Stuart Katz, MD, MS<br>Helen L. and Martin S. Kimmel Professor of Advanced Cardiac Therapeutics<br>NYU Grossman School of Medicine<br>530 First Avenue, Skirball 9R<br>New York, NY 10016<br>Tel 1-833-422-6819

1. About volunteering for this research study

Your child is being invited to take part in a research study. We are asking you, the child's parent or legal guardian, to learn about the study and you can choose whether or not you want your child to take part in this study.

Before you decide if you want your child to be part of this study, you will need to know what the study is about, what your child will have to do in this study, the good things that may come out of being part of the study, and the possible risks (or harms) from being in this study. You may also want to talk about this study and this form with your family, friends, or doctor. If you have any questions about the study or

Approved For Period: 12/4/2022 - 8/31/2023

about this form, please ask us. If you decide that you want your child to take part in this study, you must sign this form. We will give you a copy of this form to keep once you have signed it.

2. Why are we doing this study?

This study is part of a research project paid for by the National Institutes of Health (NIH) called RECOVER (Researching COVID to Enhance Recovery). We are doing this study to understand how COVID, the infection caused by a virus called SARS-CoV-2, affects child health, and why some children who get COVID are still sick many months after being infected. Taking a long time to recover from COVID is called “Long COVID” or “PASC”, which stands for Post-Acute Sequelae of SARS-CoV-2.

Children, including infants, toddlers, pre-school and school-age children, and young adults who have and have not had COVID will be part of the study. This consent form talks about the Part 1 and Part 2 study visits for children from birth to 5 years of age who will be in the research study. If your child reaches their sixth birthday, we will ask you to read and sign a consent form for children ages 6-17 years. You can choose whether you want your child to continue in the study.

3. How long will your child be in the study? How many other people will be in the study?

We are asking that your child be in Part 1 of the study. We may also ask your child to be in Part 2. Being in Part 1 can last up to 6 months. Being in Part 2 can last up to four years. There will be about 19,500 children in Part 1 and about 10,000 children in Part 2. About 4,000 of these children will be infants, toddlers, and pre-school children from birth to 5 years of age.

4. What will your child be asked to do in the study?

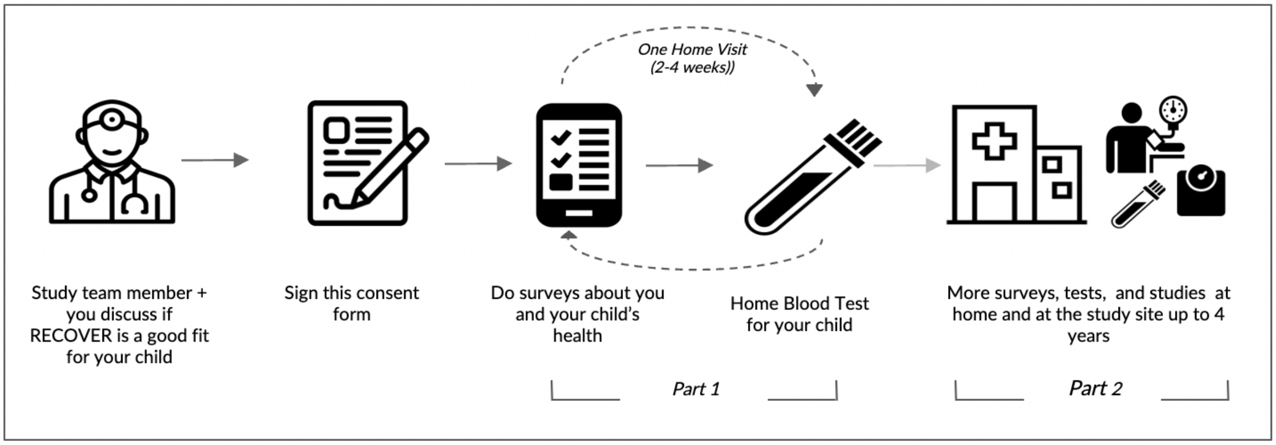

The picture shows the first two parts of the study. All the parents or legal guardians who would like their child to take part in the study will sign the consent form and get the Part 1 surveys and tests. Only about half of the children who do the Part 1 surveys and tests will be asked to do Part 2 surveys and tests. You can choose for your child to be in only Part 1, and not be in Part 2. You are being asked to read and sign this consent because your child has been asked to be in Part 1, and may be asked to be in Part 2. If you choose for your child to be in this study, we will ask you to sign this consent form before your child does any of the Part 1 or Part 2 surveys and tests.

Part 1 Surveys and Tests

Approved For Period: 12/4/2022 - 8/31/2023

Part 1 Surveys and Tests will be done at home. The surveys and tests may be done by telephone, by computer, tablet or smartphone, or by using mailed paper copies of the surveys and tests. The surveys and tests will take about 1 hour and can be done all together at one time, or can be done separately over a longer time, even different days over a week.

For Part 1, we will ask questions about your child's health and home and pre-school activities, your child's health behaviors, and any medicines your child may be taking. We will ask questions about your child's home, and the neighborhood your child lives in. In addition, we will ask questions about COVID testing, COVID vaccines, and whether your child had any health problems because of COVID.

While your child is in the study, we will check national registries (places where information about people across the country is kept) and your child's medical records to find out about your child's health and about any tests your child may have had outside of the research study. Information from your child's health records may be included in the study to help the team understand how your child was affected by COVID or the COVID vaccine.

For Part 1, you will also get kits sent to your home with instructions on how to collect a little blood and spit (saliva) from your child (aged ≥ 24 months) for the study at home, and how to mail these to us. Blood will be drawn using an at-home collection device (Tasso m-20) through a needle prick to the skin of their arm, thigh or back. If your child has gotten sick with COVID in the past month, we will wait 8 weeks before getting the Part 1 blood and spit. Some children and young adults who did not have COVID will also wait 8 weeks before getting the Part 1 blood and spit. The total amount of blood collected for Part 1 of the study is less than one teaspoon (5 mL). One of the blood tests is for antibodies to the virus that causes COVID. Antibodies are made by your child's body when your child is exposed to a new germ. You will get the results of your child's blood test, and the research staff will explain to you what the results mean. The rest of the blood and the saliva will be used for tests in the future.

If the antibody test does not show that your child had an infection with the virus that causes COVID, we may ask your child to give a little blood up to two more times for repeat antibody testing about three months apart. This blood will be collected at home or at the study site for testing. You will get the results of the testing.

We may let your child's main doctor know that your child is part of this study. If your child is having any health problems, your child's doctors will work with you to decide the best way to take care of these problems. Being part of the study does not change the healthcare your child gets from their doctor.

Part 2 Surveys and Tests
The number of Part 2 study visits will depend on your child's history related to COVID. Counting from the time you enter the study, up to ten Part 2 visits may be scheduled over four years. We will tell you how many visits your child will have based on their history related to COVID. Some of the Part 2 visits are surveys you can do at home, and for some you will need to bring your child to the study site (a clinic or hospital with a room for the study visit).

At each Part 2 visit, you will also be asked to fill out surveys at home with questions about any changes in your child's health, including any testing for COVID, COVID vaccination, medicines, and your child's overall health. You will be asked questions about how COVID and the COVID vaccine may have affected your child, and your household, and whether your child had any health problems due to COVID or the COVID vaccine. We will look over your child's health records so we can get information such as test results, any visits to the hospital or a doctor's office, and what kinds of medicines your child got.

If your child has just gotten sick with COVID in the past month, you will be asked to complete surveys at home for up to three Part 2 visits scheduled about once every two weeks. You will be asked to fill out short surveys about your child's symptoms by telephone, by computer, tablet or smartphone, or by using mailed paper copies of the surveys. The questions will take less than 30 minutes to finish. If your child is not feeling well, you can skip a survey. One more visit will be scheduled about eight weeks later at the study site for children 2-5 years of age. At this visit your child will have a check-up exam, short surveys

Approved For Period: 12/4/2022 - 8/31/2023

about your child's symptoms, collection of spit (saliva) and a blood test (about one tablespoon or 15 mL). Blood and spit collection will be postponed until the child reaches the age of 24 months. Some children and young adults who did not have COVID will also have these short surveys and the Part 2 visit scheduled about eight weeks after joining the study.

If your child aged 3-5 years has just gotten sick with COVID in the past month, we may send you a tool called a pulse oximeter to your home. A pulse oximeter uses a red light to measure the oxygen in the blood of your child. Oxygen is part of the air we breathe. To measure the oxygen, the oximeter is put over a finger so that the red light shines on the skin of the fingertip. The staff will explain to you exactly how to use the oximeter for your child. The oximeter will show how fast your child's heart is beating (pulse), and the amount of oxygen in your child's blood. We will ask you to measure your child's oxygen with the oximeter once in the morning and once in the evening for 7 days at home, and then once a week in the morning for three more weeks at home. We will give you a piece of paper to write down the measurements and ask you to send the paper back to the researchers.

If your child had COVID more than a month ago, five Part 2 visits will be scheduled at about 12, 18, 24, 36, and 48 months after you enter the study. The Part 2 visits are a mix of surveys that may be done at home and tests that will be done at the study site. For infants ages newborn to 1 year, all the Part 2 tests will be at home. For children ages 2-5 years, Part 2 surveys and tests will take about one hour at home and 1-2 hours at the study site for each visit. The questions done at home will ask about your child's health and how your child's behavior and development has changed over time. The following table has a description of the Part 2 tests that are done at the study site. Some of the tests may not be done depending on your child's age and how your child is feeling.

**Name of test**	**Where survey or test will take place**	**Information about what will happen**
Check-up or physical exam ages 2-5 years	Study site	Study staff will measure your child's height, weight, pulse, blood pressure, and oxygen level.
Electrocardiogram (ECG) ages 3-5 years	Study site	An electrocardiogram is a test that records the electrical signals that make the heart beat. Your child will be asked to lie down on a bed or stretcher. Sticky pads will be put on the skin of your child's chest, arms and legs. Wires from a computer will be attached to the sticky pads to record the electric signal. The sticky pads and wires will be removed. This test takes about 15 minutes.
Breathing tests (Spirometry) ages 3-5 years	Study site	Breathing tests measure how well the lungs are working. Your child will be asked to breathe into a tube and blow as hard and fast as they can. This test takes about 30 minutes.
Getting blood ages 2-5 years	Study site (or home)	1 tablespoon of blood (up to 15 mL) will be taken from a vein in your child's arm with a thin needle. Some of this blood will be used to test the effects of COVID on the body and some will be stored for future tests.
Joint flexibility test (Beighton Scale) ages 3-5 years	Study site	You will be asked to move parts of your child's body so we can see how well your child's arms and legs, spine, elbows, knees and fingers move. This will take about 5 minutes.

Approved For Period: 12/4/2022 - 8/31/2023

Name of test	Where survey or test will take place	Information about what will happen
Tests to see how your child's brain thinks and learns (Neurocognitive testing) ages 3-5 years	Study site	This test uses special survey questions to measure how the brain works. We are interested in the way your child's brain thinks and learns.

The tests listed in the table may be repeated at Part 2 visits for up to 4 years after you enter the study.

Visit	Week 2	Week 4	Week 8	Month 6	Month 12	Month 18	Month 24	Month 36	Month 48
Surveys	X*	X*	X	X	X	X	X	X	X
Check-up**			X	X	X		X	X	X
ECG***			X	X	X		X	X	X
Spirometry***			X	X	X		X	X	X
Spit Collection**			X						
Blood Collection**			X		X		X	X	X

Weeks 2, 4, and 8 are only scheduled if your child has just gotten sick with COVID in the past month. For children ages newborn to 1 year, only home surveys will be done at 8 weeks; other tests including spit and blood collection will be postponed until after the child reaches age of ≥ 24 months. Some children and young adults who did not have COVID will also do surveys and tests on weeks 2, 4, and 8.

Squares marked with X* means that these visits will include short home surveys done at home taking less than 30 minutes. Other survey visits will have longer surveys done at home or at the study site taking about one hour.

** means for children ages 2-5 years

*** means for children ages 3-5 years

The amount of blood collected depends on the age of the child at the time of the study visit. For children under 24 months, no blood will be collected. For children aged 2-5 years, one tablespoon of blood (15 mL) will be collected. If your child continues to be in Part 2 of the study past the age of 5 years, two tablespoons of blood (24 mL) will be collected. The most amount of blood that could collected for all the Part 2 visits over 4 years is a little less than one-half cup (105 mL). The amount of blood collected for your child may be less, depending on your child's age and history of COVID.

In addition to the study visits listed in the table, we will check your child's medical record about any tests or procedures that their doctor has ordered while in the study. We will keep track of things your child's doctor has collected, like blood, spit (saliva), and any liquid or tissues from the body, and may use some or all of these samples for future testing.

Other ways to keep track of your child's health for the study
We may ask you to share information with us about your child's health and activity level by using a product or device made by a company. Products or devices are things like fitness trackers, mobile apps that can be used on a smartphone or tablet, websites and web apps, or types of computer software. If you don't already have the product or device, we may give you one for your child to use for the RECOVER study. If we give your child a product or device to use, you must agree to the company's rules before your child can use it, just as if you bought the product or service yourself. These rules may ask you to agree to certain things, like not to sue the company if something goes wrong with the product or device. These rules may also allow the company to get, keep, or give others a copy of your information that comes from the product or device. Although the RECOVER study will protect the copy of the

Approved For Period: 12/4/2022 - 8/31/2023

information about your child that you give us, we cannot protect or control what the company does with the copy that goes to them. If you do not agree to the company's rules, you do not have to take and use the product or device. You can say no to taking the product or device and still have your child be in other parts of the RECOVER study.

Communicating with the Research Team
The research team will contact you by phone, email or text messages, depending on what you prefer. When the research team sends email messages that include information about your child's health that is linked to you or your child by name, they will help keep your personal information confidential by "encrypting" the message. There is no way to encrypt the messages sent by text. This means that information you send or receive by text message could be looked at by someone who was not supposed to see it, or by your mobile/cell phone provider or company. When text messages are sent, there may be risks related to your privacy or your child's privacy. Please indicate whether you agree to receive text messages from the research team:

______ Yes, I agree to receive texts from the research team. ________ *Initial here*

______ No, I do not agree to receive texts from the research team. ______ *Initial here*

Please make sure to keep the research team updated if your address, email, or mobile/cell phone number changes during the study.

Future Use of Survey and Test Results Data
Information that identifies you or your child, such as your name and address, will be removed from the questionnaires and test results. After this happens, information may be used for future research studies or shared with other researchers, and we will not request additional informed consent from you to use these results.

Future Use of Blood, Saliva, and Other Things From Your Child's Body (called Biospecimens)
Some of the Part 1 and Part 2 samples of blood and spit (saliva) will be sent to a storage place called the RECOVER Research Biorepository at Mayo Clinic, MN to be stored for future research tests. These stored samples of blood and spit are called "study samples".

Study samples in this storage place will be used mostly for research on COVID and the long-term effects of COVID, but they may also be used for research on other health problems.

Your child's study information and study samples may be shared with researchers around the world, including those working as part of businesses. However, the group in charge of this study (leaders of the RECOVER project at the NIH) controls who can get the information and study samples. Your child's name or other information that identifies your child will not be provided to other researchers. Samples that are stored will be given a number (called a code). Only the researchers where you signed up to be part of this research (your study site) and the researchers who are in charge of keeping study information will have the key that links, or connects, the code number given to study samples with your child's name or other personal information. Researchers must also agree to not try to figure out who your child is. If you change your mind about sharing your child's study samples, you can take back your child's study samples and study information by telling the Study Site Leader listed at the beginning of this form.

At the end of the study, your child's personal information will be taken out of the RECOVER database, and there will no longer be a key to link your child's information with the study samples. After taking away the key, the data and study samples we got from your child for this study may be used for future research studies or shared with other researchers. We will not ask for consent from you to use these data and study samples. After the linking key is removed, you will no longer be able to request to take back your child's study samples.

Approved For Period: 12/4/2022 - 8/31/2023

5. What are the possible risks or discomforts for your child?

Being in this study may involve some added risks or discomforts to your child from the surveys and tests done as part of the study. In addition to the risks listed below, there may be risks that have not appeared before. You should contact the Study Site Leader as soon as possible if you are concerned about anything that happens to your child while she/he is part of the study.

Possible loss of privacy (confidentiality)
When we share your child's information and study samples, there is a small risk that people may get to see it who are not supposed to. Researchers will do their best to protect your child's information by keeping study information and study samples in safe places and separate from your child's name and contact information.

The research team may communicate with you electronically, such as over email or text message. While the researchers will take steps to protect you and your child's privacy and confidentiality, there is a risk that people may see the messages who are not supposed to. The researchers will only send limited information in these messages.

When you use apps or software made by companies, such as wearable fitness trackers, wearable sleep monitors, or other mobile or web apps, there is a small risk that people outside the research study may get to see your child's information who are not supposed to.

Risk of doing survey questions
Answering the survey questions can take a long time. This may make you feel tired, uncomfortable, or frustrated. You can stop or take a break if you need to.

Risk of having blood taken
Some of the blood samples will be taken through a needle placed into a vein in your child's arm, thigh or back. Having blood taken may be uncomfortable or make your child feel dizzy or faint (pass out). Tell the staff right away if your child is uncomfortable or feeling dizzy. Redness, pain, bruising, bleeding, or infection may also happen where the needle goes into the skin during blood collection.

Other testing risks
Electrocardiography: The sticky pads (electrodes) that are placed on the skin of the chest may be uncomfortable, and cause skin redness or itching.

Breathing tests (also called pulmonary tests) can make your child feel tired, lightheaded or dizzy. These symptoms are short-lasting with no long-term risks.

Collection of other specimens including spit (saliva) may be unpleasant or embarrassing. Those who work for the study will make sure your child is given a private space to give these samples.

Findings about Your Child's Genes that Were Not Planned or Not Part of the Main Study
It is possible that during the study, we may do tests on your child's blood or saliva that find things out about your child's genes. Genes are the part of your body that have DNA, the building blocks of the body that are passed on from parent to child. You can decide whether you want to be told about these gene test results. There can be a risk in knowing these results. New information may be found that show that your child has genes that make it more likely that your child will have certain health problems. Knowing this information can be stressful, lead to worry and affect your relationship with your family. There may also be good things (benefits) about knowing about how genes might affect your child's health; sometimes you can change your child's habits or have screening tests for your child that will help keep your child from getting a disease or help treat a disease earlier.

Group Risks: Although we will not give researchers your child's name, we will give them basic information such as your child's race, ethnic group, and sex. This information helps researchers learn whether the factors that lead to health problems are the same in different groups of people. It is possible that such findings could one day help a group of people of the same race, ethnic group, or sex as your

Approved For Period: 12/4/2022 - 8/31/2023

child. However, the findings could also be used to support harmful stereotypes, or discriminate against a group of people of the same race, ethnic group, or sex as your child.

6. What if new information becomes available?

During this study we may find information that could be important to you and your child. This includes information that might cause you to change your mind about your child being in the study. We will let you and your child know as soon as possible if this kind of information is found.

New findings of study tests
In this part of the form, we are telling you about new information that may be discovered from study tests.

As part of this study, study test results from certified clinical laboratories (blood tests) and study tests from licensed doctors (body scans and other tests of your child's heart, lung, belly, and brain) may be shared with you and your child. The study doctors will evaluate the study test results to determine whether or not there is any possible impact on your child's health. If something that could affect your child's health is found on the study test, the study doctors will speak to you in person or on the telephone to explain the new information within 10 days after finding the new information. The test findings may also be included in your child's medical record, which means that anybody who is allowed to see your child's medical record, including your child's health care providers outside of the study, will be able to see the test results. Your child's health care providers may order more tests or treatments that are not part of the study testing. You will be financially responsible for any tests or treatments ordered by your child's health care provider that are not part of the study.

New findings from genetic research
In this part of the form, we are telling you about new information that may be discovered in genetic research tests that will be done in this study. DNA is the material that makes up your child's genes. Researchers plan to do research on the DNA in your child's genes while your child is in the study, and also in the future after your child has finished all of the study visits.

The genetic testing for the study is being done for research, but the study doctors might find a change in your child's gene DNA that may increase risk of certain diseases or health conditions for your child and your family. You can choose whether or not you would like to know about the results of the genetic testing.

If a change in your child's gene DNA that may increase risk for certain diseases or health conditions is found, the study doctor will double-check this result in a certified clinical laboratory to determine whether or not there is any possible impact on the health of your child or your family. There is no cost to you for double-checking the result in a certified clinical laboratory. If a result that could affect your child's health is found on the certified clinical laboratory test, the study doctors will speak to you in person or on the telephone to explain the new information and arrange for you to see a genetic counselor to better understand the test results. If you choose to know about these genetic test results, it is important for you to know that results from a certified clinical laboratory may also be included in your child's medical record, which means that anybody who is allowed to see your child's medical record, including your child's health care providers outside of the study, will be able to see the test results. Your child's health care providers may order more tests or treatments that are not part of the study and may ask you to meet with a genetic counselor to help you understand the results of your child's genetic test. You will be financially responsible for any tests, treatments, and counseling ordered by your child's health care provider that are not part of the study.

It is possible that your child may have other changes in gene DNA that we do not come across as part of this research. If you would like to have a complete review of your child's genetic information, we recommend that your child undergo further genetic testing in a certified laboratory and seek genetic counseling to help you understand the results of the testing.

Approved For Period: 12/4/2022 - 8/31/2023

Please initial one of the options below to confirm whether you would like to be informed of findings from study gene testing:

Please initial next to your choice below:

_____ Yes, I would like to be told about gene test results that might affect my child's health.
Initials here

_____ No, I would not like to be told about gene test results that might affect my child's health.
Initials here

If you are providing permission on behalf of your child, your child will have the opportunity to change this decision once they turn the legal age of majority (become an adult).

7. What are the possible good things (benefits) for your child from being part of the study?

We do not expect that your child's health will get better from being a part of this study. Being part of the study may help you, your child, and your child's doctor better understand problems that are due to COVID. The results of the study will be important in helping children, caregivers, and parents understand how COVID affects the body long-term.

8. What else can I do if I decide not to have my child be part of the study?

You do not have to have your child be part of this research study. You do not have to take part in this research study for your child to be treated for infection with the virus that causes COVID (SARS-CoV-2 infection). If you decide not to have your child participate in the study, what you decide will not affect your child's health care in the future, how you pay for your child's health care, or if you can get health insurance for you or your child.

9. Will I be paid for my child being in this study?

The parents or legal guardians of children who take part in the RECOVER study will be given payments on behalf of the time and efforts their child is spending on the study.

Because you may be getting money for your child to be part of this research study, you will need to give the study staff either your Social Security number or your Alien Registration number. You will also be asked to fill out a form called the IRS W9. This is because NYU Langone is required by laws of the United States to report to the Internal Revenue Service (IRS) any amounts that are paid to research participants that are equal to or greater than $600. You may need to pay taxes on payments for research that are greater than $600. If you do not have either of these numbers or are not willing to fill out the IRS W9 form, your child can be in the study but will not get any money.

You must keep a record of all the money given to you while your child is part of this research study and any other research study that you or your child are part of for each year (from January to December). You must let us know right away when the total amount of money you get for you or your child being part of research studies is the same as or likely to be greater than $600 total (not including money to pay for the cost of travel) in any one year (from January to December).

The use of your child's personal or study information and study samples may lead to new tests or drugs, or other things that may be sold to make money. There are no plans to give any money to you or your child if this happens.

Approved For Period: 12/4/2022 - 8/31/2023

10. Will I or my child have to pay for anything?

There are no costs to you or your child related to your child being part of the study. Money from the National Institutes of Health (NIH) will cover the costs of your child being part of the study. You or your insurance company will not be asked to pay for the costs of your child's visits related to the study. You and/or your health insurance may be billed for the costs of your child's health care during this study if these costs would have happened even if your child were not in the study. If your insurance does not cover these costs or you do not have insurance, these costs will be up to you to take care of.

11. What happens if your child is hurt from being in the study?

For emergencies, call 911. If you think your child has been hurt because of being part of this research study, tell the Study Site Leader as soon as you can. The Study Site Leader's name and phone number are listed at the top of page 1 of this consent form.

If your child is hurt because of being part of this research, your study site staff can help you find a doctor to give your child treatment if you want. We may ask your insurance company, or someone else, if appropriate, to pay for the costs of the treatment due to your child being hurt, but you may also need to pay for some of this cost.

There are no plans for the [study site] or NYU Grossman School of Medicine or NYU Langone Health to pay you or give you anything else for your child being hurt. You do not give up the rights you have under the law by signing this form.

12. When is the study over? Can my child leave the study before it ends?

This study will last for up to 4 years. This study may be stopped early. It is also possible that your child may be taken out of the study early for the following reasons:

- The researchers in charge of the study feel it is important to do this for your child's health or safety.
- You or your child have not followed study instructions.
- The group funding the study, the main researchers in charge, or other group whose job it is to be in charge of looking at the safety of the study decide to stop the study.
- More information about Long COVID is known so that the study is no longer needed.

At any time, if you do not wish for your child to continue to be in the study, you are free to have your child leave the study. Leaving the study will not affect you or your child's care, how your child's health care is paid for, or what kind of health insurance you or your child can get.

13. How will we protect your child's confidentiality (privacy)?

Your child's medical information is also called protected health information, or "PHI", because it is protected by federal and state laws, such as the Health Insurance Portability and Accountability Act, or HIPAA. This includes information in your child's research record as well as information in your child's health record at the place where you signed up to be part of the research (your study site (XXX)). In following the rules of your study site and with HIPAA, only those people who have a reason to look at your child's health information because of their job can look at this information.

Medical information created by this research study may become part of your child's medical record. We may include your child's research information in your child's medical record for several reasons, including for the billing of services provided in connection with the study, to securely document any medical services your child receives, and so that other members of the NYU Langone Health community and your study site who may treat your child have access to important information about your child's health.

Approved For Period: 12/4/2022 - 8/31/2023

You have a right to look at the information in your child's health record. In some cases, when it is needed to make sure the research is done in the best way possible so that results can be trusted, you may not be able to see or make a copy of certain information about the study while the study is going on, but you will have the right to see and copy the information once the study is over in line with your research center's policies and the law.

Certificate of Confidentiality
To help us further protect your child's privacy (confidentiality), this research is covered by a Certificate of Confidentiality from the National Institutes of Health (NIH). This adds special protection for the research information (data, documents, or biospecimens) that may identify you or your child.

Research information protected by this Certificate of Confidentiality cannot be disclosed to anyone else who is not connected with the research, without your consent. With this Certificate of Confidentiality, the researchers may not disclose or use research information that may identify you or your child in any federal, state, or local civil, criminal, administrative, legislative, or other action, suit, or proceeding, or be used as evidence, for example, if there is a court subpoena, without your consent. However, disclosure, without your consent, is still necessary if there is a federal, state, or local law that requires disclosure (such as to report child abuse or diseases caused by germs that can be spread to others).

The Certificate of Confidentiality cannot be used to refuse a request for information from appropriate government agencies responsible for the project.

By agreeing to be in this research and signing below, you are giving your consent (permission) to share your child's research information with others at NYU Langone Health and other researchers who are working together with NYU Langone Health who are listed in the next section. This means that your child's research information, including lab results and body pictures, may be included in your child's medical record.

14. HIPAA Authorization

As noted in the Confidentiality part of this consent form above, federal laws in the U.S. have been made so that we, and the researchers working with us, health care providers, and the people who care for your child protect the privacy of information that identifies you or your child (used to know who you are) and relates to your child's past, present, and future physical and mental health problems. We are asking for your permission (authorization) to use and share your child's health information with others related to this study, in other words, in order for this research to happen, including doing and watching over the study.

Your child's treatment outside of this study, payment for your child's health care, and your child's health care benefits will not be affected even if you do not give permission (authorize) for the use and sharing of your child's information for this study.

What information may be used or shared with others related to this study?
All information in your child's research record for this study may be used and shared with those people who are in the list at the end of this part of the consent form. Also, information in your child's health record that the research team believes may be important to the study may be looked at by those in the list. This includes, for example, results from your child's study visits, laboratory tests, body pictures, scans, other tests, surveys, and diaries.

Who can use and share information in connection with this study?
The following individuals may use, share, or get you and your child's information for this research study:

- The research team, including the Study Site Leader and other people helping with the study or who are in charge of watching over the study at the place where you signed up to be part of the research study
- The researchers at NYU Grossman School of Medicine, who are in charge of helping with and watching over the study at all the places across the country where the study is happening

Approved For Period: 12/4/2022 - 8/31/2023

- Non-research staff who need this information to do their jobs (such as for treatment, payment, or health care operations)
- The researchers at Massachusetts General Hospital, who are in charge of storing the information for this study, the researchers at Mayo Clinic who are in charge of the research biorepository, and other RECOVER study centers or national centers in charge of storing research information.
- The group that funded the study: National Institutes of Health (National Heart Lung and Blood Institute)
- The ethical review board (also called institutional review board or IRB) that oversees the research and the research quality improvement programs
- The group that is watching over the safety of patients and families in the study (called the Observational Study Monitoring Board). The National Institutes of Health decides who will be in this expert group.
- A company hired to provide at-home blood collection devices for the study, for purposes of arranging and shipping the device(s) to your address (Tasso Inc.).
- A company hired to oversee the quality of the RECOVER research information (Biomedical Research Alliance of New York)
- People or groups that we hire to do work for the study, such as fitness tracker companies, smartphone application companies, data storage companies, insurers, and lawyers
- Governmental agencies in charge of watching over the research (for example, the US Department of Health and Human Services)
- Health care providers, including your child's doctors and others who care for your child related to this study, and laboratories or other people who are looking at your child's health information as part of this study

Your and your child's information may be re-disclosed (shared) or used for other reasons if the person who gets your information is not required by law to protect the privacy of the information.

What if you do not want to give permission to use and share your child's information for this study?
Signing this form is voluntary. You do not have to give us permission to use and share your child's information, but if you do not, you will not be able to be part of this study.

Can you change your mind and withdraw (take back) permission to use or share your child's information?
Yes, you may take back your permission to use and share your child's health information at any time for this research study. If you choose to take back your permission, you can choose to stop collection of new information, or request that previously collected information, including biological samples, be removed from the study. If you take back your permission, we will not be able to take back information that has already been used or shared with others. To take back your permission, send a written notice by mail or email to the Study Site Leader noted at the top of page 1 of this form. If you take back your permission, your child will no longer be able to stay in this study.

How long can your child's information be used or shared?
Your permission to use or share your child's personal health information for this study will never end unless you take it back.

15. What is an Electronic Medical Record?

An Electronic Medical Record (EMR) is an electronic version of your child's medical chart, or health record. An EMR is simply a computer version of a paper health record.

Having this EMR will let researchers keep information about your child related to your child being part of this research study. You must agree to make this EMR if you want to be part of this study. In order to make your child's EMR, the study team will need to get basic information about you and your child that would be similar to the information you would provide the first time you visit a hospital or other place you

Approved For Period: 12/4/2022 - 8/31/2023

get health care, for example, your name, your child's name, the name of your child's main doctor, the type of insurance your child has, your child's date of birth and other health-related information.

What information may be put in the EMR?
Information related to your child being part of the research (like laboratory tests, research-related notes, imaging studies (studies to look at parts of the body), and other study tests, etc.) will be put in your child's EMR at your study site listed on the first page.

This information can be seen by people who work at your child's study site who are not part of the research team. Information that is in your child's EMR may also be shared with others who your study site has decided should be able to look at your child's EMR (for example, health insurance company, disability provider, etc.).

Will you be able to look at research-related information within the Electronic Medical Record?
A law called the "21st Century Cures Act" makes it easier for patients to look at their EMR.

As part of this research study, some research-related information will be put in your child's EMR and you will be able to see it right away. You may not be able to see some other research-related information until the end of the study.

16. The Institutional Review Board (IRB) and how it protects you

The Institutional Review Board (IRB) reviews all research involving people before it can be started and then as long as the study continues. The main concern of the IRB is to protect the people who are part of the study. For questions about your child's rights while she/he is part of the study, contact the NYU IRB Office number on (212) 263-4110. The NYU School of Medicine's IRB and other IRBs that may be part of this study are made up of doctors, nurses, scientists, and people from the community.

17. Who can I call with questions, or if I am worried about my child's rights as a research subject?

You can call the IRB with your questions or concerns. Our telephone numbers are listed below. Stuart Katz, MD is the person in charge of this research study. His name and phone number are listed on the first page of this form. If you want to speak with someone not directly involved in this research study, please contact [insert name of contact or IRB]. You can call them at [insert contact information]. You can talk to them about:

- Your child's rights as a research participant
- Your concerns about the research
- A complaint about the research. Also, if you feel pressured to have your child take part in this research study, or to continue with it, they want to know and can help.

Approved For Period: 12/4/2022 - 8/31/2023

When you sign this form, you are agreeing to have your child take part in this research study as described to you. This means that you have read the consent form, your questions have been answered, and you have decided to volunteer to have your child be in the study.

____
Name of Child Participant (Print)

Name of Parent/Guardian (Print)	Signature of Parent/Guardian	Date
Name of Person Obtaining Consent (Print)	Signature of Person Obtaining Consent	Date

Approved For Period: 12/4/2022 - 8/31/2023

Witness to Consent Process for Non-English-Speaking Subjects (using a translated consent form OR "Short Form" in Parent/Guardian's Spoken Language)

Statement of Witness

As someone who understands both English and the language spoken by the Parent/Guardian, I listened to and heard the research team member talk about the information in the English version of the consent form with the Parent/Guardian in their own language, and that the Parent/Guardian was given the chance to ask questions.

Name of Witness (Print)	Signature of Witness	Date

Witness to Consent of a Parent/Guardian Who Cannot Read or Write

Statement of Witness

I represent that the consent form was presented orally to the Parent/Guardian in their own language, that the Parent/Guardian was given the opportunity to ask questions, and that the subject has shown their consent and authorization for being part of the study by (check box that applies).

☐Parent/Guardian making their own "X" above in the subject signature line

☐Parent/Guardian showed approval for being part of the study in another way; describe:

Name of Witness (Print)	Signature of Witness	Date

Approved For Period: 12/4/2022 - 8/31/2023
