# Supplementary material for: Researching COVID to enhance recovery (RECOVER) pediatric study protocol: Rationale, objectives and design
Source: PLoS One. 2024 May 7;19(5):e0285635. doi: 10.1371/journal.pone.0285635 (PMC11075869; doi:10.1371/journal.pone.0285635)
Supplement: S7 File — (PDF) [file pone.0285635.s023.pdf]

[Insert logo here]

Other Biological Parent
Informed Consent Form

**Title of Study:**<br><br><br>**Study Site Leader:**	NIH RECOVER: A Multi-site Observational Study of Post-Acute Sequelae of SARS-CoV-2 Infection in Pediatric Populations<br>Study #: s21-01231<br><br>Name<br>Title<br>Institution<br>Address 1<br>Address 2<br>City, State, Zip Code<br>Telephone
**Study Leader:**	Stuart Katz, MD, MS<br>Helen L. and Martin S. Kimmel Professor of Advanced Cardiac Therapeutics<br>NYU Grossman School of Medicine<br>530 First Avenue, Skirball 9R<br>New York, NY 10016<br>Tel 1-833-422-6819
**Research Information Leader:**	Andrea Foulkes, Sc.D.<br>Director Biostatistics, Massachusetts General Hospital<br>Professor of Medicine, Harvard Medical School<br>50 Staniford Street, Suite 560<br>Boston, MA 02114<br>Tel (617) 724-8208
**Emergency Contact:**	Insert Emergency Contact<br>Insert Phone Number/Pager, etc.
**For questions or concerns about the study, please call:**	Stuart Katz, MD, MS<br>Helen L. and Martin S. Kimmel Professor of Advanced Cardiac Therapeutics<br>NYU Grossman School of Medicine<br>530 First Avenue, Skirball 9R<br>New York, NY 10016<br>Tel 1-833-422-6819

1. About volunteering for this research study

You are being invited to take part in a research study. You can choose whether or not you want to take part in this study.

Before you decide if you want to be part of this study, you will need to know what the study is about, what you will have to do in this study, the good things that may come out of being part of the study, and the possible risks (or harms) from being in this study. You may also want to talk about this study and this form with your family, friends, or doctor. If you have any questions about the study or about this form, please ask us. If you decide to take part in this study, you must sign this form. We will give you a copy of this form to keep once you have signed it.

2. What is the purpose of this study?

This study is part of a research project paid for by the National Institutes of Health (NIH) called RECOVER (Researching COVID to Enhance Recovery). We are doing this study to understand how

Approved For Period: 12/4/2022 - 8/31/2023

COVID, the infection caused by a virus called SARS-CoV-2, affects the body, and why some people who got COVID are still sick many months after being infected. This condition is called “Long COVID” or “PASC”, which stands for Post-Acute Sequelae of SARS-CoV-2.

Children and young adults who have and have not had COVID-19 will be part of the study. The main person taking care of these children (called caregiver) is also being invited to take part in this study. If the primary caretaker is a biological parent, we are asking the other biological parent to be in the study as well. Your child and the child’s caregiver can be in the study even if you decide that you do not want to be in the study.

We are asking you to be in this research study to understand how a parent’s genes may affect how a child gets better from COVID-19. DNA is the material that makes up your genes and is inside your cells. All living things are made of cells. The genes in your cells have the instructions that tell our bodies how to grow and work. For example, genes control the color of a person’s hair or eyes. Genes are passed from a parent to child.

3. Who is the main caregiver?

The main caregiver is a person, like a family member (biological (blood related) or non-biological (not blood related)) or guardian, who is in charge of taking care of the child who is part of the study. The main caregiver:

- Is the adult who spends the most time with the child.
- Is the person most responsible for taking care of the child every day.
- Lives in the same home as the child.
- Knows the most about the child.
- Must be older than 18 years of age if the main caregiver is not blood related.

You are being asked to be in this study if the main caregiver is a biological parent of the child and you are the other biological parent. You may not be able to give consent if you are not yet an adult (have not reached the age of majority, as defined by the local state regulations). In this case, your parent(s) or guardian will be asked to give consent. We will also ask you to agree to take part in the study.

Throughout the consent form, “I”, “you” and “your” always refers to the “other biological parent” (the person who is taking part in the study).

4. How long will you be in the study? How many other people will be in the study?

This study will last 4 years. Your participation will require one study visit.

We plan to have up to 19,500children and young adults up to age 25 years old be part of this study across the country.

The main caregivers of all of these children and young adults will be invited to be part of the study. We think that up to 19,500caregivers will be in the study. We also think that up to 19,500other biological parents will also be in the study.

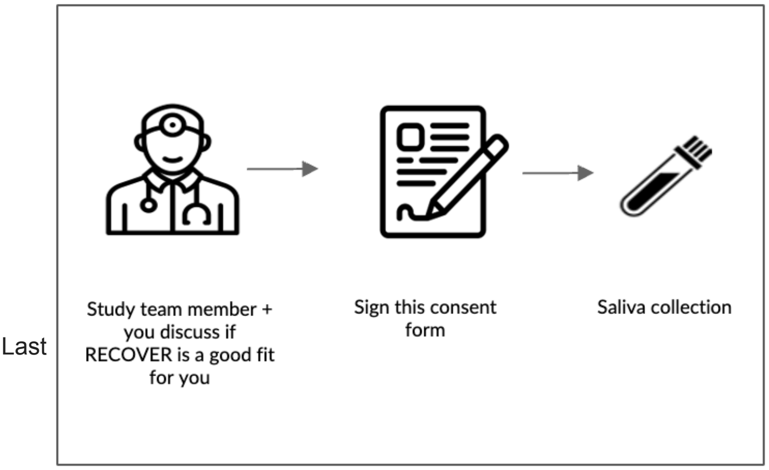

5. What will you be asked to do in the study?

If you choose to take part in the study, we will ask you to sign this consent form before you do any other parts of the study. We will use a computer, tablet or smart phone to

have you sign the form. The study staff will help you with this.

You will be asked to give your name, date of birth, sex, race and ethnicity and contact information for you and two family members or friends including mailing address, telephone, and email. You will also get a kit with instructions on how to send us a little of your saliva, and how to mail this to us. The saliva will be used for tests in the future.

Communicating with the Research Team
The research team will contact you by phone, email or text messages, depending on what you prefer. When the research team sends email messages that include information about your health that is linked to who you are (identifiable), they will help keep your personal information confidential by “encrypting” the message. There is no way to encrypt the messages sent by text. This means that information you send or receive by text message could be looked at by someone who was not supposed to see it, or by your mobile/cell phone provider or company. Therefore, when text messages are sent, there may be risks related to your privacy. Please indicate whether you agree to receive text messages from the research team:

______ Yes, I agree to receive texts from the research team. ________ *Initial here*

______ No, I do not agree to receive texts from the research team. ______ *Initial here*

Please make sure to keep the research team updated if your address, email, or mobile/cell phone number changes during the study.

Future Use of Survey and Test Results Data
Information that identifies you, such as your name and address, will be removed from the questionnaires and test results. After this happens, information may be used for future research studies or shared with other researchers without a risk of loss of confidentiality to you and we will not request additional informed consent from you to use these results.

Future Use of Saliva (called Biospecimens)
Biospecimens (spit (saliva)) will be sent to a storage place called the RECOVER Research Biorepository at Mayo Clinic, MN to be stored for future research tests. These are called “study samples”.

Saliva in this storage place will be used mostly for research on COVID and the long-term effects of COVID, but they may also be used for research on other health problems.

Your study information and study samples may be shared with researchers around the world, including those working as part of businesses. However, the group in charge of this study (leaders of the RECOVER project at the NIH) controls who can get the information and study samples. To use your information and study samples, researchers must ask the leaders of the RECOVER study if they can be shared. Your name or other information that identifies who you are will not be provided to other researchers. Samples that are stored will be given a number (called a code). Only the researchers where you signed up to be part of this research (your study site) and the researchers who are in charge of keeping study information will have the key that links, or connects, the code number given to study samples with personal information. Researchers must also agree to not try to figure out who you are. If you change your mind about sharing your samples, you can take back your study samples and study information by telling the Study Site Leader listed at the beginning of this form.

At the end of the study, your personal information will be taken out of the RECOVER database, and there will no longer be a key to link your information with the study samples. After taking away the key, the data and study samples we got from you for this study may be used for future research studies or shared with other researchers. We will not ask for consent from you to use these data and study samples. After the linking key is removed, you will no longer be able to request to take back your study samples.

Approved For Period: 12/4/2022 - 8/31/2023

6. What are the possible risks or discomforts?

Being in this study may involve some added risks or discomforts from study procedures. In addition to the risks listed below, there may be risks that have not appeared before. You should contact the Study Site Leader as soon as possible if you are concerned about anything while you are part of the study.

Possible loss of privacy (confidentiality)
When we share your information and study samples, there is a small risk that people may get to see it who are not supposed to. Researchers will do their best to protect your information by keeping study information and study samples in safe places and separate from your name and contact information. Researchers using your study samples must agree not to try to find out who you are. However, there is a small chance they may be able to find out who you are.

The research team may communicate with you electronically, such as over email or text message. While the researchers will take steps to protect your privacy and confidentiality, text messages are not encrypted and not a secure mode of communication, and there is a risk that people may see the messages who are not supposed to. The researchers will only send limited information in these messages.

Findings about Your Genes that Were Not Planned or Not Part of the Main Study
It is possible that during the study, we may do tests on your saliva that find out things out about your genes. You can decide whether you want to be told about these things. There can be a risk in knowing these results. For example, researchers may look at the full set of genes in your body, including looking at the exact order of DNA, the building block of the genes. This is called whole genome sequencing. New information may be found that show that you have genes that make it more likely that you will have certain health problems. Knowing this information can be stressful, lead to worry and affect your relationship with your family. There may also be good things about (benefits) knowing about how genes might affect your health; sometimes you can change your habits or have screening tests that will help you from getting a disease or help you treat a disease earlier.

Group Risks for Gene Testing
Although we will not give researchers your name, we will give them basic information such as your race, ethnic group, and sex. This information helps researchers learn whether the factors that lead to health problems are the same in different groups of people. It is possible that such findings could one day help people of the same race, ethnic group, or sex as you. However, they could also be used to support harmful stereotypes, stigmatize, or discriminate against members of a socially defined group such as race or ethnicity.

7. Can you be in the study if you are pregnant or breastfeeding?

You can be part of this study if you are pregnant or breastfeeding. Being part of this study does not involve any tests in which there is radiation exposure, or any tests that would not be safe during pregnancy or for an infant that is drinking your breast milk.

8. What if new information becomes available?

During this study we may find information that could be important to you. This includes information that might cause you to change your mind about being in the study. We will let you know as soon as possible if this kind of information is found.

New findings from genetic research
In this part of the form, we are telling you about new information that may be discovered in genetic research tests that will be done in this study. DNA is the material that makes up your genes. Researchers plan to do research on the DNA in your genes while you are in the study, and also in the future after you have finished all of the study visits.

Approved For Period: 12/4/2022 - 8/31/2023

The genetic testing for the study is being done for research, but the study doctors might find a change in your gene DNA that may increase risk of certain diseases or health conditions for you and your family. You can choose whether or not you would like to know about the results of the genetic testing.
If a change in your gene DNA that may increase risk for certain diseases or health conditions is found, the study doctor will double-check this result in a certified clinical laboratory to determine whether or not there is any possible impact on the health of you or your family. There is no cost to you for double-checking the result in a certified clinical laboratory. If a result that could affect your health is found on the certified clinical laboratory test, the study doctors will speak to you in person or on the telephone to explain the new information within 10 days after finding the new information. If you choose to know about these genetic test results, it is important for you to know that results from a certified clinical laboratory may also be included in your medical record, which means that anybody who is allowed to see your medical record, including your health care providers outside of the study, will be able to see the test results. Your health care providers may order more tests or treatments that are not part of the study, and may ask you to meet with a genetic counselor to help you understand the results of the genetic test. You will be financially responsible for any tests, treatments, and counseling ordered by your health care provider that are not part of the study.

It is possible that you may have other changes in gene DNA that we do not come across as part of this research. If you would like to have a complete review of your genetic information, we recommend that you undergo further genetic testing in a certified laboratory and seek genetic counseling to help you understand the results of the testing.

Please initial one of the options below to confirm whether you would like to be informed of findings from study gene testing:

Please initial next to your choice below:

_____ Yes, I would like to be told about gene test results that might affect my health.
Initials here

_____ No, I would not like to be told about gene test results that might affect my health.
Initials here

9. What are the possible good things (benefits) from being part of the study?

We do not expect that your health will get better from being a part of this study. The results of the study will be important in helping patients, caregivers, and parents understand how COVID affects the body long-term.

10. What else can I do if I decide not to be part of the study?

You do not have to be part of this research study. You do not have to take part in this research study to be treated for infection with the virus that causes COVID-19 (SARS-CoV-2 infection). If you decide not to participate in the study, what you decide will not affect your health care in the future, how you pay for health care or if you can get health insurance.

11. Will I be paid for being in this study?

You will not receive payment for participation in this study.

Approved For Period: 12/4/2022 - 8/31/2023

12. Will I have to pay for anything?

There are no costs to you related to your being part of the study. Money from the National Institutes of Health (NIH) will cover the costs of your being part of the study. You or your insurance company will not be asked to pay for the costs of your visits related to the study. You and/or your health insurance may be billed for the costs of health care during this study if these costs would have happened even if you were not in the study. If your insurance does not cover these costs or you do not have insurance, these costs will up to you to take care of.

13. What happens if you are hurt from being in the study?

For emergencies, call 911. If you think you have been hurt because of being part of this research study, tell the Study Site Leader as soon as you can. The Study Site Leader's name and phone number are listed at the top of page 1 of this consent form.

If you are hurt because of being part of this research, your study site staff can help you find a doctor to give you treatment if you want. We may ask your insurance company, or someone else, if appropriate, to pay for the costs of the treatment due to your being hurt, but you may also need to pay for some of this cost.

There are no plans for the [study site] or NYU Grossman School of Medicine or NYU Langone Health to pay you or give you anything else for being hurt. You do not give up the rights you have under the law by signing this form.

14. When is the study over? Can I leave the study before it ends?

This study will last for 4 years. Your participation in this study will include one visit. This study may be stopped early. It is also possible that you may be taken out of the study for the following reasons:

- The researchers in charge of the study feel it is important to do this for your health or safety.
- You have not followed study instructions.
- The group funding the study, the main researchers in charge, or other group whose job it is to be in charge of looking at the safety of the study decide to stop the study.
- More information about Long COVID is known so that the study is no longer needed.

At any time, if you do not wish to continue to be in the study, you are free to leave the study. Leaving the study will not affect you or your care, how your health care is paid for, or what kind of health insurance you can get.

15. How will we protect your confidentiality (privacy)?

Your medical information is also called protected health information, or "PHI", because it is protected by federal and state laws, such as the Health Insurance Portability and Accountability Act, or HIPAA. This includes information in your research record as well as information in your health record at the place where you signed up to be part of your research (your study site (XXX)). In following the rules of your study site and with HIPAA, only those people who have a reason to look at your health information because of their job can look at this information.

Medical information created by this research study may become part of your medical record. We may include your research information in your medical record for several reasons, including for the billing of services provided in connection with the study, to securely document any medical services you receive, and so that other members of the NYU Langone Health community who may treat you have access to important information about your health.

You have a right to look at the information in your health record. In some cases, when it is needed to make sure the research is done in the best way possible so that results can be trusted, you will not be

Approved For Period: 12/4/2022 - 8/31/2023

able to see or make a copy of certain information about the study while the study is going on, but you will have the right to see and copy the information once the study is over in line with your research center's policies and the law.

Certificate of Confidentiality
To help us further protect your privacy (confidentiality), this research is covered by a Certificate of Confidentiality from the National Institutes of Health (NIH). This adds special protection for the research information (data, documents, or biospecimens) that may identify you.

Research information protected by this Certificate of Confidentiality cannot be disclosed to anyone else who is not connected with the research, without your consent. With this Certificate of Confidentiality, the researchers may not disclose or use research information that may identify you in any federal, state, or local civil, criminal, administrative, legislative, or other action, suit, or proceeding, or be used as evidence, for example, if there is a court subpoena, without your consent. However, disclosure, without your consent, is still necessary if there is a federal, state, or local law that requires disclosure (such as to report child abuse or diseases caused by germs that can be spread to others).

The Certificate of Confidentiality cannot be used to refuse a request for information from appropriate government agencies responsible for the project.

By agreeing to be in this research and signing below, you are giving your consent (permission) to share research information with others at NYU Langone Health and other researchers who are working together with NYU Langone Health who are listed in the next section. This means that your research information, including lab results, may be included in your medical record.

16. HIPAA Authorization

As noted in the Confidentiality part of this consent form above, federal laws in the U.S. have been made so that we, and the researchers working with us, health care providers, and the people who care for you protect the privacy of information that identifies you (used to know who you are) and relates to your past, present, and future physical and mental health problems. We are asking for your permission (authorization) to use and share your health information with others related to this study, in other words, in order for this research to happen, including doing and watching over the study.

Your treatment outside of this study, payment for your health care, and your health care benefits will not be affected even if you do not give permission (authorize) the use and sharing of your information for this study.

Who can use and share information in connection with this study?
The following individuals may use, share, or get your information for this research study:

- The research team, including the Study Site Leader and other people helping with the study or who are in charge of watching over the study at the place where you signed up to be part of the research study
- The researchers at NYU Grossman School of Medicine, who are in charge of helping with and watching over the study at all the places across the country where the study is happening
- Non-research staff who need this information to do their jobs (such as for treatment, payment, or health care operations)
- The researchers at Massachusetts General Hospital, who are in charge of storing the information for this study, the researchers at Mayo Clinic who are in charge of the research biorepository, and other RECOVER study centers or national centers in charge of storing research information
- The group that funded the study: National Institutes of Health (National Heart Lung and Blood Institute)
- The ethical review board (also called institutional review board or IRB) that oversees the research and the research quality improvement programs

Approved For Period: 12/4/2022 - 8/31/2023

- The group that is watching over the safety of patients and families in the study (called the Observational Study Monitoring Board). The National Institutes of Health decides who will be in this expert group
- A company hired to oversee the quality of the RECOVER research information ((Biomedical Research Alliance of New York)
- People or groups that we hire to do work for the study, such as fitness tracker companies, smartphone application companies, data storage companies, insurers, and lawyers
- Governmental agencies in charge of watching over the research (for example, the US Department of Health and Human Services)
- Health care providers, including your doctors and others who care for you related to this study, and laboratories or other people who are looking at your health information as part of this study

Your information may be re-disclosed (shared) or used for other reasons if the person who gets your information is not required by law to protect the privacy of the information.

What if you do not want to give permission to use and share your information for this study?
Signing this form is voluntary. You do not have to give us permission to use and share your information, but if you do not, you will not be able to be part of this study.

Can you change your mind and withdraw (take back) permission to use or share your information?
Yes, you may take back your permission to use and share your health information at any time for this research study. If you choose to take back permission, you can choose to stop collection of new information, or request that previously collected information, including biological samples, be removed from the study. If you take back your permission, we will not be able to take back information that has already been used or shared with others. To take back your permission, send a written notice by mail or email to the Study Site Leader noted at the top of page 1 of this form. If you take back your permission, you will no longer be able to stay in this study.

How long can your information be used or shared?
Your permission to use or share your personal health information for this study will never **end** unless you take it back.

17. What is an Electronic Medical Record?

An Electronic Medical Record (EMR) is an electronic version of your medical chart, or health record. An EMR is simply a computer version of a paper health record.

Having this EMR will let researchers keep information about you related to your being part of this research study. You must agree to make this EMR if you want to be part of this study. In order to make your EMR, the study team will need to get basic information about you that would be similar to the information you would provide the first time you visit a hospital or other place you get health care, for example, your name, the name of your main doctor, the type of insurance you have, your date of birth and other health-related information.

What information may be put in the EMR?
Information related to your being part of the research will be put in your EMR at your study site listed on the first page.

This information can be seen by people who work at your study site who are not part of the research team. Information that is in your EMR may also be shared with others who your study site has decided should be able to look at your EMR (for example, health insurance company, disability provider, etc.).

Will you be able to look at research-related information within the Electronic Medical Record?
A law called the “21st Century Cures Act” makes it easier for patients to look at their EMR.

Approved For Period: 12/4/2022 - 8/31/2023

As part of this research study, some research-related information will be put in your EMR and you will be able to see it right away. You may not be able to see some other research-related information until the end of the study.

18. The Institutional Review Board (IRB) and how it protects you

The Institutional Review Board (IRB) reviews all research involving people before it can be started and then as long as the study continues. The main concern of the IRB is to protect the people who are part of the study. For questions about your rights while you are part of the study, contact the NYU IRB Office number on (212) 263-4110. The NYU School of Medicine's IRB and other IRBs that may be part of this study are made up of doctors, nurses, scientists, and people from the community.

19. Who can I call with questions, or if I am worried about my rights as a research subject?

You can call the IRB with your questions or concerns. Our telephone numbers are listed below. Stuart Katz, MD is the person in charge of this research study. His name and phone number are listed on the first page of this form. If you want to speak with someone not directly involved in this research study, please contact [insert name of contact or IRB]. You can call them at [insert contact information]. You can talk to them about:

- Your rights as a research participant
- Your concerns about the research
- A complaint about the research. Also, if you feel pressured to take part in this research study, or to continue with it, they want to know and can help.

Approved For Period: 12/4/2022 - 8/31/2023

When you sign this form, you are agreeing to take part in this research study as described to you. This means that you have read the consent form, your questions have been answered, and you have decided to volunteer.

Name of Participant (Print)	Signature of Participant	Date

Name of Person Obtaining Consent (Print)	Signature of Person Obtaining Consent	Date

Signature of Parent(s)/Guardian for subject who has not yet reached the age of majority in their state. I give my consent for my child to take part in this research study and agree to allow his/her health information to be used and shared as described above.

Name of Parent (Print)	Signature of Parent	Date

Approved For Period: 12/4/2022 - 8/31/2023

Witness to Consent Process for Non-English-Speaking Subjects (using a translated consent form OR "Short Form" in Subject's Spoken Language)

Statement of Witness
As someone who understands both English and the language spoken by the subject, I listened to and heard the research team member talk about the information in the English version of the consent form with the subject in the subject's own language, and that the subject was given the chance to ask questions.

Name of Witness (Print)	Signature of Witness	Date

Witness to Consent of a Subject Who Cannot Read or Write

Statement of Witness
I represent that the consent form was presented orally to the subject in the subject's own language, that the subject was given the opportunity to ask questions, and that the subject has shown their consent and authorization for being part of the study by (check box that applies).

☐ Subject making his/her own "X" above in the subject signature line
☐ Subject showed approval for being part of the study in another way; describe:

Name of Witness (Print)	Signature of Witness	Date

Approved For Period: 12/4/2022 - 8/31/2023
