# Supplementary material for: Researching COVID to enhance recovery (RECOVER) pediatric study protocol: Rationale, objectives and design
Source: PLoS One. 2024 May 7;19(5):e0285635. doi: 10.1371/journal.pone.0285635 (PMC11075869; doi:10.1371/journal.pone.0285635)
Supplement: S1 Data — (ZIP) [file pone.0285635.s024.zip › Jernigan_Letter_for_PLOS_One.pdf]

NYU Grossman School of Medicine

Dear Editorial Team,

Re:

MS ID#: PONE-D-23-10495R1

MS Title: Researching COVID to enhance recovery (RECOVER) pediatric study protocol: Rationale, objectives and design

The following individuals should be recognized as co-authors on this manuscript, we kindly request for them to be added to the author list.

Tamara Bradford
Maryanne Chrisant
Audrey Dionne
Stephanie Handler
Keren Hasbani
Camden Hebson
Kimberly McHugh
Julie Miller
Elizabeth C. Mitchell
Onyekachukwu Osakwe
Michael A. Portman
S. Kristen Sexson Tejtel
Shubika Srivastava
Felicia Trachtenberg

Kind Regards,

DocuSigned by: Rachel S. Gross
CFD201852640450...

3/5/2024

Rachel Gross, MD, MS

Date

Author	Signature	Date
Terry L. Jernigan	DocuSigned by: Terry L. Jernigan 0D5744A40E074D1...	3/5/2024

NYU RECOVER Clinical Science Core
462 First Avenue, 8th Floor New York, NY 10016 | Rachel.Gross@nyulangone.org
